# Visible-light promoted regioselective amination and alkylation of remote C(sp^3^)-H bonds

**DOI:** 10.1038/s41467-020-15167-2

**Published:** 2020-03-19

**Authors:** Quanping Guo, Qiang Peng, Hongli Chai, Yumei Huo, Shan Wang, Zhaoqing Xu

**Affiliations:** Institute of Drug Design & Synthesis, Institute of Pharmacology, Key Laboratory of Preclinical Study for New Drugs of Gansu Province, School of Basic Medical Science, Lanzhou University, 199 West Donggang Road, Lanzhou, 730000 China

**Keywords:** Reaction mechanisms, Synthetic chemistry methodology, Photocatalysis

## Abstract

The C-N cross coupling reaction has always been a fundamental task in organic synthesis. However, the direct use of N-H group of aryl amines to generate N-centered radicals which would couple with alkyl radicals to construct C-N bonds is still rare. Here we report a visible light-promoted C-N radical cross coupling for regioselective amination of remote C(sp^3^)-H bonds. Under visible light irradiation, the N-H groups of aryl amines are converted to N-centered radicals, and are then trapped by alkyl radicals, which are generated from Hofmann-Löffler-Freytag (HLF) type 1,5-hydrogen atom transfer (1,5-HAT). With the same strategy, the regioselective C(sp^3^)-C(sp^3^) cross coupling is also realized by using alkyl Hantzsch esters (or nitrile) as radical alkylation reagents. Notably, the α-C(sp^3^)-H of tertiary amines can be directly alkylated to form the C(sp^3^)-C(sp^3^) bonds via C(sp^3^)-H − C(sp^3^)-H cross coupling through the same photoredox pathway.

## Abstract (web summary)

C-N bond forming is an established strategy to form amines, which are quintessential in chemical synthesis and in nature. Here, the authors report three classes of photoredox reactions, involving C(sp^3^)-N coupling between N-centered radicals and alkyl radicals and C(sp^3^)- C(sp^3^) coupling via C(sp^3^)-H alkylation.

## Introduction

**Fig. 1:**
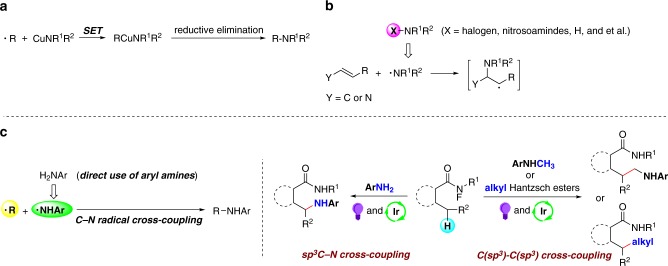
sp^3^ C–N bond formations with N-center radicals. **a** Cu-catalyzed radical sp^3^ C–N cross-couplings^6–8^. **b** Addition reactions between N-center radicals and alkenes (enamines)^9^. **c** This work: sp^3^ C–N cross-coupling between alkyl radical and N-center radical. X = halogen, nitrosoamides, H, and so on. Y = C or N.

Amines are quintessential moieties in pharmaceuticals, nature products, and organic materials^1,2^. In the past few decades, transition-metal-catalyzed sp^2^ C–N couplings of aryl halides (and pseudo halides) with amine nucleophiles have been well developed, such as Buchwald–Hartwig reaction^3^, Ullmann coupling^4^, and Chan–Lam amination^5^. However, the alkylation of amines using alkyl electrophiles is largely underdeveloped due to the β-hydrogen elimination from the metal-alkyl intermediate^6–8^. Recently, significant progress has been made in transition-metal-catalyzed radical sp^3^ C–N bond formations. Fu and coworkers recently disclosed the photoinduced, Cu-catalyzed intermolecular and intramolecular alkylation of amides^9,10^. Very recently, Macmillan^11^ and Hu^12^ reported a series of Cu-catalyzed, photoinduced decarboxylative sp^3^ C–N coupling reactions, respectively. In these approaches, the trapping of alkyl radicals by Cu-amine species and the reductive eliminations of Cu intermediates were key steps for the cross-couplings (Fig. 1a).

In the past few years, the addition reactions of N-center radical to alkenes (or enamine intermediates) have been developed (Fig. 1b)^13–20^. However, the direct cross-coupling between alkyl- and N-based radicals in the absence of stabilization by transition-metal complex has been rarely explored. Moreover, in the reactions, the amine (or amide) compounds need to be converted to the corresponding N-radical precursors (e.g., N-halogens and N-nitrosoamides) by separated steps. The direct use of the N–H group of aryl amines to generate N_aryl_-center radicals and couple with alkyl radicals is still rare^17^.

The regioselective C–H functionalization is one of the most fundamental reactions in organic synthetic chemistry. In recent years, the functionalization of C(sp^3^)–H bonds has become an important and intensive task to the organic synthetic community. In the past decade, great progress has been achieved in C(sp^3^)–H functionalization at unactivated sites, which allows streamlined synthesis of target compounds and late-stage modification of complex structures. Recently, the application of Hofmann–Löffler–Freytag (HLF)-type 1,5-hydrogen atom transfer (1,5-HAT) in C(sp^3^)–H functionalization reactions received much attention due to their unique regioselectivities^21,22^. Although the amidyl radical formation and its subsequent 1,5-HAT process have been well established, the followed transformations of the C-center radical are still limited, and the reactions mainly focused on cyclization^23–29^, atom transfer (halogenation)^30–33^, Giese reaction^34–37^, azidation^38,39^, cyanation^40,41^, trifluoromethylation^42^, and arylation)^43,44^. So far, using the HLF-type C-center radical for sp^3^ C–N or C(sp^3^)–C(sp^3^) couplings is still very rare^45^, and the direct cross-coupling between C(sp^3^)–H and N–H is not realized. We here report an example of sp^3^ C–N cross-coupling reaction between N-center- and alkyl radicals. Notably, the aryl amines are directly converted to N-center radicals under visible-light irradiation (Fig. 1c). By using the same photoredox catalytic 1,5-HAT strategy, the regioselective C(sp^3^)–H alkylation can also be realized when Hantzsch esters are used as alkylation reagents. The primary, secondary, and tertiary alkylation are all compatible under standard conditions. It is worth noting that the α-C(sp^3^)–H of tertiary amines can be directly alkylated to form C(sp^3^)–C(sp^3^) bonds without pre-functionalization.

## Results

### Investigation of the sp^3^ C–N coupling reaction conditions

The investigation was initiated by using *N*-(tert-butyl)-*N*-fluoro-2-methylbenzamide (**1a**) and aniline (**2a**) as model substrates. A series of photocatalysts (Ir and Ru complexes, or organic photocatalysts), light sources, solvents, additives, and the substrate ratios were tested (see the Supplementary Information for details). The results indicated the optimal reaction conditions (condition A): under 24-W violet LED (390–410 nm) irradiation, **1a** and **2a** (3 equiv) were dissolved in DMF (0.1 M), Ir(ppy)_2_(dtbpy)PF_6_ (1 mol %) was used as the photocatalyst, K_2_CO_3_ (3.0 equiv) was used as basic additive, and the reaction was stirred at room temperature for 12 h. Under these reaction conditions, the desired sp^3^ C–N coupling product (**3a**) was isolated in 73% yield. Notably, to achieve this transformation, a suitable photocatalyst with well-balanced redox potential was required. The organic photocatalysts **A**–**D** have relatively strong oxidative properties, whereas the reductive activities were moderate. In contrast, the Ir- and Ru-based photocatalysts have good redox abilities, which were compatible for the reaction (Table 1).

**Table 1:** Optimization of reaction conditions^a^.


Entry	Change to “condition A”	Yield (%)^b^
1	Condition A	73
2	No light	0
3	No photocatalyst	0
4	Without K_2_CO_3_	0
5	A,B,C,D instead of G	0
6	E, F, H instead of G	20–64
7	Other bases instead of K_2_CO_3_	0–49
8	Other solvents instead of DMF	0–55

^a^Unless noted, the reactions were carried out using **1a** (0.1 mmol), **2a** (3 equiv), photocatalyst (1 mol %), base (3.0 equiv), and DMF (1 ml), under Ar, and stirred at rt for 12 h under 24-W violet LED irradiation.

^b^Isolated yields.

### Scope of sp^3^ C–N coupling reactions

**Fig. 2:**
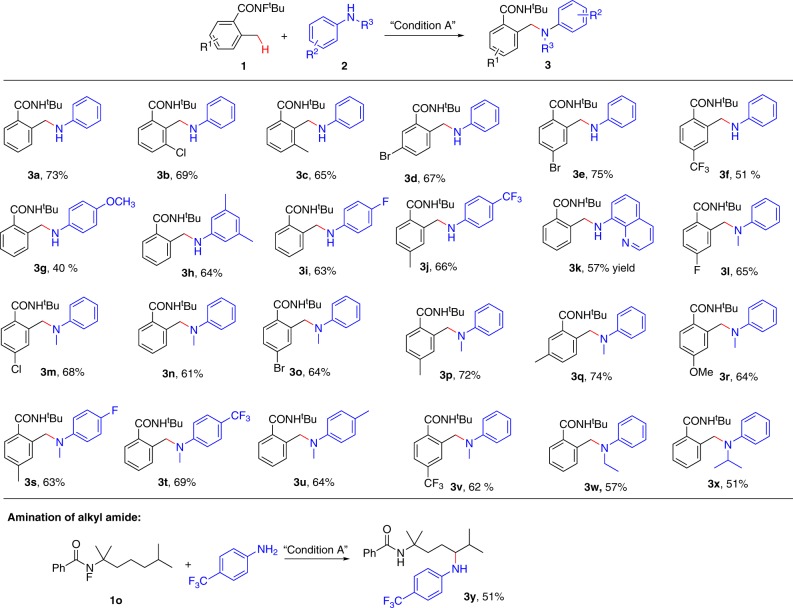
Substrate scope of sp^3^ C–N coupling reactions. All reactions were conducted in 0.2 mmol scale. Yields referred to isolated yields.

With the optimal reaction conditions in hand, the substrate scope of carboxylamides and anilines was examined, and the results are summarized in Fig. 2. To our delight, the carboxamides and anilines bearing electron-donating and electron-withdrawing groups at *o*-, *m*-, or *p*-position of the aryl ring were compatible with moderate-to-good yields (Fig. 2, **3a**–**3x**). A range of functional groups, such as –CH_3_, –OCH_3_, –CF_3_, and halides (–F, –Cl, and –Br) were all tolerated. The bulkier amines, such as EtNHPh and i-PrNHPh, were successfully converted to the corresponding products **3w** and **3x** with satisfactory yields, respectively. Notably, the amination of alkyl amide was also achieved under standard conditions (**3y**). However, the alkyl-substituted amines, such as CyNH_2_ and n-Bu_2_NH, failed to give the corresponding amination products.

### Alkylation of tertiary amine α-C(sp^3^)–H bonds

Tertiary amine motifs are widely represented in many pharmaceuticals and advanced materials^1,2^. Direct functionalization of tertiary amine provides an efficient pathway to synthesize structurally diversified tertiary amines. In 2006, Li and coworkers reported a cross-dehydrogenative-coupling reaction, which could directly couple the α-C(sp)^3^–H of tertiary amines with nucleophiles under oxidative conditions^46–49^. Very recently, the photoredox-induced α-C(sp)^3^–H functionalization of tertiary amines was achieved, which could functionalize the α-C(sp)^3^–H under mild and external oxidant-free conditions^50,51^. Despite these achievements, the direct C(sp^3^)–C(sp^3^) cross-coupling reactions between tertiary amines α-C(sp^3^)–H and unactivated C(sp^3^)–H were still not realized.

**Fig. 3:**
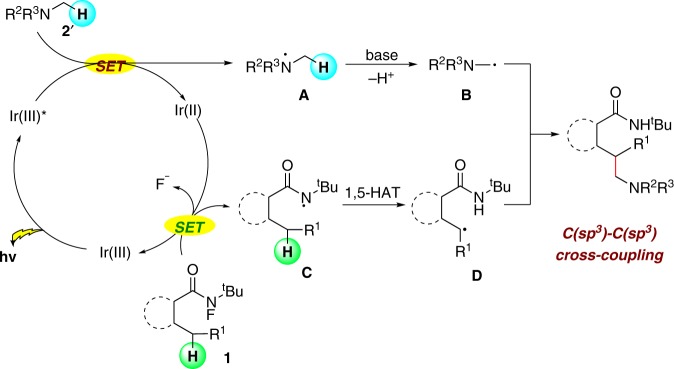
Design plan for alkylation of tertiary amine α-C(sp^3^)–H bonds. Hypothesis of the mechanism for photoinduced C(sp^3^)–C(sp^3^) coupling reactions.

Encouraged by the success of photoredox sp^3^ C–N coupling, we decided to explore an alternate route to realize regioselective C(sp^3^)–C(sp^3^) coupling between tertiary amines α-C(sp^3^)–H and unactivated C(sp^3^)–H using the photoredox 1,5-HAT strategy. In our initial hypothesis, upon irradiation, the high valent photocatalyst could accept an electron from amine and simultaneously generate an amino radical cation **A** through single-electron transfer (SET) process (Fig. 3). The amino radical cation would then form α-amino alkyl radical **B** by deprotonation. The intermediate **B** could be captured by C-center radical **D** that was generated through 1,5-HAT, and furnished the C(sp^3^)–C(sp^3^) coupling.

### Scope of alkylation of tertiary amine α-C(sp^3^)–H bonds

**Fig. 4:**
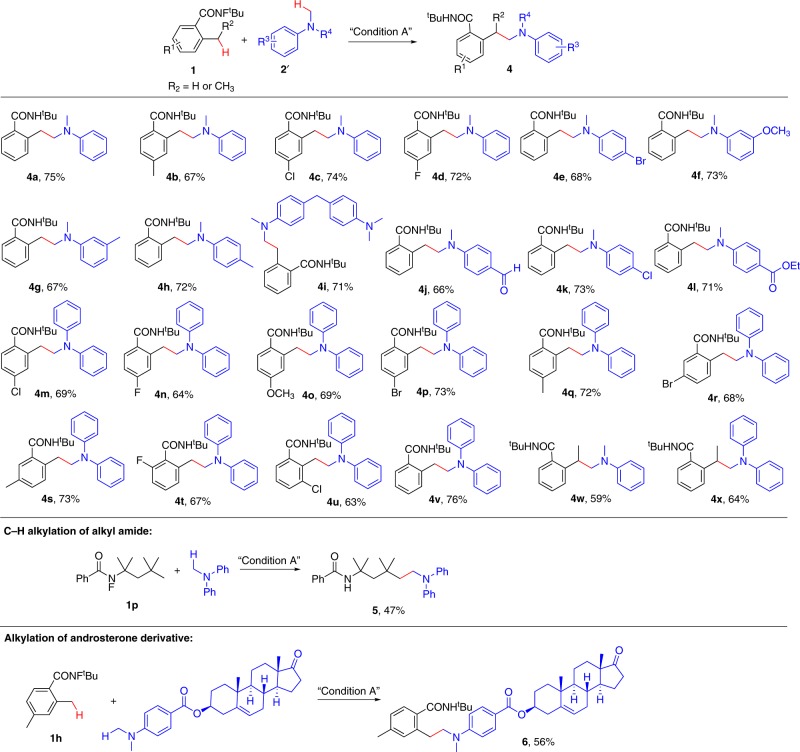
Substrate scope of alkylation of tertiary amine α-C(sp^3^)–H bonds. All reactions were conducted in 0.2 mmol scale. Yields referred to isolated yields.

With the above hypothesis in mind, we began our study by using **1a** and *N*,*N*-dimethylaniline (**2a’**) as model substrates to optimize the reaction conditions (see the Supplementary Information for details). Under the optimal conditions (condition A), the desired C(sp^3^)–C(sp^3^) cross-coupling product (**4a**) was obtained in 75% yield. The generality of the reaction was examined by using a variety of tertiary amines and carboxamides (Fig. 4, **4a**–**4o**). To our delight, uniformly good results were obtained with various substrates bearing sensitive functional groups. The C–H functionalization of alkyl amide was also realized with good yield (**5**). Furthermore, the late-stage modification of androsterone-derived amine was achieved in 56% yield with the ester group untouched **6**.

### C(sp^3^)–C(sp^3^) coupling reaction using alkyl Hantzsch ester

In the past few years, the HLF-type radical cross-coupling reactions were intensively studied^21–44^. However, its application in the construction of C(sp^3^)–C(sp^3^) was still rare^45^. Hantzsch esters were first synthesized by A. R. Hantzsch in 1881, and widely used in pharmaceutical chemistry. With the rapid development of radical chemistry, various alkylation reactions using 4-substituted Hantzsch esters as alkylation reagent have been developed^52–55^. However, the cross-coupling between alkyl Hantzsch esters and C(sp^3^)–H was still not realized.

In the above successful C(sp^3^)–H alkylation reactions (Fig. 4), the alkyl radicals were generated through 1,2-SET of N-center radical, which restricted the scope of alkyl substrates. Alkyl Hantzsch ester has the ability to serve both as a single-electron reductant and alkyl radical precursor. We envisioned that alkyl Hantzsch esters could be used instead of tertiary amine as the alkylation reagents for the direct C(sp^3^)–C(sp^3^) cross-coupling.

### Scope of C(sp^3^)–C(sp^3^) coupling

**Fig. 5:**
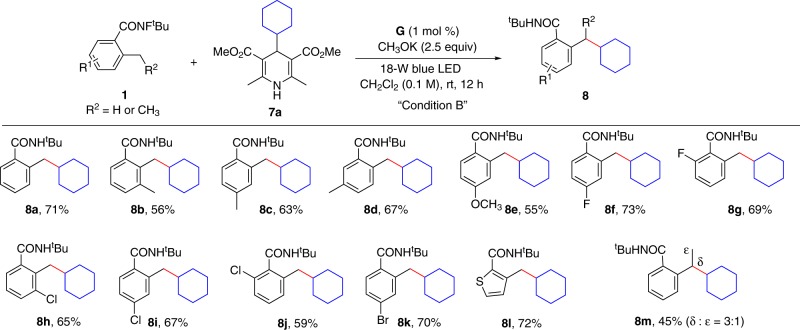
Substrate scope of carboxylamides with cyclohexyl Hantzsch ester. All reactions were conducted in 0.2 mmol scale. Yields referred to isolated yields.

Initially, cyclohexyl Hantzsch ester (**7a**) was used as model substrate to optimize the reaction conditions. After the screening of reaction parameters, the desired C(sp^3^)–C(sp^3^) cross-coupling product (**8a**) could be obtained in 71% yield (condition B, see the Supplementary Information for details). Then, the substrate scope of carboxylamides was examined (Fig. 5). To our delight, the carboxamides bearing electron-donating and electron-withdrawing groups at *o*-, *m*-, or *p*-position of the aryl ring were compatible with moderate-to-good yields (**8a**–**8l**). A range of functional groups, such as –CH_3_, –OCH_3_, and halides (–F, –Cl, and –Br), were all tolerated (**8a**–**8k**). The thiophene-derived substrate delivered the desired product with 72% yield (**8l**). The regioisomers were found in the case of **8m**, which might be attributed to the competing 1,6-HAT pathway^32,56^.

**Fig. 6:**
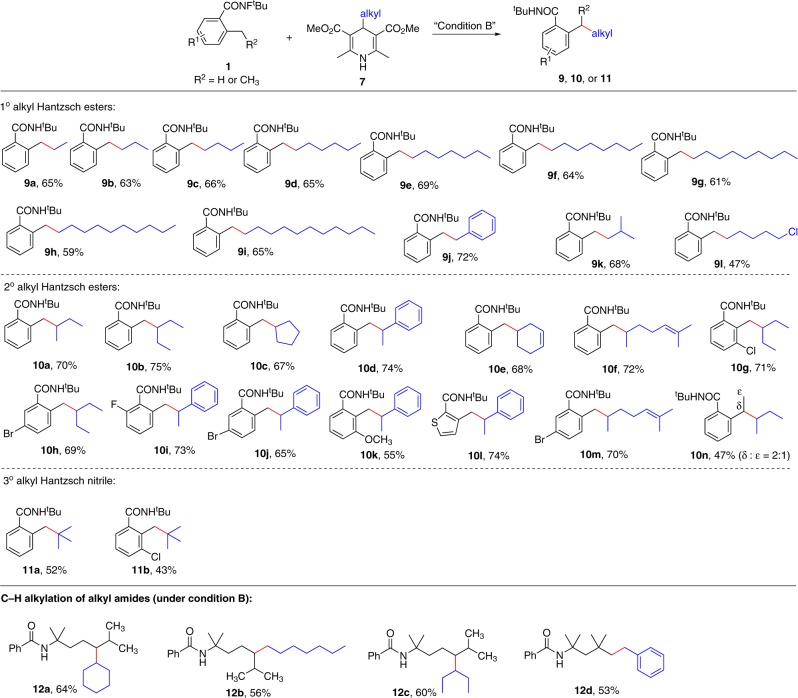
Substrate scope of alkyl Hantzsch esters and nitrile. All reactions were conducted in 0.2 mmol scale. Yields referred to isolated yields.

To further explore the substrate scope, a variety of alkyl Hantzsch esters were examined (Fig. 6). To our delight, the primary and the secondary alkyl Hantzsch esters, as well as the tertiary alkyl Hantzsch nitrile, all proceeded smoothly in satisfactory results (**9a**–**11b**) with the sensitive functional groups (halogens and alkenes) untouched. The results indicated the general ability of our strategy for the construction of C(sp^3^)–C(sp^3^) bonds in the synthetic chemistry. Notably, the aryl Hantzsch esters failed to give any desired products under our standard conditions. It should be noted that our method was suitable not only for *o*-methylbenzamide, but also alkyl amide. As shown in Fig. 6, under the standard reaction conditions, **1o** and **1p** were smoothly coupled with alkyl Hantzsch esters in satisfactory yields (**12a**–**12d**).

### Synthetic applications

**Fig. 7:**
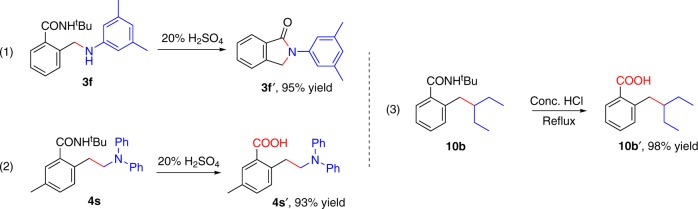
Synthetic applications. (1) Synthesis of lactam **3f’**. (2) and (3) Converted amides to acids.

To demonstrate the synthetic application of our method, the amination and alkylation products were readily converted to the corresponding lactam (**3f’**) and acid (**4s’** and **10b’**) through simple operations with excellent yields, respectively (Fig. 7).

### Mechanistic investigations

**Fig. 8:**
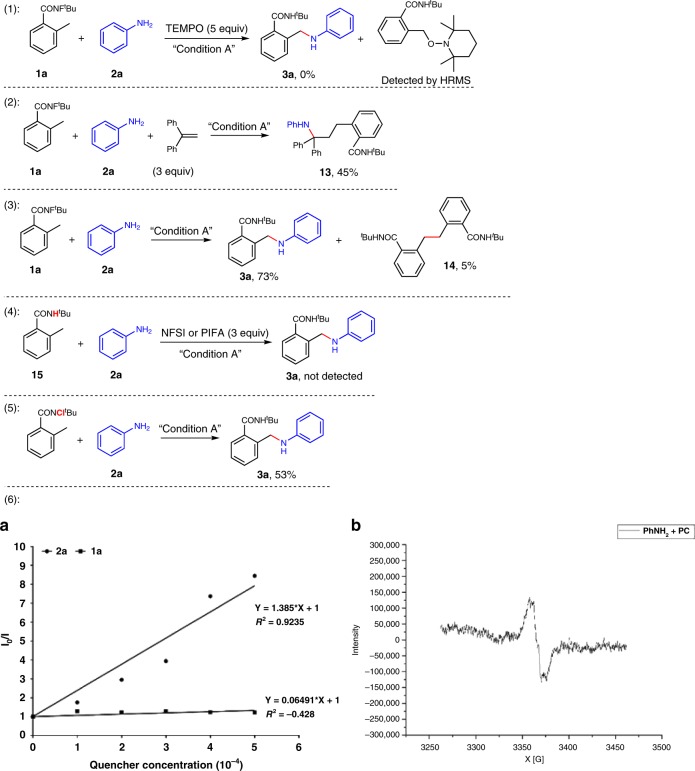
Mechanistic studies. Equation (1) Radical trapping reaction with TEMPO. (2) Radical trapping reaction with ethane-1,1-diyldibenzene. (3) Homo-coupling product. (4) The results under oxidative conditions. (5) The result with *N*-chloroamide substrate. (6) (**a**) Fluorescence quenching of Ir(ppy)_2_(dtbpy)PF_6_ by **2a** and **1a**. (**b**) EPR experiment result of PhNH_2_ (**2a**, PhNH_2_ (0.1 mmol) and Ir(ppy)_2_(dtbpy)PF_6_ (5 mol%) in hexafluoroisopropanol (1 ml), stirred at room temperature for 1 h under 400-nm irradiation, and directly used for EPR experiments).

In order to gain some mechanistic insight of this sp^3^ C–N coupling reaction, several control experiments were carried out (Fig. 8). The reaction was completely shut down by 2,2,6,6-tetramethylpiperidin-1-oxyl (TEMPO, Equation (1)). Furthermore, when the radical scavenger ethane-1,1-diyldibenzene was added to the reaction, the corresponding three-component-type product **13** was obtained in 45% yield (Equation (2)). When **1a** and aniline (**2a**) were used as substrates, the desired product **3a** was isolated in 73% yield. Notably, the homo-coupling product **14** was also obtained in the reaction system with 5% yield (Equation (3)). These results suggested that a) the radical pathway might be involved in the reaction; b) the HLF-type 1,5-HAT proceeded in the system and formed the C-center radical; c) aryl amine possibly converted to the corresponding N-center radical under standard conditions; d) the radical–radical coupling route might be responsible for this sp^3^ C–N bond formation reaction. We also tried this reaction under oxidative conditions. In the presence of *N*-fluorobenzenesulfonimide (NFSI, 3 equiv)^43,44^ or [bis(trifluoroacetoxy)iodo]benzene (PIFA, 3 equiv)^57^, the un-fluoride amide substrate **15** failed to produce the amination product under standard conditions (Equation (4)). In addition, the *N*-chloroamide could also give the desired product with modest yield (Equation  (5)). These results indicated that the pre-activation of the substrates is crucial to this coupling reaction. In our initial hypothesis, the step that aryl amine converts to the corresponding N-center radical was crucial for this transformation. To verify this hypothesis, emission quenching and electron paramagnetic resonance experiments have been conducted, and the results indicated that the radical species was generated in the system (Equation (6)), see the Supplementary Information for details). The Stern−Volmer plot showed strong quenching of Ir(ppy)_2_(dtbpy)PF_6_ (*E*_1/2_*^III/II^ = +0.66 V vs. SCE) by PhNH_2_ (**2a**) (*E*_1/2_^red^ = +0.94 V vs. SCE), favoring a reductive quenching cycle. These evidences indicated that the excited-state Ir(ppy)_2_(dtbpy)PF_6_ might undergo a SET process that furnished the formation of N_aryl_-center radical.

### Proposed mechanism

**Fig. 9:**
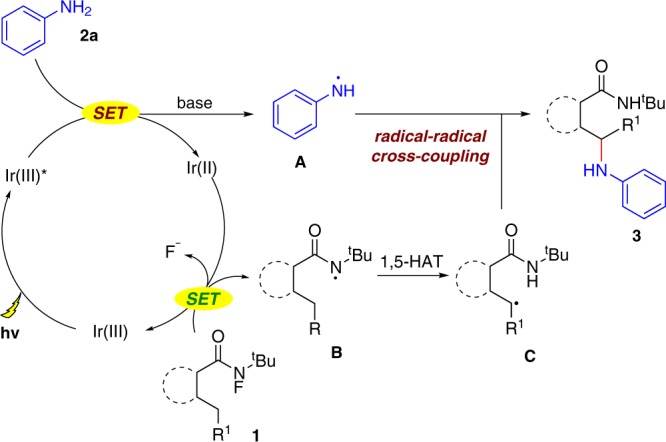
Proposed mechanism. Proposed mechanism for photoinduced C(sp^3^)–N coupling reactions.

Based on our investigations and previous reports, a plausible mechanism is proposed in Fig. 9 (see the Supplementary Information for details). The reaction starts with the oxidation of **2** by the excited-state Ir(III)* in the presence of a base, yielding amine radical **A** and Ir(II). Then, the Ir(II) (*E*_1/2_^III/II^ = −1.51 V vs. SCE)^58,59^ species facilitated the second SET process of substrate **1** (*E*_p_^0/–1^(**1a**) = –0.84 V vs. SCE in MeCN) to generate the amidyl radical **B**. The subsequent 1,5-HAT formed the radical intermediate **C** along with the oxidation of Ir(II) to Ir(III) to close the catalytic cycle. Finally, the radical–radical cross-coupling between N-center radical **A** and C-center radical intermediate **C** was proposed to provide the sp^3^ C–N cross-coupling product **3**.

## Discussion

In conclusion, we disclosed a visible-light-promoted C–N-radical cross-coupling to realize the regioselective amination of remote C(sp^3^)–H bonds. In the reactions, the N-center radicals were directly generated from aryl amines under visible-light irradiation. Using the photoinduced HLF-type 1,5-HAT strategy, the regioselective C(sp^3^)–C(sp^3^) cross-coupling was also achieved by using alkyl Hantzsch esters (or nitrile) as alkylation reagents. Notably, the α-C(sp^3^)–H of tertiary amines was directly alkylated to form the C(sp^3^)–C(sp^3^) bonds via C(sp^3^)–H–C(sp^3^)–H cross-coupling. All the reactions proceeded at room temperature without the assistance of external oxidants.

## Methods

### General procedure for condition A

In a dry 10-ml glass test tube, substrate *N*-fluoroamides (0.2 mmol), amine (0.6 mmol, 3 equiv), Ir(ppy)_2_(dtbpy)PF_6_ (1 mol%), and K_2_CO_3_ (0.6 mmol, 3 equiv) were dissolved in DMF (2.0 mL) under Ar atmosphere. The glass test tube was then transferred to a 24-W violet-light photoreactor, where it was irradiated for 12 h. The residue was added water (10 mL) and extracted with ethyl acetate (5 mL × 3). The combined organic phase was dried over Na_2_SO_4_. The resulting crude residue was purified via column chromatography on silica gel to afford the desired products.

### General procedure for condition B

In a dry 10-ml glass test tube, substrate *N*-fluoroamides (0.2 mmol), Hantzsch esters or Hantzsch nitrile (0.6 mmol, 3 equiv), Ir(ppy)_2_(dtbpy)PF_6_ (1 mol%), and MeOK (0.5 mmol, 2.5 equiv) were dissolved in DCM (2.0 mL) under Ar atmosphere. The glass test tube was then transferred to a 18-W blue LED photoreactor, where it was irradiated for 12 h. The residue was added water (10 mL) and extracted with DCM (5 mL × 3). The combined organic phase was dried over Na_2_SO_4_. The resulting crude residue was purified via column chromatography on silica gel to afford the desired products.

## Supplementary information

Supplementary Information

## Data Availability

The authors declare that the data supporting the findings of this study are available within the article and its Supplementary Information. Data are also available from the corresponding author on request.
