# Supplementary material for: Visible-light promoted regioselective amination and alkylation of remote C(sp3)-H bonds
Source: Nat Commun. 2020 Mar 19;11:1463. doi: 10.1038/s41467-020-15167-2 (PMC7081228; doi:10.1038/s41467-020-15167-2)
Supplement: Supplementary file 1 — Supplementary Information [file 41467_2020_15167_MOESM1_ESM.pdf]

Supplementary Information

Visible-light Promoted Regioselective Amination and Alkylation of Remote C(sp^3)-H bonds

Guo *et al.*

Supplementary Methods

General Information

Unless stated otherwise, all reactions were carried out under argon atmosphere. All solvents were purified and dried according to standard methods prior to use. ^{1}H NMR, ^{13}C NMR, and ^{19}F NMR spectra were recorded on a Bruker instrument (300 MHz, 75MHz, and 282 MHz) spectrometer in $CDCl_3$ using tetramethylsilane (TMS) as the internal standard unless otherwise noted. Data for ^{1}H NMR are recorded as follows: chemical shift (δ, ppm), multiplicity (s = singlet, d = doublet, t = triplet, m = multiplet, br = broad, q = quartet, coupling constant(s) in Hz integration). Data for ^{13}C NMR and ^{19}F NMR are reported in terms of chemical shift (δ, ppm). HRMS were obtained by the ESI ionization sources. GC-MS were obtained by the EI sources.

Materials and Methods: Commercial grade reagents and solvents were purchased from Energy-Chemical and Inno-Chemical, and used as received without further purifications.

Optimization of Reaction Condition A.

Supplementary Table 1. Photo Catalyst Screening.[a,b]

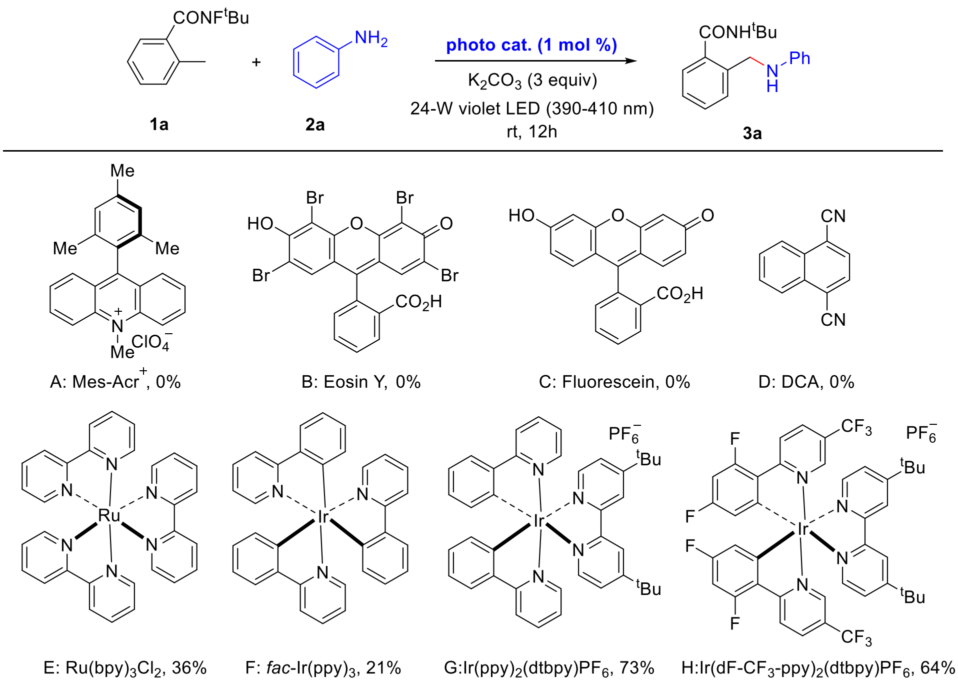

[a] Unless otherwise noted, the reactions were carried out by using **1a** (0.1 mmol), **2a** (3.0 equiv), photo cat. (1 mol %), DMF (1 ml), K_2CO_3 (3 equiv) under Ar and stirred at room temperature for 12 h under 24-W violet LED (390-410 nm) light irradiation. [b] Isolated yields.

Supplementary Table 2. Light Sources Screening.[a,b]

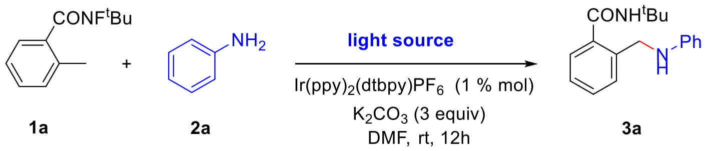

Entry	Light source	Yield(%)[b]	Entry	Light source	Yield(%)[b]
1	violet LED (24-W, 390-410 nm)	73	4	18-W green LED	trace
2	18-W blue LED	53	5	25-W 365 nm UV light	19
3	18-W white LED	21	6	25-W 254 nm UV light	16

[a] **1a** (0.1 mmol), **2a** (3.0 equiv), photo cat. (1 mol %), DMF (1 ml), base (3 equiv), under Ar and stirred at room temperature for 12 h under specific light irradiation. [b] Isolated yields.

Supplementary Table 3. Bases Screening.[a,b]

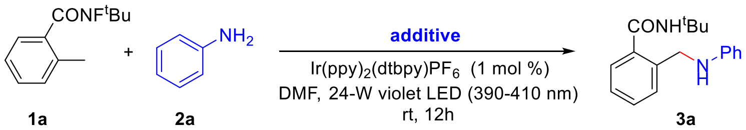

Entry	base	yield(%)[b]	Entry	base	yield(%)[b]
1	K_2CO_3	73	10	CsF	51
2	Na_2CO_3	22	11	$NaHCO_3$	25
3	Cs_2CO_3	35	12	$KHCO_3$	27
4	K_3PO_4	10	13	LiOH	23
5	*t*-BuONa	42	14	NaOH	5
6	*t*-BuOK	15	15	DABCO	26
7	*t*-BuOLi	36	16	TBACl	24
8	KOMe	26	17	TBAI	trace
9	NaOMe	37			

[a] **1a** (0.1 mmol), **2a** (3.0 equiv), photo cat. (1 mol %), DMF (1 ml), base (3 equiv), under Ar and stirred at room temperature for 12 h under 24-W violet LED (390-410 nm) light irradiation. [b] Isolated yields.

Supplementary Table 4. Solvents Screening.[a,b]

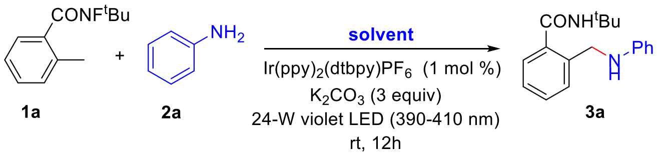

Entry	solvent	yield (%)[b]	Entry	solvent	yield (%)[b]
1	DMF	73	5	toluene	37
2	THF	44	6	DMAc	61
3	DMSO	66	7	1,4-dioxane	24
4	MeCN	22	8	H_2O	trace

[a] **1a** (0.1 mmol), **2a** (3.0 equiv), photo cat. (1 mol %), solvent (1 ml), K_2CO_3 (3.0 equiv), under Ar and stirred at room temperature for 12 h under 24-W violet LED (390-410 nm) light irradiation. [b] Isolated yields.

Optimization of Reaction Condition B.

Supplementary Table 5. Photo Catalyst Screening.[a,b]

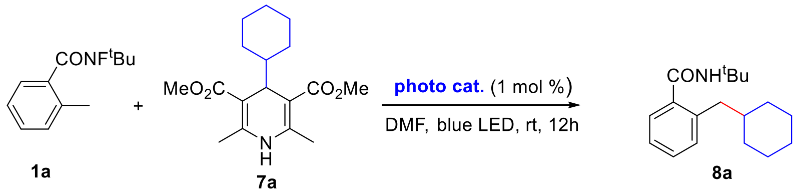

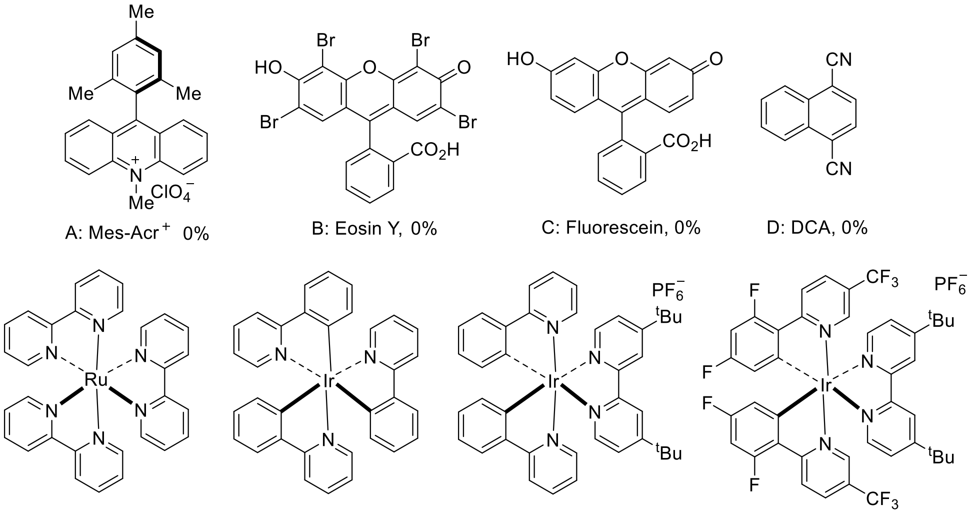

A: Mes-Acr$^+$ 0% B: Eosin Y, 0% C: Fluorescein, 0% D: DCA, 0%

E: $Ru(bpy)_3Cl_2$, 0% F: *fac*-$Ir(ppy)_3$, 20% G:$Ir(ppy)_2(dtbpy)PF_6$, 25% H:$Ir(dF\text{-}CF_3\text{-}ppy)_2(dtbpy)PF_6$, 16%

[a] Unless otherwise noted, the reactions were carried out by using **1a** (0.1 mmol), **7a** (2.0 equiv), photo cat. (1 mol %), DMF (1 ml), under Ar and stirred at room temperature for 12 h under 18-W blue LED light irradiation. [b] Isolated yields.

Supplementary Table 6. Light Sources Screening.[a,b]

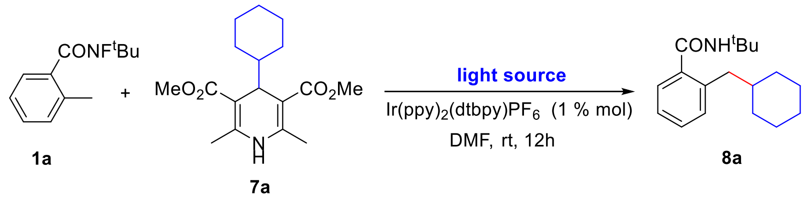

Entry	Light source	Yield(%)[b]	Entry	Light source	Yield(%)[b]
1	18-W blue LED	25	4	18-W green LED	0
2	violet LED (24-W, 390-410 nm)	23	5	25-W 365 nm UV light	0
3	18-W white LED	12	6	25-W 254 nm UV light	0

[a] **1a** (0.1 mmol), **7a** (2.0 equiv), $Ir(ppy)_2(dtbpy)PF_6$ (1 % mol), DMF (1 ml), under Ar and stirred at room temperature with specific light irradiation. [b] Isolated yields.

Supplementary Table 7. Additive Screening.[a,b]

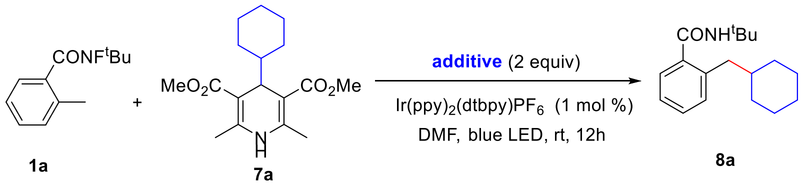

entry	additive	yield(%)[b]	entry	additive	yield(%)[b]
1	Cs_2CO_3	36	10	CsF	26
2	Na_2CO_3	31	11	$NaHCO_3$	25
3	K_2CO_3	35	12	$KHCO_3$	24
4	K_3PO_4	28	13	LiOH	27
5	*t*-BuONa	27	14	NaOH	24
6	*t*-BuOK	35	15	DABCO	21
7	*t*-BuOLi	33	16	TBACl	24
8	KOMe	46	17	TBAI	25
9	NaOMe	40			

[a] **1a** (0.1 mmol), **7a** (2.0 equiv), photo cat. (1 mol %), DMF (1 ml), additive (2.0 equiv), under Ar and stirred at room temperature for 12 h under 18-W blue LED light irradiation. [b] Isolated yields.

Supplementary Table 8. Solvents Screening.[a,b]

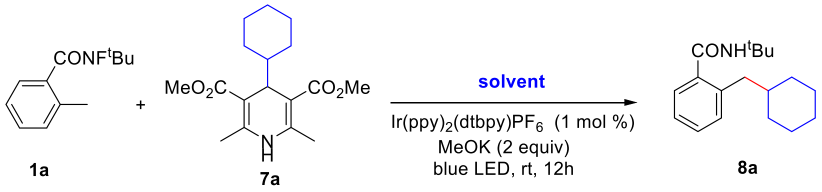

entry	solvent	yield(%)[b]	entry	solvent	yield(%)[b]
1	DMF	46	5	toluene	35
2	THF	39	6	DMAc	43
3	DCM	65	7	1,4-dioxane	24
4	MeCN	28	8	H_2O	13

[a] **1a** (0.1 mmol),**7a** (2.0 equiv), photo cat. (1 mol %), solvent (1 ml), MeOK (2.0 equiv), under Ar and stirred at room temperature for 12 h under 18-W blue LED light irradiation. [b] Isolated yields.

Supplementary Table 9. Control Experiments.[a,b]

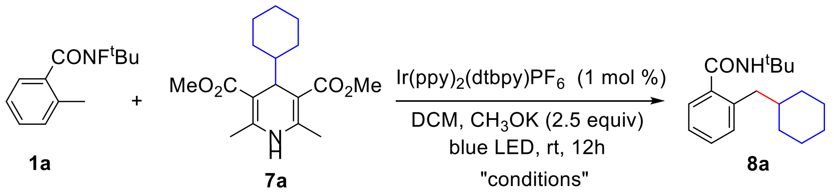

entry	change from the "conditions"	yield(%)[b]
1	no change	65
2	CH_3OK (3.0 equiv)	67
3	CH_3OK (2.5 equiv)	68
4	CH_3OK (1.0 equiv)	40
5	CH_3OK (2.5 equiv),**7a** (3.0 equiv)	71
6	CH_3OK (2.5 equiv), **7a** (2.5 equiv)	68
7	CH_3OK (2.5 equiv), **7a** (1.0 equiv)	32
9	without Ir cat.	0
10	In the dark, 24 h at rt	0
11	In the dark, 12 h at 80 ^oC	0

[a] 0.1 mmol scale. [b] Isolated yields.

List of substrates.

Supplementary Table 10. List of *N*-fluoroamides[1]

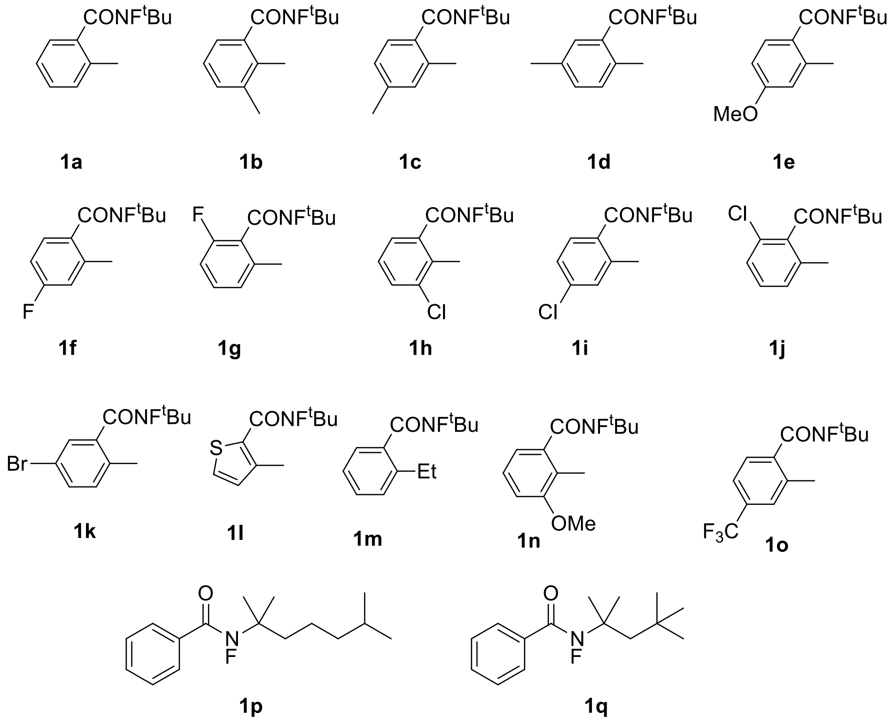

Supplementary Table 11. List of amines.

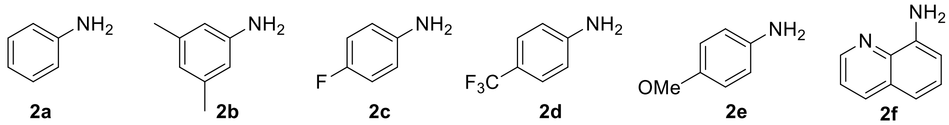

2a 2b 2c 2d 2e 2f

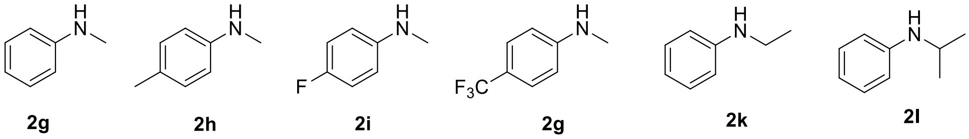

2g 2h 2i 2g 2k 2l

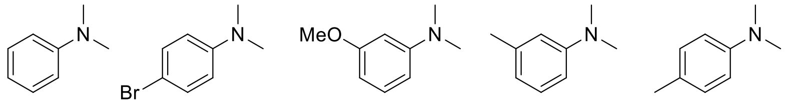

2m 2n 2o 2p 2q

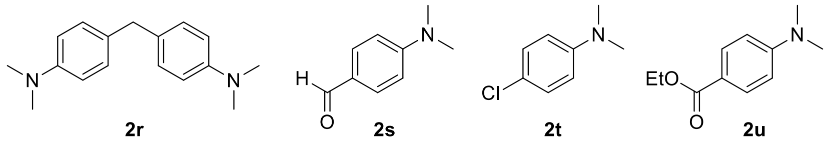

2r 2s 2t 2u

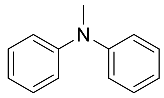

2v

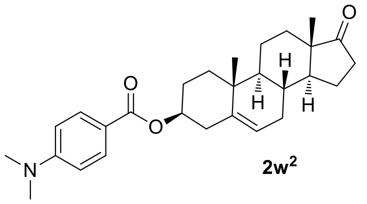

2w[2]

Supplementary Table 12. List of Hantzsch esters or Hantzsch nitrile.[3]

2° alkyl Hantzsch ester:

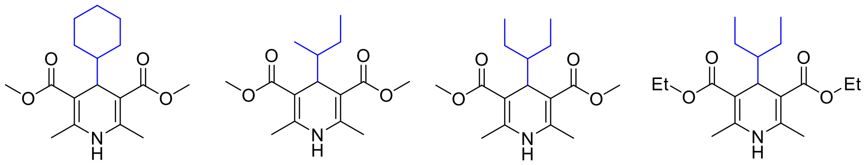

7a 7b 7c 7ca

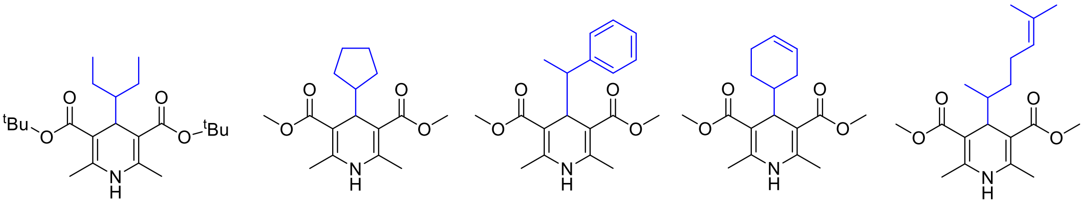

7cb 7d 7e 7f 7g

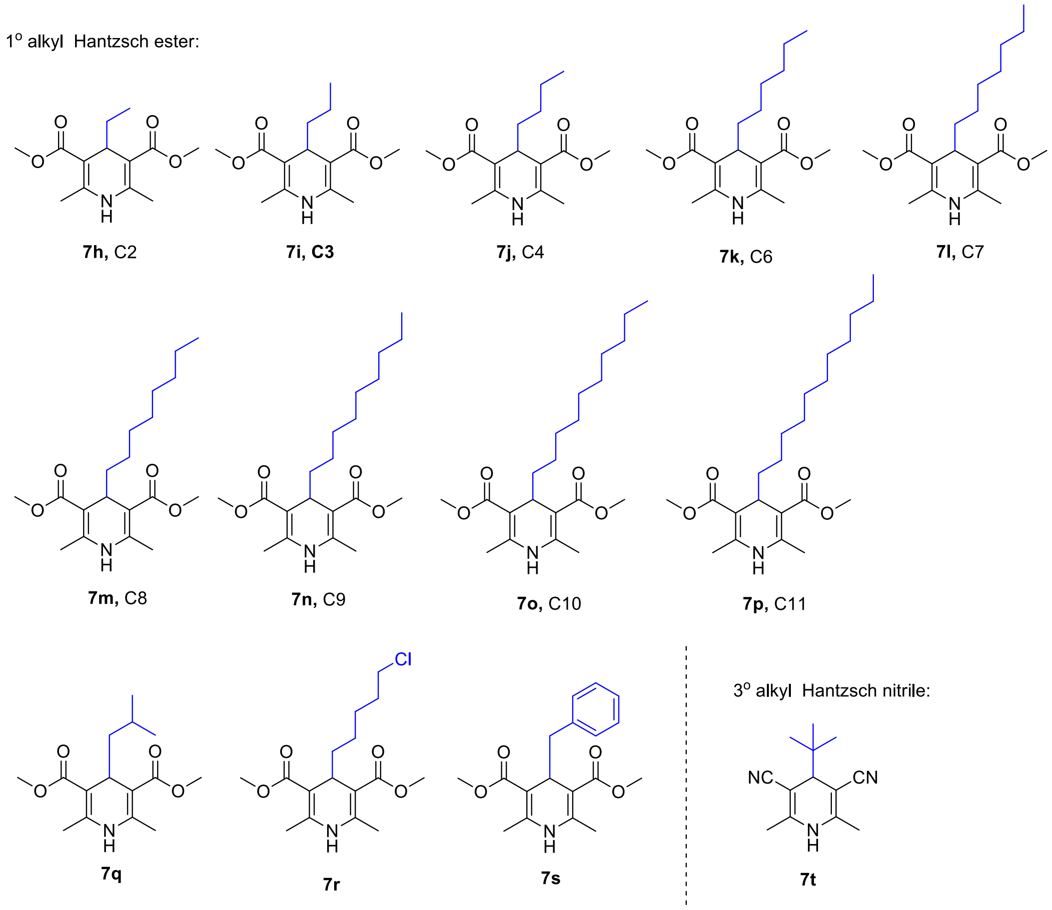

General procedure.

General procedure of "condition A"

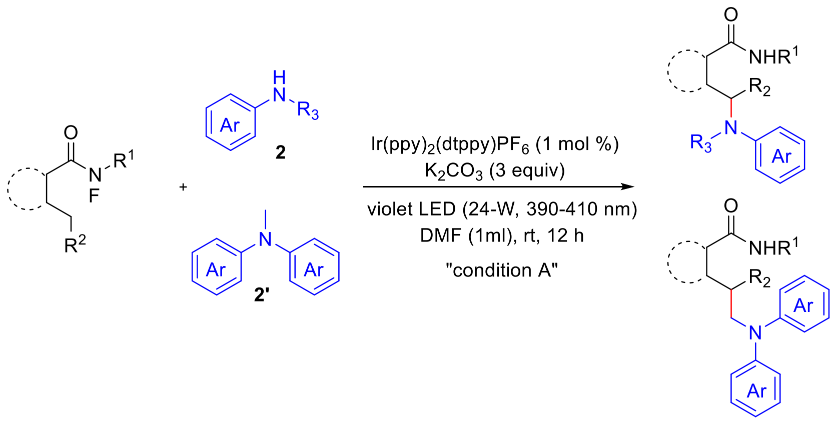

In a dry 10 ml glass test tube, substrate *N*-fluoroamides (0.2 mmol), aniline (0.6 mmol, 3 equiv), Ir(ppy)$_2$(dtbpy)PF$_6$ (1 mol %), and K_2CO_3 (0.6 mmol, 3 equiv) were dissolved in DMF (2.0 mL) under Ar atmosphere. The glass test tube was then transferred to a 24-W violet light photoreactor, where it was irradiated for 12 h. Then, the reaction mixture was quenched by H_2O, extracted by EtOAc, wash by saturation NaCl, dried over anhydrous sodium sulfate, concentrated in vacuo, and the residue was purified by column chromatography to afford the product.

General procedure of "condition B"

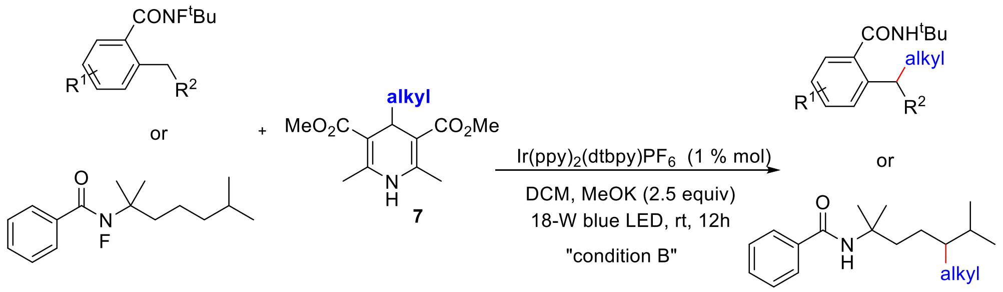

In a dry 10 ml glass test tube, substrate *N*-fluoroamides (0.2 mmol), Hantzsch esters or Hantzsch nitrile (0.6 mmol, 3 equiv), Ir(ppy)$_2$(dtbpy)PF$_6$ (1 mol %), and MeOK (0.5 mmol, 2.5 equiv) were dissolved in DCM (2.0 mL) under Ar atmosphere. The glass test tube was then transferred to an 18-W blue LED photoreactor, where it was irradiated for 12 h. Then, the reaction mixture was quenched by H_2O, extracted by DCM, wash by saturation NaCl, dried over anhydrous sodium sulfate, concentrated in vacuo, and the residue was purified by column chromatography to afford the product.

Synthetic application:[4-5]

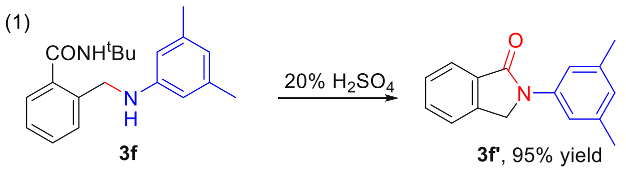

***N*-(tert-butyl)-2-(((3,5-dimethylphenyl)amino)methyl)benzamide** (**3f**, 31.0 mg, 0.1 mmol) was dissolved in sulfuric acid (20 wt% in H_2O). The reaction mixture was stirred for 12 h at 100 ºC. Then, it was cooled down to the room temperature and extracted with ethyl ether. The organic layer was dried over anhydrous sodium sulfate, concentrated in vacuo, and the residue was purified by chromatography on silica gel (*n*-hexane/EtOAc) to give the **2-(3,5-dimethylphenyl)isoindolin-1-one** (**3f'**, 95%).

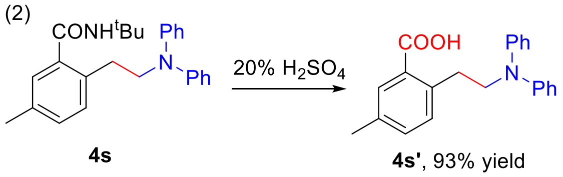

***N*-(tert-butyl)-2-(2-(diphenylamino)ethyl)-5-methylbenzamide** (**4s**, 37.2 mg, 0.1 mmol) was dissolved in sulfuric acid (20 wt% in H_2O). The reaction mixture was stirred for 12 h at 100 ºC. Then, it was cooled down to the room temperature and extracted with ethyl ether. The organic layer was dried over anhydrous sodium sulfate, concentrated in vacuo, and the residue was purified by chromatography on silica gel (*n*-hexane/EtOAc) to give the **2-(2-(diphenylamino)ethyl)-5-methylbenzoic acid** (**4S'**, 93%).

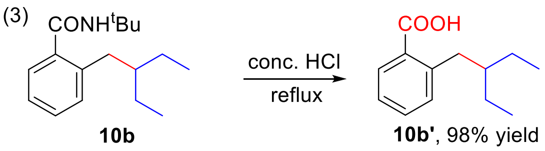

***N*-(tert-butyl)-2-(2-ethylbutyl)benzamide** (**10b**, 26.1 mg, 0.1 mmol) was dissolved in conc. HCl (1 mL). The mixture was then reflux for 48 hours. Then, it was cooled down to the room temperature and extracted with ethyl ether. The organic layer was dried over anhydrous sodium sulfate, concentrated in vacuo, and the residue was purified by chromatography on silica gel (*n*-hexane/EtOAc) to give the **2-(2-ethylbutyl)benzoic acid** (**10b'**, 20.2 mg, 98%).

Supplementary Discussion

The Mechanistic Study.

Trapping with TEMPO:

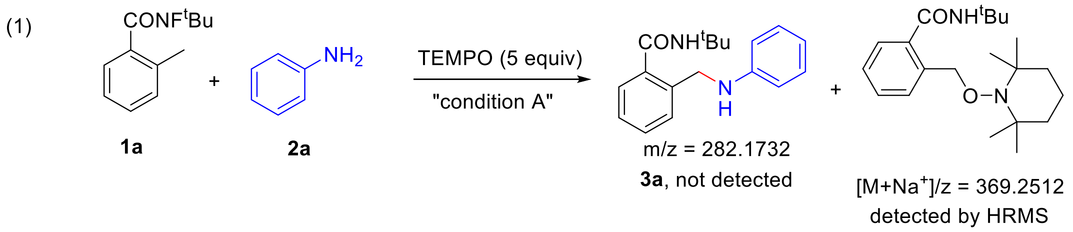

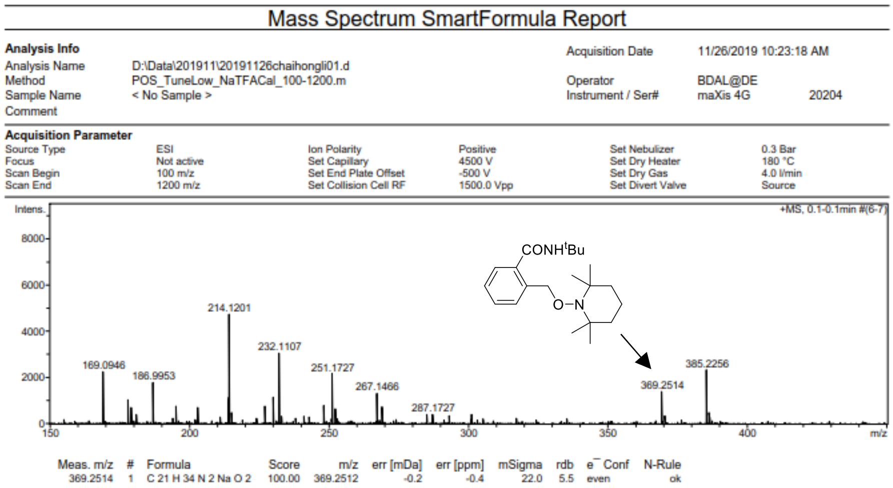

Supplementary Figure 1. Mass Spectrum of TEMPO addition.

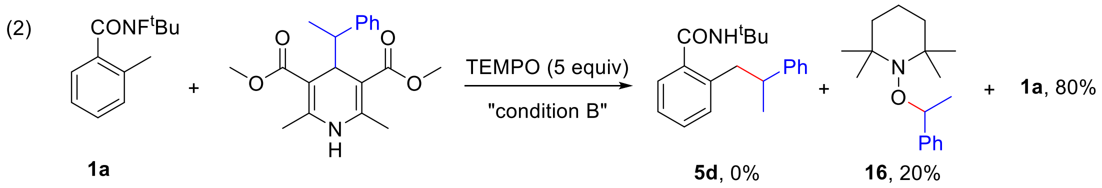

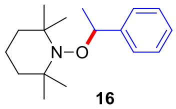

16

2,2,6,6-tetramethyl-1-(1-phenylethoxy)piperidine (16)

^{1}HNMR (300 MHz, $CDCl_3$) δ 7.21-7.42 (m, 5H), 4.77 (dd, J = 6.6 Hz, 13.3 Hz, 1H), 1.48 (d, J = 6 Hz, 6H), 1.37 (s, 3H), 1.29 (s, 3H), 1.16 (s, 3H), 1.02 (s, 3H), 0.65 (s, 3H).

^{13}CNMR (75 MHz, $CDCl_3$) δ 145.86, 128.00, 126.77, 126.61, 83.14, 59.67, 40.38, 23.58, 20.35, 17.25.

HRMS (ESI): $C_{17}H_{27}NO+Na^+$ Calcd: 284.1985, Found: 284.1986.

Radical clock experiments:

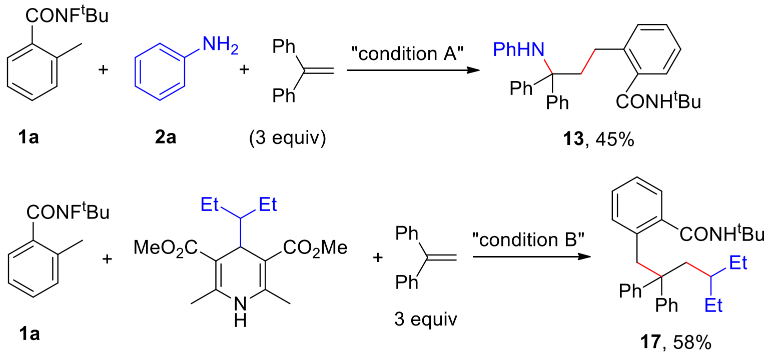

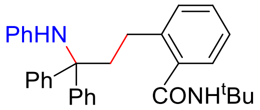

***N*-(tert-butyl)-2-(3,3-diphenyl-3-(phenylamino)propyl)benzamide (13)**, 40.4 mg, yield: 45%. Yellow oil.

^{1}HNMR (300 MHz, CDC13): δ 7.59 (d, J = 7.8 Hz, 4H), 7.10-7.36 (m, 9H), 6.95 (t, J = 7.5 Hz, 2H), 6.81 (d, J = 7.2 Hz, 1H), 6.55 (t, J = 7.2 Hz, 1H), 6.46 (d, J = 7.8 Hz, 2H), 5.69 (s, 1H), 5.62 (s, 1H), 2.72-2.78 (m, 2H), 2.48-2.55 (m, 2H), 1.49 (s, 9H).

^{13}CNMR (75 MHz, CDC13): δ 170.13, 146.66, 144.37, 140.68, 137.01, 130.36, 129.82, 128.35, 128.10, 127.85, 126.47, 125.89, 116.64, 115.64, 65.93, 51.91, 44.74, 28.96, 28.81 (d, J = 3.8 Hz)

HRMS (ESI) $C_{32}H_{34}N_2O+Na^+$ Calcd: 485.2563, Found: 485.2573.

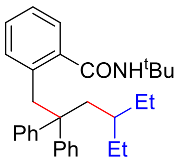

***N*-(tert-butyl)-2-(4-ethyl-2,2-diphenylhexyl)benzamide (17)**, 52.1 mg, yield: 58%. Yellow oil.

^{1}HNMR (300 MHz, $CDCl_3$) δ 7.15-7.25 (m, 11H), 7.06 (t, J = 7.5 Hz, 1H), 6.93 (t, J = 7.8 Hz, 1H), 6.37 (d, J = 7.8 Hz, 1H), 5.31 (s, 1H), 3.83 (s, 2H), 1.89 (d, J = 4.5 Hz, 2H), 1.45 (s, 9H), 1.20-1.30 (m, 1H), 0.83-0.93 (m, 4H), 0.56 (t, J = 7.2 Hz, 6H).

^{13}CNMR (75 MHz, $CDCl_3$) δ 17.24, 147.22, 139.72, 136.69, 131.18, 129.28, 128.34, 127.59, 126.49, 125.80, 125.65, 52.63, 51.63, 43.23, 42.23, 35.33, 28.72, 25.37, 10.23.

HRMS (ESI): $C_{31}H_{39}NO+Na^+$ Calcd: 464.2924, Found: 464.2940.

Homo coupling results:

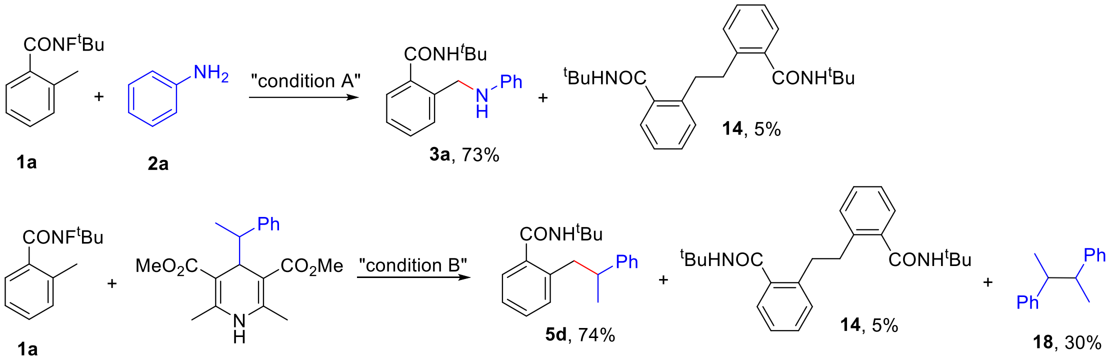

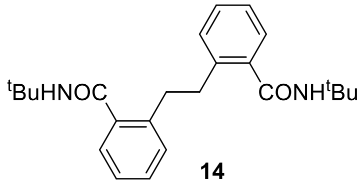

14

2,2'-(ethane-1,2-diyl)bis(N-(tert-butyl)benzamide) (14), 3.8 mg, yield: 5%. Colorless oil.

^{1}HNMR (300 MHz, $CDCl_3$) δ 7.25-7.31 (m, 5H), 7.18 (t, J = 7.5 Hz, 3H), 5.64 (s, 2H), 3.09 (s, 4H), 1.45 (s, 18H).

^{13}CNMR (75 MHz, $CDCl_3$) δ 169.85, 139.46, 137.84, 130.54, 129.44, 126.60, 125.92, 51.75, 35.16, 28.82.

HRMS (ESI) $C_{24}H_{32}N_2O_2$+Na^+ Calcd: 403.2361, Found: 403.2360.

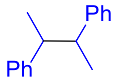

18

butane-2,3-diyldibenzene (18), dr = 1:1.3. Colorless oil.

^{1}HNMR (300 MHz, $CDCl_3$) δ 6.93-7.34 (m, 10H), 2.93-2.95 (m, 1H), 2.79-2.80 (m, 1H), 1.28 (d, J = 6.6 Hz, 3H), 1.02 (d, J = 6 Hz, 3H).

^{13}CNMR (75 MHz, $CDCl_3$) δ 146.51, 145.86, 128.31, 127.85, 127.80, 127.04, 126.08, 125.72, 47.29, 46.50, 29.75, 21.08, 17.97.

HRMS (ESI) $C_{16}H_{18}$+Na^+ Calcd: 233.1301, Found: 233.1302.

Isotope tracing:

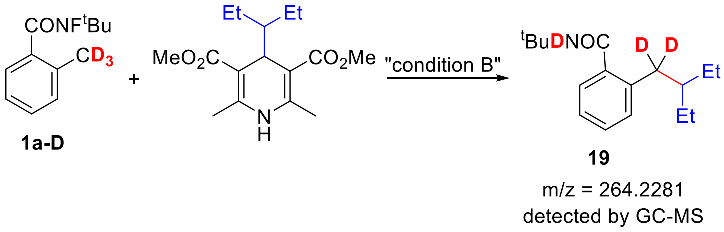

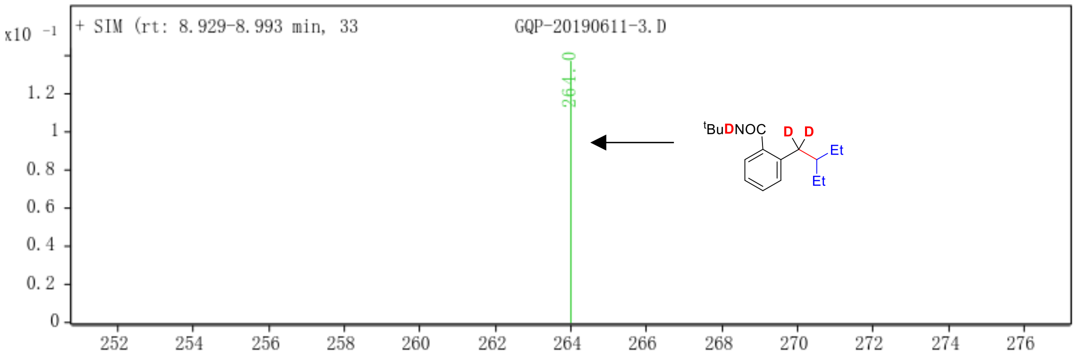

Supplementary Figure 2. GC-MS of isotope tracing.

Reactions under oxidative conditions:

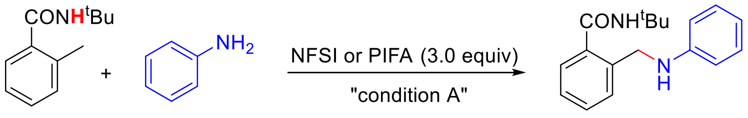

3a, not detected

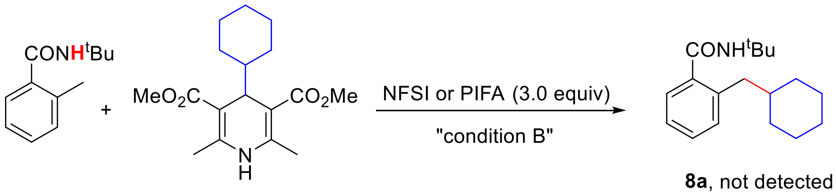

8a, not detected

The reactions with *N*-chloro-amide substrate.

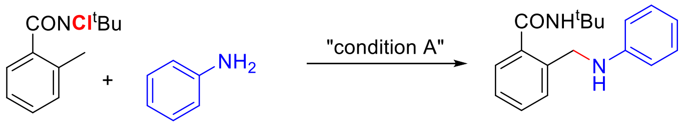

3a, 53%

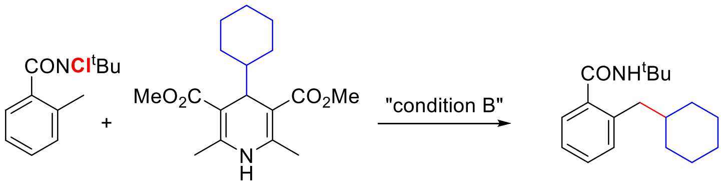

8a, 68%

Other radical precursors:

Other radical precursors, such as $PhSO_2Na$ and $BnBF_3K$ were used instead of *N*,*N*-dimethylaniline.

(a) When $PhSO_2Na$ was used as radical precursor, the desired product was obtained in 45% yield.

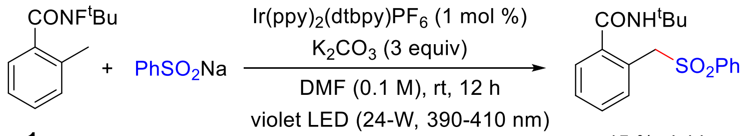

1a 45 % yield

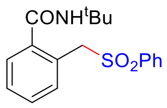

***N*-(tert-butyl)-2-((phenylsulfonyl)methyl)benzamide,** white solid, mp: 143-144℃.

^{1}HNMR (300 MHz, $CDCl_3$): δ 7.81 (d, *J* = 7.2 Hz, 2H), 7.68 (t, *J* = 7.5 Hz, 1H), 7.55 (t, *J* = 7.8 Hz, 2H), 7.46-7.49 (m, 1H), 7.27-7.41 (m, 2H), 7.11 (d, *J* = 7.2 Hz, 1H), 6.37 (br, 1H), 4.68 (s, 2H), 1.50 (s, 9H).

^{13}CNMR (75 MHz, $CDCl_3$): δ 168.62, 139.88, 138.78, 133.96, 132.53, 129.57, 129.29, 129.07, 128.38, 128.06, 124.63, 58.98, 52.08, 28.70.

HRMS (ESI): $C_{18}H_{21}NO_3S+Na^+$ Calcd: 354.1134, Found: 354.1134.

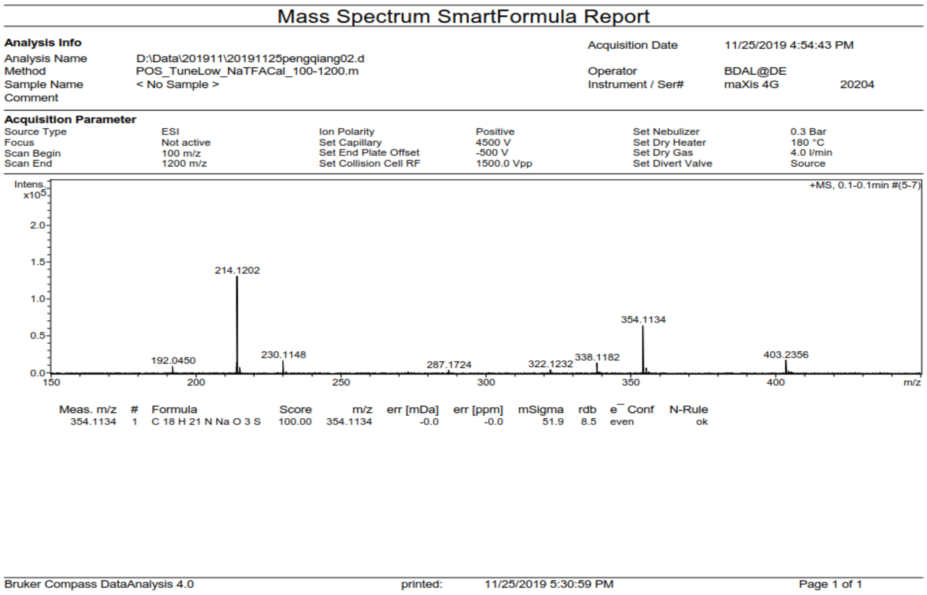

Supplementary Figure 3. Mass Spectrum of the **$PhSO_2Na$** reaction.

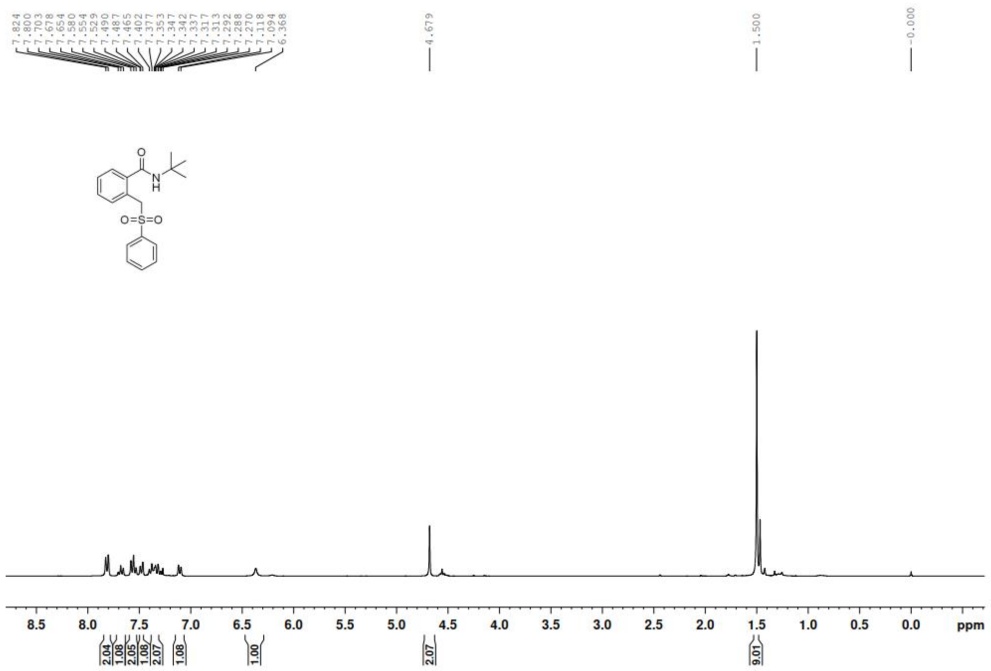

Supplementary Figure 4. ^{1}H NMR (300 MHz, $CDCl_3$) spectra for compound ***N*-(tert-butyl)-2-((phenylsulfonyl)methyl)benzamide.**

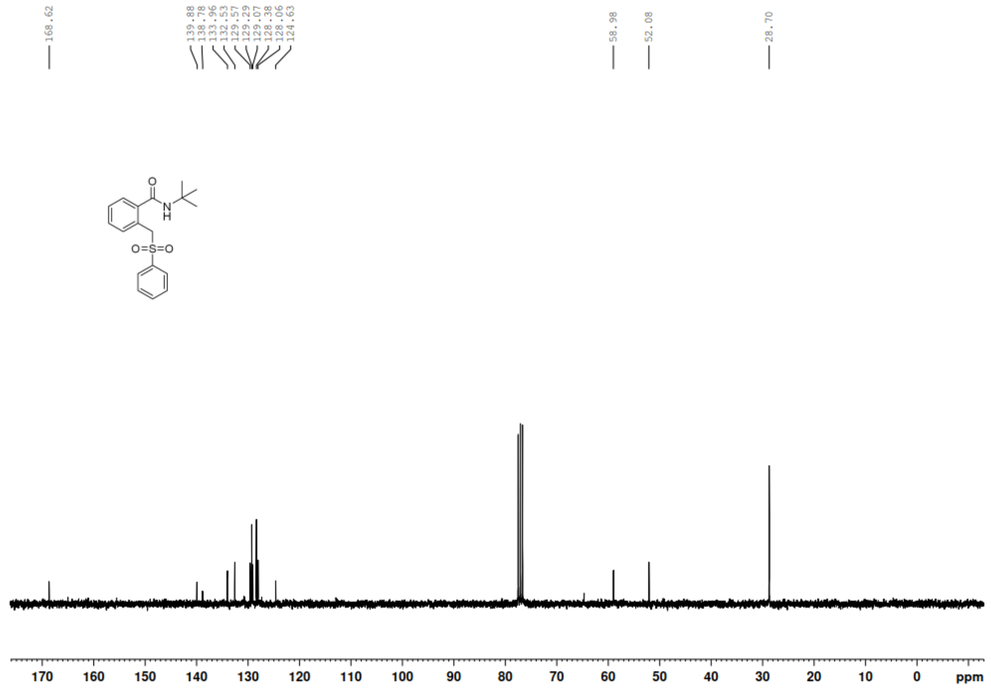

Supplementary Figure 5. ^{13}C NMR (75 MHz, $CDCl_3$) spectra for compound ***N*-(tert-butyl)-2-((phenylsulfonyl)methyl)benzamide.**

(b) When $BnBF_3K$ was used as radical precursor, the desired product was obtained in 38% yield.

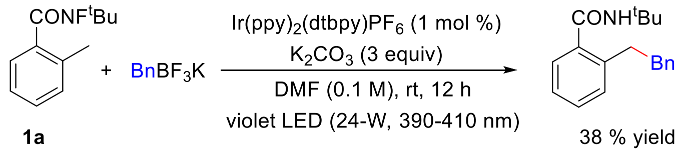

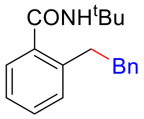

***N*-(tert-butyl)-2-phenethylbenzamide**. Pale yellow solid, mp: 97-100℃.

^{1}HNMR (300 MHz, $CDCl_3$) δ 7.16-7.32 (m, 9H), 5.48(s, 1H), 3.07 (t, J = 7.2 Hz, 2H), 2.92 (t, J = 8.7 Hz, 2H), 1.45 (s, 9H).

^{13}CNMR (75 MHz, $CDCl_3$) δ 169.72, 141.81, 139.44, 137.95, 130.21, 129.45, 128.55, 128.31, 126.58, 125.96, 125.88, 51.70, 38.08, 35.47, 28.78.

HRMS (ESI): $C_{19}H_{23}NO+Na^+$ Calcd: 304.1672, Found: 304.1672.

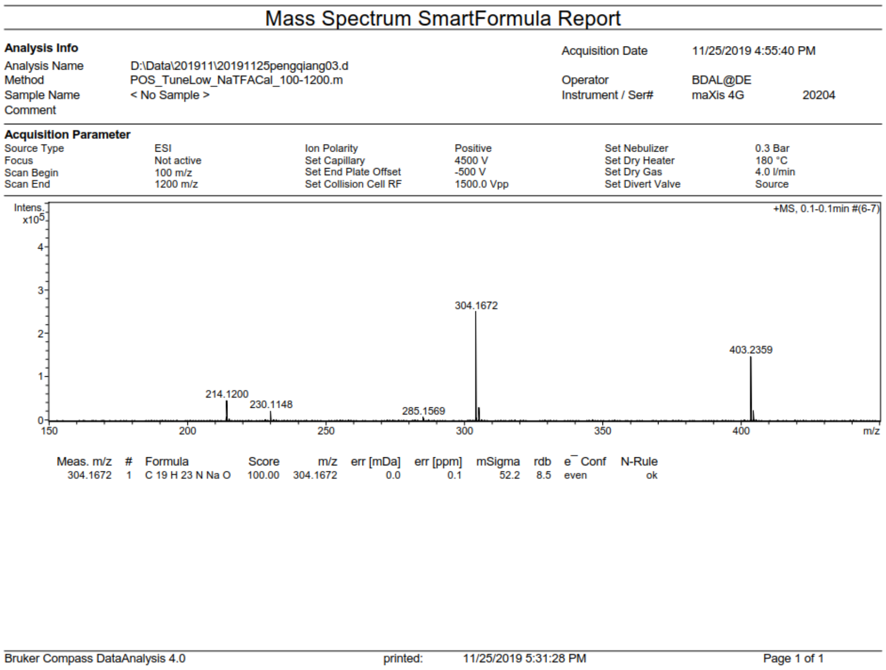

Supplementary Figure 6. Mass Spectrum of the **$BnBF_3K$** reaction.

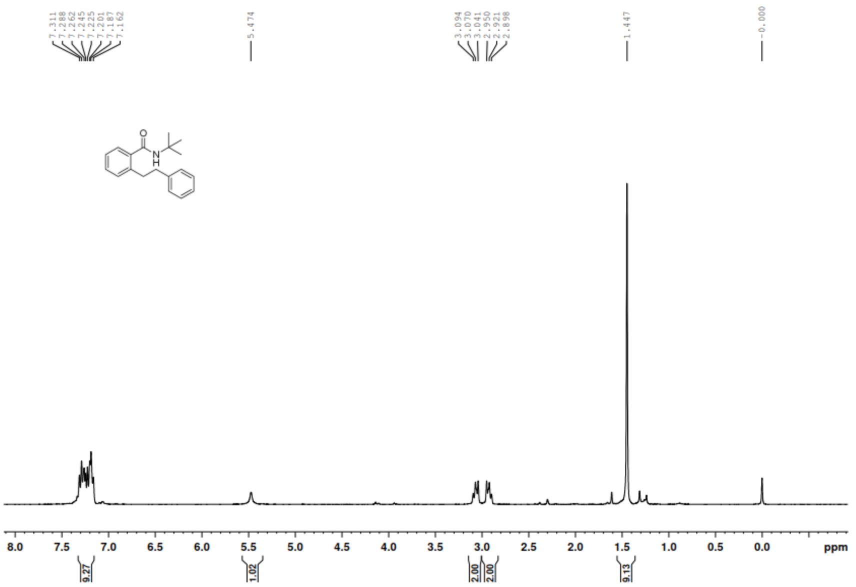

Supplementary Figure 7. ^{1}H NMR (300 MHz, $CDCl_3$) spectra for compound ***N*-(tert-butyl)-2-phenethylbenzamide**.

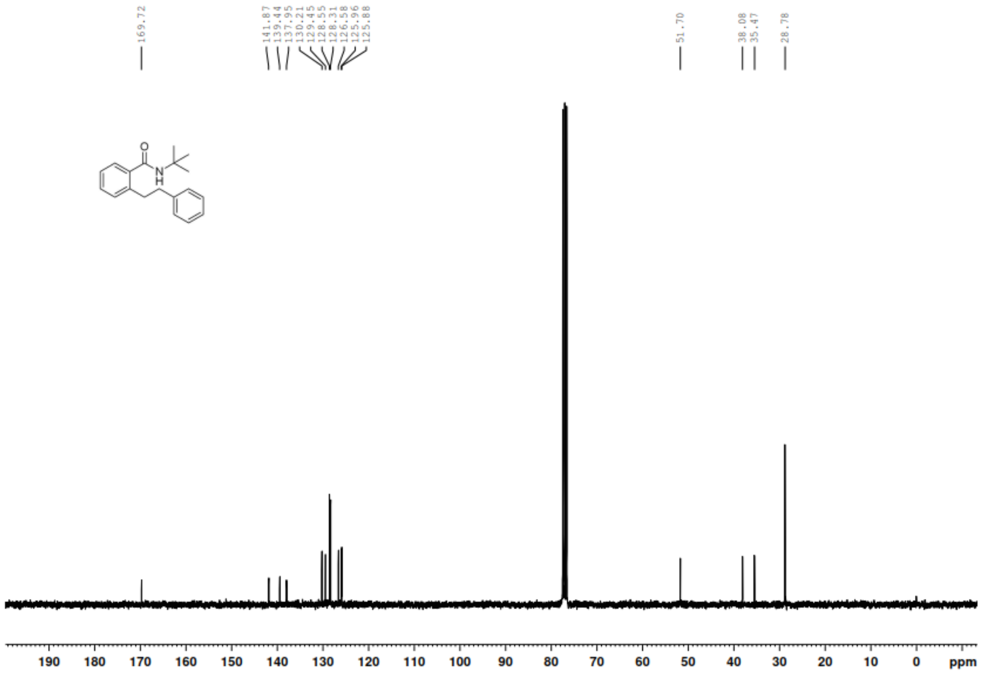

Supplementary Figure 8. ^{13}C NMR (75 MHz, $CDCl_3$) spectra for compound ***N*-(tert-butyl)-2-phenethylbenzamide**.

Cyclic Voltammetry Experiments:

Cyclic Voltammetry was performed on a CH Instruments Electrochemical Workstation model CHI760E. A solution of the sample in MeCN (0.001 M) was tested with 0.1 M Bu_4NPF_6 as the supporting electrolyte, using a glassy carbon as the working electrode, a Pt as the counter electrode, and a saturated calomel electrode reference electrode. Scan rate = 0.05 V/s, 2 sweep segments, a sample interval of 0.001 V.

Reductive potential of substrate *N*-fluorocarboxamides 1a and 1o.

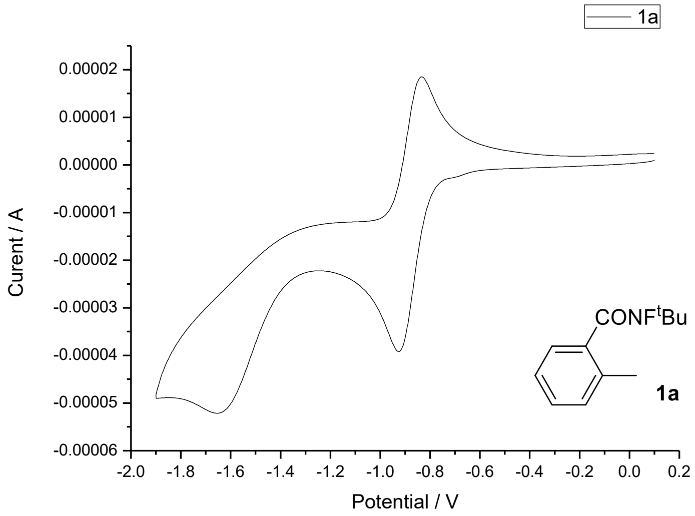

Supplementary Figure 9. Cyclic Voltammogram of **1a**, $E_p^{0/-1}$(**1a**) = - 0.84 V (vs SCE).

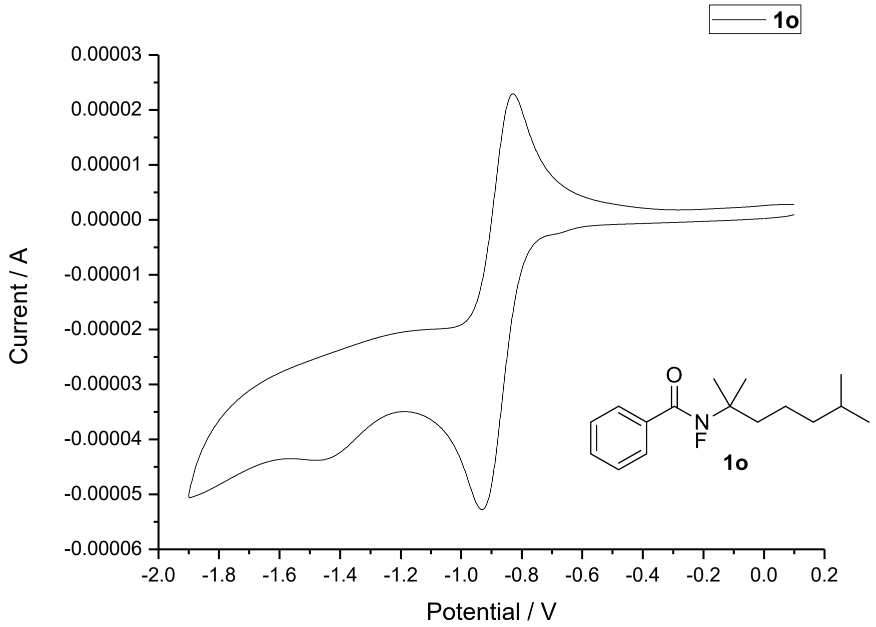

Supplementary Figure 10. Cyclic Voltammogram of **1o**, $E_p^{0/-1}$(**1o**) = - 0.83 V (vs SCE).

Reductive potential of substrate Hantzsch ester PhNH2 (2a).

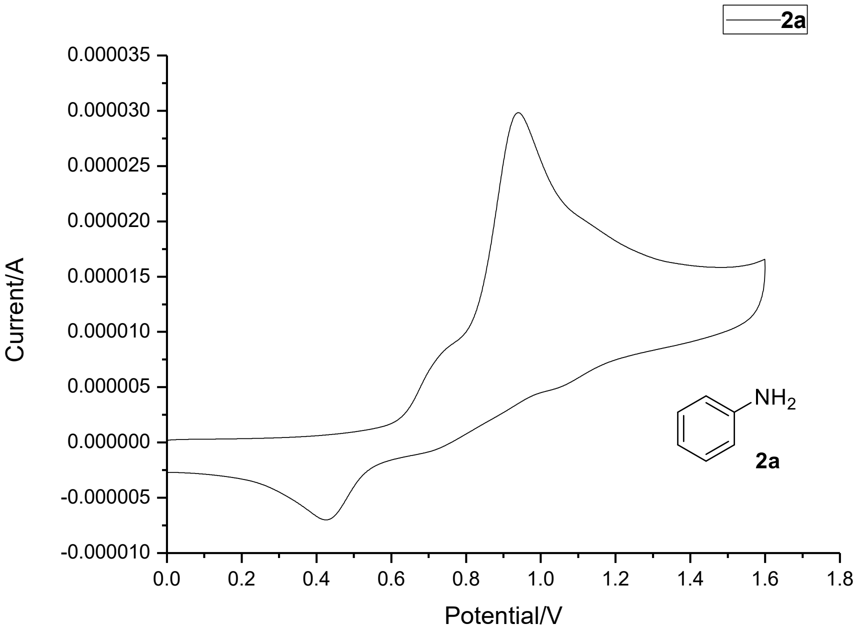

Supplementary Figure 11. Cyclic Voltammogram of **PhNH2 (2a)**, $E_p^{0/+1}$(**2a**) = 0.94 V (vs SCE).

Reductive potential of substrate Hantzsch ester 7c, 7ca, 7cb.

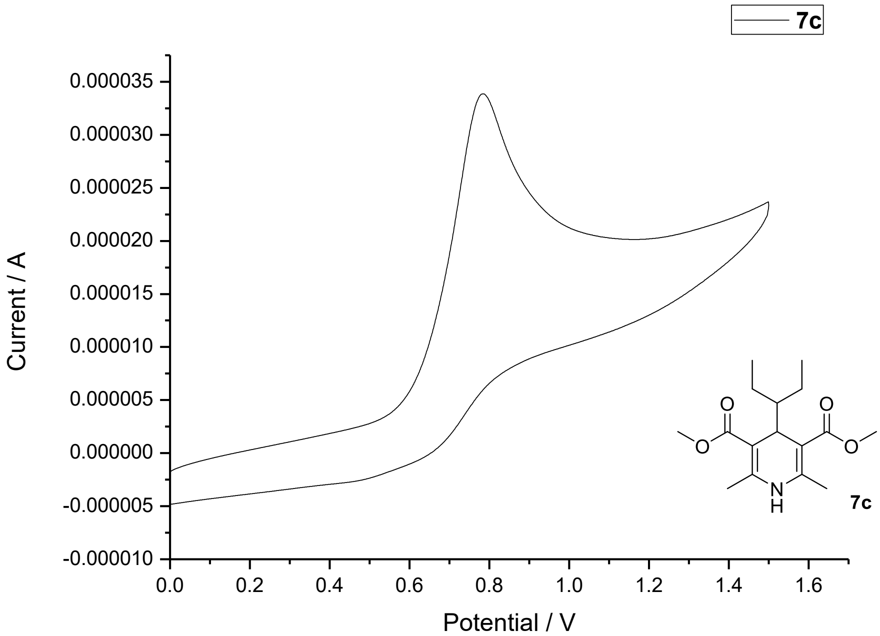

Supplementary Figure 12. Cyclic Voltammogram of **7c**, $E_p^{0/+1}$(**7c**) = 0.78 V (vs SCE)

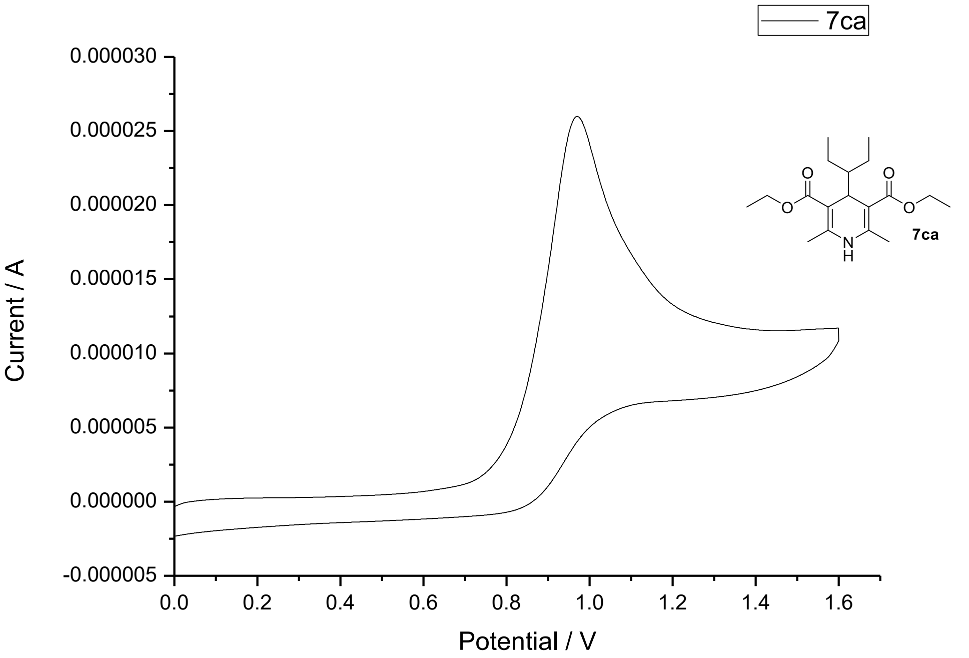

Supplementary Figure 13. Cyclic Voltammogram of **7ca**, $E_p^{0/+1}$(**7ca**) = 0.97 V (vs SCE).

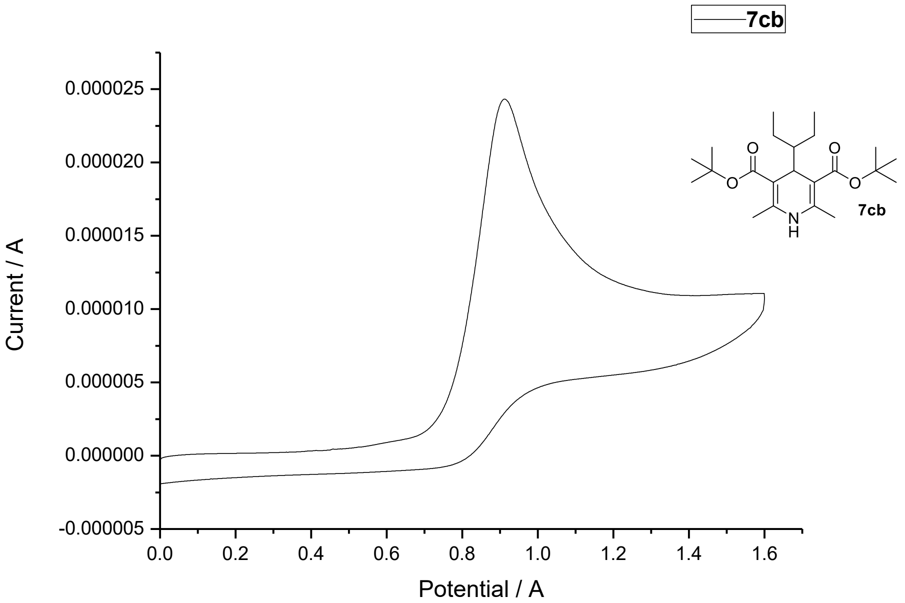

Supplementary Figure 14. Cyclic Voltammogram of **7cb**, $E_p^{0/+1}$(**7cb**) = 0.91 V (vs SCE).

Stern-Volmer fluorescence quenching study.

DMF was degassed with a stream of argon for 30 min. Ir(ppy)$_2$(dtbpy)PF$_6$ (4.6 mg, 5.0 μmol) was dissolved in 1.0 mL DMF to prepare a 5×10^{-3} M solution. 100 μL of this solution was added to each of a set of 6 volumetric flasks (10 mL). Subsequently, the solution of quencher **1a** or **2a** in DMF (1.0 mL, 0.025 M) was added in increasing amounts (0, 100 μL, 200 μL, 300 μL, 400 μL, 500 μL) to the volumetric

flasks and the volume of volumetric flasks were adjusted to 10 mL by adding DMF. Emission intensities were recorded by using a HITACHI spectrofluorophotometer F-7000. All solutions were excited at 375 nm and the fluorescence emission spectra were recorded.

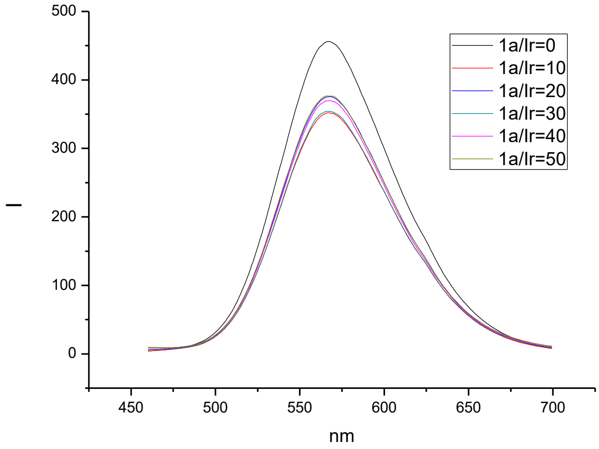

Supplementary Figure 15. The emission quenching spectrum of $Ir(ppy)_2(dtbpy)PF_6$ by various concentrations of quencher **1a.**

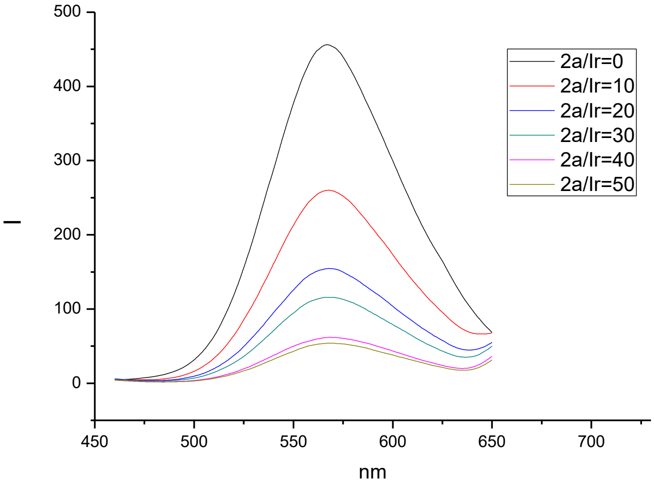

Supplementary Figure 16. The emission quenching spectrum of $Ir(ppy)_2(dtbpy)PF_6$ by various concentrations of quencher **2a.**

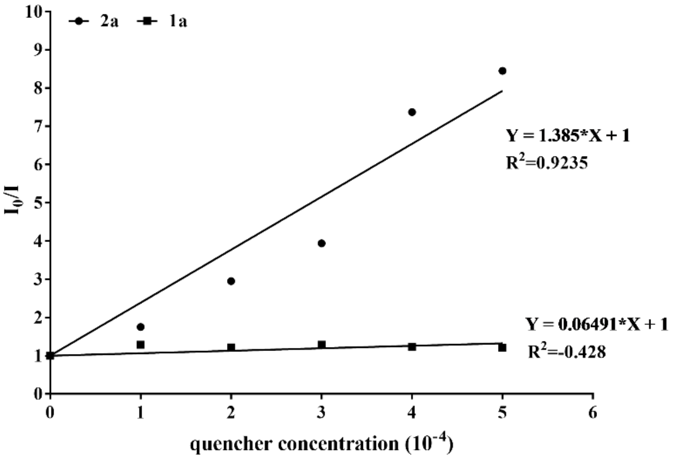

Supplementary Figure 17. Stern-Volmer plot for the emission quenching of Ir(ppy)$_2$(dtbpy)PF$_6$ by various concentrations of quencher **1a** or **2a**.

EPR experients

EPR spectra were recorded at room temperature without using any free radical spin trapping agent. According to general procedure,[4] the HFIP solutions of PC (Ir(ppy)$_2$(dtbpy)PF$_6$), aniline (**2a**), or PC (Ir(ppy)$_2$(dtbpy)PF$_6$) with aniline **2a** were respectively stirred under violet LED (24-W, 390-410 nm) irradiation for 1h, and then measured by EPR. As shown in Figure S10, EPR signals were observed only in Fig. c, where PC (Ir(ppy)$_2$(dtbpy)PF$_6$) and aniline (**2a**) were added together in the HFIP solution. The result indicated that radical species were generated therein.

(a)

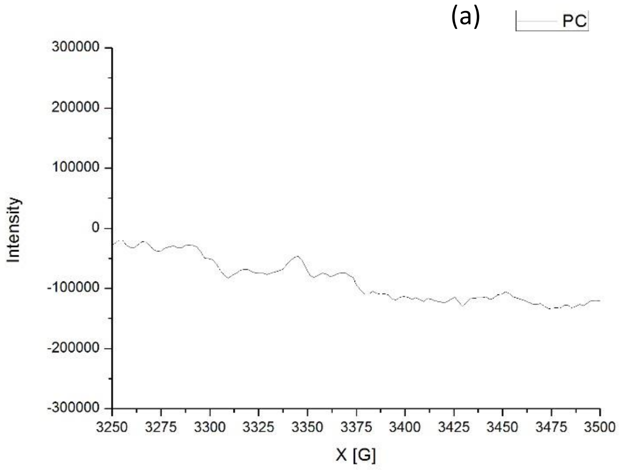

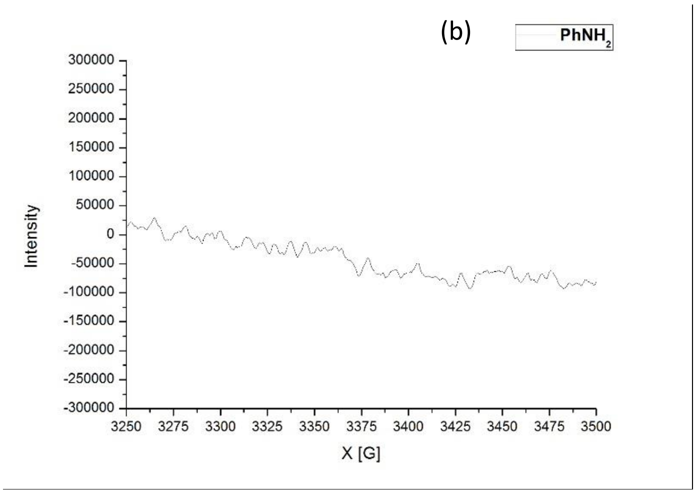

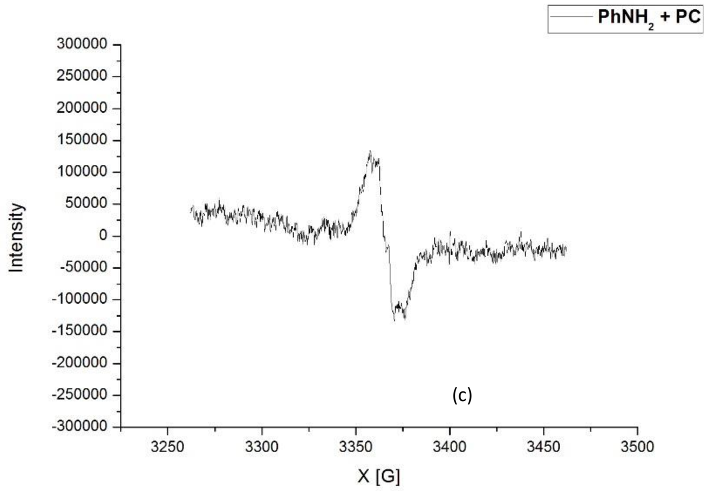

Supplementary Figure 18. EPR experiments. (a): PC (Ir(ppy)$_2$(dtbpy)PF$_6$, 0.005 mol, 5 mol %); (b): PhNH$_2$ (**2a**, 0.1 mol), and (c): [PhNH$_2$ (**2a**, 0.1 mmol) + PC (Ir(ppy)$_2$(dtbpy)PF$_6$, 5 mol %)] in HFIP (1 ml), respectively. Stirred at room temperature under violet LED (24-W, 390-410 nm) irradiation for 1h, and the mixture were directly used for EPR experiments.

Proposed mechanism.

"condition A"

Based on our investigations and previous reports,[6] a plausible mechanism was proposed in Supplementary Figure 19. The reaction starts with the oxidation of **2** by the excited-state Ir(III)* in the presence of base, yielding amine radical **A** and Ir(II). Then, the Ir(II) ($E_{1/2}^{III/II}$ = −1.51 V vs SCE)[7-9] species facilitated the second SET process of substrate **1** ($Ep^{0/-1}$(**1a**) = -0.84 V versus SCE in MeCN)[7-9] to generate the amidyl radical **B**. The subsequently 1,5-HAT formed the radical intermediate **C** along with the oxidation of Ir(II) to Ir(III) to close the catalytic cycle. Finally, the radical-radical cross-coupling between N-center radical **A** and C-center radical intermediate **C** was proposed to provide the sp^3 C-N cross coupling product **3**.

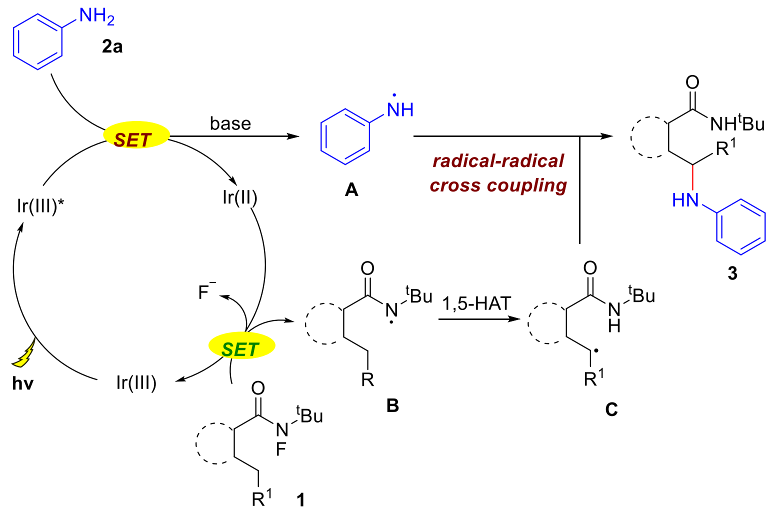

Supplementary Figure 19. Proposed mechanism of amine.

"condition B"

Based on our investigations and previous reports,[6] a plausible mechanism was proposed in Supplementary Figure 20. The reaction starts with the oxidation of **7** by the excited-state Ir(III)* in the presence of base, yielding alkyl radical **A** and Ir(II). Then, the Ir(II) ($E_{1/2}^{III/II}$ = −1.51 V vs SCE)[7-9] species facilitated the second SET process of substrate **1** ($Ep^{0/-1}$(**1a**) = -0.84 V versus SCE in MeCN)[7-9] to generate the amidyl radical **B**. The subsequently 1,5-HAT formed the radical intermediate **C** along with the oxidation of Ir(II) to Ir(III) to close the catalytic cycle. Finally, the radical-radical cross-coupling between alkyl radical **A** and C-center radical intermediate **C** was proposed to provide the sp^3 C-C cross coupling product **3**.

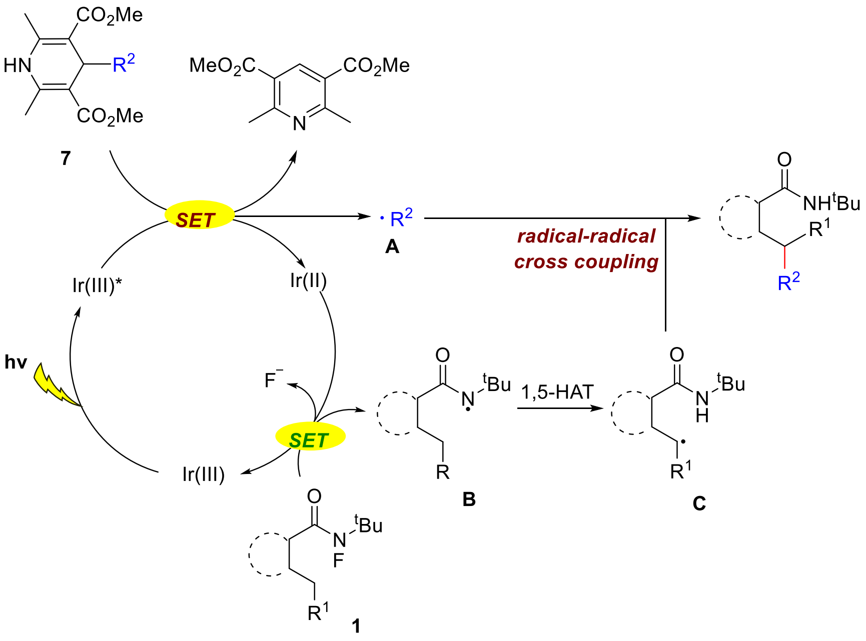

Supplementary Figure 20. Proposed mechanism of Hantzsch esters.

The wavelength of the violet LED (24-W, 390-410 nm).

The wavelength of the violet LED light we used (recorded on an AVANTES® AvaSpec-ULS2048 spectrometer instrument):

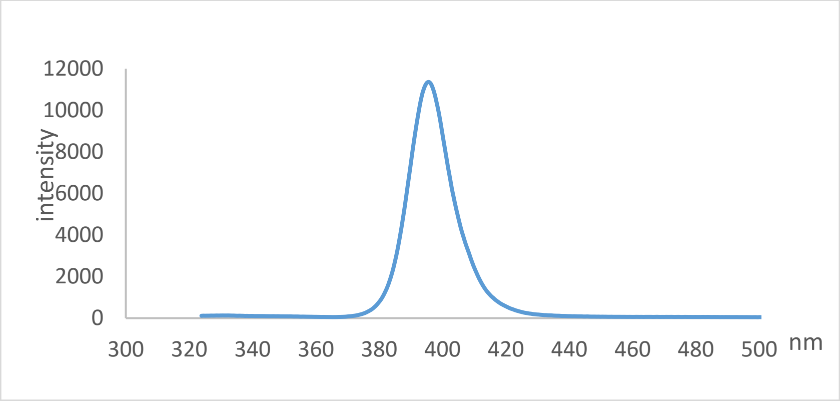

Supplementary Figure 21. The wavelength of the violet LED.

Characterization of products.

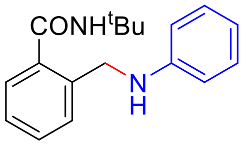

3a

***N*-(tert-butyl)-2-((phenylamino)methyl)benzamide (3a),** 41.2 mg, yield: 73%. Yellow oil.
^{1}HNMR (300 MHz, $CDCl_3$): δ 7.61-7.64 (m, 1H), 7.37 (s, 3H), 7.21 (t, *J* = 8.1 Hz, 2H), 7.05 (s, 1H), 6.79 (t, *J* = 7.2 Hz, 1H), 6.71 (d, *J* = 7.8 Hz, 2H), 4.34 (s, 2H), 4.13 (br, 1H), 1.34 (s, 9H).
^{13}CNMR (75 MHz, $CDCl_3$): δ 168.44, 147.38, 137.90, 135.73, 130.14, 129.33, 128.82, 128.04, 118.80, 114.56, 113.70, 51.49, 47.32, 28.67.
HRMS (ESI): $C_{18}H_{22}N_2O+Na^+$ Calcd: 305.1624, Found: 305.1615.

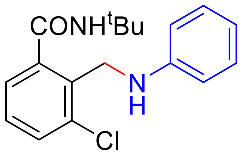

3b

***N*-(tert-butyl)-3-chloro-2-((phenylamino)methyl)benzamide (3b),** 43.7 mg, yield: 69%. White solid, mp: 172-173°C.
^{1}HNMR (300 MHz, $CDCl_3$): δ 7.53 (d, *J* = 7.5 Hz, 1H), 7.46 (d, *J* = 7.8 Hz, 1H), 7.22-7.34 (m, 3H), 7.02 (s, 1H), 6.84 (t, *J* = 7.2 Hz, 1H), 6.77 (d, *J* = 7.8 Hz, 2H), 4.46 (s, 2H), 4.05 (br, 1H), 1.30 (s, 9H).
^{13}CNMR (75 MHz, $CDCl_3$): δ 167.51, 147.27, 140.89, 135.24, 132.73, 131.06, 129.36, 129.24, 127.61, 119.31, 114.05, 51.70, 43.90, 28.48.
HRMS (ESI): $C_{18}H_{21}ClN_2O+Na^+$ Calcd: 339.1235, Found: 339.1227.

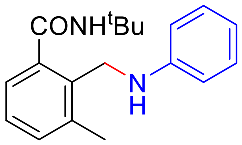

3c

***N*-(tert-butyl)-3-methyl-2-((phenylamino)methyl)benzamide (3c),** 38.5 mg, yield: 65%. Yellow oil.
^{1}HNMR (300 MHz, $CDCl_3$): δ 7.44 (t, *J* = 4.2 Hz, 1H), 7.21-7.27 (m, 3H), 6.78-6.84 (m, 2H), 6.74 (d, *J* = 8.1 Hz, 2H), 4.26 (s, 2H), 3.90 (br, 1H), 2.42 (s, 3H), 1.30 (s, 9H).
^{13}CNMR (75 MHz, $CDCl_3$): δ 168.98, 147.69, 139.61, 137.58, 133.00, 132.00, 129.36, 128.02, 126.43, 118.79, 113.64, 51.52, 43.53, 28.59, 19.39.
HRMS (ESI): $C_{19}H_{24}N_2O+Na^+$ Calcd: 319.1781, Found: 319.1773.

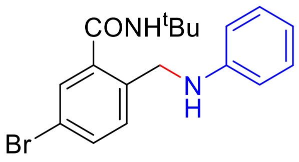

3d

5-bromo-*N*-(tert-butyl)-2-((phenylamino)methyl)benzamide (3d), 48.4 mg, yield: 67%. White solid, mp: 114-117°C.

^{1}HNMR (300 MHz, $CDCl_3$): δ 7.72 (s, 1H), 7.49 (d, J = 8.1 Hz, 1H), 7.17-7.23 (m, 3H), 6.87 (s, 1H), 6.79 (t, J = 7.2 Hz, 1H), 6.67 (d, J = 7.8 Hz, 2H), 4.31 (s, 2H), 4.09 (br, 1H), 1.34 (s, 9H).

^{13}CNMR (75 MHz, $CDCl_3$): δ 166.92, 147.10, 139.50, 134.93, 133.02, 131.65, 129.37, 121.82, 118.97, 113.67, 51.77, 46.70, 28.61.

HRMS (ESI): $C_{18}H_{21}BrN_2O+Na^+$ Calcd: 383.0729, Found: 383.0715.

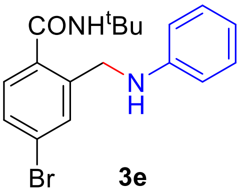

3e

4-bromo-*N*-(tert-butyl)-2-((phenylamino)methyl)benzamide (3e), 54.2 mg, yield: 75%. Yellow oil.

^{1}HNMR (300 MHz, $CDCl_3$): δ 7.53 (s, 1H), 7.49 (s, 2H), 7.19-7.26 (m, 2H), 7.00 (s, 1H), 6.81 (t, J = 7.5 Hz, 1H), 6.70 (d, J = 7.5 Hz, 2H), 4.31 (s, 2H), 4.11 (br, 1H), 1.33 (s, 9H).

^{13}CNMR (75 MHz, $CDCl_3$): δ 167.51, 147.03, 138.05, 136.61, 132.85, 131.08, 130.47, 129.40, 124.22, 119.10, 113.69, 51.67, 46.92, 28.63.

HRMS (ESI): $C_{18}H_{21}BrN_2O+Na^+$ Calcd: 383.0729, Found: 383.0712.

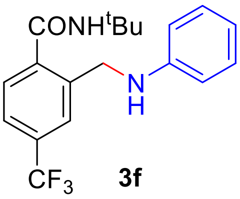

3f

***N*-(tert-butyl)-2-((phenylamino)methyl)-4-(trifluoromethyl)benzamide (3f),** 35.7 mg, yield: 51%. Yellow solid, mp: 153-155°C.

^{1}HNMR (300 MHz, $CDCl_3$): δ 7.88 (d, J = 7.8 Hz, 1H), 7.77 (d, J = 7.5 Hz, 1H), 7.62 (br, 1H), 7.51 (t, J = 7.8 Hz, 1H), 7.24-7.30 (m, 2H), 6.89 (t, J = 7.5 Hz, 1H), 6.78 (d, J = 7.8 Hz, 2H), 4.39 (s, 2H), 3.97 (br, 1H), 1.24 (s, 9H).

^{19}FNMR (282 MHz) δ -58.34.

^{13}CNMR (75 MHz, $CDCl_3$): δ 167.14, 146.81, 141.77, 133.44, 132.59, 129.43, 128.50, 127.50 (q, J = 6.0 Hz), 126.03(t, J = 273.0 Hz), 119.98, 114.30, 51.63, 43.07, 28.35.

HRMS (ESI): $C_{19}H_{21}F_3N_2O+Na^+$ Calcd: 373.1498, Found: 373.1502.

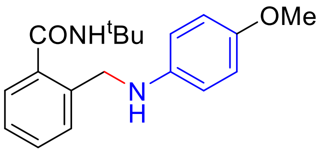

3g

***N*-(tert-butyl)-2-(((4-methoxyphenyl)amino)methyl)benzamide (3g),** 24.9 mg, yield: 40%. White oil liquid.

^{1}HNMR (300 MHz, $CDCl_3$): δ 7.61-7.65 (m, 1H), 7.54 (br, 1H), 7.24-7.33 (m, 3H), 6.75 (d, *J* = 9 Hz, 2H), 6.63 (d, *J* = 8.7 Hz, 2H), 4.18 (s, 2H), 3.70 (s, 3H), 1.24 (s, 9H).

^{13}CNMR (75 MHz, $CDCl_3$): δ 167.06, 152.15, 140.12, 137.10, 134.14, 129.28, 129.13, 128.32, 127.20, 114.15, 113.79, 54.64, 50.32, 47.41, 27.65.

HRMS (ESI): $C_{19}H_{24}N_2O_2$+Na^+ Calcd: 335.1703, Found: 335.1734.

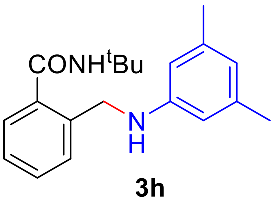

3h

***N*-(tert-butyl)-2-(((3,5-dimethylphenyl)amino)methyl)benzamide (3h),** 39.7 mg, yield: 64%. White solid, mp: 128-131°C.

^{1}HNMR (300 MHz, $CDCl_3$): δ 7.63-7.66 (m, 1H), 7.34-7.37 (m, 3H), 7.22 (s, 1H), 6.47 (s, 1H), 6.35 (s, 2H), 4.30 (s, 2H), 3.93 (br, 1H), 2.25 (s, 6H), 1.34 (s, 9H).

^{13}CNMR (75 MHz, $CDCl_3$): δ 168.33, 147.45, 138.97, 137.94, 135.68, 130.15, 128.96, 128.04, 120.91, 111.75, 51.44, 47.52, 28.68, 21.50.

HRMS (ESI): $C_{20}H_{26}N_2O$+Na^+ Calcd: 333.1937, Found: 333.1926.

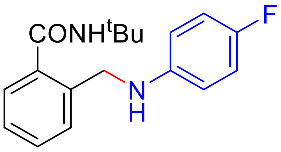

3i

***N*-(tert-butyl)-2-(((4-fluorophenyl)amino)methyl)benzamide (3i),** 37.8 mg, yield: 63%. White solid, mp: 147-150°C.

^{1}HNMR (300 MHz, $CDCl_3$): δ 7.61-7.64 (m, 1H), 7.35-7.38 (m, 3H), 7.04 (s, 1H), 6.93 (t, *J* = 8.7 Hz, 2H), 6.63-6.68 (m, 2H), 4.29 (s, 2H), 4.06 (br, 1H), 1.34 (s, 9H).

^{19}FNMR (282 MHz) δ -126.16.

^{13}CNMR (75 MHz, $CDCl_3$): δ 168.43, 143.66, 137.88, 135.51, 130.17, 128.82, 128.13, 115.96, 115.5.66, 114.68, 114.58, 51.51, 47.93, 28.69.

HRMS (ESI): $C_{18}H_{21}FN_2O$+Na^+ Calcd: 323.1530, Found: 323.1519.

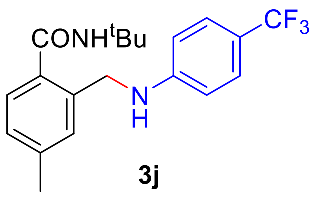

3j

***N*-(tert-butyl)-4-methyl-2-(((4-(trifluoromethyl)phenyl)amino)methyl)benzamide (3j),** 48.1 mg, yield: 66%. White solid, mp: 174-175℃.

^{1}HNMR (300 MHz, $CDCl_3$): δ 7.39 (d, *J* = 8.7 Hz, 3H), 7.19 (s, 1H), 7.12 (d, *J* = 7.8 Hz, 1H), 6.66 (d, *J* = 8.4 Hz, 2H), 6.15 (s, 1H), 4.76 (br, 1H), 4.41 (d, *J* = 5.7 Hz, 2H), 2.36 (s, 3H), 1.39 (s, 9H).

^{19}FNMR (282 MHz) δ -61.04.

^{13}CNMR (75 MHz, $CDCl_3$): δ 169.16, 150.28, 140.45, 136.44, 134.42, 130.68, 128.42, 127.96, 126.63, 126.58, 112.35, 51.68, 46.45, 28.75, 21.29.

HRMS (ESI): $C_{20}H_{23}F_3N_2O+Na^+$ Calcd: 387.1655, Found: 387.1645.

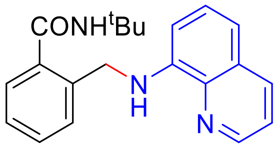

3k

***N*-(tert-butyl)-2-((quinolin-8-ylamino)methyl)benzamide (3k),** 38.0 mg, yield: 57%. White oil liquid.

^{1}HNMR (300 MHz, $CDCl_3$): δ 8.69-8.71 (m, 1H), 8.10 (d, *J* = 8.4 Hz, 1H), 7.60-7.63 (m, 1H), 7.35-7.49 (m, 5H), 7.15 (d, *J* = 7.8 Hz, 1H), 6.82 (d, *J* = 7.8 Hz, 2H), 6.41 (br, 1H), 4.58 (s, 2H), 1.27 (s, 9H).

^{13}CNMR (75 MHz, $CDCl_3$): δ 168.48, 147.14, 143.94, 137.99, 136.09, 135.45, 130.08, 129.92, 128.57, 128.50, 127.92, 127.66, 121.50, 115.40, 106.18, 51.53, 46.38, 28.53.

HRMS (ESI): $C_{21}H_{23}N_3O+Na^+$ Calcd: 356.1733, Found: 356.1731.

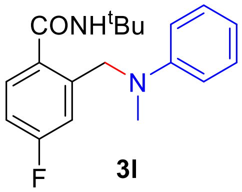

3l

***N*-(tert-butyl)-4-fluoro-2-((methyl(phenyl)amino)methyl)benzamide (3l),** 40.9 mg, yield: 65%. Yellow oil.

^{1}HNMR (300 MHz, $CDCl_3$): δ 7.43-7.48 (m, 1H), 7.20-7.23 (m, 2H), 6.94-6.99 (m, 2H), 6.72-6.79 (m, 3H), 6.05 (s, 1H), 4.63 (s, 2H), 3.00 (s, 3H), 1.42 (s, 9H).

^{19}FNMR (282 MHz) δ -110.05.

^{13}CNMR (75 MHz, $CDCl_3$): δ 167.98, 149.47, 140.60 (d, *J* = 6.8 Hz), 132.80, 129.45 (d, *J* = 9.0 Hz), 129.19, 117.51, 115.16 (d, *J* = 22.5 Hz), 113.65 (d, *J* = 21 Hz), 113.00, 54.82, 51.84, 38.91, 28.82.

HRMS (ESI): $C_{19}H_{23}FN_2O+Na^+$ Calcd: 337.1687, Found: 337.1680.

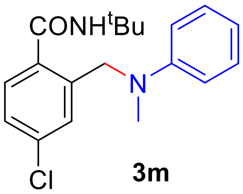

3m

***N*-(tert-butyl)-4-chloro-2-((methyl(phenyl)amino)methyl)benzamide (3m),** 45.0 mg, yield: 68%. White solid, mp: 109-112℃.

^{1}HNMR (300 MHz, $CDCl_3$): δ 7.41 (d, *J* = 8.7 Hz, 1H), 7.21-7.27 (m, 4H), 6.74-6.80 (m, 3H), 6.13 (s, 1H), 4.58 (s, 2H), 2.98 (s, 3H), 1.40 (s, 9H).

^{13}CNMR (75 MHz, $CDCl_3$): δ 167.87, 149.59, 139.20, 136.07, 135.19, 129.19, 128.90, 128.28, 127.16, 117.73, 113.25, 54.89, 51.90, 38.90, 28.78.

HRMS (ESI): $C_{19}H_{23}ClN_2O+Na^+$ Calcd: 353.1391, Found: 353.1381.

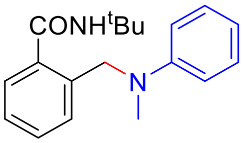

3n

***N*-(tert-butyl)-2-((methyl(phenyl)amino)methyl)benzamide (3n),** 36.2 mg, yield: 61%. Yellow oil.

^{1}HNMR (300 MHz, $CDCl_3$): δ 7.46-7.49 (m, 1H), 7.20-7.35 (m, 5H), 6.77 (d, *J* = 8.7 Hz, 3H), 6.19 (s, 1H), 4.63 (s, 2H), 2.98 (s, 3H), 1.40 (s, 9H).

^{13}CNMR (75 MHz, $CDCl_3$): δ 168.86, 149.78, 137.01, 136.55, 129.88, 129.13, 128.41, 127.50, 127.03, 117.34, 113.16, 54.75, 51.72, 38.73, 28.82.

HRMS (ESI): $C_{19}H_{24}N_2O+Na^+$ Calcd: 319.1781, Found: 319.1771.

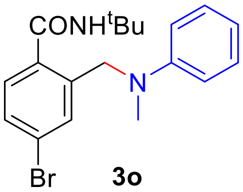

3o

4-bromo-*N*-(tert-butyl)-2-((methyl(phenyl)amino)methyl)benzamide (3o), 36.8 mg, yield: 49%. Yellow oil.

^{1}HNMR (300 MHz, $CDCl_3$): δ 7.41-7.43 (m, 2H), 7.32-7.35 (m, 1H), 7.21-7.24 (m, 2H), 6.74-6.81 (m, 3H), 6.11 (s, 1H), 4.57 (s, 2H), 2.97 (s, 3H), 1.39 (s, 9H).

^{13}CNMR (75 MHz, $CDCl_3$): δ 167.90, 149.66, 139.32, 135.74, 131.23, 130.20, 129.20, 129.06, 124.38,

117.81, 113.34, 54.91, 51.91, 38.89, 28.79.

HRMS (ESI): $C_{19}H_{23}BrN_2O+Na^+$ Calcd: 397.0886, Found: 397.0874.

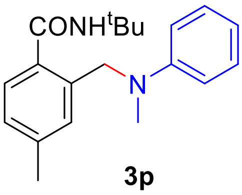

3p

***N*-(tert-butyl)-4-methyl-2-((methyl(phenyl)amino)methyl)benzamide (3p),** 44.7 mg, yield: 72%. White solid, mp: 105-108°C.

^{1}HNMR (300 MHz, $CDCl_3$): δ 7.39 (d, *J* = 7.5 Hz, 1H), 7.22 (d, *J* = 7.5 Hz, 2H), 7.06-7.10 (m, 2H), 6.73-6.81 (m, 3H), 6.22 (s, 1H), 4.58 (s, 2H), 2.95 (s, 3H), 2.30 (s, 3H), 1.38 (s, 9H).

^{13}CNMR (75 MHz, $CDCl_3$): δ 168.91, 149.99, 140.01, 136.53, 134.27, 129.11, 127.73, 127.66, 117.38, 113.29, 112.51, 54.96, 51.57, 38.60, 28.82, 21.46.

HRMS (ESI): $C_{20}H_{26}N_2O+Na^+$ Calcd: 333.1937, Found: 333.1931.

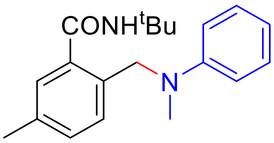

3q

***N*-(tert-butyl)-5-methyl-2-((methyl(phenyl)amino)methyl)benzamide (3q),** 45.9 mg, yield: 74%. White solid, mp: 105-108°C.

^{1}HNMR (300 MHz, $CDCl_3$): δ 7.30 (s, 1H), 7.21 (d, *J* = 7.5 Hz, 2H), 7.12 (s, 2H), 6.72-6.81 (m, 3H), 6.27 (s, 1H), 4.55 (s, 2H), 2.94 (s, 3H), 2.34 (s, 3H), 1.38 (s, 9H).

^{13}CNMR (75 MHz, $CDCl_3$): δ 168.93, 149.88, 137.04, 136.89, 133.13, 130.46, 129.10, 128.74, 128.36, 117.45, 113.39, 54.51, 51.61, 38.57, 28.79, 20.90.

HRMS (ESI): $C_{20}H_{26}N_2O+Na^+$ Calcd: 333.1937, Found: 333.1929.

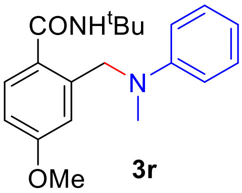

3r

***N*-(tert-butyl)-4-methoxy-2-((methyl(phenyl)amino)methyl)benzamide (3r),** 41.8 mg, yield: 64%. Yellow oil.

^{1}HNMR (300 MHz, $CDCl_3$): δ 7.47 (d, *J* = 7.8 Hz, 1H), 7.21 (d, *J* = 7.5 Hz, 2H), 6.77 (d, *J* = 8.7 Hz, 5H), 6.22 (s, 1H), 4.61 (s, 2H), 3.74 (s, 3H), 2.97 (s, 3H), 1.39 (s, 9H).

^{13}CNMR (75 MHz, $CDCl_3$): δ 168.56, 160.85, 149.82, 139.09, 129.46, 129.34, 129.10, 117.43, 114.38, 113.27, 111.40, 55.27, 55.16, 51.53, 38.71, 28.84.

HRMS (ESI): $C_{20}H_{26}N_2O_2+Na^+$ Calcd: 349.1886, Found: 349.1878.

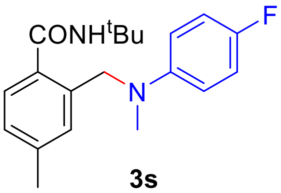

3s

***N*-(tert-butyl)-2-(((4-fluorophenyl)(methyl)amino)methyl)-4-methylbenzamide (3s),** 41.4 mg, yield: 63%. White solid, mp: 147-150°C.

^{1}HNMR (300 MHz, $CDCl_3$):δ 7.41 (d, *J* = 7.8 Hz, 1H), 7.09 (d, *J* = 7.8 Hz, 1H), 7.03 (s, 1H), 6.93 (t, *J* = 9.0 Hz, 2H), 6.71-6.77 (m, 2H), 6.38 (s, 1H), 4.48 (s, 2H), 2.89 (s, 3H), 2.31 (s, 3H), 1.37 (s, 9H).

^{19}FNMR (282 MHz): δ -127.59.

^{13}CNMR (75 MHz, $CDCl_3$): δ 168.82, 157.66, 154.52, 146.68, 139.98, 136.16, 134.40, 129.56, 127.92 (d, *J* = 12.0 Hz), 115.45 (d, *J* = 21.2Hz), 114.99 (d, *J* = 7.5 Hz), 55.92, 51.53, 39.29, 28.83, 21.42.

HRMS (ESI): $C_{20}H_{25}FN_2O+Na^+$ Calcd: 351.1843, Found: 351.1833.

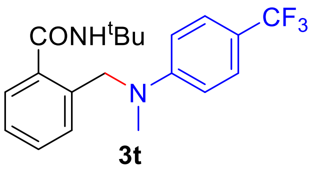

3t

***N*-(tert-butyl)-2-((methyl(4-(trifluoromethyl)phenyl)amino)methyl)benzamide (3t),** 50.3 mg, yield: 69%. White solid, mp: 127-130°C.

^{1}HNMR (300 MHz, $CDCl_3$): δ 7.41 (d, *J* = 8.1 Hz, 3H), 7.26-7.35 (m, 2H), 7.15 (d, *J* = 7.2 Hz, 1H), 6.70 (d, *J* = 8.4 Hz, 2H), 5.78 (s, 1H), 4.79 (s, 2H), 3.09 (s, 3H), 1.46 (s, 9H).

^{19}FNMR (282 MHz): δ -60.87.

^{13}CNMR (75 MHz, $CDCl_3$): δ 168.90, 151.59, 136.38, 130.14, 127.12, 127.04, 127.00, 126.89, 126.44, 126.39, 111.31, 54.09, 51.92, 38.86, 28.87.

HRMS (ESI): $C_{20}H_{23}F_3N_2O+Na^+$ Calcd: 387.1655, Found: 387.1646.

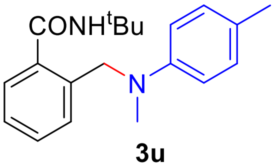

3u

***N*-(tert-butyl)-2-((methyl(p-tolyl)amino)methyl)benzamide (3u),** 39.7 mg, yield: 64%. White solid, mp: 118-121°C.

^{1}HNMR (300 MHz, $CDCl_3$): δ 7.50-7.53 (m, 1H), 7.21-7.32 (m, 3H), 7.04 (d, *J* = 8.1 Hz, 2H), 6.72 (d, *J* = 8.4 Hz, 2H), 6.51 (s, 1H), 4.52 (s, 2H), 2.90 (s, 3H), 2.25 (s, 3H), 1.38 (s, 9H).

^{13}CNMR (75 MHz, $CDCl_3$): δ 168.77, 147.78, 137.27, 136.26, 129.81, 129.65, 129.04, 127.90, 127.16, 114.00, 55.33, 51.62, 39.01, 28.80, 20.30.

HRMS (ESI): $C_{20}H_{26}N_2O+Na^+$ Calcd: 333.1937, Found: 333.1927.

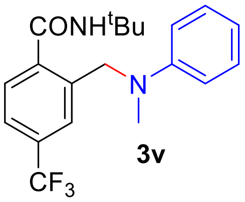

3v

***N*-(tert-butyl)-2-((methyl(phenyl)amino)methyl)-4-(trifluoromethyl)benzamide (3v),** 45.2mg, yield: 62%. Pale yellow solid, mp: 115-117°C.

^{1}HNMR (300 MHz, $CDCl_3$): δ 7.74 (d, *J* = 7.8 Hz, 1H), 7.59 (d, *J* = 7.2 Hz, 1H), 7.43 (t, *J* = 7.8 Hz, 1H), 7.21-7.27 (m, 2H), 6.87 (d, *J* = 8.4 Hz, 2H), 6.80 (t, *J* = 7.5 Hz, 1H), 5.89 (br, 1H), 4.64 (s, 2H), 2.70 (s, 3H), 1.19 (s, 9H).

^{19}FNMR (282 MHz, $CDCl_3$): δ -58.01.

^{13}CNMR (75 MHz, $CDCl_3$): δ 168.10, 150.07, 140.53, 135.71, 132.35, 129.87, 127.63, 126.96 (q, *j* = 6.0 Hz), 125.93 (t, *J* = 266.25 Hz), 118.55, 114.30, 51.60, 50.88 (d, *J* = 2.3 Hz), 37.53, 28.69.

HRMS (ESI): $C_{20}H_{23}F_3N_2O+Na^+$ Calcd: 387.1655, Found: 387.1661.

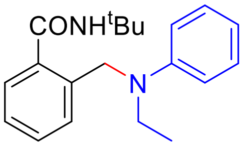

3w

***N*-(tert-butyl)-2-((ethyl(phenyl)amino)methyl)benzamide (3w),** 35.4 mg, yield: 57%. White solid, mp: 114-117°C.

^{1}HNMR (300 MHz, $CDCl_3$): δ 7.37-7.40 (m, 1H), 7.18-7.24 (m, 3H), 7.12 (t, *J* = 7.8 Hz, 2H), 6.60-6.65 (m, 3H), 6.07 (br, 1H), 4.55 (s, 2H), 3.37 (q, *J* = 7.2 Hz, 2H), 1.34 (s, 9H), 1.09 (t, *J* = 6.9 Hz, 3H).

^{13}CNMR (75 MHz, $CDCl_3$): δ 168.94, 148.25, 136.74, 129.92, 129.20, 128.14, 127.40, 126.92, 116.73, 112.96, 51.81, 51.72, 44.98, 28.83, 11.72.

HRMS (ESI): $C_{20}H_{26}N_2O+Na^+$ Calcd: 333.1937, Found: 333.1944.

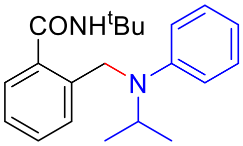

3x

***N*-(tert-butyl)-2-((isopropyl(phenyl)amino)methyl)benzamide (3x),** 33.1 mg, yield: 51%. White solid, mp: 144-146°C.

^{1}HNMR (300 MHz, $CDCl_3$): δ 7.38 (t, *J* = 7.8 Hz, 2H), 7.28-7.31 (m, 1H), 7.23 (d, *J* = 7.5 Hz, 1H), 7.17 (t, *J* = 7.8 Hz, 2H), 6.65-6.89 (m, 3H), 5.78 (br, 1H), 4.59 (s, 2H), 4.21-4.30 (m, 1H), 1.49 (s, 9H), 1.20 (d, *J* = 6.6 Hz, 6H).

^{13}CNMR (75 MHz, $CDCl_3$): δ 169.26, 149.14, 138.90, 135.60, 129.88, 129.11, 127.72, 126.30, 116.37, 113.15, 51.86, 48.19, 46.24, 28.92, 19.74.

HRMS (ESI): $C_{21}H_{28}N_2O+Na^+$ Calcd: 347.2094, Found: 347.2111.

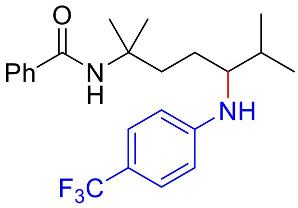

3y

***N*-(2,6-dimethyl-5-((4-(trifluoromethyl)phenyl)amino)heptan-2-yl)benzamide (3y),** 41.5 mg, yield: 51%. Colorless oil.

^{1}HNMR (300 MHz, $CDCl_3$): δ 7.65 (d, *J* = 6.9 Hz, 2H), 7.37-7.51 (m, 3H), 7.30 (d, *J* = 8.7 Hz, 2H), 6.56 (d, *J* = 8.7 Hz, 2H), 5.81 (s, 1H), 3.97 (d, *J* = 7.2 Hz, 1H), 3.23 (br, 1H), 1.88-1.95 (m, 1H), 1.80-1.86 (m, 2H), 1.38-1.40 (m, 6H), 1.25-1.32 (m, 2H), 0.88-0.95 (m, 6H).

^{19}FNMR (282 MHz) δ -60.85.

^{13}CNMR (75 MHz, $CDCl_3$): δ 167.06, 150.95, 135.75, 131.22, 128.56, 126.64, 126.59, 117.82 (d, *J* = 32.3 Hz), 111.89, 58.16, 54.11, 35.73, 31.10, 27.61, 27.45, 25.69, 18.79, 18.18

HRMS (ESI): $C_{23}H_{29}F_3N_2O+Na^+$ Calcd: 429.2124, Found: 429.2119.

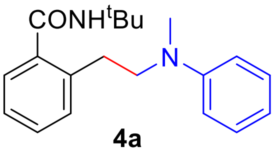

4a

***N*-(tert-butyl)-2-(2-(methyl(phenyl)amino)ethyl)benzamide (4a),** 46.6 mg, yield: 75%. White solid, mp: 53-56°C.

^{1}HNMR (300 MHz, $CDCl_3$): δ 7.32 (d, *J* = 6.9 Hz, 2H), 7.21-7.26 (m, 4H), 6.77 (d, *J* = 8.4 Hz, 2H), 6.68 (t, *J* = 7.2 Hz, 1H), 5.61 (s, 1H), 3.63 (t, *J* = 7.5 Hz, 2H), 2.98 (t, *J* = 8.1 Hz, 2H), 2.92 (s, 3H), 1.47 (s, 9H).

^{13}CNMR (75 MHz, $CDCl_3$): δ 169.67, 148.87, 138.14, 137.65, 130.90, 129.67, 129.23, 126.60, 126.21, 115.90, 112.03, 54.59, 51.84, 38.21, 30.67, 28.84.

HRMS (ESI) $C_{20}H_{26}N_2O+Na^+$ Calcd: 333.1937, Found: 333.1935.

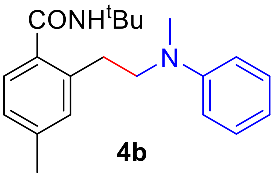

4b

***N*-(tert-butyl)-4-methyl-2-(2-(methyl(phenyl)amino)ethyl)benzamide (4b),** 43.5 mg, yield: 67%. Yellow oil.

^{1}HNMR (300 MHz, $CDCl_3$):δ 7.20-7.27 (m, 3H), 7.00-7.03 (m, 2H), 6.78 (d, *J* = 8.1 Hz, 2H), 6.68 (t, *J* = 7.2 Hz, 1H), 5.59 (s, 1H), 3.61 (t, *J* = 7.5 Hz, 2H), 2.92-2.98 (m, 5H), 2.34 (s, 3H), 1.46 (s, 9H).

^{13}CNMR (75 MHz, $CDCl_3$): δ 169.77, 148.91, 139.68, 137.69, 135.33, 131.61, 129.23, 126.80, 126.68, 115.86, 112.04, 54.60, 51.73, 38.17, 30.59, 28.85, 21.25.

HRMS (ESI) $C_{21}H_{28}N_2O+Na^+$ Calcd: 347.2094, Found: 347.2092.

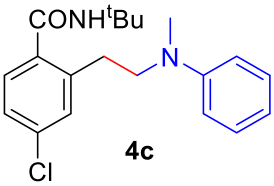

4c

***N*-(tert-butyl)-4-chloro-2-(2-(methyl(phenyl)amino)ethyl)benzamide (4c),** 51.0 mg, yield: 74%. White solid, mp: 99-102°C.

^{1}HNMR (300 MHz, $CDCl_3$): δ 7.16-7.28 (m, 5H), 6.76 (d, *J* = 8.1 Hz, 2H), 6.70 (t, *J* = 7.2 Hz, 1H), 5.61 (s, 1H), 3.61 (t, *J* = 7.5 Hz, 2H), 2.91-2.97 (m, 5H), 1.45 (s, 9H).

^{13}CNMR (75 MHz, $CDCl_3$): δ 168.67, 148.74, 139.89, 136.48, 135.34, 130.80, 129.28, 128.03, 126.31, 116.18, 112.12, 54.40, 52.01, 38.26, 30.57, 28.79.

HRMS (ESI): $C_{20}H_{25}ClN_2O$+Na^+ Calcd: 367.1548, Found: 367.1544.

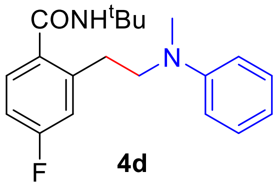

4d

***N*-(tert-butyl)-4-fluoro-2-(2-(methyl(phenyl)amino)ethyl)benzamide (4d),** 47.3 mg, yield: 72%. White solid, mp: 63-66°C.

^{1}HNMR (300 MHz, $CDCl_3$):δ 7.21-7.31 (m, 3H), 6.90-6.95 (m, 2H), 6.76 (d, *J* = 8.1 Hz, 2H), 6.69 (t, *J* = 7.2 Hz, 1H), 5.57 (s, 1H), 3.63 (t, *J* = 7.5 Hz, 2H), 2.98 (t, *J* = 7.8 Hz, 2H), 2.92 (s, 3H), 1.46 (s, 9H).

^{19}FNMR (282 MHz): δ -111.22.

^{13}CNMR (75 MHz, $CDCl_3$): δ 168.80, 164.72, 161.42, 148.77, 140.91 (d, *J* = 5.3 Hz), 134.28, 129.27, 128.61 (d, *J* = 9 Hz), 117.58 (d, *J* = 21 Hz), 116.15, 113.02(d, *J* = 21 Hz), 112.11, 54.37, 51.92, 38.25, 30.77, 28.80.

HRMS (ESI): $C_{20}H_{25}FN_2O$+Na^+ Calcd: 351.1843, Found: 351.1841.

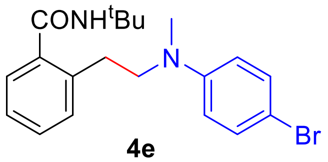

4e

2-(2-((4-bromophenyl)(methyl)amino)ethyl)-*N*-(tert-butyl)benzamide (4e), 52.9 mg, yield: 68%. White solid, mp: 70-72°C.

^{1}HNMR (300 MHz, $CDCl_3$): δ 7.17-7.35 (m, 6H), 6.64 (d, *J* = 9.0 Hz, 2H), 5.60 (s, 1H), 3.61 (t, *J* = 7.5 Hz, 2H), 2.94 (t, *J* = 8.1 Hz, 2H), 2.89 (s, 3H), 1.47 (s, 9H).

^{13}CNMR (75 MHz, $CDCl_3$): δ 169.60, 147.87, 138.08, 137.44, 131.80, 130.93, 129.73, 126.59, 126.33, 113.67, 107.73, 54.57, 51.87, 38.31, 30.63, 28.85.

HRMS (ESI): $C_{20}H_{25}BrN_2O$+Na^+ Calcd: 411.1042, Found: 411.1033.

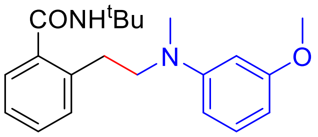

4f

***N*-(tert-butyl)-2-(2-((3-methoxyphenyl)(methyl)amino)ethyl)benzamide (4f),** 49.7 mg, yield: 73%. Yellow oil.

¹HNMR (300 MHz, $CDCl_3$): δ 7.23-7.28 (m, 2H), 7.04-7.19 (m, 3H), 6.31-6.35 (m, 1H), 6.17-6.24 (m, 2H), 5.53 (s, 1H), 3.74 (s, 3H), 3.55 (t, *J* = 7.2 Hz, 2H), 2.91 (t, *J* = 8.1 Hz, 2H), 2.83 (s, 3H), 1.39 (s, 9H).

¹³CNMR (75 MHz, $CDCl_3$): δ 168.61, 159.85, 149.32, 137.06, 136.66, 129.92, 128.84, 128.65, 125.56, 125.19, 104.18, 99.95, 97.46, 54.11, 53.57, 50.79, 37.25, 29.82, 27.79.

HRMS (ESI): $C_{21}H_{28}N_2O_2$+Na^+ Calcd: 363.2043, Found: 363.2039.

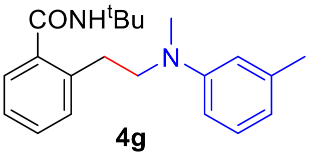

4g

***N*-(tert-butyl)-2-(2-(methyl(m-tolyl)amino)ethyl)benzamide (4g),** 43.5 mg, yield: 67%. Yellow oil.

¹HNMR (300 MHz, $CDCl_3$): δ 7.32 (d, *J* = 7.2 Hz, 2H), 7.15-7.25 (m, 2H), 7.09-7.13 (m, 1H), 6.59 (s, 2H), 6.52 (d, *J* = 7.2 Hz, 1H), 5.59 (s, 1H), 3.61 (t, *J* = 7.2 Hz, 2H), 2.98 (t, *J* = 7.8 Hz, 2H), 2.90 (s, 3H), 2.32 (s, 3H), 1.46 (s, 9H).

¹³CNMR (75 MHz, $CDCl_3$): δ 169.63, 149.00, 138.84, 138.15, 137.79, 130.88, 129.64, 129.06, 126.59, 126.17, 116.93, 112.83, 109.30, 54.65, 51.80, 38.25, 30.79, 28.83, 21.95.

HRMS (ESI): $C_{21}H_{28}N_2O$+Na^+ Calcd: 347.2094, Found: 347.2086.

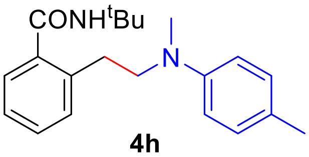

4h

***N*-(tert-butyl)-2-(2-(methyl(p-tolyl)amino)ethyl)benzamide (4h),** 46.7 mg, yield: 72%. White solid, mp: 79-82°C.

¹HNMR (300 MHz, $CDCl_3$): δ 7.31 (d, *J* = 6.9 Hz, 2H), 7.22 (d, *J* = 7.2 Hz, 2H), 7.05 (d, *J* = 8.4 Hz, 2H), 6.70 (d, *J* = 8.4 Hz, 2H), 5.60 (s, 1H), 3.59 (t, *J* = 7.5 Hz, 2H), 2.96 (t, *J* = 7.8 Hz, 2H), 2.89 (s, 3H), 2.25 (s, 3H), 1.46 (s, 9H).

¹³CNMR (75 MHz, $CDCl_3$): δ 169.66, 146.91, 138.15, 137.78, 130.87, 129.75, 129.63, 126.60, 126.14, 125.19, 112.45, 54.93, 51.81, 38.37, 30.50, 28.84, 20.22.

HRMS (ESI): $C_{21}H_{28}N_2O$+Na^+ Calcd: 347.2094, Found: 347.2085.

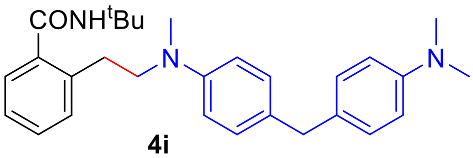

4i

***N*-(tert-butyl)-2-(2-((4-(4-(dimethylamino)benzyl)phenyl)(methyl)amino)ethyl)benzamide (4i),** 63.0 mg, yield: 71%. Yellow oil.

^{1}HNMR (300 MHz, $CDCl_3$): δ 7.30 (d, *J* = 6.9 Hz, 2H), 7.21 (d, *J* = 7.2 Hz, 2H), 7.03-7.08 (m, 4H), 6.69 (d, *J* = 8.4 Hz, 4H), 5.59 (s, 1H), 3.80 (s, 2H), 3.59 (t, *J* = 7.5 Hz, 2H), 2.96 (t, *J* = 7.8 Hz, 2H), 2.90 (s, 6H), 2.88 (s, 3H), 1.45 (s, 9H).
^{13}CNMR (75 MHz, $CDCl_3$): δ 169.65, 149.06, 147.21, 138.15, 137.77, 130.85, 130.50, 129.62, 129.57, 129.52, 129.41, 126.59, 126.13, 113.07, 112.33, 54.85, 51.81, 40.96, 39.87, 38.32, 30.62, 28.84.
HRMS (ESI): $C_{29}H_{37}N_3O+Na^+$ Calcd: 466.2829, Found: 466.2825.

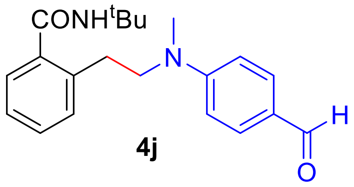

4j

***N*-(tert-butyl)-2-(2-((4-formylphenyl)(methyl)amino)ethyl)benzamide (4j),** 44.7 mg, yield: 66%. White solid, mp: 99-102°C.
^{1}HNMR (300 MHz, $CDCl_3$): δ 9.64 (s, 1H), 7.65 (d, *J* = 8.7 Hz, 2H), 7.25 (t, *J* = 7.2 Hz, 2H), 7.10-7.16 (m, 2H), 6.72 (d, *J* = 9 Hz, 2H), 5.59 (s, 1H), 3.68 (t, *J* = 7.5 Hz, 2H), 2.89-2.95 (m, 5H), 1.41 (s, 9H).
^{13}CNMR (75 MHz, $CDCl_3$): δ 189.20, 168.53, 152.34, 136.98, 135.85, 131.13, 129.96, 128.83, 125.63, 125.59, 123.98, 109.97, 53.21, 50.90, 37.44, 30.17, 27.82.
HRMS (ESI): $C_{21}H_{26}N_2O_2+Na^+$ Calcd: 361.1886, Found: 361.1883.

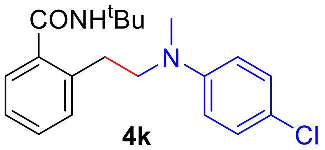

4k

***N*-(tert-butyl)-2-(2-((4-chlorophenyl)(methyl)amino)ethyl)benzamide (4k),** 50.4 mg, yield: 73%. White solid, mp: 96-99°C.
^{1}HNMR (300 MHz, CDCl3): δ 7.30-7.33 (m, 2H), 7.14-7.24 (m, 4H), 6.68 (d, *J* = 8.7 Hz, 2H), 5.60 (s, 1H), 3.61 (t, *J* = 7.5 Hz, 2H), 2.95 (t, *J* = 7.8 Hz, 2H), 2.89 (s, 3H), 1.47 (s, 9H).
^{13}CNMR (75 MHz, $CDCl_3$): δ 169.61, 147.48, 138.08, 137.44, 130.91, 129.71, 128.92, 126.58, 126.31, 120.66, 113.16, 54.66, 51.85, 38.35, 30.62, 28.84.
HRMS (ESI): $C_{20}H_{25}ClN_2O+Na^+$ Calcd: 367.1548, Found: 367.1538.

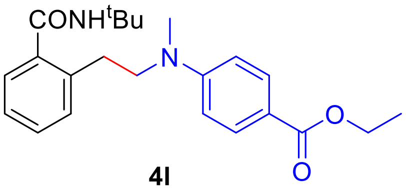

4l

ethyl 4-((2-(tert-butylcarbamoyl)phenethyl)(methyl)amino)benzoate (41), 54.3 mg, yield: 71%. White solid, mp: 96-99°C.

^{1}HNMR (300 MHz, CDCl3): δ 7.90 (d, *J* = 8.7 Hz, 2H), 7.16-7.34 (m, 4H), 6.71 (d, *J* = 9.0 Hz, 2H), 5.64(s, 1H),4.31 (q, *J* = 7.2 Hz, 2H), 3.71 (t, *J* = 7.5 Hz, 2H), 2.95-3.01 (m, 5H), 1.48 (s, 9H), 1.36 (t, *J* = 7.2 Hz, 3H).
^{13}CNMR (75 MHz, $CDCl_3$): δ 169.58, 167.06, 152.04, 138.03, 137.16, 131.36, 130.97, 129.78, 126.62, 126.46, 117.01, 110.54, 60.04, 54.15, 51.88, 38.32, 31.00, 28.84, 14.05.
HRMS (ESI): $C_{23}H_{30}N_2O_3$+Na^+ Calcd: 405.2149, Found: 405.2142.

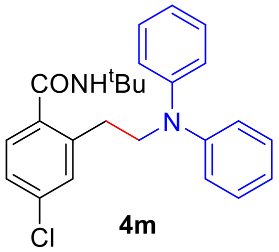

4m

***N*-(tert-butyl)-4-chloro-2-(2-(diphenylamino)ethyl)benzamide (4m),** 56.2 mg, yield: 69%. White solid, mp: 120-121°C.
^{1}HNMR (300 MHz, $CDCl_3$): δ 7.14-7.29 (m, 7H), 7.01 (d, *J* = 7.8 Hz, 4H), 6.49 (t, *J* = 7.2 Hz, 2H), 5.48 (s, 1H), 3.99 (t, *J* = 7.5 Hz, 2H), 3.08 (t, *J* = 7.8 Hz, 2H), 1.41 (s, 9H).
^{13}CNMR (75 MHz, $CDCl_3$): δ 168.64, 147.67, 139.53, 136.51, 135.31, 130.89, 129.33, 127.91, 126.39, 121.28, 120.96, 53.55, 51.99, 31.50, 28.77.
HRMS (ESI): $C_{25}H_{27}ClN_2O$+Na^+ Calcd: 429.1704, Found: 429.1705.

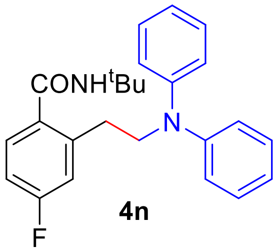

4n

***N*-(tert-butyl)-2-(2-(diphenylamino)ethyl)-4-fluorobenzamide (4n),** 53.9 mg, yield: 69%. White solid, mp: 144-147°C.
^{1}HNMR (300 MHz, $CDCl_3$): δ 7.23-7.28 (m, 5H), 6.84-7.03 (m, 8H), 5.46 (s, 1H), 4.01 (t, *J* = 7.5 Hz, 2H), 3.10 (t, *J* = 7.8 Hz, 2H), 1.42 (s, 9H).
^{19}FNMR (282 MHz) δ: -111.21.
^{13}CNMR (75 MHz, $CDCl_3$): δ 168.76, 164.69, 161.38, 147.68, 140.55 (d, *J* = 7.5 Hz), 134.31, 129.32, 128.50 (d, *J* = 8.3 Hz), 121.10 (d, *J* = 24.0 Hz), 117.70 (d, *J* = 21.0 Hz), 113.09 (d, *J* = 21 Hz), 53.53, 51.90, 31.64, 28.77.
HRMS (ESI): $C_{25}H_{27}FN_2O$+Na^+ Calcd: 413.2000, Found: 413.1997.

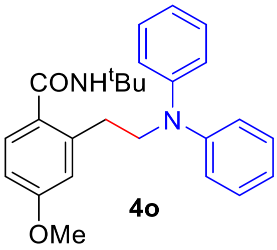

4o

***N*-(tert-butyl)-2-(2-(diphenylamino)ethyl)-4-methoxybenzamide (4o),** 55.5 mg, yield: 69%. White solid, mp: 84-87°C.

^{1}HNMR (300 MHz, $CDCl_3$): δ 7.22-7.28 (m, 5H), 7.02 (d, *J* = 7.8 Hz, 4H), 6.92 (t, *J* = 7.2 Hz, 2H), 6.65-6.72 (m, 2H), 5.48 (s, 1H), 4.01 (t, *J* = 7.5 Hz, 2H), 3.76 (s, 3H), 3.12 (t, *J* = 7.8 Hz, 2H), 1.41 (s, 9H).

^{13}CNMR (75 MHz, $CDCl_3$): δ 169.42, 160.33, 147.79, 139.74, 130.76, 129.23, 128.19, 121.07, 120.95, 116.64, 111.07, 55.30, 53.68, 51.63, 31.80, 28.81.

HRMS (ESI): $C_{26}H_{30}N_2O_2+Na^+$ Calcd: 425.2199, Found: 425.2195.

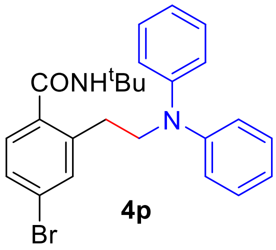

4p

4-bromo-*N*-(tert-butyl)-2-(2-(diphenylamino)ethyl)benzamide (4p), 65.9 mg, yield: 73%. White solid, mp: 170-173°C.

^{1}HNMR (300 MHz, $CDCl_3$): δ 7.16-7.33 (m, 7H), 7.02 (d, *J* = 7.8 Hz, 4H), 6.93 (t, *J* = 7.2 Hz, 2H), 5.48 (s, 1H), 4.01 (t, *J* = 7.8 Hz, 2H), 3.11 (t, *J* = 7.8 Hz, 2H), 1.42 (s, 9H).

^{13}CNMR (75 MHz, $CDCl_3$): δ 169.64, 147.78, 138.20, 137.71, 130.94, 129.63, 129.26, 126.53, 126.29, 121.09, 120.94, 53.84, 51.79, 31.53, 28.79.

HRMS (ESI): $C_{25}H_{27}BrN_2O+Na^+$ Calcd: 473.1199, Found: 473.1189.

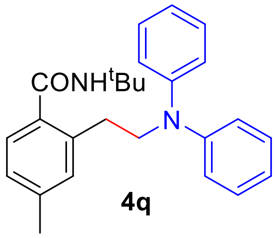

4q

***N*-(tert-butyl)-2-(2-(diphenylamino)ethyl)-4-methylbenzamide (4q),** 55.7 mg, yield: 72%. White solid, mp: 119-122°C.

^{1}HNMR (300 MHz, $CDCl_3$):δ 7.23-7.29 (m, 4H), 7.19 (d, *J* = 7.5 Hz, 1H), 7.04 (d, *J* = 8.1 Hz, 4H), 6.90-6.99 (m, 4H), 5.48 (s, 1H), 3.99 (t, *J* = 7.5 Hz, 2H), 3.09 (t, *J* = 8.1 Hz, 2H), 2.31 (s, 3H), 1.41 (s, 9H).

^{13}CNMR (75 MHz, $CDCl_3$): δ 169.74, 147.81, 139.62, 137.31, 135.39, 131.62, 129.24, 126.85, 126.59, 121.05, 120.96, 53.88, 51.69, 31.52, 28.80, 21.20.
HRMS (ESI): $C_{26}H_{30}N_2O+Na^+$ Calcd: 409.2250, Found: 409.2244.

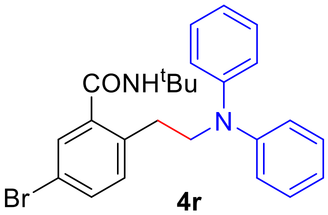

4r

5-bromo-*N*-(tert-butyl)-2-(2-(diphenylamino)ethyl)benzamide (4r), 61.4 mg, yield: 68%. White solid, mp: 184-187°C.
^{1}HNMR (300 MHz, $CDCl_3$): δ 7.40-7.42 (m, 2H), 7.22-7.28 (m, 4H), 6.91-7.06 (m, 7H), 5.46 (s, 1H), 3.98 (t, *J* = 7.5 Hz, 2H), 3.05 (t, *J* = 7.8 Hz, 2H), 1.42 (s, 9H).
^{13}CNMR (75 MHz, $CDCl_3$): δ 167.94, 147.67, 139.84, 136.39, 132.69, 132.54, 129.41, 129.30, 121.24, 120.92, 119.81, 53.53, 52.08, 31.10, 28.72.
HRMS (ESI): $C_{25}H_{27}BrN_2O+Na^+$ Calcd: 473.1199, Found: 473.1191.

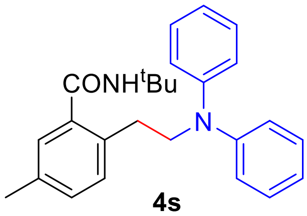

4s

***N*-(tert-butyl)-2-(2-(diphenylamino)ethyl)-5-methylbenzamide (4s),** 56.4 mg, yield: 73%. White solid, mp: 138-141°C.
^{1}HNMR (300 MHz, $CDCl_3$): δ 7.22-7.28 (m, 4H), 7.01-7.12 (m, 7H), 6.93 (t, *J* = 7.2 Hz, 2H), 5.48 (s, 1H), 3.97 (t, *J* = 7.8 Hz, 2H), 3.06 (t, *J* = 7.8 Hz, 2H), 2.32 (s, 3H), 1.42 (s, 9H).
^{13}CNMR (75 MHz, $CDCl_3$): δ 169.78, 147.79, 138.12, 135.97, 134.08, 130.79, 130.27, 129.25, 127.14, 121.05, 120.94, 53.94, 51.75, 31.05, 28.80, 20.88.
HRMS (ESI): $C_{26}H_{30}N_2O+Na^+$ Calcd: 409.2250, Found: 409.2245.

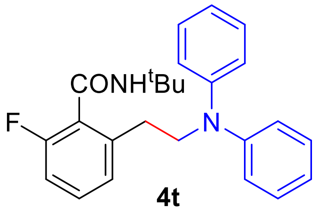

4t

***N*-(tert-butyl)-2-(2-(diphenylamino)ethyl)-6-fluorobenzamide (4t),** 52.3 mg, yield: 67%. White solid, mp: 162-165°C.
^{1}HNMR (300 MHz, $CDCl_3$): δ 7.23-7.29 (m, 5H), 6.90-7.03 (m, 8H), 5.51 (s, 1H), 4.01 (t, *J* = 7.5 Hz, 2H), 3.05 (t, *J* = 7.8 Hz, 2H), 1.42 (s, 9H).
^{19}FNMR (282 MHz): δ -116.87.

^{13}CNMR (75 MHz, CDCl$_3$): δ 164.29, 160.61, 157.36, 147.66, 139.78 (d, *J* = 3.0 Hz), 130.30 (d, *J* = 9.0 Hz), 129.32, 126.33 (d, *J* = 18.0 Hz), 126.08 (d, *J* = 3.0 Hz), 121.10 (d, *J* = 24.1 Hz), 113.64 (d, *J* = 22.5 Hz), 53.66, 52.28, 31.37, 28.77.

HRMS (ESI): $C_{25}H_{27}FN_2O+Na^+$ Calcd: 413.2000, Found: 413.1995.

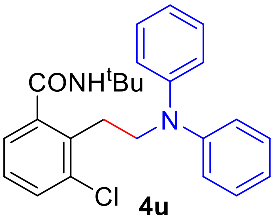

4u

***N*-(tert-butyl)-3-chloro-2-(2-(diphenylamino)ethyl)benzamide (4u),** 51.3 mg, yield: 63%. White solid, mp: 91-94°C.

^{1}HNMR (300 MHz, CDCl$_3$): δ 7.35-7.39 (m, 1H), 7.24-7.30 (m, 4H), 7.13-7.19 (m, 6H), 6.94 (t, *J* = 7.2 Hz, 2H), 5.49 (s, 1H), 4.01 (t, *J* = 8.1 Hz, 2H), 3.20 (t, *J* = 8.4 Hz, 2H), 1.42 (s, 9H).

^{13}CNMR (75 MHz, CDCl$_3$): δ 168.65, 147.71, 140.76, 135.74, 134.57, 130.68, 129.21, 127.46, 124.95, 121.14, 52.08, 51.53, 29.36, 28.80.

HRMS (ESI): $C_{25}H_{27}ClN_2O+Na^+$ Calcd: 429.1704, Found: 429.1703.

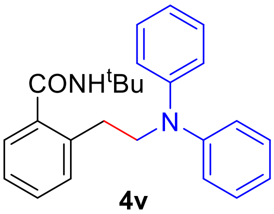

4v

***N*-(tert-butyl)-2-(2-(diphenylamino)ethyl)benzamide (4v),** 56.6 mg, yield: 76%. White solid, mp: 176-178°C.

^{1}HNMR (300 MHz, CDCl$_3$): δ 7.16-7.31 (m, 8H), 7.02 (d, *J* = 7.8 Hz, 4H), 6.93 (t, *J* = 7.2 Hz, 2H), 5.49 (s, 1H), 4.01 (t, *J* = 7.5 Hz, 2H), 3.11 (t, *J* = 7.8 Hz, 2H), 1.42 (s, 9H).

^{13}CNMR (75 MHz, CDCl$_3$): δ 169.64, 147.77, 138.19, 137.31, 130.95, 129.64, 129.27, 126.54, 126.29, 121.09, 120.93, 53.84, 51.80, 31.53, 28.79.

HRMS (ESI): $C_{25}H_{28}N_2O+Na^+$ Calcd: 395.2094, Found: 395.2090.

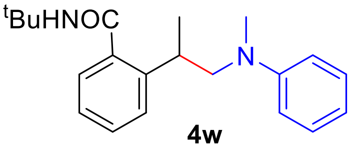

4w

***N*-(tert-butyl)-2-(1-(methyl(phenyl)amino)propan-2-yl)benzamide (4w),** 38.3 mg, yield: 59%. White solid, mp: 78-81°C.

^{1}HNMR (300 MHz, CDCl$_3$): δ 7.40 (s, 2H), 7.16-7.26 (m, 4H), 6.68 (d, *J* = 8.7 Hz, 3H), 5.49 (s, 1H), 3.40-3.67 (m, 3H), 2.79 (s, 3H), 1.28-1.33 (m, 12H).

^{13}CNMR (75 MHz, CDCl$_3$): δ 169.86, 149.46, 142.66, 138.53, 129.57, 129.15, 126.99, 126.75, 126.07, 116.03, 111.95, 60.65, 51.72, 39.11, 34.89, 28.65, 19.34.

HRMS (ESI): $C_{21}H_{28}N_2O+Na^+$ Calcd: 347.2094, Found: 347.2091.

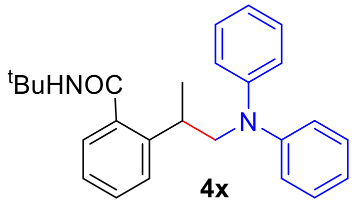

4x

***N*-(tert-butyl)-2-(1-(diphenylamino)propan-2-yl)benzamide (4x),** 49.5 mg, yield: 64%. Yellow oil.

^{1}HNMR (300 MHz, $CDCl_3$): δ 7.18-7.30 (m, 7H), 6.92 (d, J = 6.9 Hz, 7H), 5.42 (s, 1H), 3.98-4.06 (m, 1H), 3.77-3.86 (m, 1H), 3.62 (q, J = 7.2 Hz, 1H), 1.35 (d, J = 6.9 Hz, 3H), 1.32 (s, 9H).

^{13}CNMR (75 MHz, $CDCl_3$): δ 169.74, 148.61, 142.46, 138.54, 129.50, 129.17, 127.16, 126.54, 126.08, 121.23, 59.30, 51.71, 34.98, 28.65, 20.00.

HRMS (ESI): $C_{26}H_{30}N_2O+Na^+$ Calcd: 409.2250, Found: 409.2249.

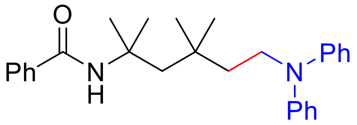

5

***N*-(6-(diphenylamino)-2,4,4-trimethylhexan-2-yl)benzamide (5),** 39.0 mg, yield: 47%. Colorless oil.

^{1}HNMR (300 MHz, $CDCl_3$): δ 7.63 (d, J = 7.2 Hz, 2H), 7.34-7.48 (m, 3H), 7.22 (d, J = 7.8 Hz, 4H), 6.94 (d, J = 8.1 Hz, 6H), 5.85 (s, 1H), 3.77 (t, J = 8.4 Hz, 2H), 1.93 (s, 2H), 1.73 (t, J = 8.4 Hz, 2H), 1.49 (s, 6H), 1.10 (s, 6H).

^{13}CNMR (75 MHz, $CDCl_3$): δ 166.74, 147.88, 136.08, 131.03, 129.25, 128.55, 126.58, 121.02, 120.78, 55.37, 49.63, 48.04, 40.93, 33.86, 29.61, 28.25.

HRMS (ESI): $C_{28}H_{34}N_2O+Na^+$ Calcd: 437.2563, Found: 437.2554.

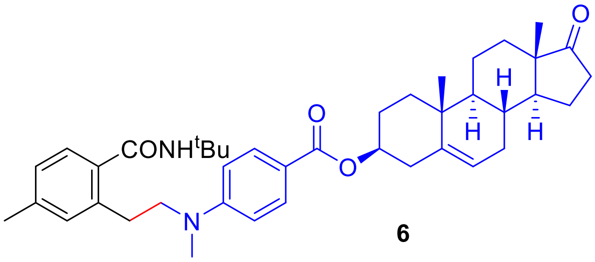

6

(3R,8R,9S,10R,13S,14S)-10,13-dimethyl-17-oxo-2,3,4,7,8,9,10,11,12,13,14,15,16,17-tetradecahydro-1H-cyclopenta[a]phenanthren-3-yl 4-((2-(tert-butylcarbamoyl)-5-methylphenethyl)(methyl)amino)benzoate (6), 71.6 mg, yield: 56%. Yellow oil.

^{1}HNMR (300 MHz, $CDCl_3$): δ 7.87 (d, J = 8.7 Hz, 2H), 7.22 (d, J = 7.5 Hz, 1H), 7.00-7.04 (m, 2H), 6.73 (d, J = 8.7 Hz, 2H), 5.62 (s, 1H), 5.34 (s, 1H), 5.21 (s, 1H), 3.68 (t, J = 7.2 Hz, 2H), 2.92-2.99 (m, 5H), 2.39-2.60 (m, 3H), 2.33 (s, 3H), 1.52-2.15 (m, 12H), 1.46 (s, 9H), 1.15-1.36 (m, 4H), 1.09 (s, 3H), 0.91 (s, 3H).

^{13}CNMR (75 MHz, $CDCl_3$):δ 169.68, 166.37, 152.02, 139.85, 139.12, 137.17, 135.20, 131.68, 131.35, 127.03, 126.68, 121.25, 117.53, 110.58, 69.72, 54.23, 51.86, 51.76, 50.45, 47.57, 38.28, 37.25, 36.72, 35.87, 34.01, 31.50, 30.90, 30.85, 29.70, 28.88, 26.45, 21.88, 21.20, 20.12, 19.02, 13.56.
HRMS (ESI): $C_{41}H_{54}N_2O_4$+Na^+ Calcd: 661.3976, Found: 661.3984.

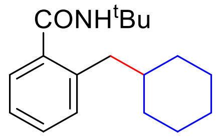

8a

***N*-(tert-butyl)-2-(cyclohexylmethyl)benzamide (8a),** 38.8 mg, yield: 71%. Pale yellow solid, mp: 85-88℃.
^{1}HNMR (300 MHz, $CDCl_3$) δ7.25-7.31 (m, 2H), 7.17 (t, *J*= 6.9 Hz, 2H), 5.55 (s, 1H), 2.68 (d, *J* = 6.8 Hz, 2H), 1.65 (d, *J* = 10.8 Hz, 6H), 1.46 (s, 9H), 1.13-1.61 (m, 3H), 0.80-1.10 (m, 2H).
^{13}CNMR (75 MHz, $CDCl_3$) δ169.92, 138.89, 138.15, 130.91, 128.98, 126.64, 125.63, 51.70, 40.85, 39.59, 33.31, 28.81, 26.53, 26.36.
HRMS (ESI): $C_{18}H_{27}NO$+Na^+ Calcd: 296.1985, Found: 296.198

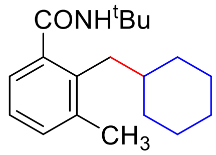

8b

***N*-(tert-butyl)-2-(cyclohexylmethyl)-3-methylbenzamide (8b),** 32.1 mg, yield: 56%. Pale yellow solid, mp: 101-110℃.
^{1}HNMR (300 MHz, $CDCl_3$) δ 7.03-7.17 (m, 3H), 5.52 (s, 1H), 2.75 (d, *J* = 6.9 Hz, 2H), 2.32 (s, 3H), 1.54-1.65 (m, 6H), 1.46 (s, 9H), 0.88-1.06 (m, 5H).
^{13}CNMR (75 MHz, $CDCl_3$) δ 170.57, 138.90, 137.80, 137.49, 131.27, 125.36, 124.53, 51.61, 39.38, 36.52, 33.54, 28.79, 26.56, 26.51, 20.41.
HRMS (ESI): $C_{19}H_{29}NO$+Na^+ Calcd: 310.2141, Found: 310.2148.

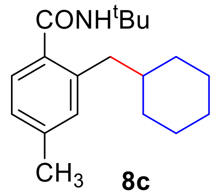

8c

***N*-(tert-butyl)-2-(cyclohexylmethyl)-4-methylbenzamide (8c),** 36.6 mg, yield: 63%. Pale yellow solid, mp: 119-121℃.
^{1}HNMR (300 MHz, $CDCl_3$) δ 7.20 (d, *J* = 8.1 Hz, 1H), 6.97 (d, *J* = 6.9 Hz, 2H), 5.53 (s, 1H), 2.64 (d, *J* = 7.2 Hz, 2H), 2.32 (s, 3H), 1.66 (d, *J* = 9.6 Hz, 6H), 1.45 (s, 9H), 1.13-1.26 (m, 3H), 0.88-0.99 (m, 2H).

^{13}CNMR (75 MHz, $CDCl_3$) δ 170.05, 138.96, 138.85, 135.38, 131.62, 126.69, 126.22, 51.59, 40.81, 39.63, 33.35, 28.82, 26.55, 26.38, 21.30.
HRMS (ESI): $C_{19}H_{29}NO+Na^+$ Calcd: 310.2141, Found: 310.2142.

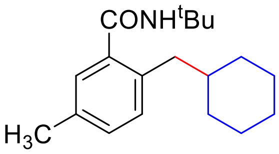

8d

***N*-(tert-butyl)-2-(cyclohexylmethyl)-5-methylbenzamide (8d),** 38.5 mg, yield: 67%. Pale yellow solid, mp: 106-109℃.
^{1}HNMR (300 MHz, $CDCl_3$) δ 7.02-7.11 (m, 3H), 5.52 (s, 1H), 2.62 (d, J = 7.2 Hz, 2H), 2.31 (s, 3H), 1.65 (d, J = 12.6 Hz, 6H), 1.45 (s, 9H), 1.11-1.16 (m, 3H), 0.80-1.05 (m, 2H).
^{13}CNMR (75 MHz, $CDCl_3$) δ 170.05, 138.06, 135.64, 135.21, 130.82, 129.64, 127.27, 51.64, 40.45, 39.63, 33.32, 28.81, 26.55, 26.36, 20.82.
HRMS (ESI): $C_{19}H_{29}NO+Na^+$ Calcd: 310.2141, Found: 310.2140.

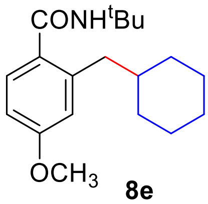

8e

***N*-(tert-butyl)-2-(cyclohexylmethyl)-4-methoxybenzamide (8e),** 33.4 mg, yield: 55%. Pale yellow solid, mp: 76-79℃.
^{1}HNMR (300 MHz, $CDCl_3$) δ 7.27 (s, 1H), 6.69 (t, J = 3 Hz, 2H), 5.52 (s, 1H), 3.80 (s, 3H), 2.67 (d, J = 6.9 Hz, 2H), 1.66 (d, J = 11.4 Hz, 6H), 1.45 (s, 9H), 1.08-1.30 (m, 3H), 0.81-1.06 (m, 2H).
^{13}CNMR (75 MHz, $CDCl_3$) δ 169.74, 159.87, 141.28, 130.91, 128.27, 116.52, 110.41, 55.24, 51.55, 41.08, 39.57, 33.30, 28.82, 26.52, 26.36.
HRMS (ESI): $C_{19}H_{29}NO_2+Na^+$ Calcd: 326.2091, Found: 326.2095.

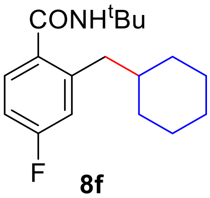

8f

***N*-(tert-butyl)-2-(cyclohexylmethyl)-4-fluorobenzamide (8f),** 42.5 mg, yield: 73%. Pale yellow solid, mp: 85-88℃.
^{1}HNMR (300 MHz, $CDCl_3$) δ 7.25-7.31 (m, 1H), 6.86 (d, J = 9.3 Hz, 2H), 5.53 (s, 1H), 2.66 (d, J = 6.9 Hz, 2H), 1.67-1.71 (m, 6H), 1.45 (s, 9H), 1.13-1.26 (m, 3H), 0.88-0.99 (m, 2H).
^{19}FNMR (282 MHz) δ -112.10.
^{3}CNMR (75 MHz, $CDCl_3$) δ 169.06, 164.37,161.08, 142.18 (d, J = 7.5 Hz), 134.32 (d, J = 3.0 Hz),

128.55 (d, *J* = 8.3 Hz), 117.44 (d, *J* = 21.0 Hz), 112.44 (d, *J* = 21.8 Hz), 51.79, 40.80, 39.44, 33.22, 28.78, 26.43, 26.28.

HRMS (ESI): $C_{18}H_{26}FNO+Na^+$ Calcd: 314.1891, Found: 314.1887.

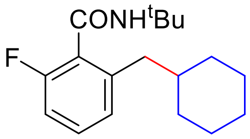

8g

***N*-(tert-butyl)-2-(cyclohexylmethyl)-6-fluorobenzamide (8g),** 40.2 mg, yield: 69%. Pale yellow solid, mp: 70-73°C.

^{1}HNMR (300 MHz, $CDCl_3$) δ 7.17-7.23 (m, 1H), 6.86-6.95 (m, 2H), 5.55 (s, 1H), 2.59 (d, J = 6.9 Hz, 2H), 1.60-1.75 (m, 6H), 1.47 (s, 9H), 1.14-1.26 (m, 3H), 0.83-0.93 (m, 2H).

^{19}FNMR (282 MHz) δ -117.37.

^{3}CNMR (75 MHz, $CDCl_3$) δ 164.48, 141.46, 129.62, 129.51, 126.03, 113.04, 112.75, 52.12, 40.66, 39.33, 33.28, 28.78, 26.49, 26.30.

HRMS (ESI): $C_{18}H_{26}FNO+Na^+$ Calcd: 314.1891, Found: 314.1896.

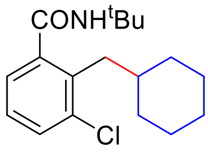

8h

***N*-(tert-butyl)-3-chloro-2-(cyclohexylmethyl)benzamide (8h),** 39.9 mg, yield: 65%. Pale yellow solid, mp: 115-118°C.

^{1}HNMR (300 MHz, $CDCl_3$) δ7.34-7.38 (m, 1H), 7.07-7.20(m, 2H), 5.53 (s, 1H), 2.87(d, *J* = 6.9 Hz, 2H), 1.61-1.67 (m, 6H), 1.46(s, 9H), 1.01-1.15(m, 5H).

^{13}CNMR (75 MHz, $CDCl_3$) δ168.94, 140.33, 137.08, 135.79, 130.53, 126.71, 125.21, 51.93, 38.81, 37.05, 33.30, 28.75, 26.45.

HRMS (ESI): $C_{18}H_{26}ClNO+Na^+$ Calcd: 330.1595, Found: 330.1598.

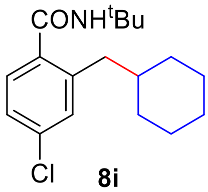

8i

***N*-(tert-butyl)-4-chloro-2-(cyclohexylmethyl)benzamide (8i),** 40.2 mg, yield: 67%. Pale yellow solid, mp: 141-144°C.

^{1}HNMR (300 MHz, $CDCl_3$) δ 7.22(d, *J* = 8.7 Hz, 1H), 7.14 (s, 2H), 5.54 (s, 1H), 2.64 (d, *J* = 7.2 Hz, 2H), 1.60-1.70 (m, 6H), 1.45 (s, 9H), 1.10-1.21 (m, 3H), 0.92-1.17 (m, 2H).

^{13}CNMR (75 MHz, $CDCl_3$) δ 168.91, 141.19, 136.49, 134.74, 130.71, 128.03, 125.75, 51.88, 40.65,

39.47, 33.23, 28.77, 26.43, 26.28.
HRMS (ESI): $C_{18}H_{26}ClNO+Na^+$ Calcd: 330.1595, Found: 330.1602.

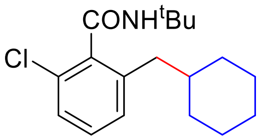

8j

***N*-(tert-butyl)-2-chloro-6-(cyclohexylmethyl)benzamide (8j),** 36.3 mg, yield: 59%. Pale yellow solid, mp: 99-102℃.
^{1}HNMR (300 MHz, $CDCl_3$) δ 7.15-7.20 (m, 2H), 7.04-7.08 (m, 1H), 5.46 (s, 1H), 2.54 (*d*, J = 6.9 Hz, 2H), 1.60-1.75 (m, 6H), 1.48 (s, 9H), 1.07-1.31 (m, 3H), 0.88-0.99 (m, 2H).
^{13}CNMR (75 MHz, $CDCl_3$) δ 166.24, 140.37, 137.58, 130.62, 129.11, 128.58, 126.81, 52.16, 41.09, 39.34, 33.30, 28.73, 26.47, 26.28.
HRMS (ESI): $C_{18}H_{26}ClNO+Na^+$ Calcd: 330.1595, Found: 330.1599.

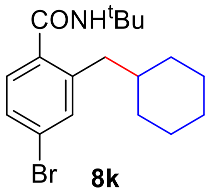

8k

4-bromo-*N*-(tert-butyl)-2-(cyclohexylmethyl)benzamide (8k), 49.3 mg, yield: 70%. Pale yellow solid, mp: 130-133℃.
^{1}HNMR (300 MHz, $CDCl_3$) δ 7.26-7.36 (m, 2H), 7.16 (d, *J* = 8.7 Hz, 1H), 5.51 (s, 1H), 2.63 (d, *J* = 6.9Hz, 2H), 1.52-1.67 (m, 6H), 1.45 (s, 9H), 1.13-1.35 (m, 3H), 0.89-0.99 (m, 2H).
^{13}CNMR (75 MHz, $CDCl_3$) δ 168.92, 141.41, 136.95, 133.63, 128.71, 128.21, 123.12, 51.89, 40.61, 39.50, 33.23, 28.77, 26.43, 26.28.
HRMS (ESI): $C_{18}H_{26}BrNO+Na^+$ Calcd: 374.1090, Found: 374.1092.

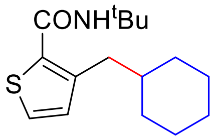

8l

***N*-(tert-butyl)-3-(cyclohexylmethyl)thiophene-2-carboxamide (8l),** 40.2 mg, yield: 72%. Pale yellow solid, mp: 94-97℃.
^{1}HNMR (300 MHz, $CDCl_3$) δ 7.04 (s, 2H), 5.59 (s, 1H), 2.96 (d, *J* = 7.2 Hz, 2H), 1.55-1.76 (m, 6H), 1.44 (s, 9H), 1.11-1.28 (m, 3H), 0.93-1.05 (m, 2H).
^{13}CNMR (75, MHz, $CDCl_3$) δ 164.61, 146.90, 134.41, 126.53, 122.14, 51.52, 40.52, 36.05, 33.05, 28.93, 26.36, 26.19.
HRMS (ESI): $C_{16}H_{25}NOS+Na^+$ Calcd: 302.1549, Found: 302.1550.

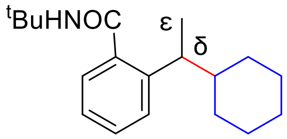

8m

***N*-(tert-butyl)-2-(1-cyclohexylethyl)benzamide (8m, δ : ε = 3 : 1),** 25.8 mg, yield: 45%. Pale yellow solid, mp: 78-81°C.

^{1}HNMR (300 MHz, $CDCl_3$) δ 7.26-7.36 (m, 3H), 7.12-7.24 (m, 1H), 5.53 (s, 1H), 2.74-2.93 (m, 1H), 1.60-1.70 (m, 4H), 1.46 (s, 9H), 1.06-1.39 (m, 8H), 0.82-0.99 (m, 2H).

^{13}CNMR (75 MHz, $CDCl_3$) δ 170.15, 144.90, 138.30, 129.30, 126.91, 126.17, 125.30, 51.77, 43.83, 41.00, 32.01, 30.49, 28.86, 26.49, 19.46.

HRMS (ESI): $C_{19}H_{29}NO+Na^+$ Calcd: 310.2141, Found: 310.2145.

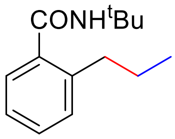

9a

***N*-(tert-butyl)-2-propylbenzamide (9a),** 28.5 mg, yield: 65%. Pale yellow solid, mp: 84-87°C.

^{1}HNMR (300 MHz, $CDCl_3$) δ 7.26-7.33 (m, 2H), 7.15-7.23 (m, 2H), 5.55 (s, 1H), 2.74 (t, J = 7.8 Hz, 2H), 1.60-1.69 (m, 2H), 1.46 (s, 9H), 0.96 (t, J = 7.2 Hz, 3H).

^{13}CNMR (75 MHz, $CDCl_3$) δ 169.85, 140.21, 137.89, 130.01, 129.31, 126.57, 125.66, 51.73, 35.23, 28.81, 24.84, 14.17.

HRMS (ESI): $C_{14}H_{21}NO+Na^+$ Calcd: 242.1515, Found: 242.1517.

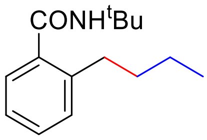

9b

***N*-(tert-butyl)-2-butylbenzamide (9b),** 29.4 mg, yield: 63%. Yellow solid, mp: 83-85°C.

^{1}HNMR (300 MHz, $CDCl_3$) δ 7.26-7.33 (m, 2H), 7.14-7.23 (m, 2H), 5.55 (s, 1H), 2.76 (t, J = 7.8 Hz, 2H), 1.54-1.65 (m, 2H), 1.46 (s, 9H), 1.31-1.42 (m, 2H), 0.95 (t, J = 7.2 Hz, 3H).

^{13}CNMR (75 MHz, $CDCl_3$) δ 169.84, 140.44, 137.82, 129.97, 129.33, 126.58, 125.61, 51.71, 33.91, 32.95, 28.81, 22.79, 13.96.

HRMS (ESI): $C_{15}H_{23}NO+Na^+$ Calcd: 256.1672, Found: 256.1677.

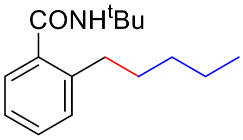

9c

***N*-(tert-butyl)-2-pentylbenzamide (9c),** 32.6 mg, yield: 66%. Yellow solid, mp: 73-76°C.

^{1}**HNMR** (300 MHz, $CDCl_3$) δ 7.26-7.33 (m, 2H), 7.14-7.23 (m, 2H), 5.55 (s, 1H), 2.76 (t, *J* = 7.5 Hz, 2H), 1.58-1.64 (m, 2H), 1.46 (s, 9H), 1.33-1.35 (m, 4H), 0.89 (t, *J* = 6.9 Hz, 3H).
^{13}**CNMR** (75 MHz, $CDCl_3$) δ 169.84, 140.49, 137.80, 129.97, 129.34, 126.58, 125.62, 51.72, 33.20, 31.93, 31.46, 28.81, 22.55, 14.05.
HRMS (ESI): $C_{16}H_{25}NO+Na^+$ Calcd: 270.1828, Found: 270.1831.

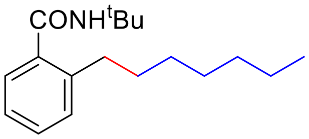

9d

***N*-(tert-butyl)-2-heptylbenzamide (9d),** 35.8 mg, yield: 65%. Yellow solid, mp: 39-42℃.
^{1}**HNMR** (300 MHz, $CDCl_3$) δ 7.26-7.32 (m, 2H), 7.17-7.23 (m, 2H), 5.55 (s, 1H), 2.76 (t, *J* = 2.76 Hz, 2H), 1.61 (s, 2H), 1.46 (s, 9H), 1.18-1.40 (m, 8H), 0.85 (t, *J* = 6.9 Hz, 3H).
^{13}**CNMR** (75 MHz, $CDCl_3$) δ 169.84, 140.48, 137.81, 129.96, 129.33, 126.58, 125.61, 51.72, 33.26, 31.83, 31.79, 29.72, 29.19, 28.81, 22.66, 14.09.
HRMS (ESI): $C_{18}H_{29}NO+Na^+$ Calcd: 298.2141, Found: 298.2146.

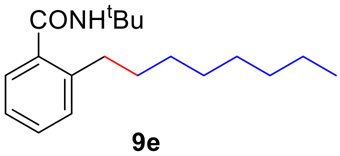

9e

***N*-(tert-butyl)-2-octylbenzamide (9e),** 39.9 mg, yield: 69%. Yellow oil.
^{1}**HNMR** (300 MHz, $CDCl_3$) δ 7.26-7.32 (m, 2H), 7.14-7.22 (m, 2H), 5.54 (s, 1H), 2.75 (t, *J* = 7.5 Hz, 2H), 1.56-1.59 (m, 2H), 1.46 (s, 9H), 1.28 (s, 10H), 0.87 (t, *J* = 6.0 Hz, 3H).
^{13}**CNMR** (75 MHz, $CDCl_3$) δ 169.84, 140.49, 137.80, 129.97, 129.33, 126.58, 125.61, 51.72, 33.27, 31.88, 31.79, 29.77, 29.49, 29.28, 28.82, 22.67, 14.11.
HRMS (ESI): $C_{19}H_{23}NO+Na^+$ Calcd: 312.2298, Found: 312.2298.

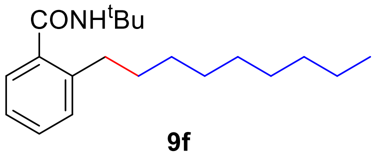

9f

***N*-(tert-butyl)-2-nonylbenzamide (9f),** 38.8 mg, yield: 64%. Yellow solid, mp: 35-38℃.
^{1}**HNMR** (300 MHz, $CDCl_3$) δ 7.26-7.32 (m, 2H), 7.14-7.22 (m, 2H), 5.55 (s, 1H), 2.75 (t, *J* = 8.1 Hz, 2H), 1.57-1.63 (m, 2H), 1.46 (s, 9H), 1.21-1.31 (m, 12H), 0.87 (t, *J* = 6 Hz, 3H).
^{13}**CNMR** (75 MHz, $CDCl_3$) δ 169.84, 140.49, 137.80, 129.96, 129.33, 126.58, 125.61, 51.72, 33.26, 31.89, 31.79, 29.76, 29.57, 29.53, 29.32, 28.82, 22.67, 14.11.
HRMS (ESI): $C_{20}H_{33}NO+Na^+$ Calcd: 326.2454, Found: 326.2452.

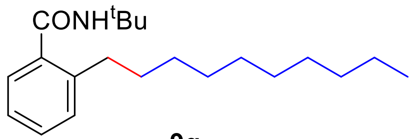

9g

***N*-(tert-butyl)-2-decylbenzamide (9g),** 38.7 mg, yield: 61%. Yellow solid, mp: 46-49°C.

^{1}HNMR (300 MHz, $CDCl_3$) δ 7.26-7.32 (m, 2H), 7.14-7.22 (m, 2H), 5.54 (s, 1H), 2.75 (t, *J* = 7.8 Hz, 2H), 1.57-1.63 (m, 2H), 1.46 (s, 9H), 1.28(s, 14H), 0.88 (t, *J* = 6.0 Hz, 3H).

^{13}CNMR (75 MHz, $CDCl_3$) δ 169.83, 140.49, 137.81, 129.96, 129.33, 126.58, 125.61, 51.72, 33.26, 31.90, 31.79, 29.77, 29.62, 29.53, 29.33, 28.82, 22.68, 14.12.

HRMS (ESI): $C_{21}H_{35}NO+Na^+$ Calcd: 340.2611, Found: 340.2610.

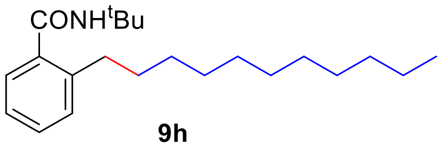

9h

***N*-(tert-butyl)-2-undecylbenzamide (9h),** 39.1 mg, yield: 59%. Yellow solid, mp: 47–50°C.

^{1}HNMR (300 MHz, $CDCl_3$) δ 7.26-7.32 (m, 2H), 7.14-7.22 (m, 2H), 5.55 (s, 1H), 2.75 (t, *J* =7.8 Hz, 2H), 1.56-1.65 (m, 2H), 1.46 (s, 9H), 1.28(s, 16H), 0.88 (t, *J* = 6.0 Hz, 3H).

^{13}CNMR (75 MHz, $CDCl_3$) δ 169.84, 140.49, 137.80, 129.96, 129.33, 126.57, 125.60, 51.72, 33.26, 31.92, 31.80, 29.77, 29.67, 29.62, 29.53, 29.35, 28.82, 22.69, 14.13.

HRMS (ESI): $C_{22}H_{37}NO+Na^+$ Calcd: 354.2767, Found: 354.2764.

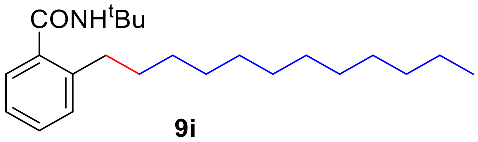

9i

***N*-(tert-butyl)-2-dodecylbenzamide (9i),** 44.9 mg, yield: 65%. Yellow solid, mp: 40-43°C.

^{1}HNMR (300 MHz, $CDCl_3$) δ7.26-7.32 (m, 2H), 7.14-7.22 (m, 2H), 5.54 (s, 1H), 2.75 (t, J = 7.8 Hz, 2H), 1.58 (s, 2H), 1.46 (s, 9H), 1.25-1.32 (m, 18H), 0.87 (t, *J* = 6.0 Hz, 3H).

^{13}CNMR (75 MHz, $CDCl_3$) δ 169.83, 140.49, 137.81, 129.96, 129.33, 126.57, 125.60, 53.42, 51.72, 33.26, 31.92, 31.79, 29.77, 29.67, 29.62, 29.53, 29.36, 28.82, 22.69, 14.12.

HRMS (ESI): $C_{23}H_{39}NO+Na^+$ Calcd: 368.2924, Found: 368.2926.

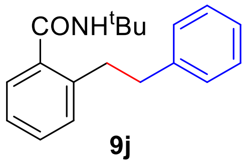

9j

***N*-(tert-butyl)-2-phenethylbenzamide (9j),** 40.5 mg, yield: 72%. Pale yellow solid, mp: 97-100°C.

^{1}HNMR (300 MHz, $CDCl_3$) δ 7.16-7.32 (m, 9H), 5.48(s, 1H), 3.07 (t, *J* = 7.2 Hz, 2H), 2.92 (t, *J* = 8.7 Hz, 2H), 1.45 (s, 9H).

^{13}CNMR (75 MHz, $CDCl_3$) δ 169.72, 141.81, 139.44, 137.95, 130.21, 129.45, 128.55, 128.31, 126.58, 125.96, 125.88, 51.70, 38.08, 35.47, 28.78.

HRMS (ESI): $C_{19}H_{23}NO+Na^+$ Calcd: 304.1672, Found: 304.1677.

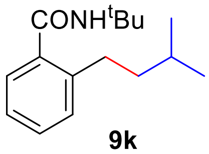

9k

***N*-(tert-butyl)-2-isopentylbenzamide (9k),** 33.6 mg, yield: 68%. Pale yellow solid, mp: 84-87°C.
^{1}HNMR (300 MHz, $CDCl_3$) δ7.26-7.32 (m, 2H), 7.19 (t, *J* = 8.4 Hz, 2H), 5.55 (s, 1H), 2.73-2.79 (m, 2H), 1.59 (s, 1H), 1.46 (s, 9H), 1.27 (s, 2H), 0.93 (d, *J* = 6.6 Hz, 6H).
^{13}CNMR (75 MHz, $CDCl_3$) δ 169.81, 140.66, 137.82, 129.92, 129.36, 126.59, 125.58, 51.72, 41.07, 31.18, 28.82, 28.27, 22.52, 14.11.
HRMS (ESI): $C_{16}H_{25}NO+Na^+$ Calcd: 270.1828, Found: 270.1827.

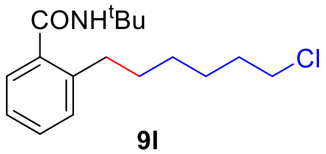

9l

***N*-(tert-butyl)-2-(6-chlorohexyl)benzamide (9l),** 27.8 mg, yield: 47%. Yellow oil.
^{1}HNMR (300 MHz, $CDCl_3$) δ 7.26-7.33 (m, 2H), 7.15-7.22 (m, 2H), 5.55 (s, 1H), 3.52 (t, *J* = 6.6 Hz, 2H), 2.77 (t, *J* = 7.8 Hz, 2H), 1.73-1.81 (m, 2H), 1.64-1.69 (m, 2H), 1.57-1.63 (m, 13H).
^{13}CNMR (75 MHz, $CDCl_3$) δ 169.77, 140.28, 137.76, 129.97, 129.39, 126.59, 125.72, 51.73, 45.09, 33.12, 32.53, 31.47, 28.88, 28.82, 26.71.
HRMS (ESI): $C_{17}H_{26}ClNO+Na^+$ Calcd: 318.1595, Found: 318.1598.

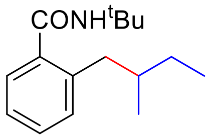

10a

***N*-(tert-butyl)-2-(2-methylbutyl)benzamide (10a),** 34.6 mg, yield: 70%. Yellow solid, mp: 60-63°C.
^{1}HNMR (300 MHz, $CDCl_3$) δ 7.29 (d, *J* = 7.8 Hz, 2H),7.10-7.19 (m, 2H), 5.55 (s, 1H), 2.83-2.90 (m, 1H), 2.38-2.60 (m, 1H), 1.55-1.74(m, 1H), 1.42 (s, 9H), 1.35-1.40 (m, 1H),1.12-1.25 (m, 1H), 0.81-0.92 (m, 6H).
^{13}CNMR (75 MHz, $CDCl_3$) δ 169.91, 139.35, 138.20, 130.88, 129.04, 126.65, 125.62, 51.70, 40.24, 36.45, 29.54, 28.89, 28.81, 18.96, 11.51.
HRMS (ESI): $C_{16}H_{25}NO+Na^+$ Calcd: 270.1828, Found: 270.1831.

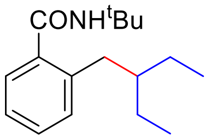

10b

***N*-(tert-butyl)-2-(2-ethylbutyl)benzamide (10b),** 39.2 mg, yield: 75%. Pale yellow solid, mp: 60-63°C.

^{1}HNMR (300 MHz, $CDCl_3$) δ 7.26-7.29 (m, 2H), 7.16 (d, *J* = 7.5 Hz, 2H), 5.55 (s, 1H), 2.73 (d, *J* = 7.2 Hz, 2H), 1.51-1.62 (m, 1H), 1.45(s, 9H), 1.24-1.34 (m, 4H), 0.84 (t, *J* = 8.4 Hz, 6H).

^{13}CNMR (75 MHz, $CDCl_3$) δ 169.90, 139.62, 138.26, 130.86, 129.09, 126.69, 125.54, 51.69, 41.96, 36.95, 28.82, 25.00, 10.72.

HRMS (ESI): $C_{17}H_{27}NO+Na^+$ Calcd: 284.1985, Found: 284.1986.

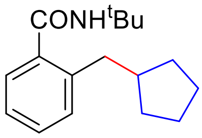

10c

***N*-(tert-butyl)-2-(cyclopentylmethyl)benzamide (10c),** 34.7 mg, yield: 67%. Pale yellow solid, mp: 63-66°C.

^{1}HNMR (300 MHz, $CDCl_3$) δ 7.26-7.30 (m, 2H), 7.14-7.23 (m, 2H), 5.56 (s, 1H), 2.79 (d, *J* = 7.5 Hz, 2H), 2.04-2.20 (m, 1H), 1.61-1.74 (m, 6H), 1.56 (s, 9H), 1.15-1.53 (m, 2H).

^{13}CNMR (75 MHz, $CDCl_3$) δ169.93, 139.86, 137.96, 130.38, 129.15, 126.58, 125.59, 51.71, 41.84, 38.80, 32.57, 28.81, 24.86.

HRMS (ESI): $C_{17}H_{25}NO+Na^+$ Calcd: 282.1828, Found: 282.1829.

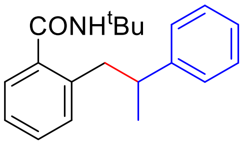

10d

***N*-(tert-butyl)-2-(2-phenylpropyl)benzamide (10d),** 43.7 mg, yield: 74%. Pale yellow solid, mp: 57-60°C.

^{1}HNMR (300 MHz, $CDCl_3$) δ 7.11-7.35 (m, 8H), 7.03 (d, *J* = 7.8 Hz, 1H), 5.46 (s, 1H), 2.97-3.14 (m, 3H), 1.45 (s, 9H), 1.25 (d, *J* = 6.3 Hz, 3H).

^{13}CNMR (75 MHz, $CDCl_3$) δ 169.91, 146.84, 138.51, 138.19, 130.90, 129.08, 128.25, 127.12, 126.60, 125.97, 125.86, 51.71, 42.05, 41.40, 28.81, 21.17.

HRMS (ESI): $C_{20}H_{25}NO+Na^+$ Calcd: 318.1828, Found: 318.1829.

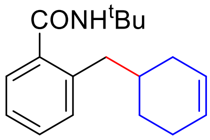

10e

N-(tert-butyl)-2-(cyclohex-3-en-1-ylmethyl)benzamide (10e), 36.9 mg, yield: 68%. Pale yellow solid, mp: 80-83°C.

^{1}HNMR (300 MHz, $CDCl_3$) δ 7.26-7.32 (m, 2H), 7.19 (d, J = 7.2 Hz, 2H), 5.50-5.67 (m, 3H), 2.76 (d, J = 6.9 Hz, 2H), 1.96-2.03 (m, 4H), 1.71-1.76 (m, 2H), 1.45 (s, 9H), 1.20-1.34 (m, 1H).

^{3}CNMR (75 MHz, $CDCl_3$) δ 169.86, 138.67, 138.20, 130.80, 129.08, 126.92, 126.66, 126.34, 125.77, 51.73, 39.81, 35.41, 31.78, 28.79, 28.65, 25.21.

HRMS (ESI): $C_{18}H_{25}NO+Na^+$ Calcd: 294.1828, Found: 294.1829.

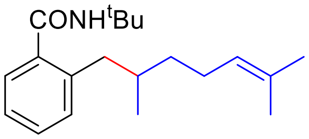

10f

N-(tert-butyl)-2-(2,6-dimethylhept-5-en-1-yl)benzamide (10f), 43.3 mg, yield: 72%. Yellow oil.

^{1}HNMR (300 MHz, $CDCl_3$) δ7.26-7.31 (m, 2H), 7.15-7.20 (m, 2H), 5.54 (s, 1H), 5.07 (t, J = 7.2 Hz, 1H), 2.82-2.89 (m, 1H), 2.52-2.60 (m, 1H), 1.87-2.11 (m, 2H), 1.75-1.83 (m, 1H), 1.71 (s, 3H), 1.67 (s, 3H), 1.45 (s, 9H), 1.24-1.39 (m, 1H), 1.11-1.23 (m, 1H), 0.84 (d, J= 6.6 Hz, 3H).

^{13}CNMR (75 MHz, $CDCl_3$) δ 169.91, 139.23, 138.21, 131.11, 130.86, 129.07, 126.63, 125.64, 124.78, 51.69, 40.54, 37.14, 34.54, 28.80, 25.74, 25.64, 19.33, 17.65.

HRMS (ESI): $C_{20}H_{31}NO+Na^+$ Calcd: 324.2298, Found: 324.2298.

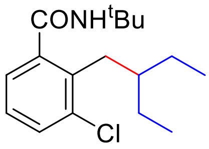

10g

N-(tert-butyl)-3-chloro-2-(2-ethylbutyl)benzamide (10g), 42.0 mg, yield: 71%. Yellow solid, mp: 81-84°C.

^{1}HNMR (300 MHz, $CDCl_3$) δ 7.35-7.38 (m, 1H), 7.19 (d, J = 6.6 Hz, 1H), 7.11 (t, J = 7.5 Hz, 1H), 5.52 (s, 1H), 2.93 (d, J =7.5 Hz, 2H), 1.62-1.69 (m, 1H), 1.45 (s, 9H), 1.17-1.40 (m, 4H), 0.85 (t, J = 7.5 Hz, 6H).

^{13}CNMR (75 MHz, $CDCl_3$) δ 168.92, 140.47, 137.73, 135.91, 130.64, 126.71, 125.33, 51.93, 41.05, 34.08, 28.78, 25.25, 10.96.

HRMS (ESI): $C_{17}H_{26}ClNO+Na^+$ Calcd: 318.1595, Found: 318.1601.

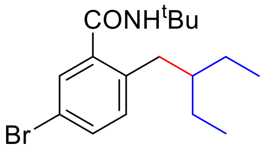

10h

5-bromo-N-(tert-butyl)-2-(2-ethylbutyl)benzamide (10h), 46.9 mg, yield: 69%. Pale yellow solid, mp: 73-76°C.

^{1}HNMR (300 MHz, $CDCl_3$) δ 7.40 (s, 2H), 7.05 (d, J = 7.8 Hz, 1H), 5.54 (s, 1H), 2.66 (d, J = 7.2

Hz, 2H), 1.50-1.69 (m, 1H), 1.45 (s, 9H), 1.22-1.31 (m, 4H), 0.83 (t, *J* = 7.2 Hz, 6H).
^{13}CNMR (75 MHz, $CDCl_3$) δ 168.16, 139.92, 138.71, 132.59, 131.99, 129.55, 119.03, 51.97, 41.90, 36.48, 28.76, 24.97, 10.68.
HRMS (ESI): $C_{17}H_{26}BrNO+Na^+$ Calcd: 362.1090, Found: 362.1096.

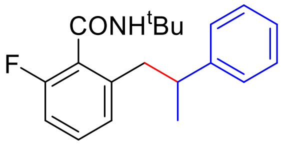

10i

***N*-(tert-butyl)-2-fluoro-6-(2-phenylpropyl)benzamide (10i),** 45.7 mg, yield: 73%. Yellow solid, mp: 93-96°C.
^{1}HNMR (300 MHz, $CDCl_3$) δ 7.11-7.28 (m, 6H), 6.80-6.90 (m, 2H), 5.43 (s, 1H), 3.06-3.17 (m, 1H), 2.88-3.02 (m, 2H), 1.46 (s, 9H), 1.27 (*d*, *J* = 6.9 Hz, 3H).
^{19}FNMR (282 MHz) δ -117.220.
^{13}CNMR (75 MHz, $CDCl_3$) δ 164.43, 160.58, 157.32, 146.44, 140.92 (*d*, *J* = 3 Hz), 129.65 (*d*, *J* = 8.3 Hz), 128.29, 127.07, 126.11, 126.03 (*d*, *J* = 3 Hz), 113.15 (*d*, *J* = 22 Hz), 52.13, 41.91, 41.17, 28.76, 21.13.
HRMS (ESI): $C_{20}H_{24}FNO+Na^+$ Calcd: 336.1734, Found: 336.1741.

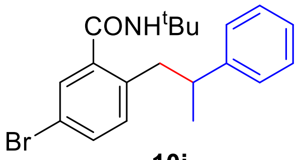

10j

5-bromo-*N*-(tert-butyl)-2-(2-phenylpropyl)benzamide 10j), 48.6 mg, yield: 65%. Pale yellow solid, mp: 90-93°C.
^{1}HNMR (300 MHz, $CDCl_3$) δ 7.10-7.37 (m, 7H), 6.86 (d, *J* = 8.1 Hz, 1H), 5.43 (s, 1H), 2.95-3.07 (m, 3H), 1.44 (s, 9H), 1.25 (d, *J* = 6.6 Hz, 3H).
^{13}CNMR (75 MHz, $CDCl_3$) δ 167.92, 146.06, 139.64, 137.34, 132.34, 131.75, 129.22, 128.10, 126.86, 125.90, 119.15, 51.74, 41.28, 41.07, 28.51, 21.05.
HRMS (ESI): $C_{20}H_{24}BrNO+Na^+$ Calcd: 396.0933, Found: 396.0942.

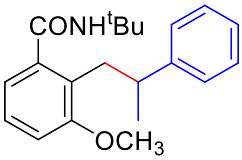

10k

***N*-(tert-butyl)-3-methoxy-2-(2-phenylpropyl)benzamide (10k),** 36.4 mg, yield: 56%. Pale yellow solid, mp: 58-61°C.
^{1}HNMR (300 MHz, $CDCl_3$) δ 7.11-7.23 (m, 6H), 6.84 (d, *J* = 7.8 Hz, 2H), 5.28 (s, 1H), 3.76 (s, 3H), 2.97-3.22 (m, 3H), 1.42 (s, 9H), 1.23 (d, *J* = 6.9 Hz, 3H).
^{13}CNMR (75 MHz, $CDCl_3$) δ 169.70, 158.14, 147.59, 140.02, 128.00, 127.32, 127.16, 126.89, 125.70, 118.79, 111.23, 55.46, 51.61, 39.72, 35.57, 28.74, 20.61.

HRMS (ESI): $C_{21}H_{27}NO_2$+Na^+ Calcd: 348.1934, Found: 348.1942.

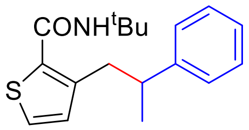

10l

***N*-(tert-butyl)-3-(2-phenylpropyl)thiophene-2-carboxamide (10l),** 44.5 mg, yield: 74%. Pale yellow solid, mp: 86-89°C.

^{1}HNMR (300 MHz, $CDCl_3$) δ 7.17-7.29 (m, 5H), 6.94-7.00 (m, 2H), 5.48 (s, 1H), 3.31-3.45 (m, 2H), 3.07-3.18 (m, 1H), 1.43 (s, 9H), 1.32 (d, *J* = 6.9 Hz, 3H).

^{3}CNMR (75 MHz, $CDCl_3$) δ 164.50, 146.74, 146.07, 134.28, 128.35, 127.13, 126.19, 126.08, 122.50, 51.51, 42.33, 37.01, 28.94, 21.69.

HRMS (ESI): $C_{18}H_{23}NOS$+Na^+ Calcd: 324.1393, Found: 324.1399.

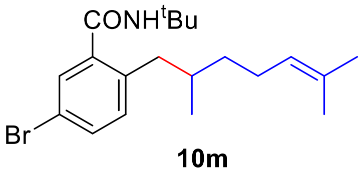

10m

5-bromo-*N*-(tert-butyl)-2-(2,6-dimethylhept-5-en-1-yl)benzamide (10m), 53.3 mg, yield: 70%. Yellow oil liquid.

^{1}HNMR (300 MHz, $CDCl_3$) δ 7.40 (d, *J* = 6.3 Hz, 2H), 7.04 (d, *J* = 8.7 Hz, 1H), 5.51 (s, 1H), 5.05 (t, *J* = 6.0 Hz, 1H), 2.75-2.83 (m, 1H), 2.46-2.54 (m, 1H), 1.88-2.04 (m, 2H), 1.66 (s, 3H), 1.58 (s, 3H), 1.45 (s, 9H), 1.10-1.39 (m, 3H), 0.82 (d, *J* = 6.6 Hz, 3H)

^{13}CNMR (75 MHz, $CDCl_3$) δ 168.16, 139.89, 138.33, 132.58, 132.02, 131.27, 129.50, 124.59, 119.16, 51.98, 40.03, 37.04, 34.43, 28.74, 25.72, 25.58, 19.25, 17.64.

HRMS (ESI): $C_{20}H_{30}BrNO$+Na^+ Calcd: 402.1403, Found: 402.1408.

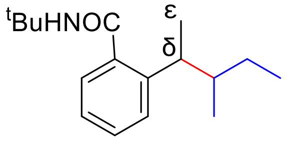

10n

***N*-(tert-butyl)-2-(3-methylpentan-2-yl)benzamide (10n,** δ : ε = 2 : 1**),** 24.5 mg, yield: 47%. Pale yellow solid, mp: 51-54°C.

^{1}HNMR (300 MHz, $CDCl_3$) δ 7.24-7.34 (m, 3H), 7.09-7.18 (m, 1H), 5.50 (s, 1H), 2.55-3.08 (m, 1H), 1.58-1.64 (m, 2H), 1.45 (s, 9H), 1.19-1.27 (m, 4H), 0.68-0.91 (m, 6H).

^{13}CNMR (75 MHz, $CDCl_3$) δ 170.15, 145.00, 138.28, 138.16, 129.29, 127.00, 126.92, 126.34, 126.14, 125.33, 51.74, 40.38, 40.20, 40.14, 28.84, 27.97, 25.85, 19.58, 18.56, 17.60, 15.62, 11.64, 10.73.

HRMS (ESI): $C_{17}H_{27}NO$+Na^+ Calcd: 284.1985, Found: 284.1992.

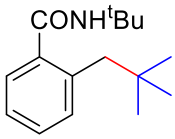

11a

***N*-(tert-butyl)-2-neopentylbenzamide (11a),** 25.7 mg, yield: 52%. Pale yellow solid, mp: 65-68°C.

^{1}HNMR (300 MHz, $CDCl_3$) δ 7.31 (t, *J* = 7.5 Hz, 2H), 7.19 (t, *J* = 6.3 Hz, 2H), 5.57 (s, 1H), 2.86 (s, 2H), 1.45 (s, 9H), 0.90 (s, 9H).

^{13}CNMR (75 MHz, $CDCl_3$) δ 170.20, 138.81, 137.80, 132.61, 128.64, 127.00, 125.75, 51.64, 45.08, 32.55, 29.73, 28.80.

HRMS (ESI): $C_{16}H_{25}NO+Na^+$ Calcd: 270.1828, Found: 270.1827.

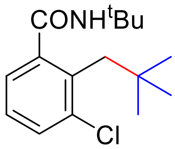

11b

***N*-(tert-butyl)-3-chloro-2-neopentylbenzamide (11b),** 18.2 mg, yield: 43%. Pale yellow solid, mp: 49–50°C.

^{1}HNMR (300 MHz, $CDCl_3$) δ 7.11-7.27 (m, 3H), 5.43 (s, 1H), 2.64 (s, 2H), 1.48 (s, 9H), 0.95 (s, 9H).

^{13}CNMR (75 MHz, $CDCl_3$) δ 166.37, 139.17, 138.32, 130.84, 129.64, 128.67, 127.13, 52.20, 45.90, 32.37, 30.09, 28.67.

HRMS (ESI): $C_{16}H_{24}ClNO+Na^+$ Calcd: 304.1439, Found: 304.1443.

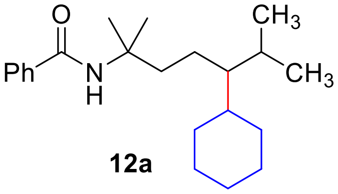

12a

***N*-(5-cyclohexyl-2,6-dimethylheptan-2-yl)benzamide (12a),** 42.2 mg, yield: 64%. Yellow oil.

^{1}HNMR (300 MHz, $CDCl_3$) δ 7.70 (d, *J* = 6.9 Hz, 2H), 7.38-7.48 (m, 3H), 5.83 (s, 1H), 1.56-1.83 (m, 6H), 1.44 (s, 6H), 0.96-1.35 (m, 9H), 0.85-0.89 (m, 8H).

^{13}CNMR (75 MHz, $CDCl_3$) δ 166.84, 136.24, 130.97, 128.49, 126.63, 54.48, 49.77, 41.43, 40.22, 31.79, 30.07, 28.90, 26.89, 22.12, 21.46, 19.19.

HRMS (ESI): $C_{22}H_{35}NO+Na^+$ Calcd: 352.2611, Found: 352.2616.

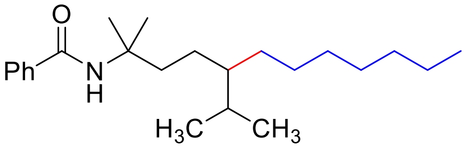

12b

***N*-(5-isopropyl-2-methyldodecan-2-yl)benzamide (12b),** 38.7 mg, yield: 56%. Yellow oil.

^{1}HNMR (300 MHz, $CDCl_3$) δ 7.70 (d, *J* = 6.9 Hz, 2H), 7.38-7.48 (m, 3H), 5.82 (s, 1H), 1.67-1.81

(m, 3H), 1.43 (s, 6H), 0.95-1.25 (m, 14H), 0.80-0.90 (m, 10H).

^{13}CNMR (75 MHz, $CDCl_3$) δ 166.83, 136.19, 130.99, 128.49, 126.63, 54.33, 44.09, 38.39, 31.91, 30.48, 30.08, 29.38, 29.23, 27.83, 26.97, 24.61, 22.68, 19.38, 19.14, 14.11.

HRMS (ESI): $C_{23}H_{39}NO+Na^+$ Calcd: 368.2924, Found: 368.2934.

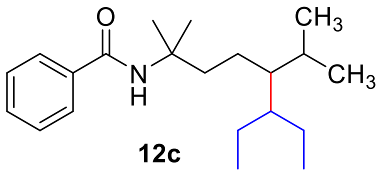

12c

***N*-(6-ethyl-5-isopropyl-2-methyloctan-2-yl)benzamide (12c),** 38.1 mg, yield: 60%. Yellow oil.

^{1}HNMR (300 MHz, $CDCl_3$) δ 7.70 (d, J = 6.6 Hz, 2H), 7.38-7.47 (m, 3H), 5.82 (s, 1H), 1.68-1.85 (m, 3H), 1.38 (s, 6H), 1.05-1.36 (m, 8H), 0.84-0.88 (m, 12H).

^{13}CNMR (75 MHz, $CDCl_3$) δ 166.83, 136.22, 130.97, 128.48, 126.62, 54.44, 45.42, 42.98, 40.93, 29.25, 26.92, 23.98, 23.04, 22.00, 21.23, 20.34, 12.71, 12.21.

HRMS (ESI):$C_{21}H_{35}NO+Na^+$ Calcd: 340.2611, Found: 340.2618.

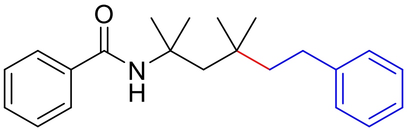

12d

***N*-(2,4,4-trimethyl-6-phenylhexan-2-yl)benzamide (12d),** 34.29 mg, yield: 53%. Yellow oil.

^{1}HNMR (300 MHz, $CDCl_3$) δ 7.65 (d, J = 6.9 Hz, 2H), 7.35-7.49 (m, 4H), 7.22-7.28 (m, 1H), 7.15 (d, J = 7.5 Hz, 3H), 5.93 (s, 1H), 2.61 (t, J = 8.7 Hz, 2H), 1.97 (s, 2H), 1.64 (t, J = 7.8 Hz, 2H), 1.55 (s, 6H), 1.11 (s, 6H).

^{13}CNMR (75 MHz, $CDCl_3$) δ 166.74, 143.21, 136.20, 131.00, 128.53, 128.32, 126.60, 125.56, 55.53, 49.37, 47.13, 34.47, 30.71, 29.56, 28.64.

HRMS (ESI):$C_{22}H_{29}NO+Na^+$ Calcd: 346.2147, Found: 346.2146.

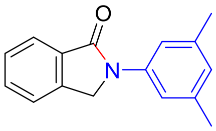

3f'

2-(3,5-dimethylphenyl)isoindolin-1-one (3f'), 22.5 mg, yield: 95%. White solid, mp: 132-135℃.

^{1}HNMR (300 MHz, $CDCl_3$): δ 7.92 (d, J = 8.1 Hz, 1H), 7.57-7.62 (m, 1H), 7.49-7.52 (m, 4H), 6.84 (s, 1H), 4.84 (s, 2H), 2.37 (s, 6H).

^{13}CNMR (75 MHz, $CDCl_3$): δ 167.46, 140.20, 139.35, 138.82, 133.38, 131.95, 128.32, 126.34, 124.10, 122.56, 117.51, 51.01, 21.60.

HRMS (ESI): $C_{16}H_{15}NO+Na^+$ Calcd: 260.1046, Found: 260.1062.

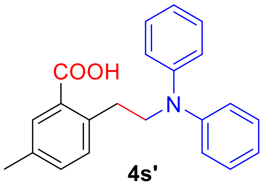

4s'

2-(2-(diphenylamino)ethyl)-5-methylbenzoic acid (4s'), 30.8 mg, yield: 93%. White solid, mp: 145-147℃.

^{1}HNMR (300 MHz, $CDCl_3$):δ 7.90 (s, 1H), 7.23-7.33 (m, 5H), 7.14 (d, *J* = 7.8 Hz, 1H), 7.05 (d, *J* = 7.8 Hz, 4H), 6.93 (t, *J* = 7.5 Hz, 2H), 4.01 (t, *J* = 7.5 Hz, 2H), 3.35 (t, *J* = 8.1 Hz, 2H), 2.40 (s, 3H).

^{13}CNMR (75 MHz, $CDCl_3$): δ 172.30, 147.85, 139.28, 136.27, 134.00, 132.18, 132.07, 129.21, 128.04, 121.08, 120.90, 53.71, 32.40, 20.90.

HRMS (ESI): $C_{22}H_{21}NO_2$+Na^+ Calcd: 354.1465, Found: 354.1468.

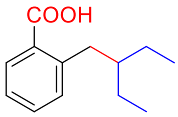

10b'

2-(2-ethylbutyl)benzoic acid (10b'), 21.6 mg, yield: 98%. White oil liquid.

^{1}HNMR (300 MHz, $CDCl_3$): δ 8.04 (d, *J* = 7.8 Hz, 1H), 7.45 (t, *J* = 7.5 Hz, 1H), 7.22-7.31 (m, 2H), 2.98 (d, *J* = 6.9 Hz, 2H), 1.52-1.59 (m, 1H), 1.25-1.36 (m, 4H), 0.87 (t, *J* = 7.5 Hz, 6H).

^{13}CNMR (75 MHz, $CDCl_3$): δ 173.65, 144.92, 132.36, 132.33, 131.64, 128.66, 125.82, 42.43, 38.34, 25.09, 10.73.

HRMS (ESI): $C_{13}H_{18}O_2$+Na^+ Calcd: 229.1199, Found: 229.1201.

NMR spectra of the products.

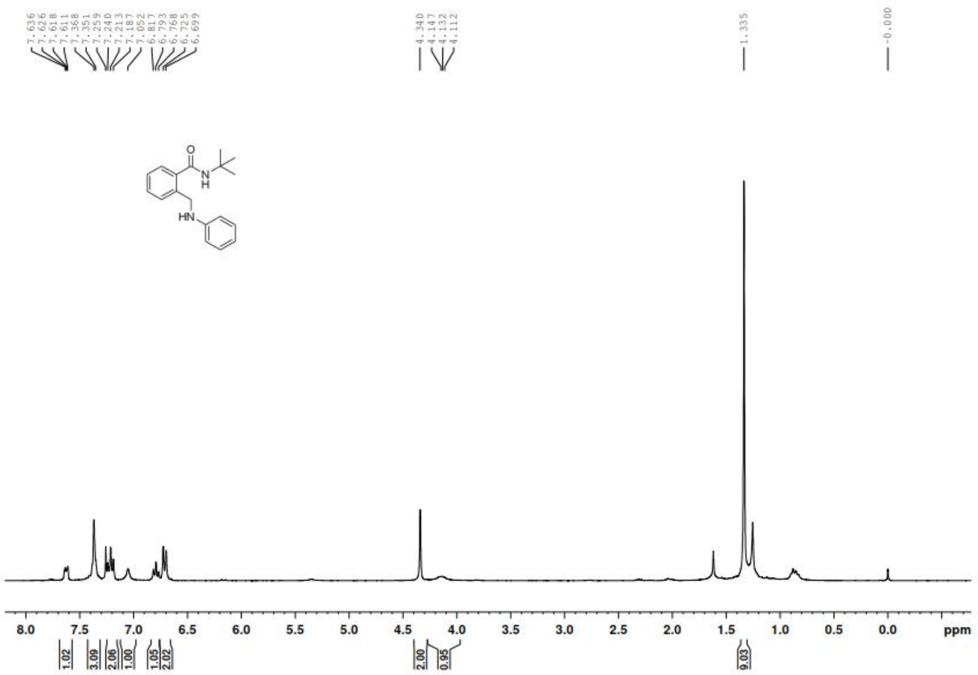

Supplementary Figure 22. ^{1}H NMR (300 MHz, $CDCl_3$) spectra for compound **3a.**

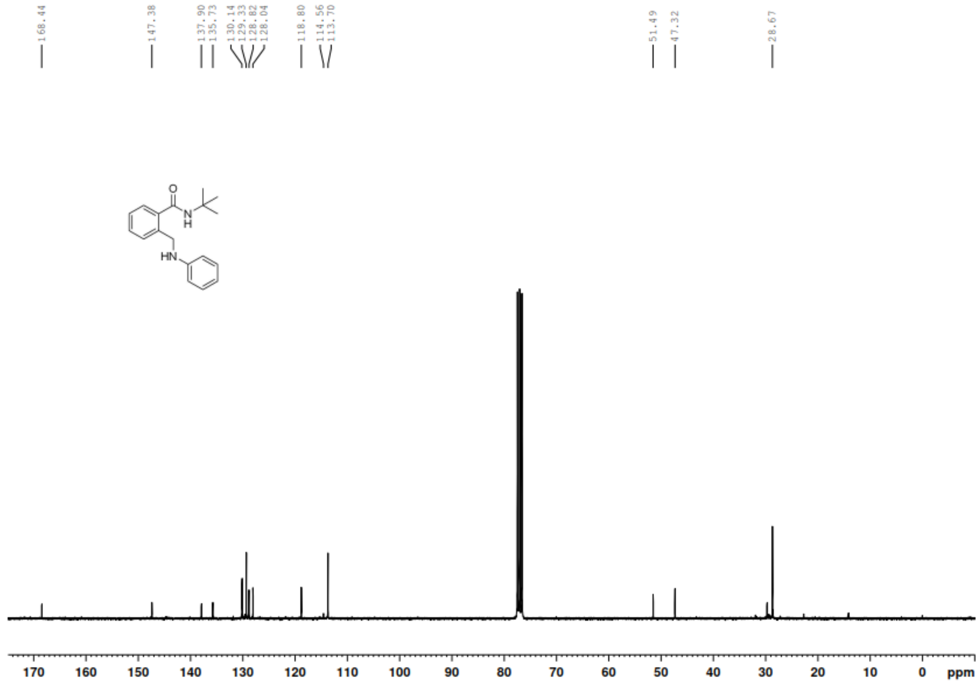

Supplementary Figure 23. ^{13}C NMR (75 MHz, $CDCl_3$) spectra for compound **3a.**

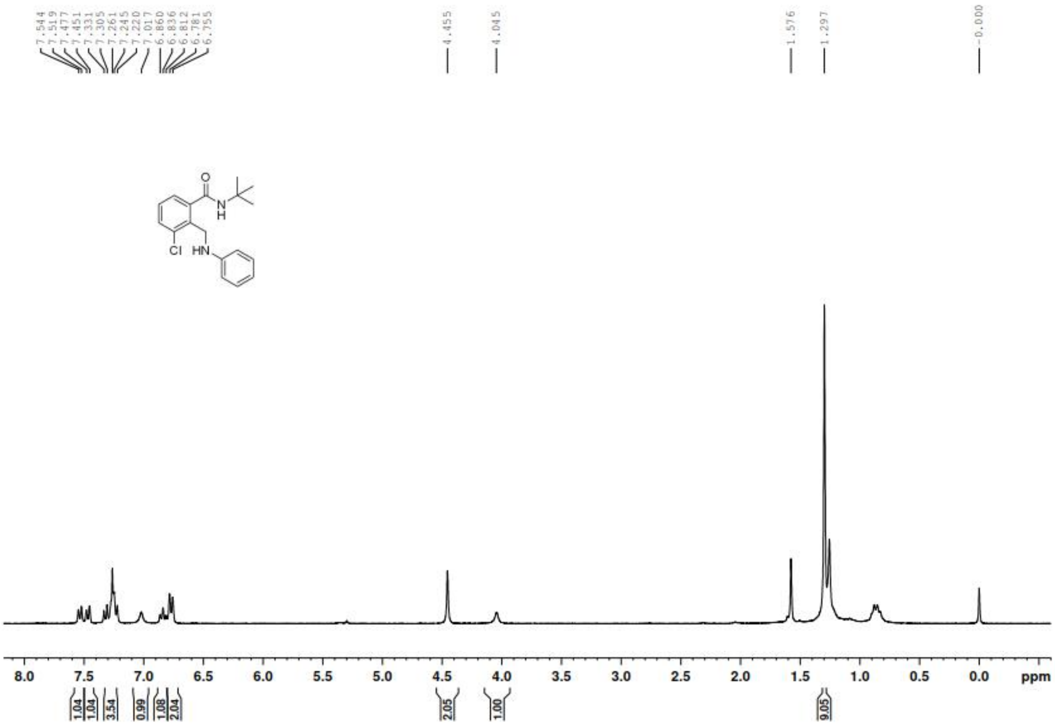

Supplementary Figure 24. ^{1}H NMR (300 MHz, $CDCl_3$) spectra for compound **3b.**

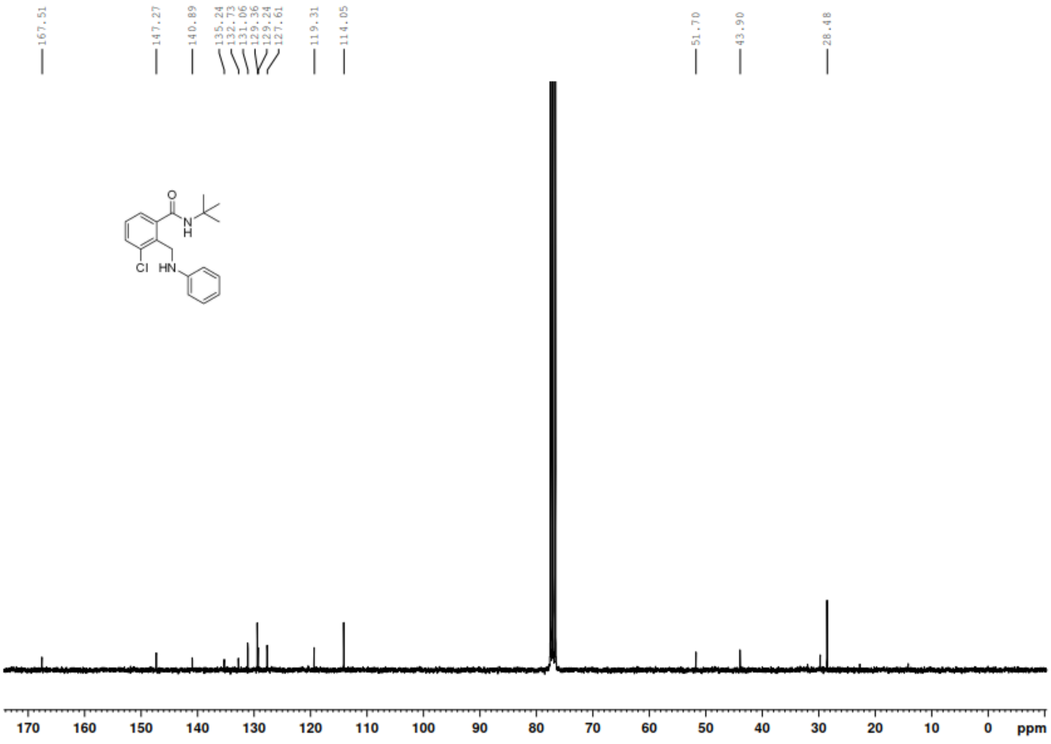

Supplementary Figure 25. ^{13}C NMR (75 MHz, $CDCl_3$) spectra for compound **3b.**

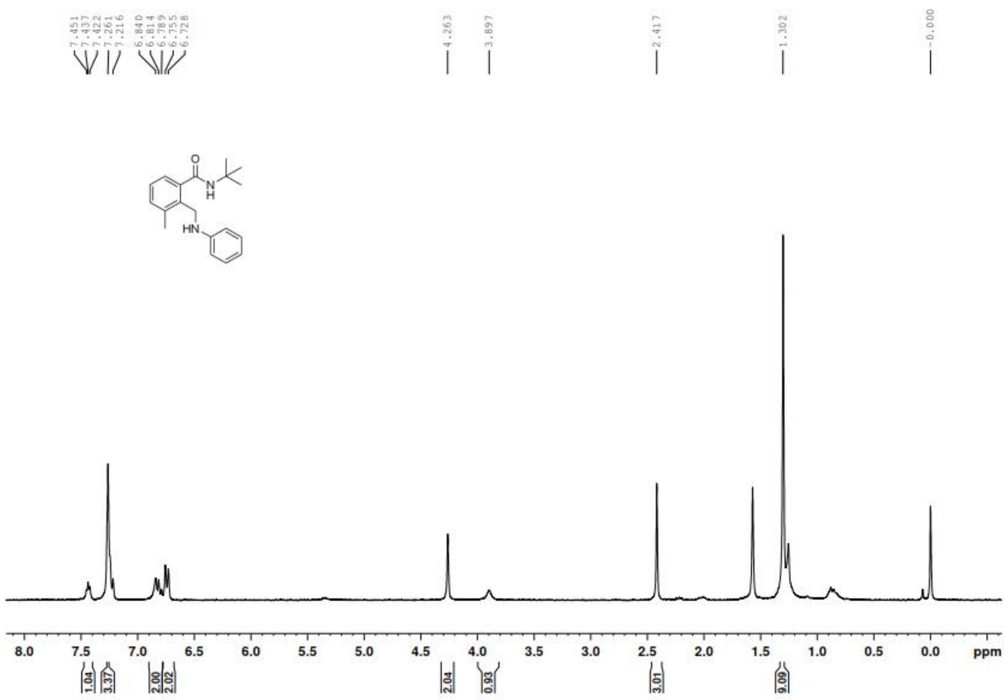

Supplementary Figure 26. ^{1}H NMR (300 MHz, $CDCl_3$) spectra for compound **3c.**

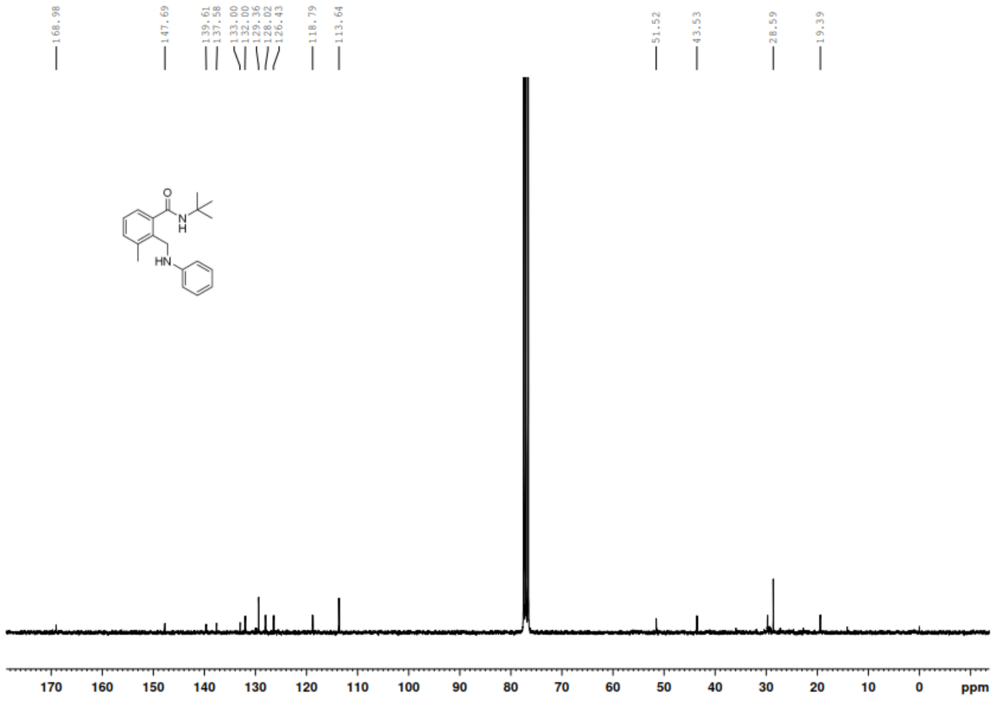

Supplementary Figure 27. ^{13}C NMR (75 MHz, $CDCl_3$) spectra for compound **3c.**

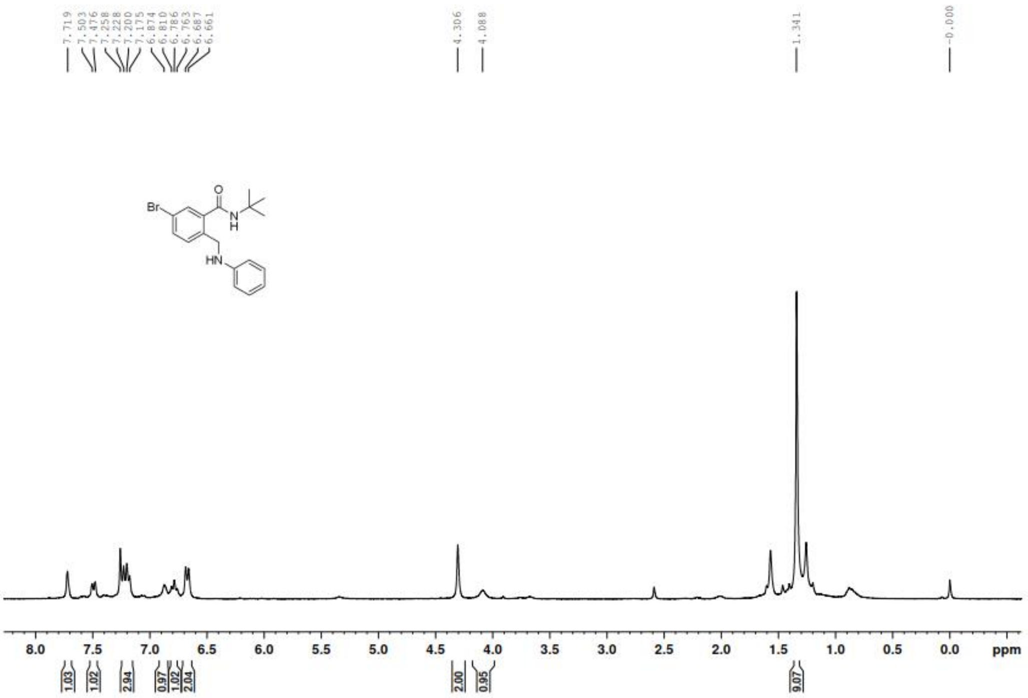

Supplementary Figure 28. ^{1}H NMR (300 MHz, $CDCl_3$) spectra for compound **3d.**

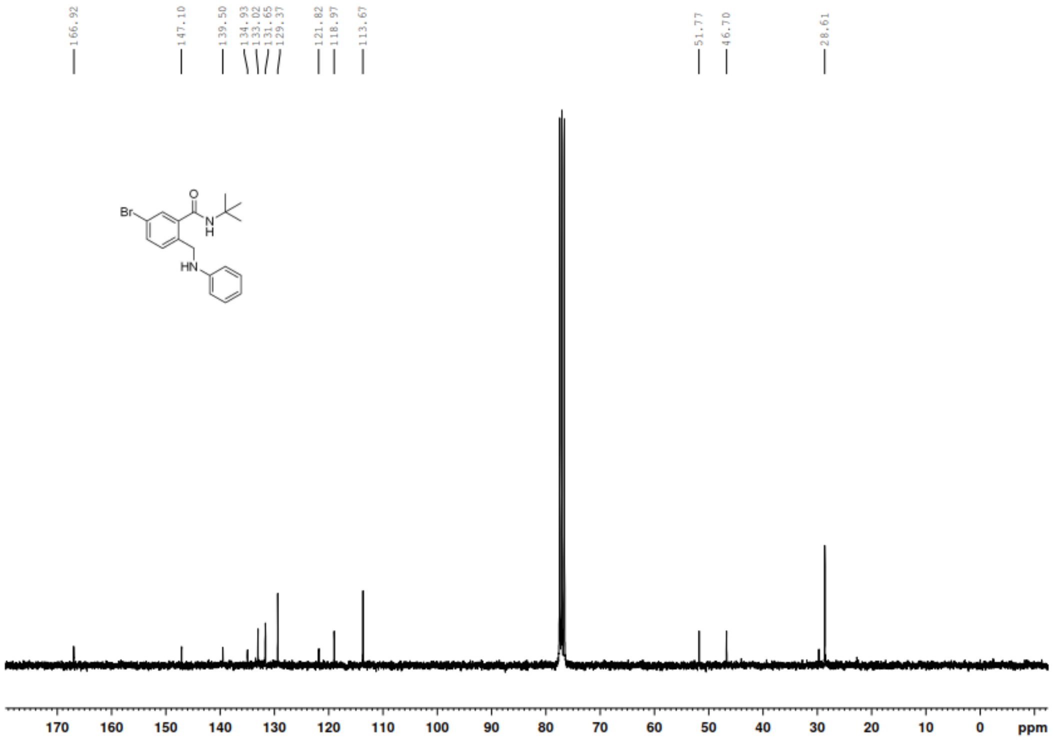

Supplementary Figure 29. ^{13}C NMR (75 MHz, $CDCl_3$) spectra for compound **3d.**

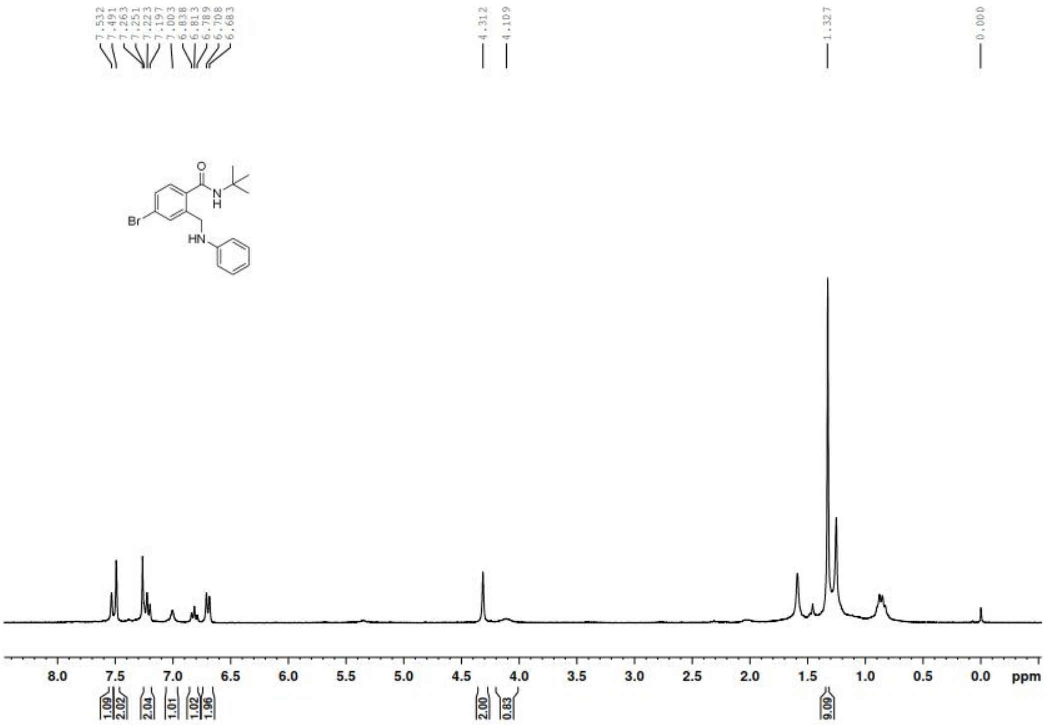

Supplementary Figure 30. ^{1}H NMR (300 MHz, $CDCl_3$) spectra for compound **3e.**

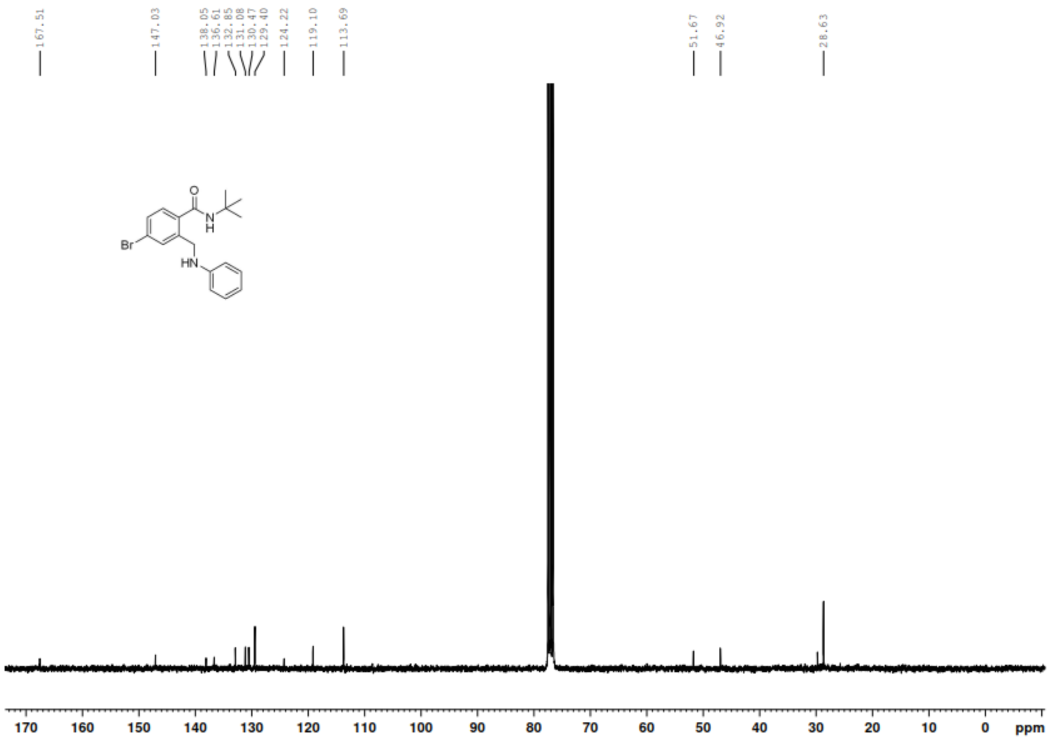

Supplementary Figure 31. ^{13}C NMR (75 MHz, $CDCl_3$) spectra for compound **3e.**

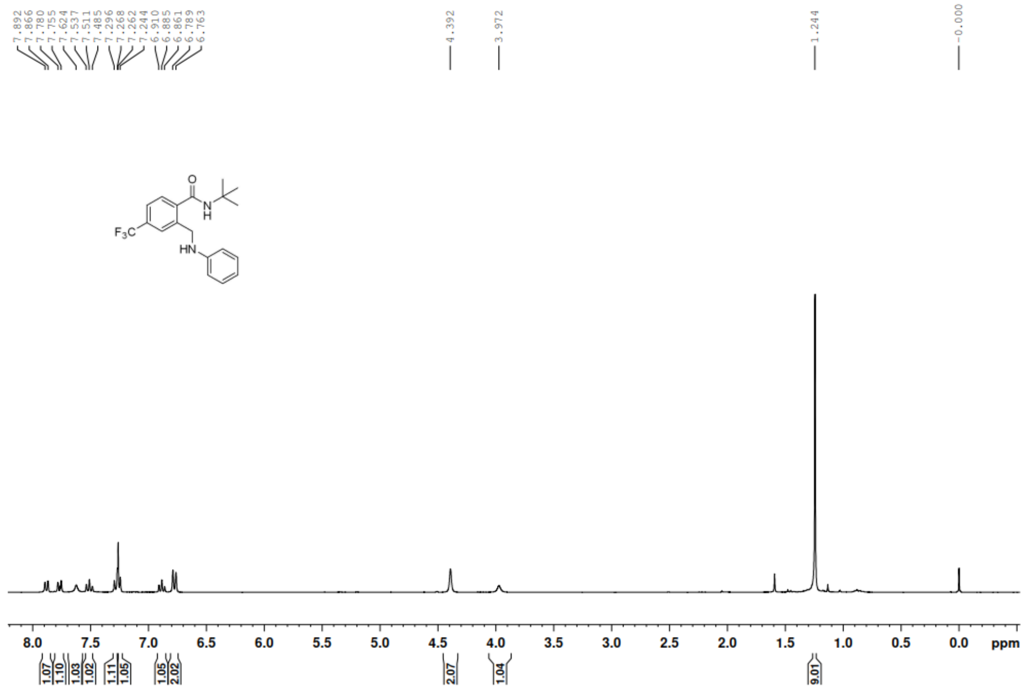

Supplementary Figure 32. ^{1}H NMR (300 MHz, $CDCl_3$) spectra for compound **3f.**

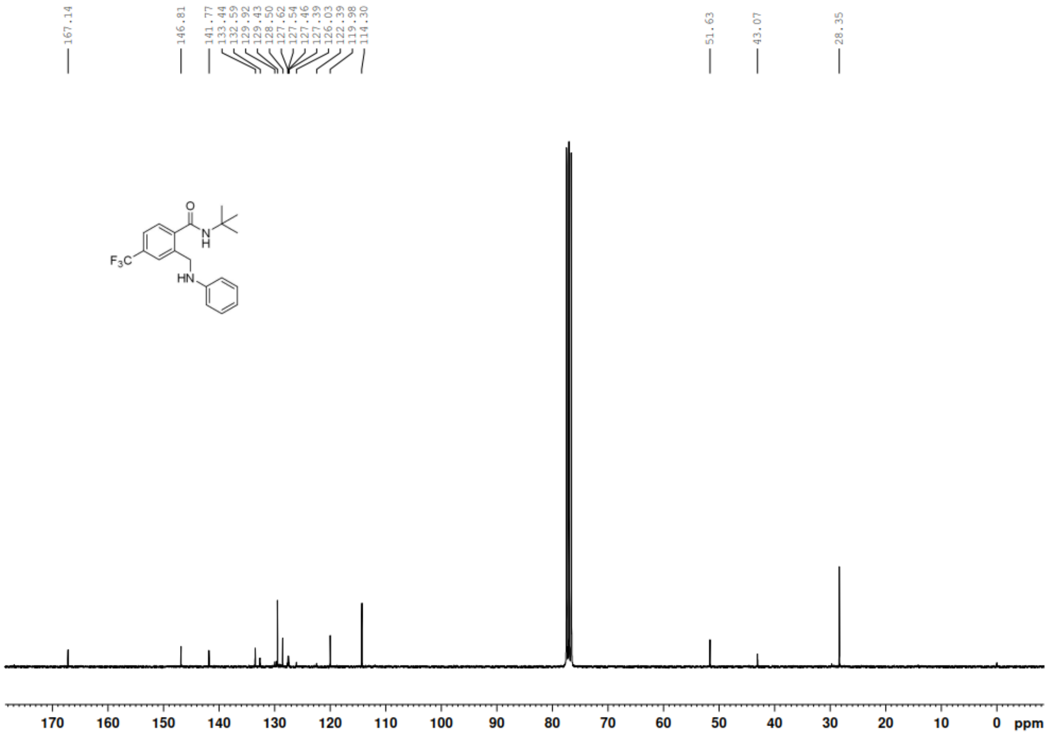

Supplementary Figure 33. ^{13}C NMR (75 MHz, $CDCl_3$) spectra for compound **3f.**

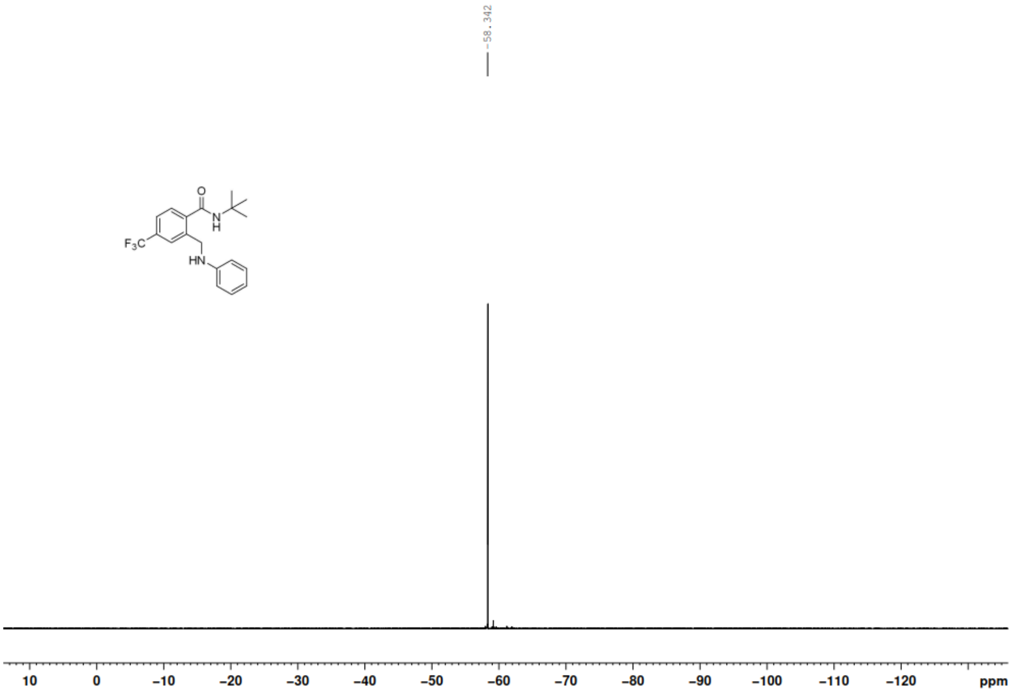

Supplementary Figure 34. ^{19}F NMR (282 MHz, $CDCl_3$) spectra for compound **3f.**

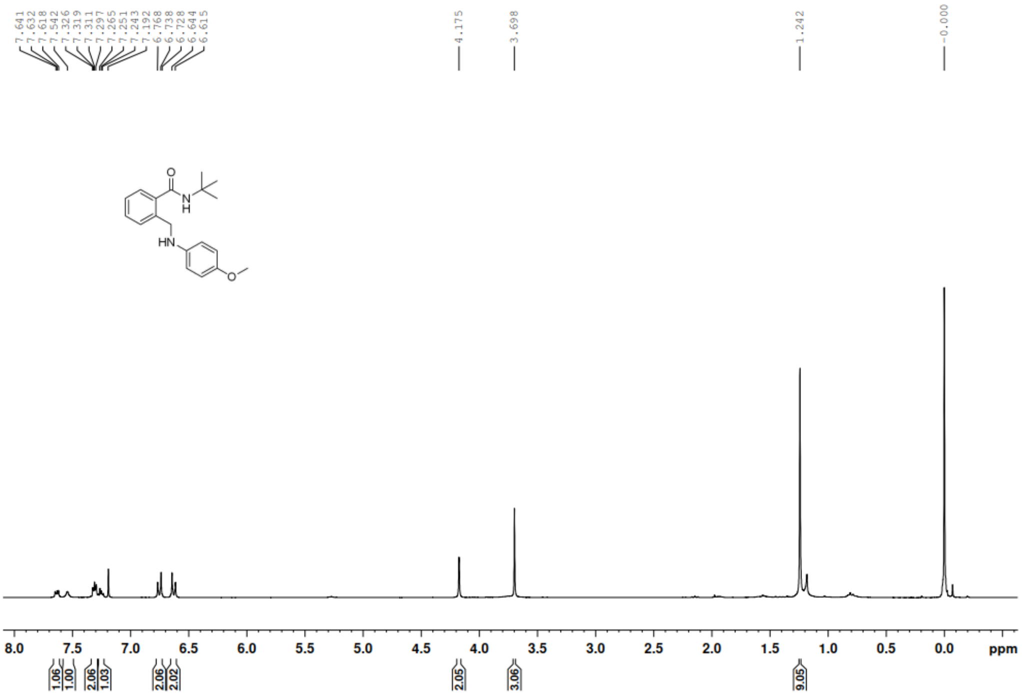

Supplementary Figure 35. ^{1}H NMR (300 MHz, $CDCl_3$) spectra for compound **3g.**

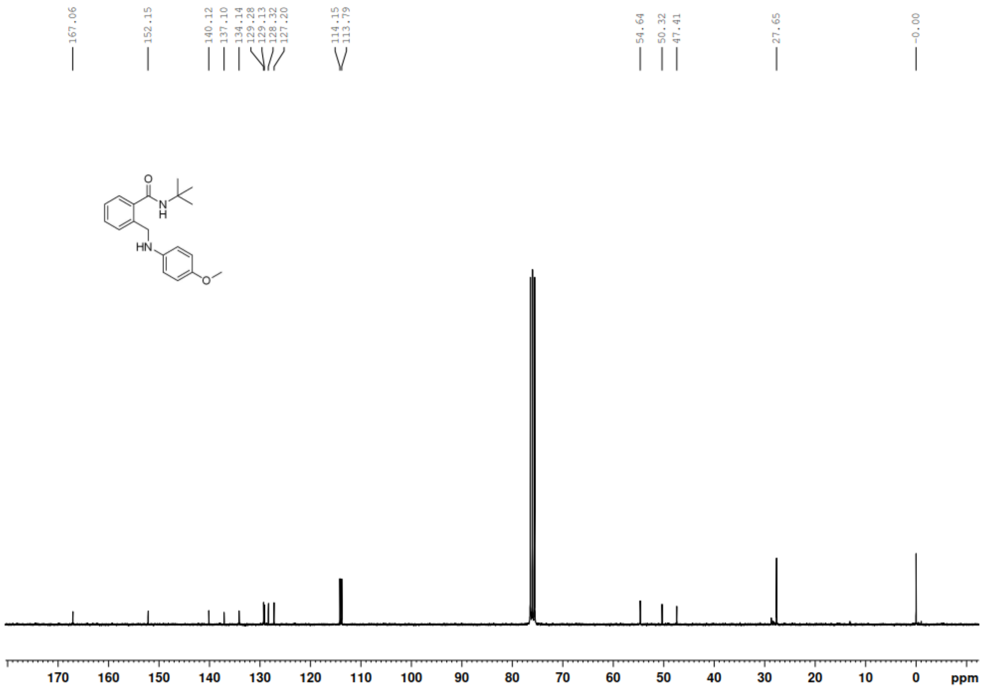

Supplementary Figure 36. ^{13}C NMR (75 MHz, $CDCl_3$) spectra for compound **3g.**

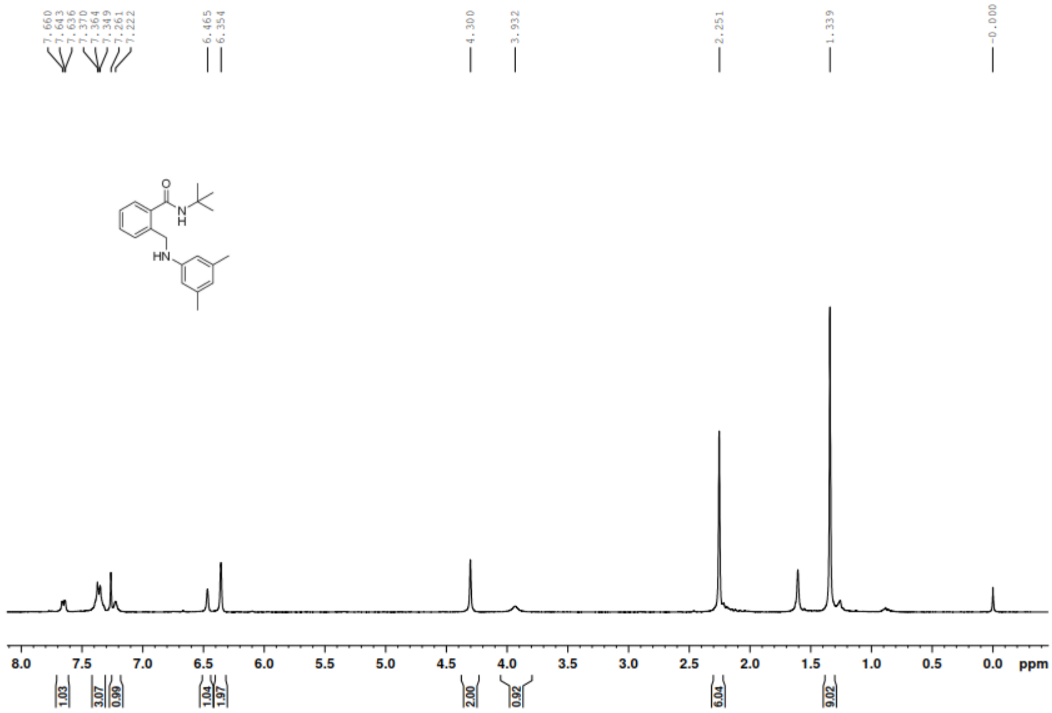

Supplementary Figure 37. ^{1}H NMR (300 MHz, $CDCl_3$) spectra for compound **3h.**

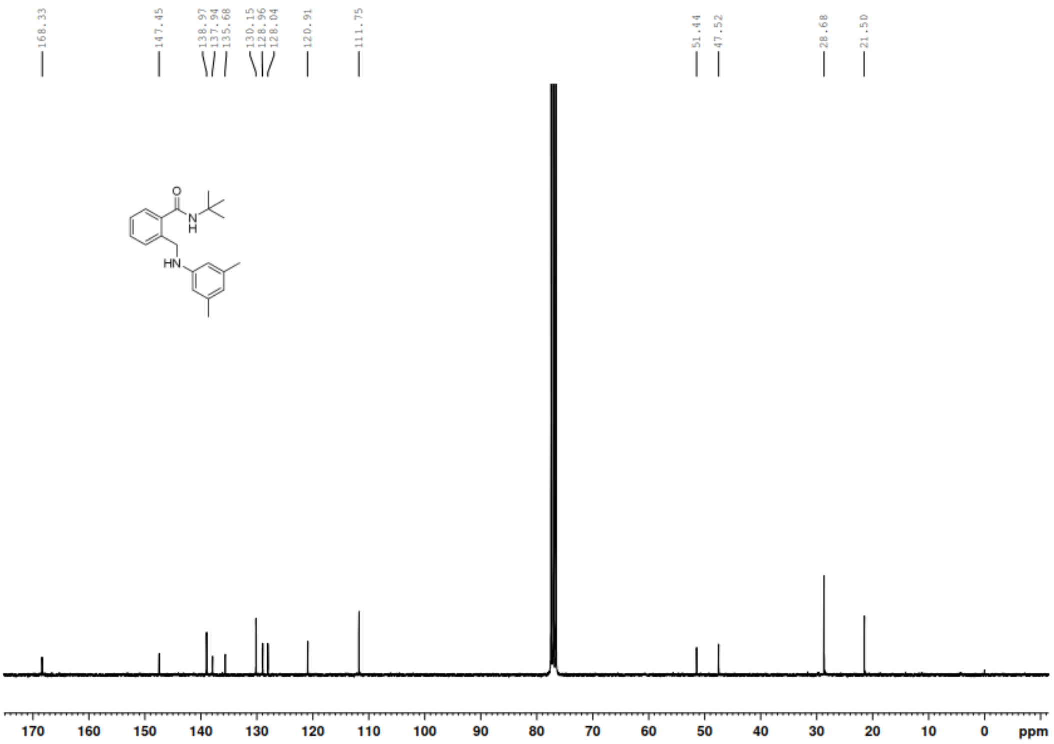

Supplementary Figure 38. ^{13}C NMR (75 MHz, $CDCl_3$) spectra for compound **3h.**

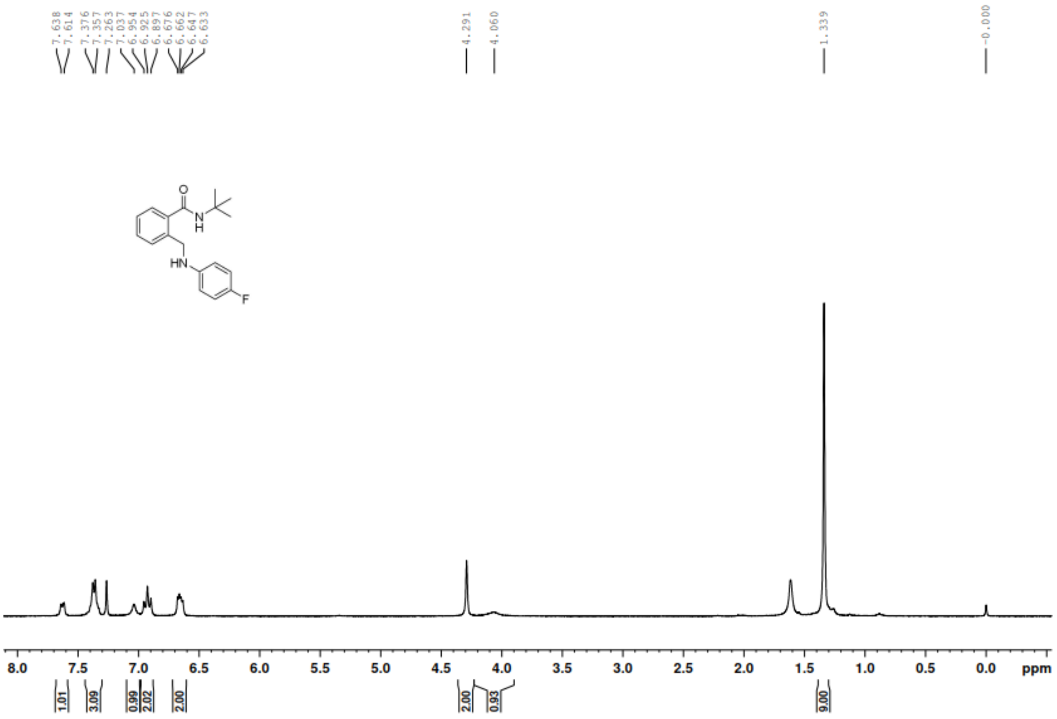

Supplementary Figure 39. ^{1}H NMR (300 MHz, $CDCl_3$) spectra for compound **3i.**

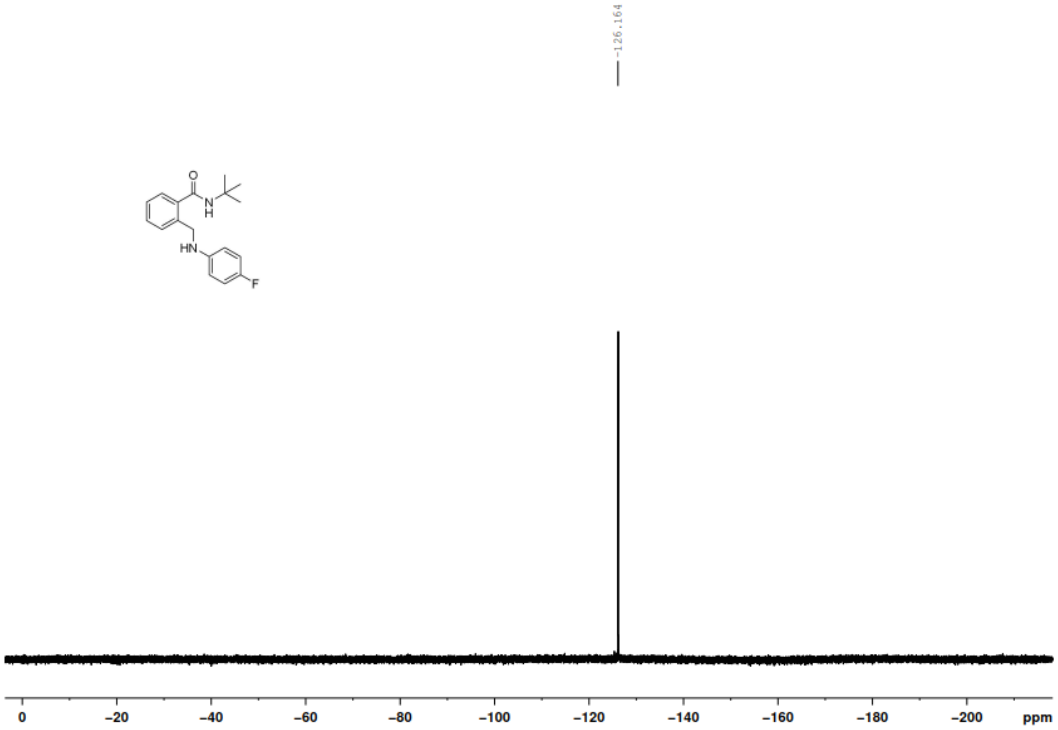

Supplementary Figure 40. ^{19}F NMR (282 MHz, $CDCl_3$) spectra for compound **3i.**

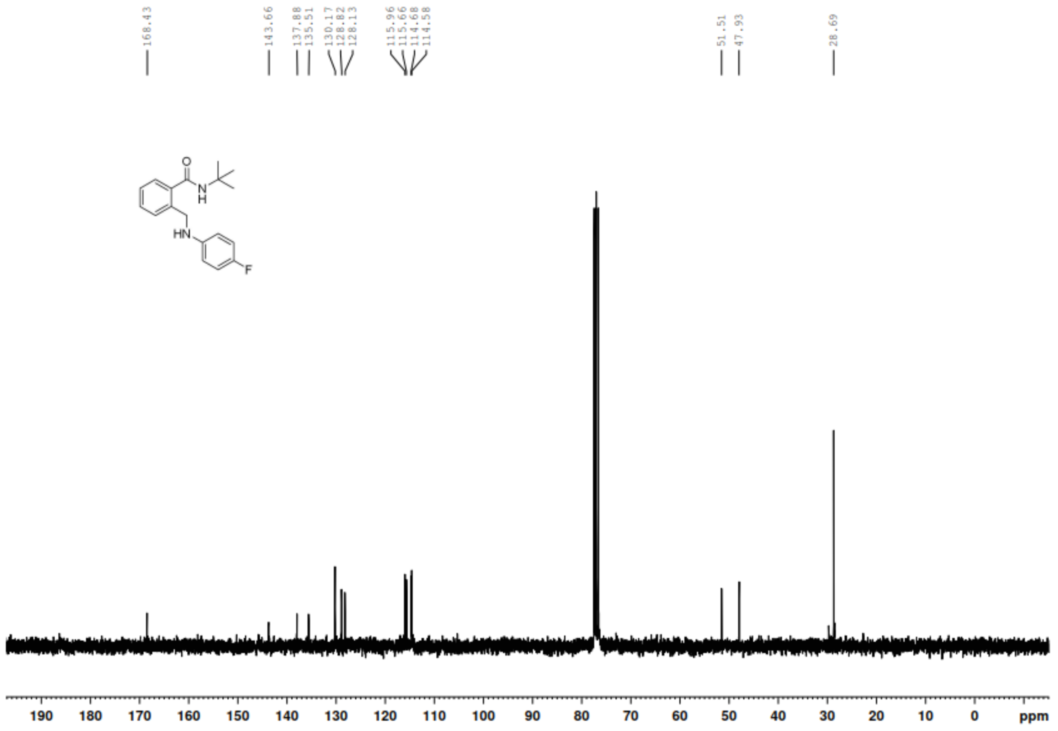

Supplementary Figure 41. ^{13}C NMR (75 MHz, $CDCl_3$) spectra for compound **3i.**

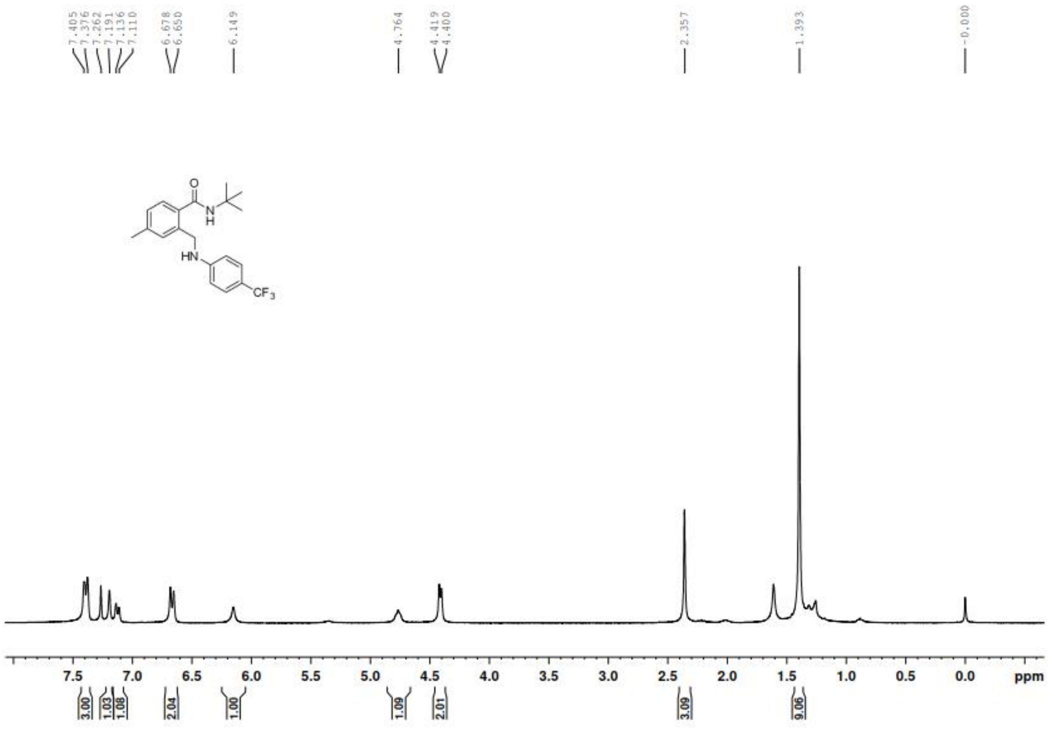

Supplementary Figure 42. ^{1}H NMR (300 MHz, $CDCl_3$) spectra for compound **3j.**

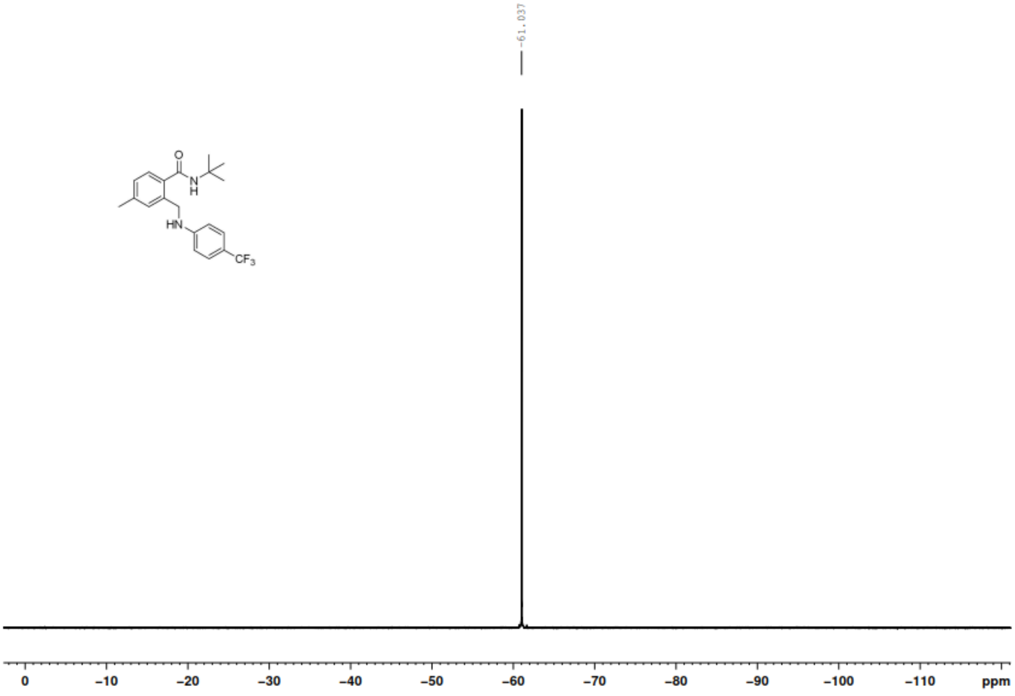

Supplementary Figure 43. ^{19}F NMR (282 MHz, $CDCl_3$) spectra for compound **3j.**

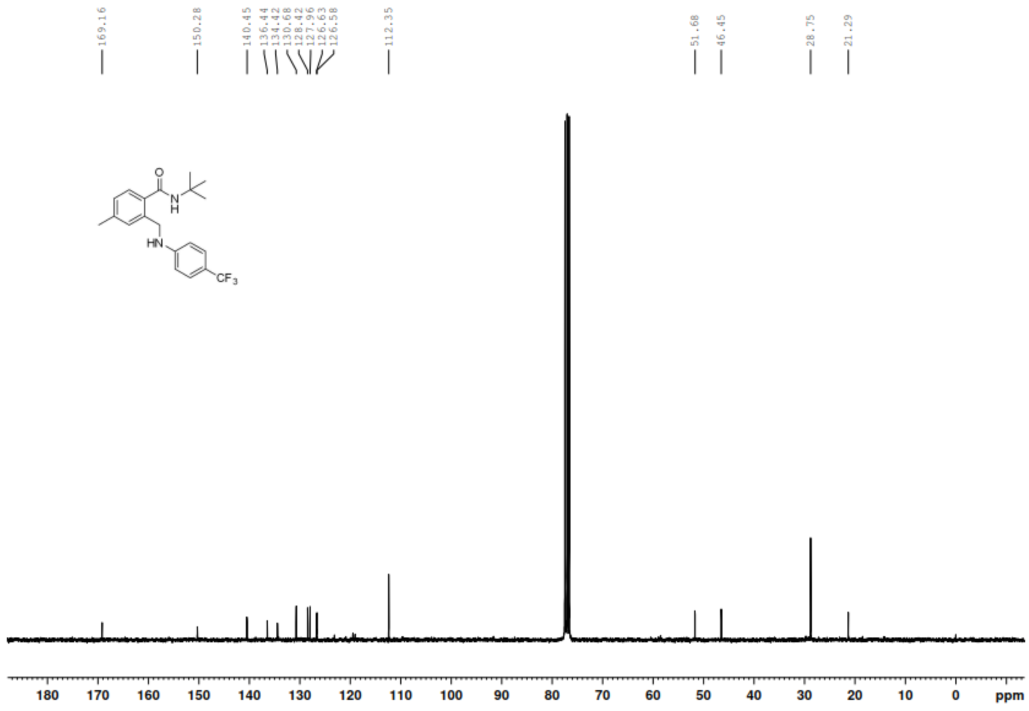

Supplementary Figure 44. ^{13}C NMR (75 MHz, $CDCl_3$) spectra for compound **3j.**

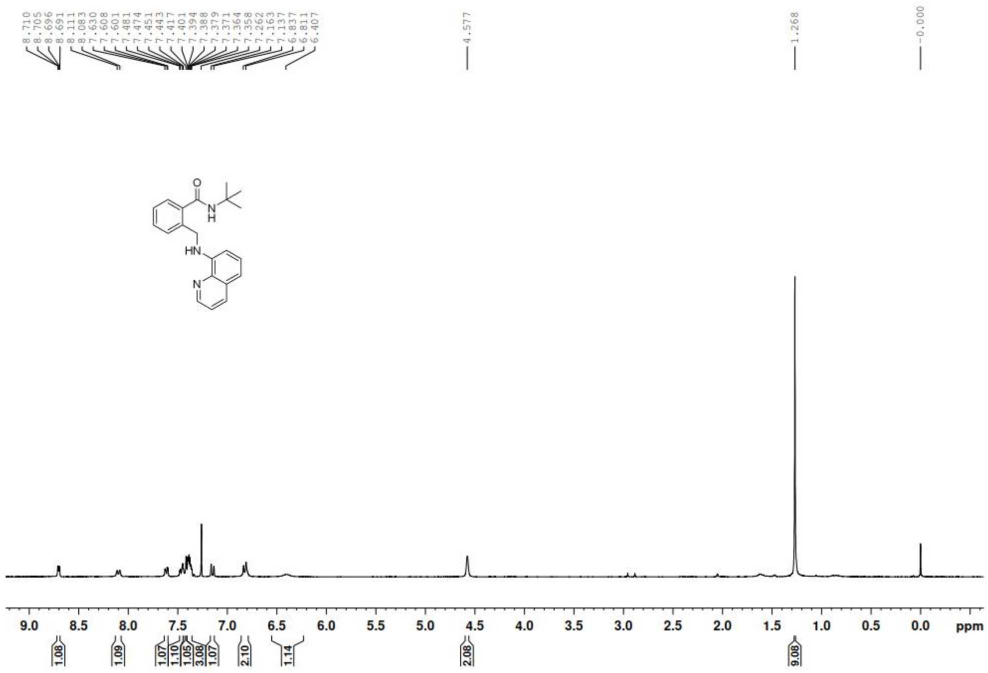

Supplementary Figure 45. ^{1}H NMR (300 MHz, $CDCl_3$) spectra for compound **3k.**

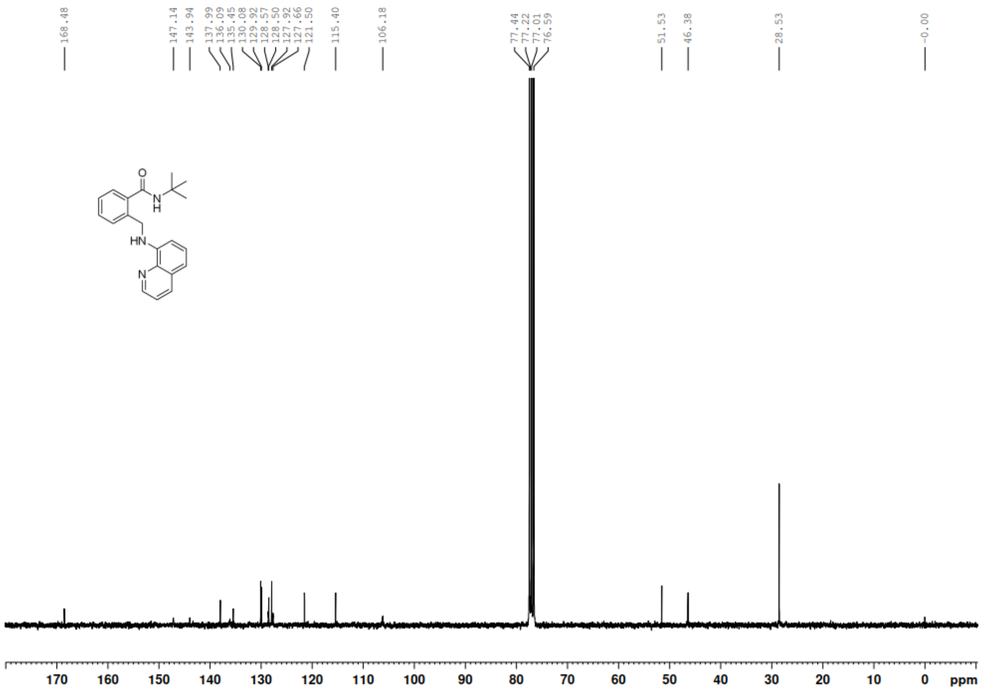

Supplementary Figure 46. ^{13}C NMR (75 MHz, $CDCl_3$) spectra for compound **3k.**

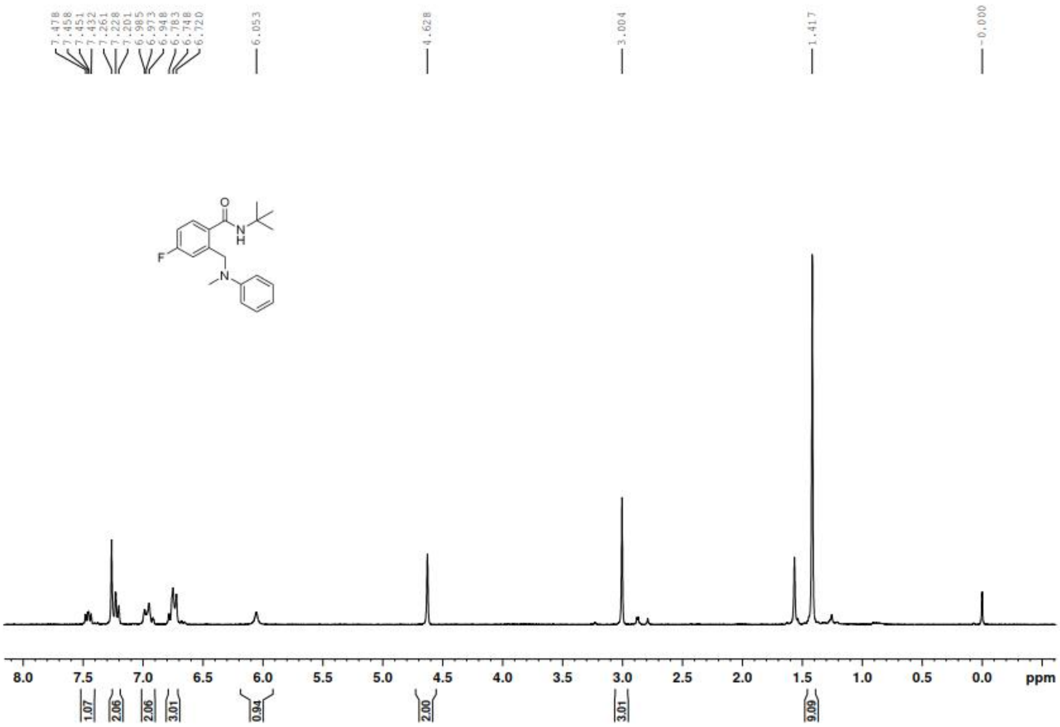

Supplementary Figure 47. ^{1}H NMR (300 MHz, $CDCl_3$) spectra for compound **3l.**

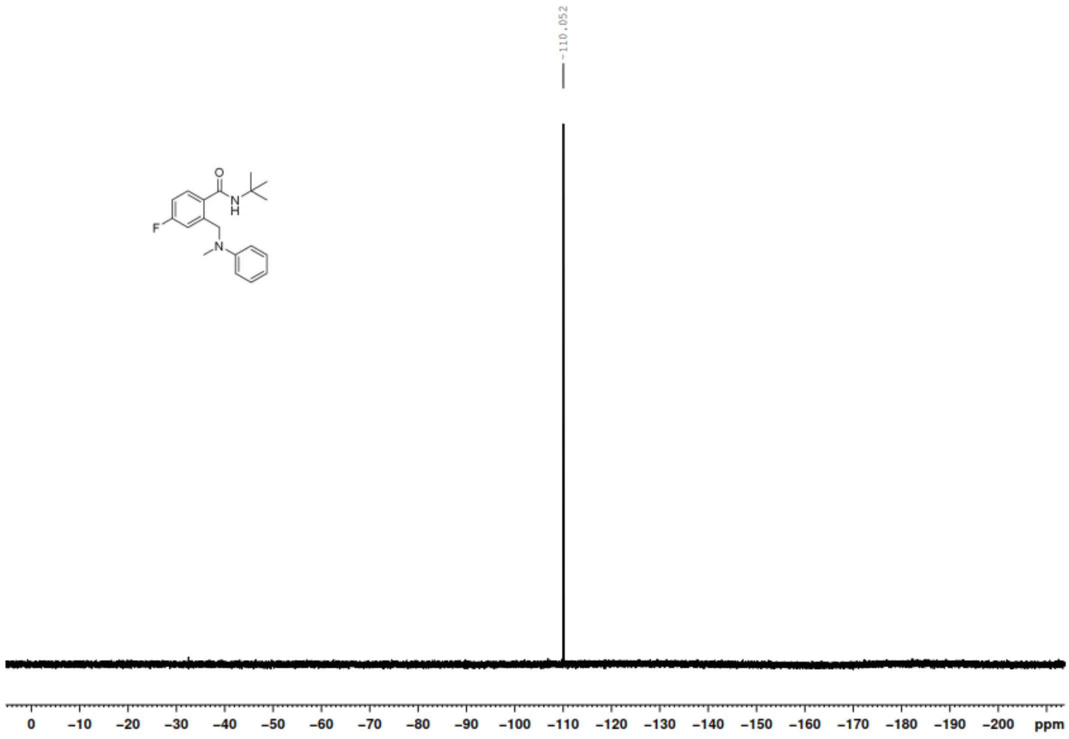

Supplementary Figure 48. ^{19}F NMR (282 MHz, $CDCl_3$) spectra for compound **3l.**

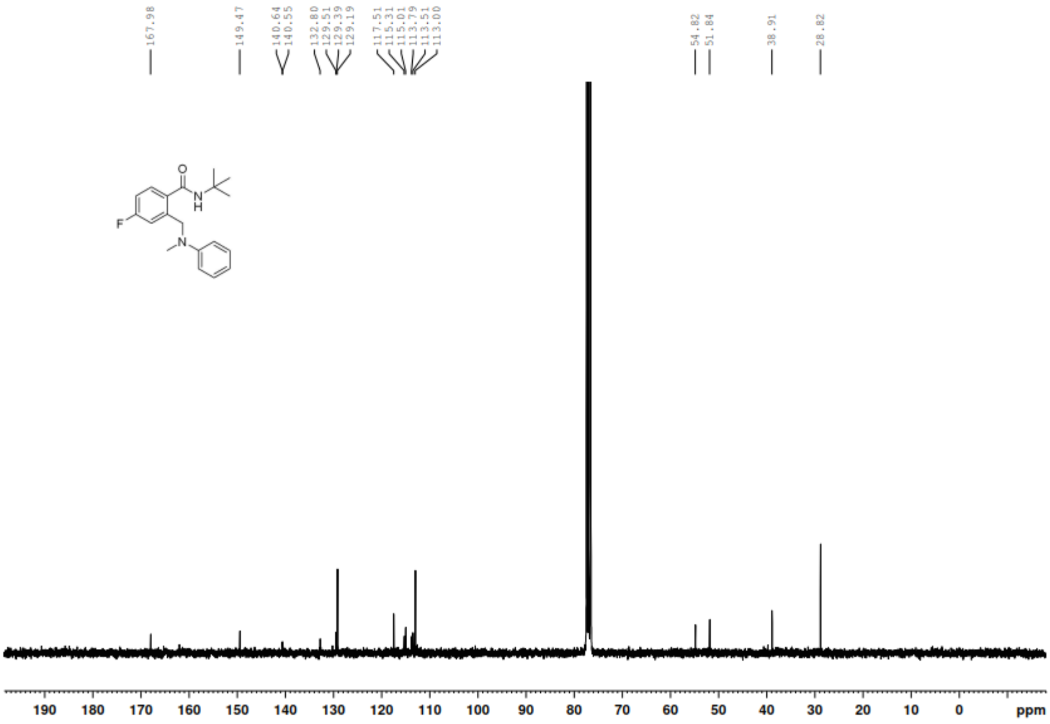

Supplementary Figure 49. ^{13}C NMR (75 MHz, $CDCl_3$) spectra for compound **3l.**

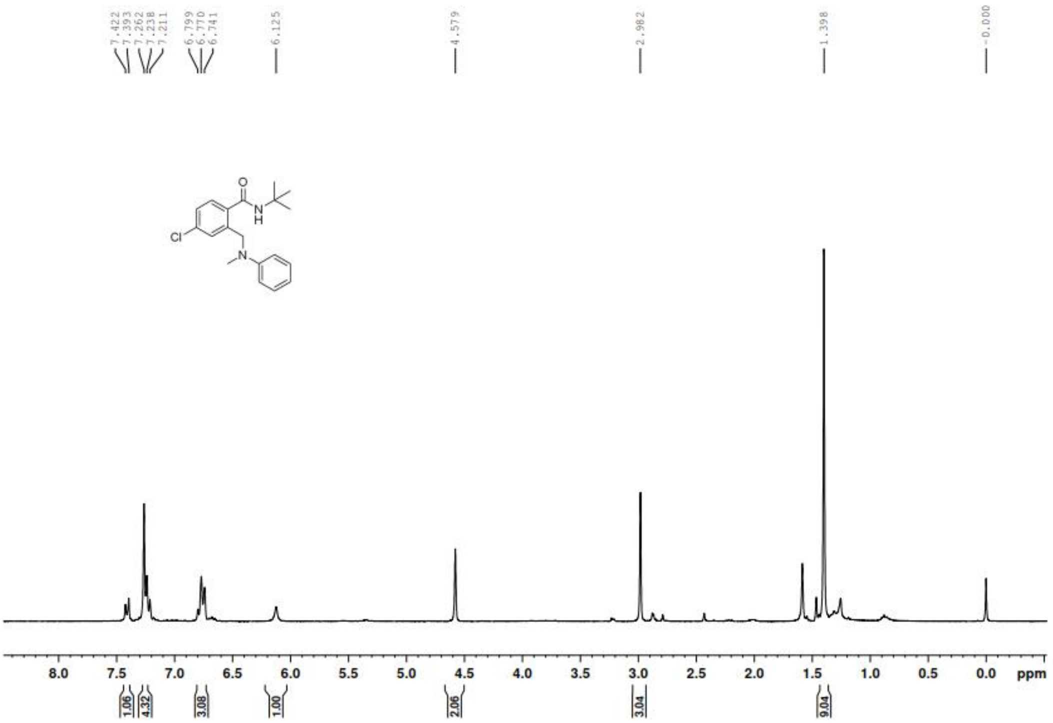

Supplementary Figure 50. ^{1}H NMR (300 MHz, $CDCl_3$) spectra for compound **3m.**

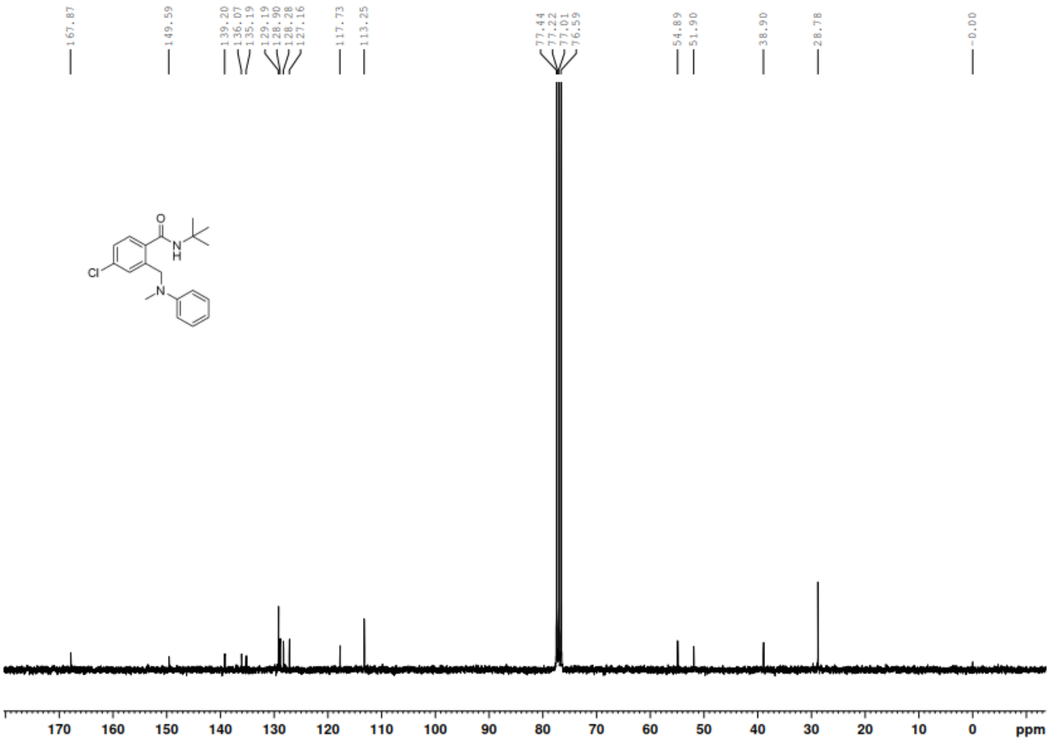

Supplementary Figure 51. ^{13}C NMR (75 MHz, $CDCl_3$) spectra for compound **3m.**

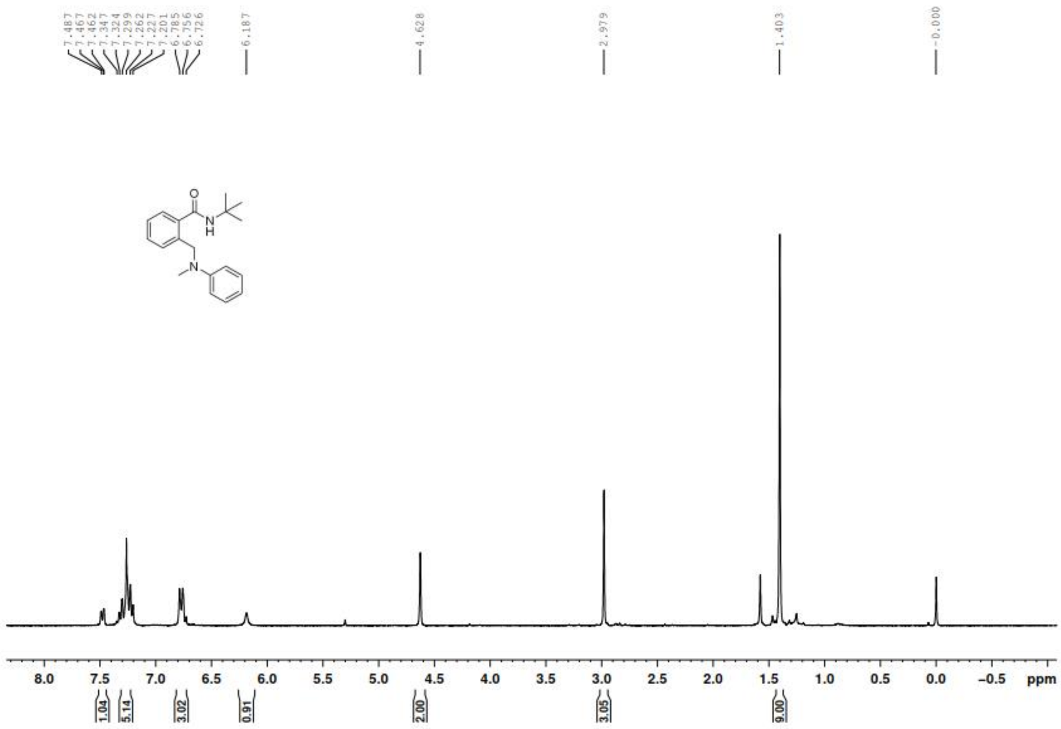

Supplementary Figure 52. ^{1}H NMR (300 MHz, $CDCl_3$) spectra for compound **3n.**

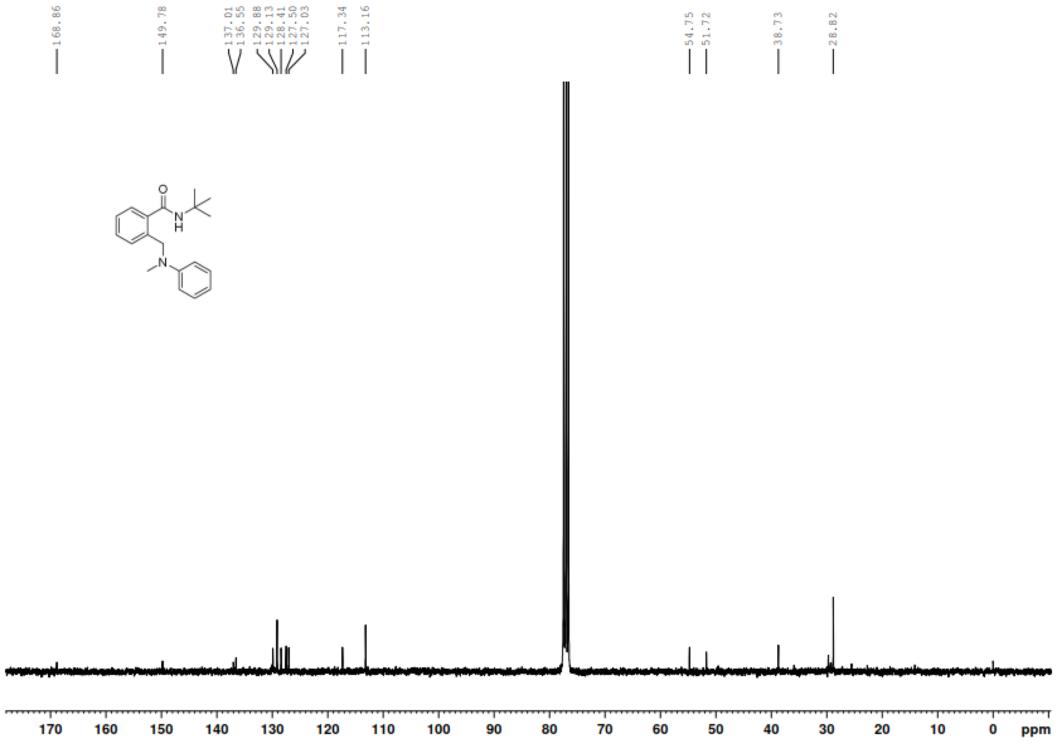

Supplementary Figure 53. ^{13}C NMR (75 MHz, $CDCl_3$) spectra for compound **3n.**

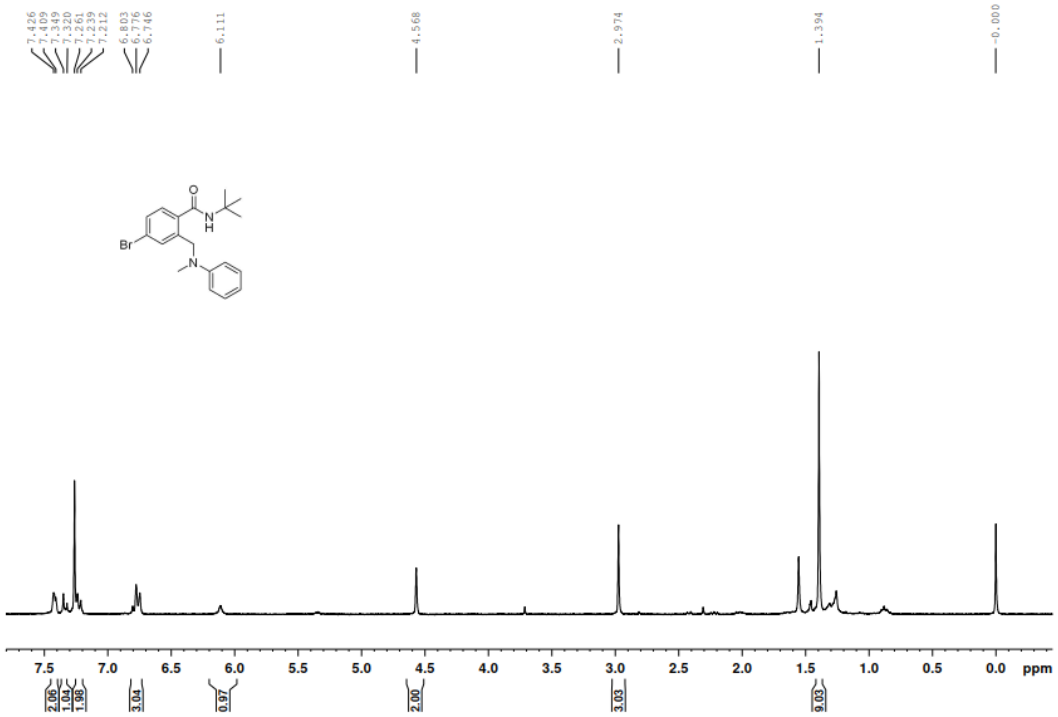

Supplementary Figure 54. ^{1}H NMR (300 MHz, $CDCl_3$) spectra for compound **3o.**

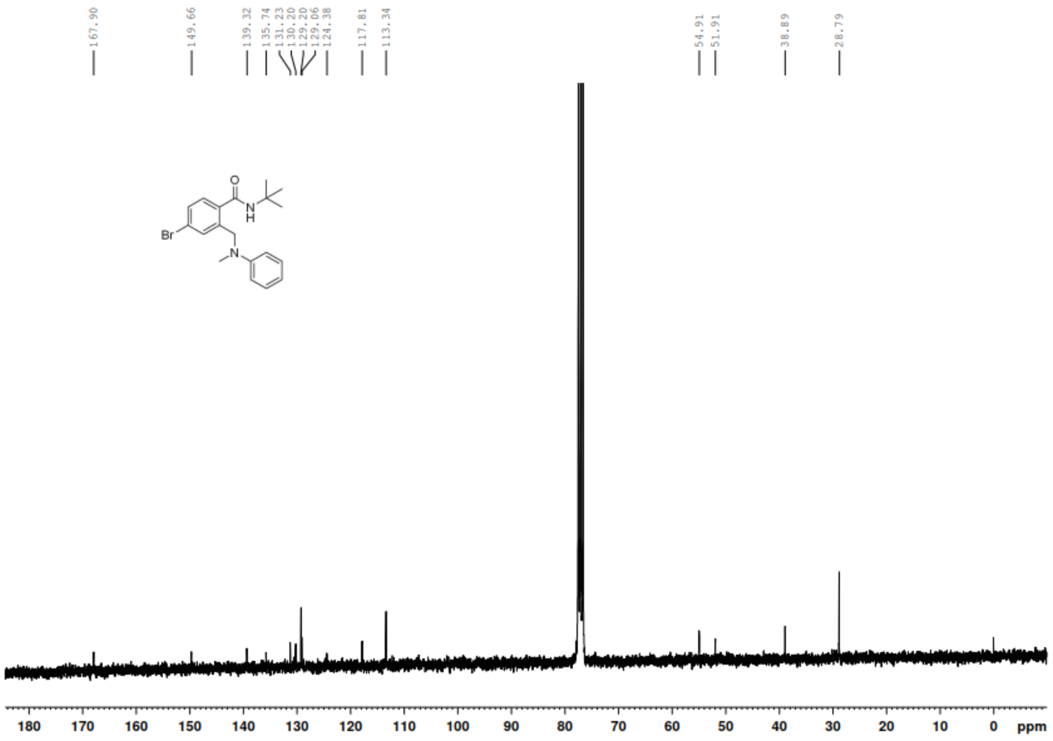

Supplementary Figure 55. ^{13}C NMR (75 MHz, $CDCl_3$) spectra for compound **3o.**

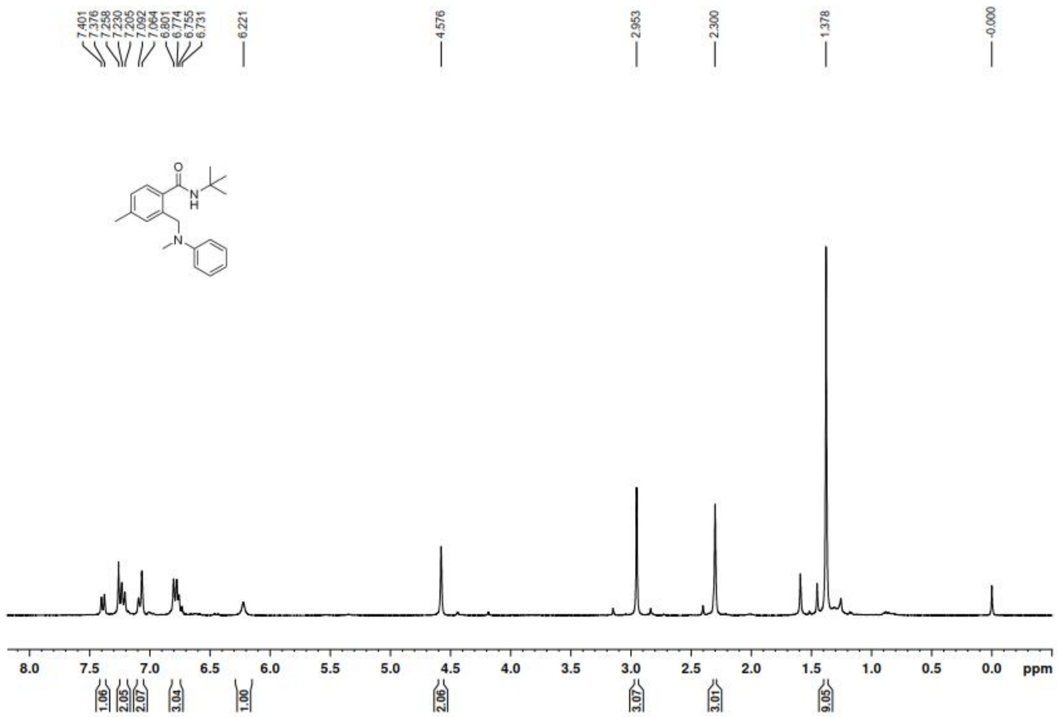

Supplementary Figure 56. ^{1}H NMR (300 MHz, $CDCl_3$) spectra for compound **3p.**

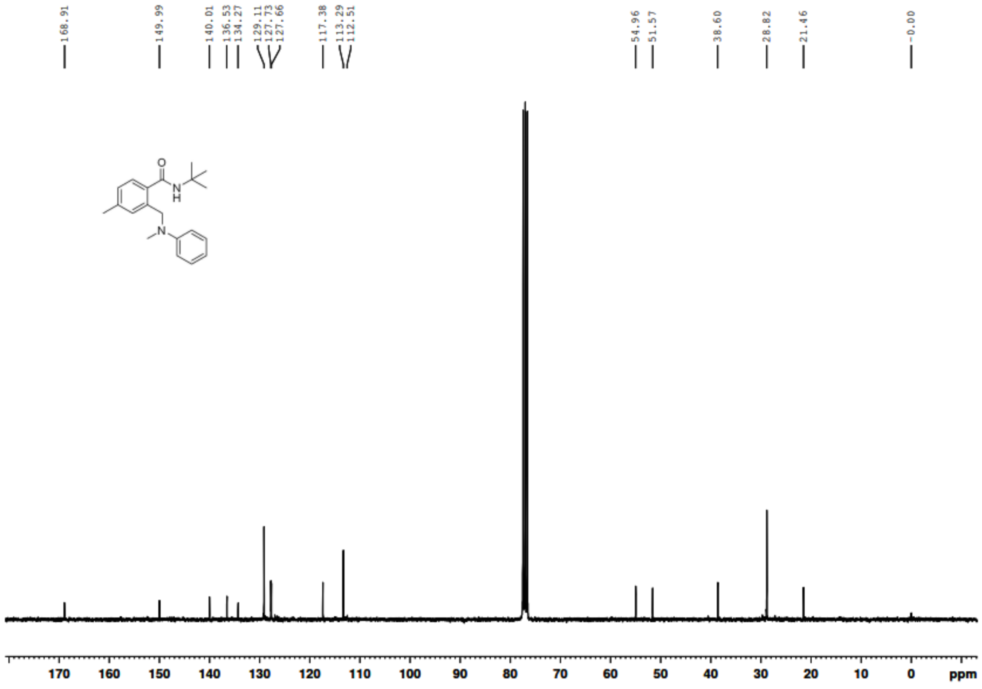

Supplementary Figure 57. ^{13}C NMR (75 MHz, $CDCl_3$) spectra for compound **3p.**

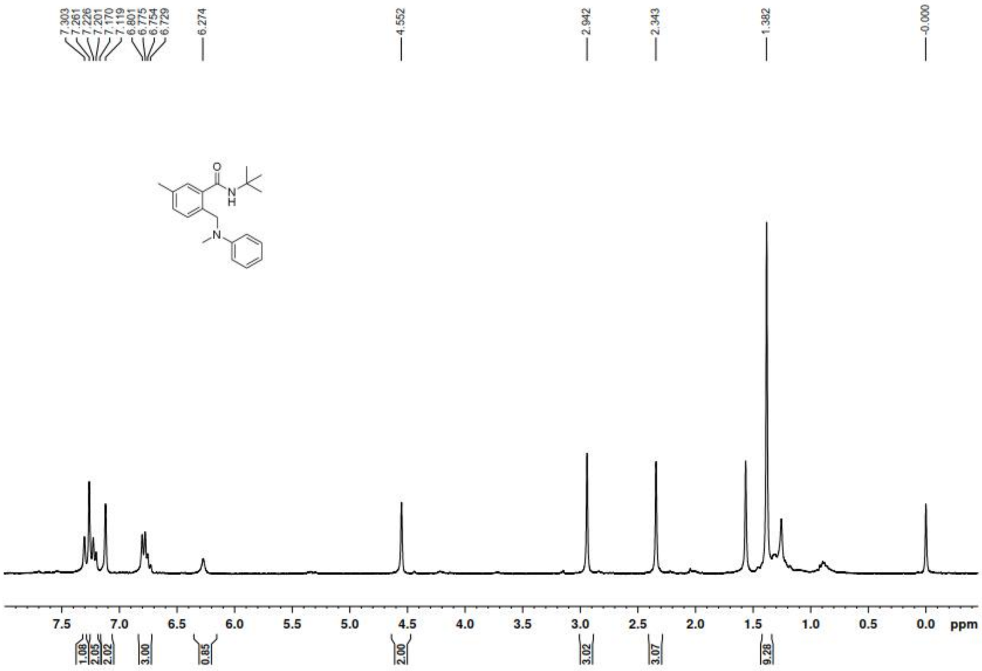

Supplementary Figure 58. ^{1}H NMR (300 MHz, $CDCl_3$) spectra for compound **3q.**

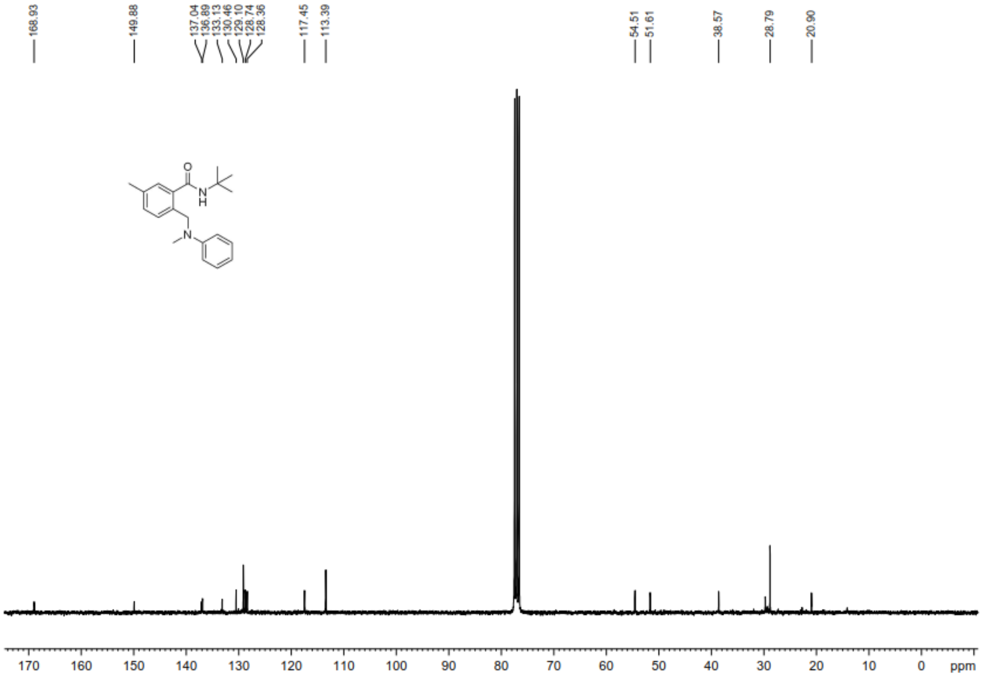

Supplementary Figure 59. ^{13}C NMR (75 MHz, $CDCl_3$) spectra for compound **3q.**

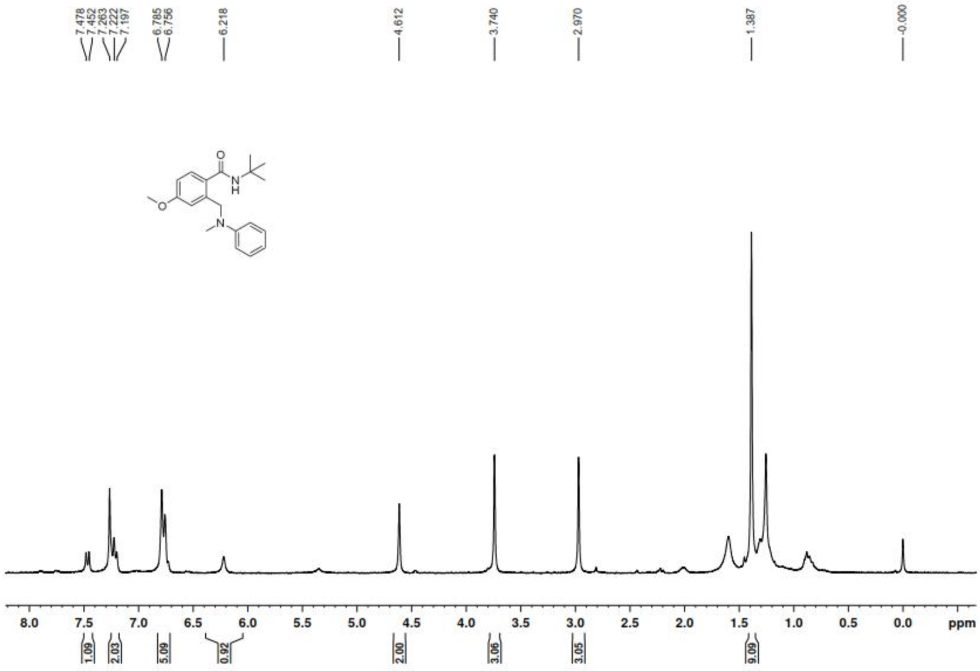

Supplementary Figure 60. ^{1}H NMR (300 MHz, $CDCl_3$) spectra for compound **3r.**

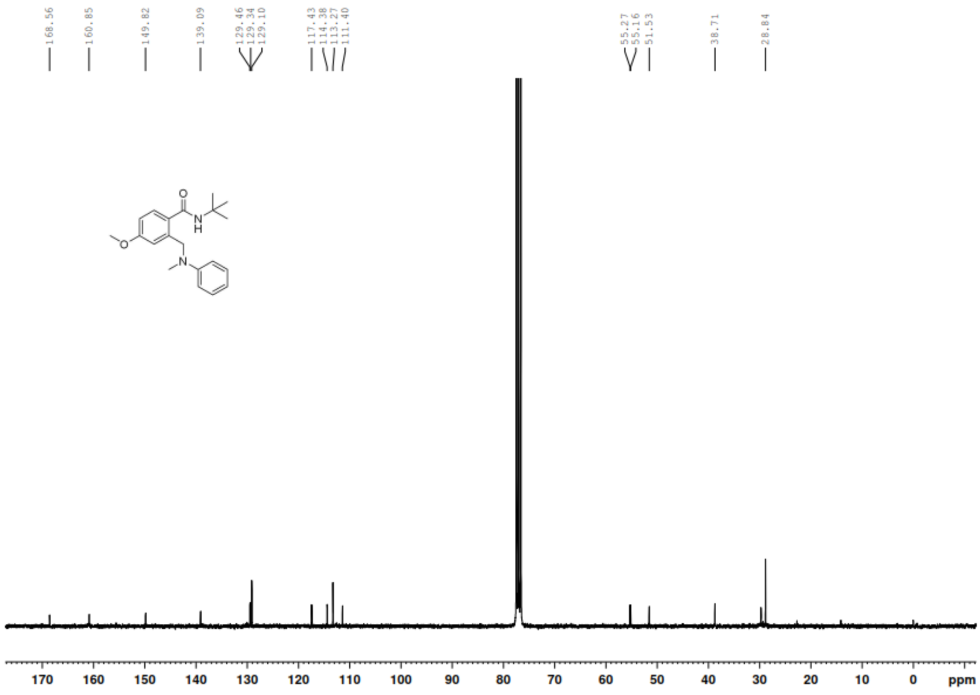

Supplementary Figure 61. ^{13}C NMR (75 MHz, $CDCl_3$) spectra for compound **3r.**

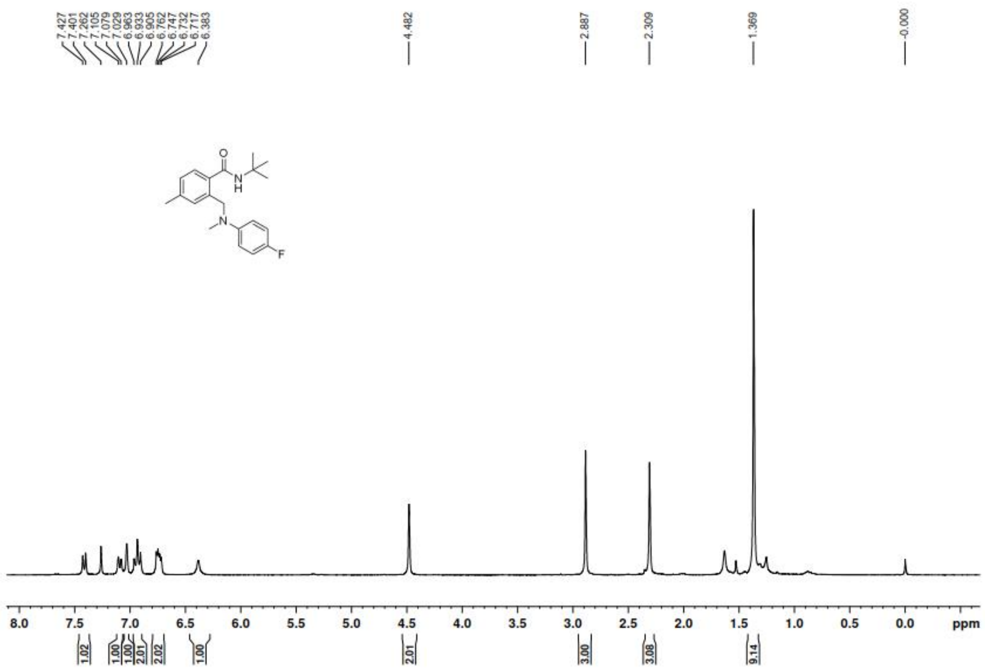

Supplementary Figure 62. ^{1}H NMR (300 MHz, $CDCl_3$) spectra for compound **3s.**

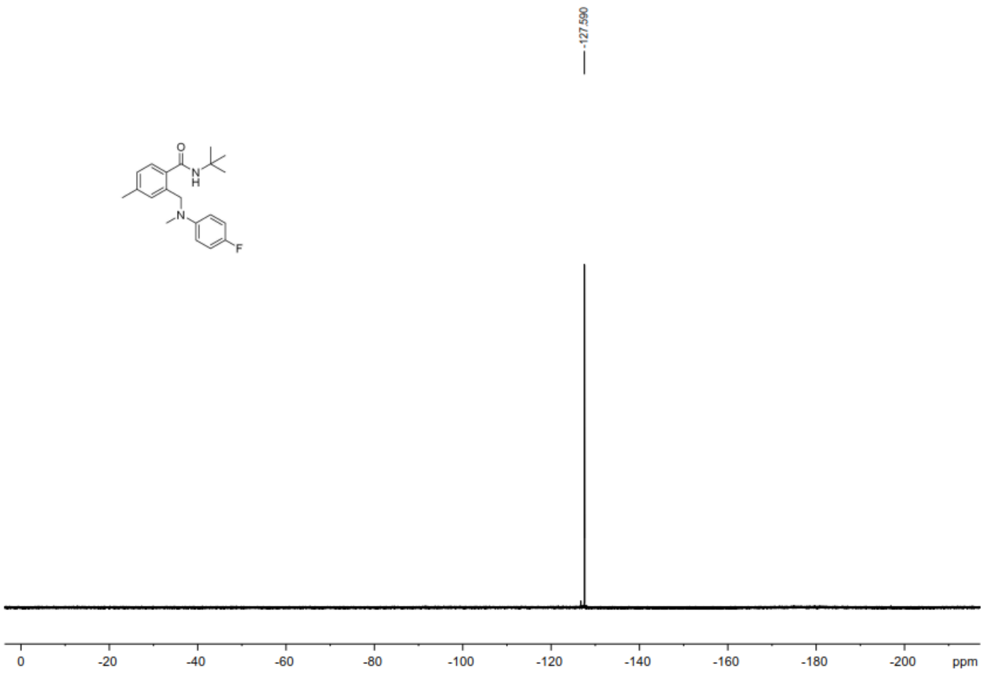

Supplementary Figure 63. ^{19}F NMR (282 MHz, $CDCl_3$) spectra for compound **3s.**

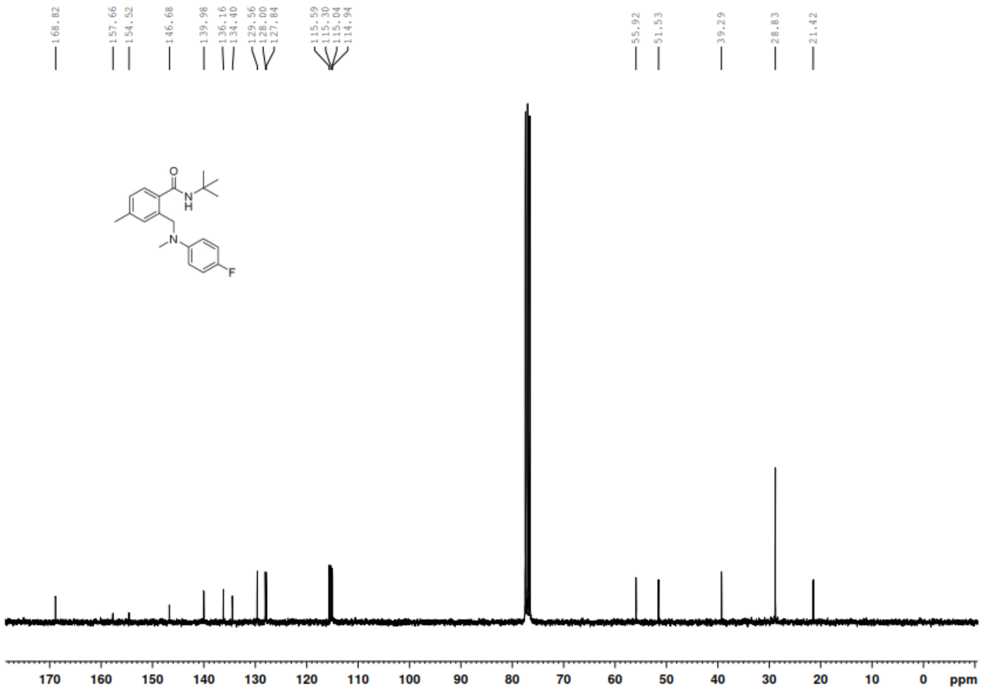

Supplementary Figure 64. ^{13}C NMR (75 MHz, $CDCl_3$) spectra for compound **3s.**

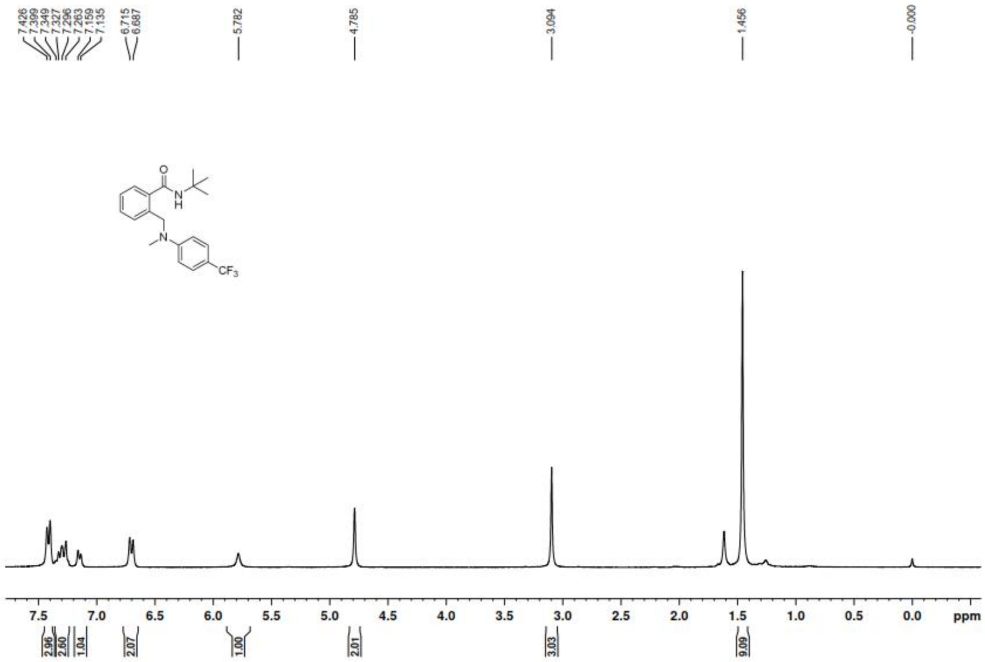

Supplementary Figure 65. ^{1}H NMR (300 MHz, $CDCl_3$) spectra for compound **3t.**

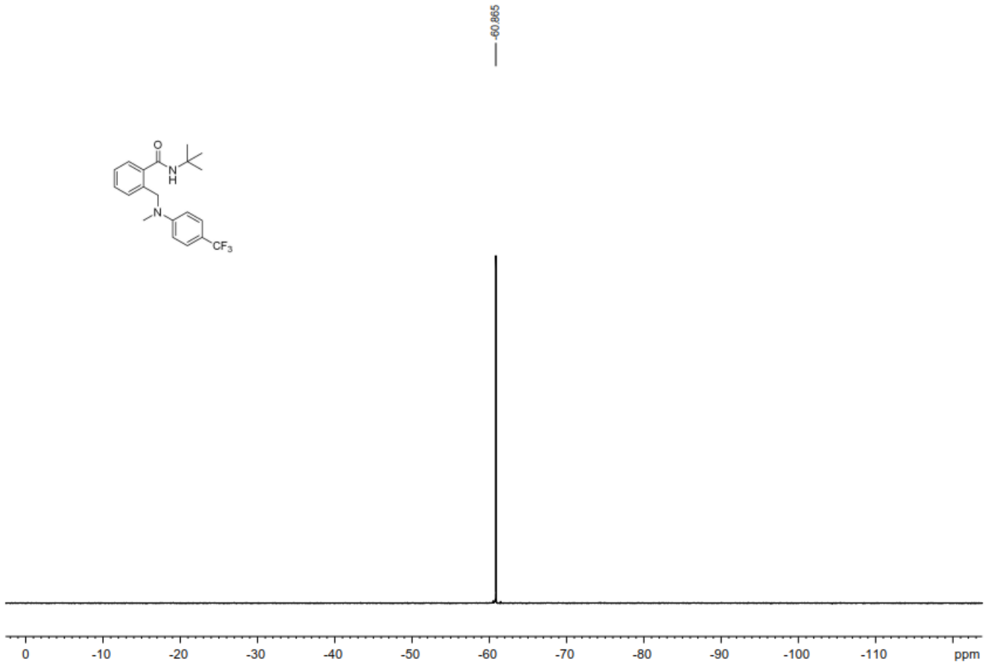

Supplementary Figure 66. ^{19}F NMR (282 MHz, $CDCl_3$) spectra for compound **3t.**

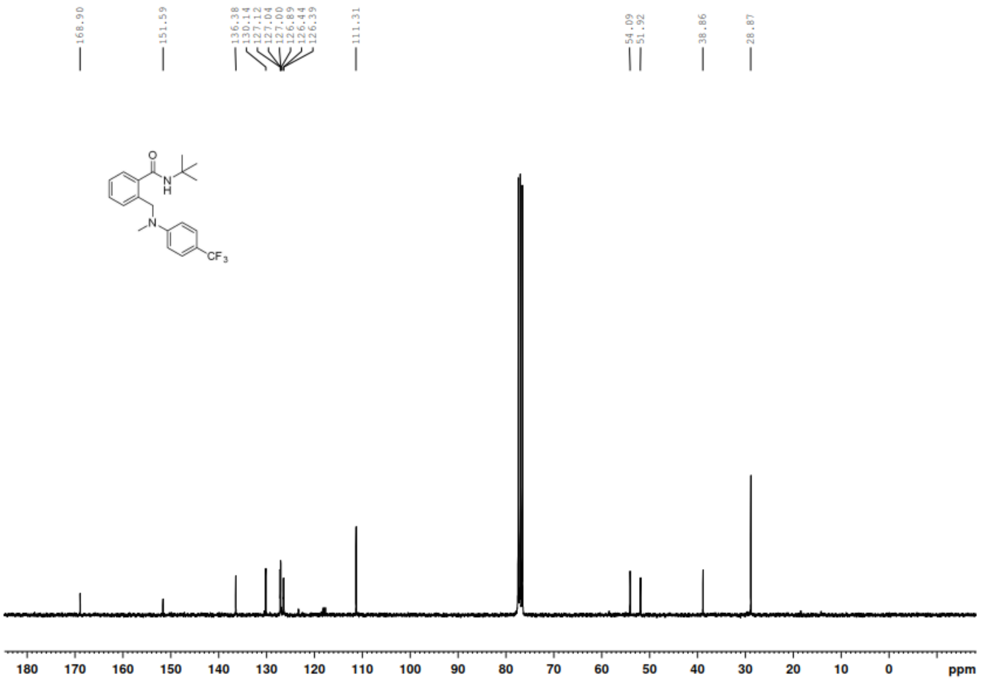

Supplementary Figure 67. ^{13}C NMR (75 MHz, $CDCl_3$) spectra for compound **3t.**

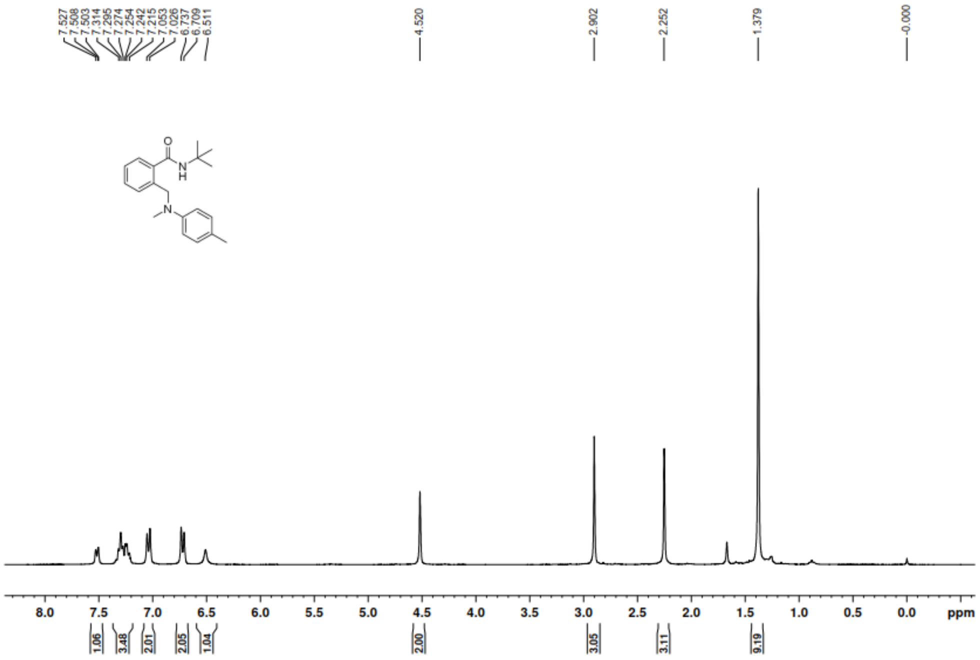

Supplementary Figure 68. ^{1}H NMR (300 MHz, $CDCl_3$) spectra for compound **3u.**

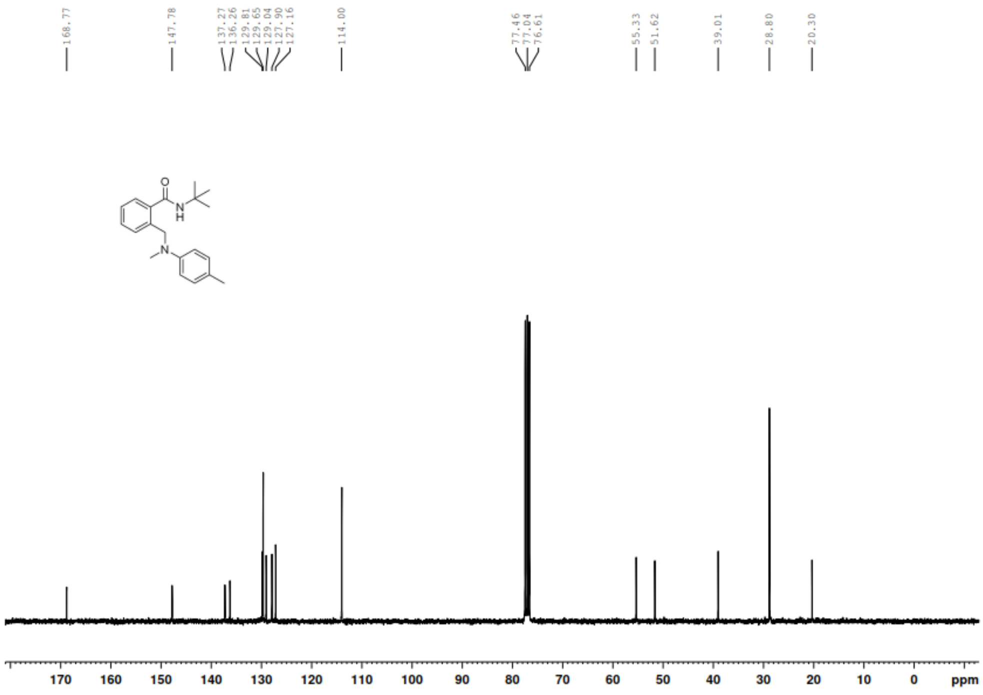

Supplementary Figure 69. ^{13}C NMR (75 MHz, $CDCl_3$) spectra for compound **3u.**

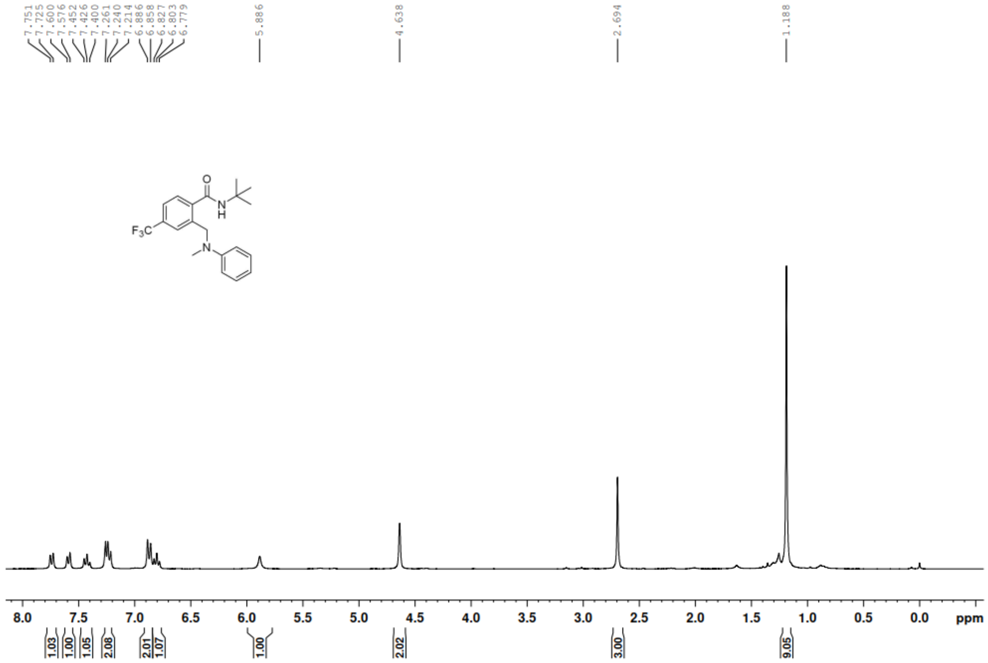

Supplementary Figure 70. ^{1}H NMR (300 MHz, $CDCl_3$) spectra for compound **3v.**

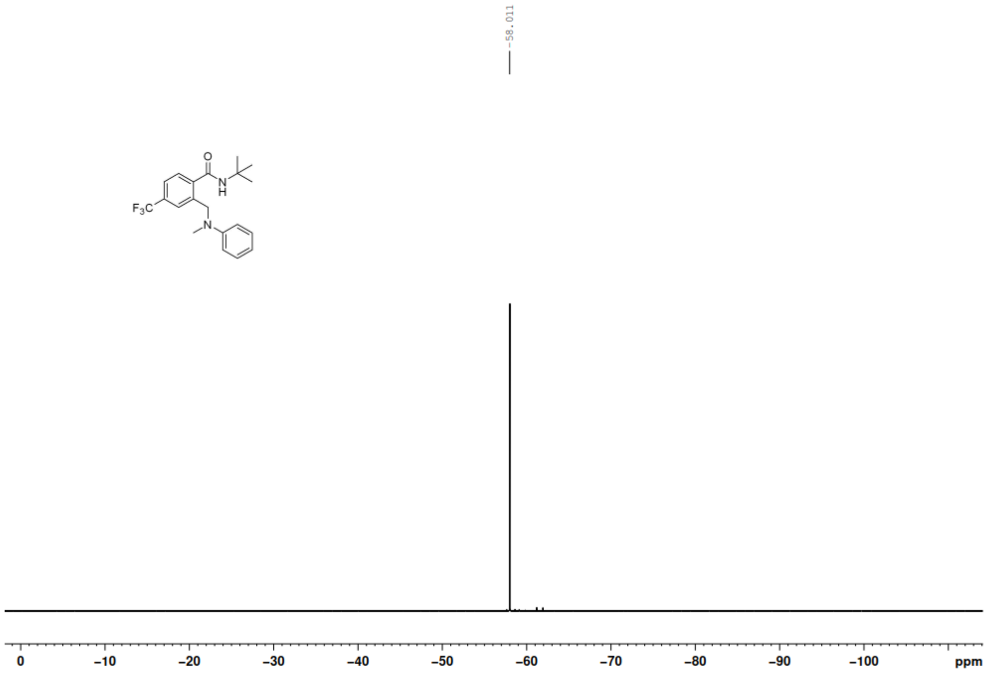

Supplementary Figure 71. ^{19}F NMR (282 MHz, $CDCl_3$) spectra for compound **3v.**

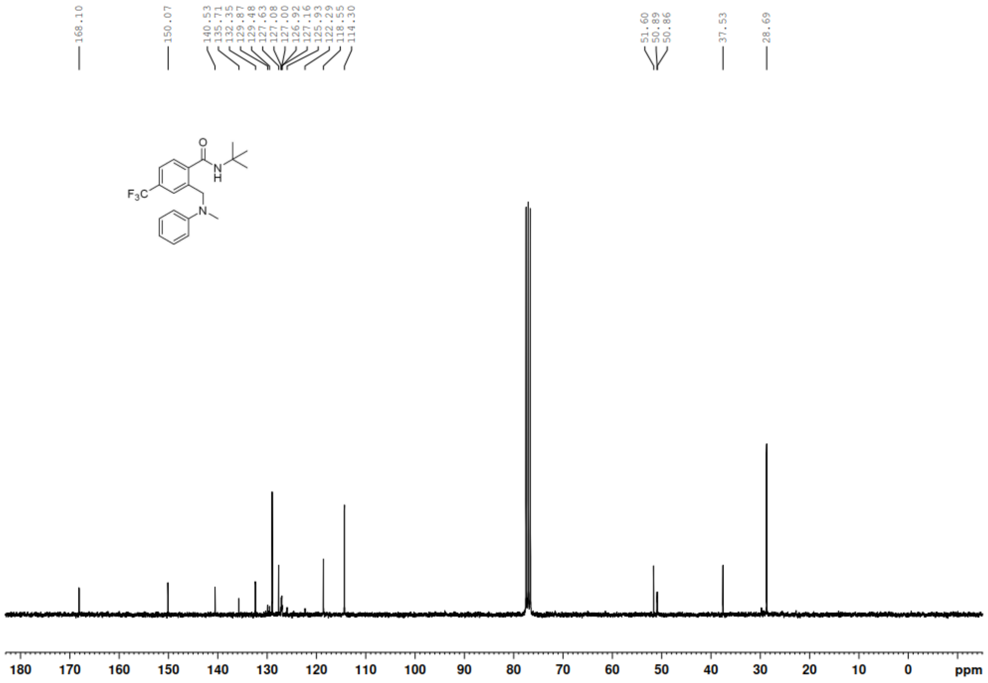

Supplementary Figure 72. ^{13}C NMR (75 MHz, $CDCl_3$) spectra for compound **3v.**

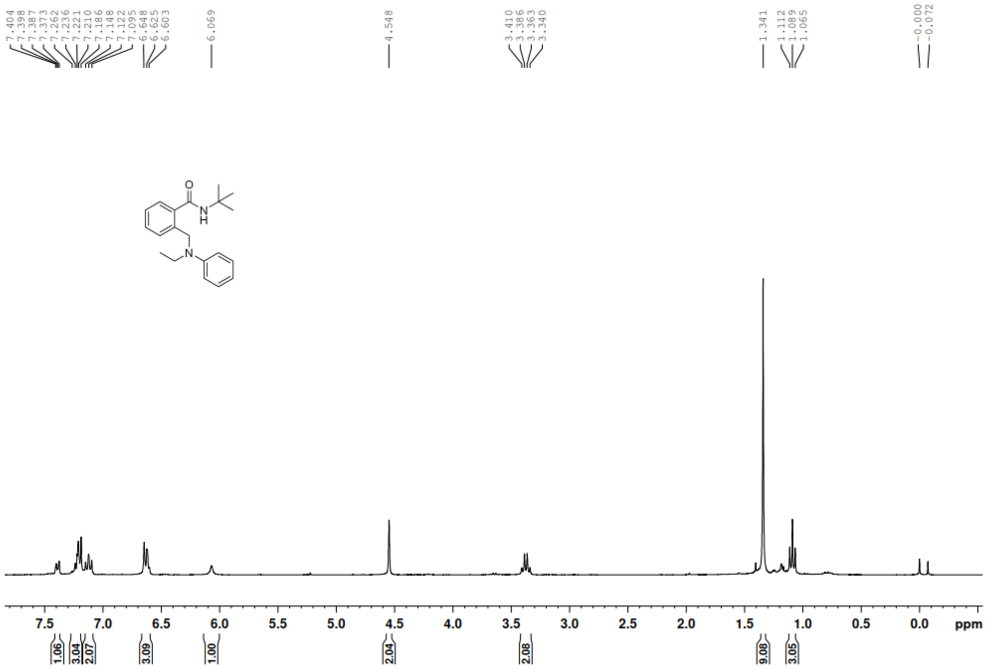

Supplementary Figure 73. ^{1}H NMR (300 MHz, $CDCl_3$) spectra for compound **3w.**

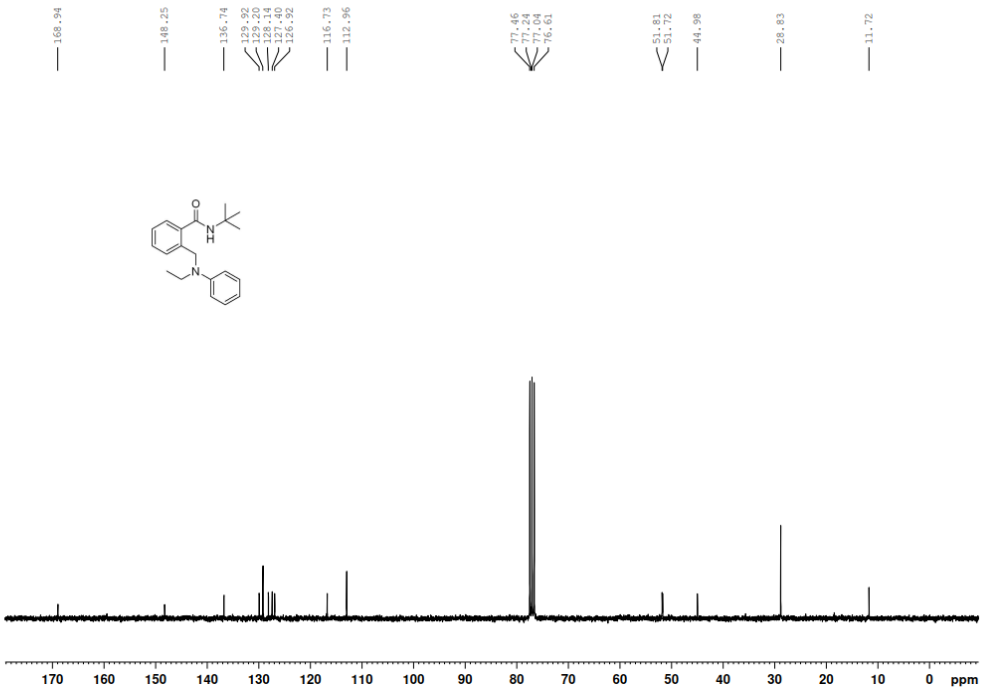

Supplementary Figure 74. ^{13}C NMR (75 MHz, $CDCl_3$) spectra for compound **3w.**

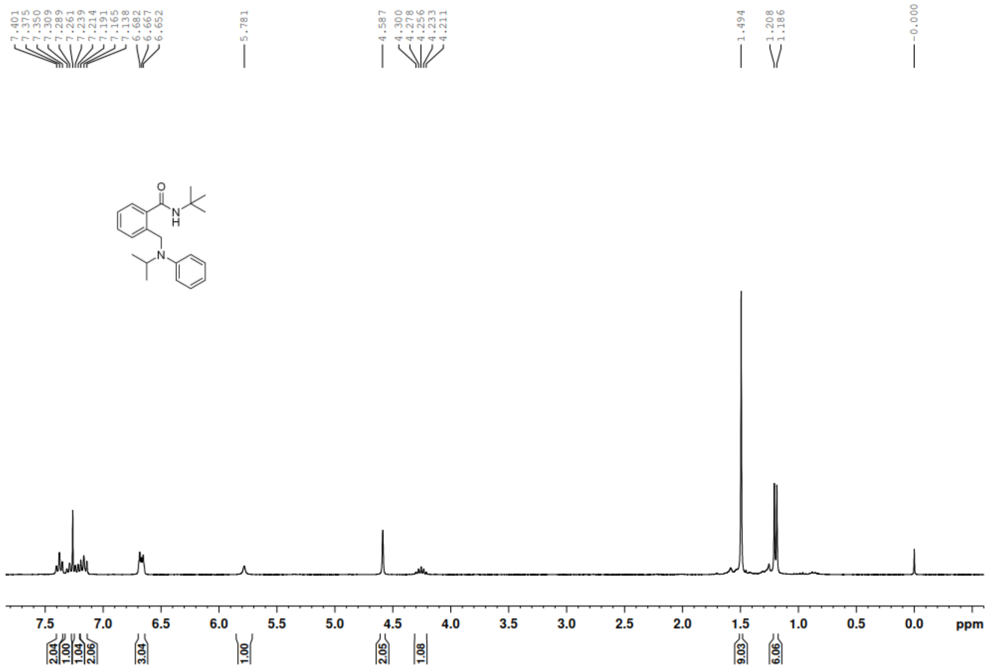

Supplementary Figure 75. ^{1}H NMR (300 MHz, $CDCl_3$) spectra for compound **3x.**

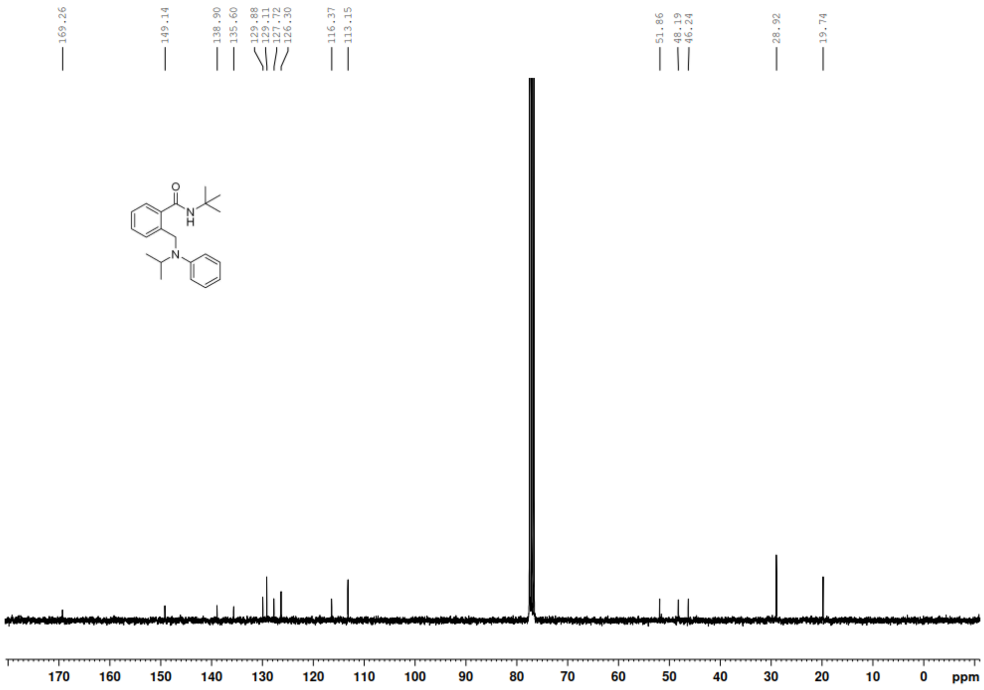

Supplementary Figure 76. ^{13}C NMR (75 MHz, $CDCl_3$) spectra for compound **3x.**

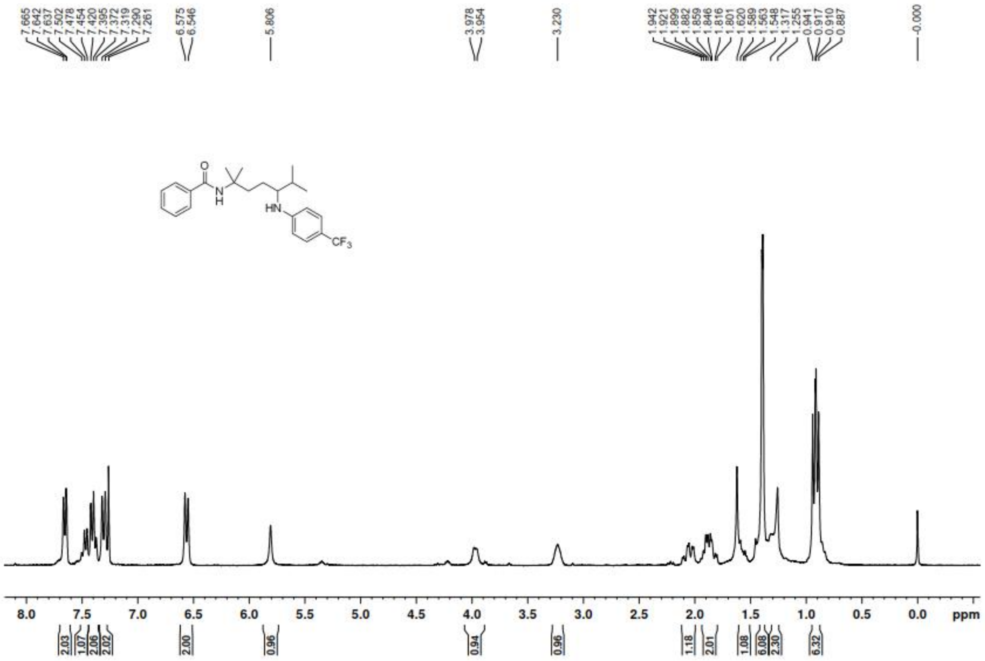

Supplementary Figure 77. ^{1}H NMR (300 MHz, $CDCl_3$) spectra for compound **3y.**

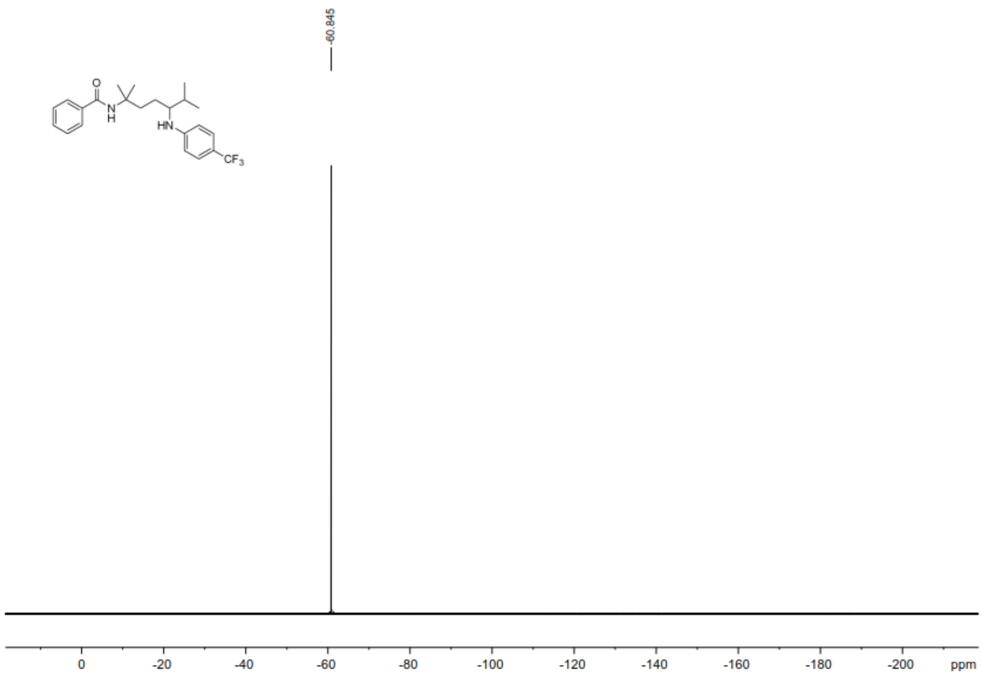

Supplementary Figure 78. ^{19}F NMR (282 MHz, $CDCl_3$) spectra for compound **3y.**

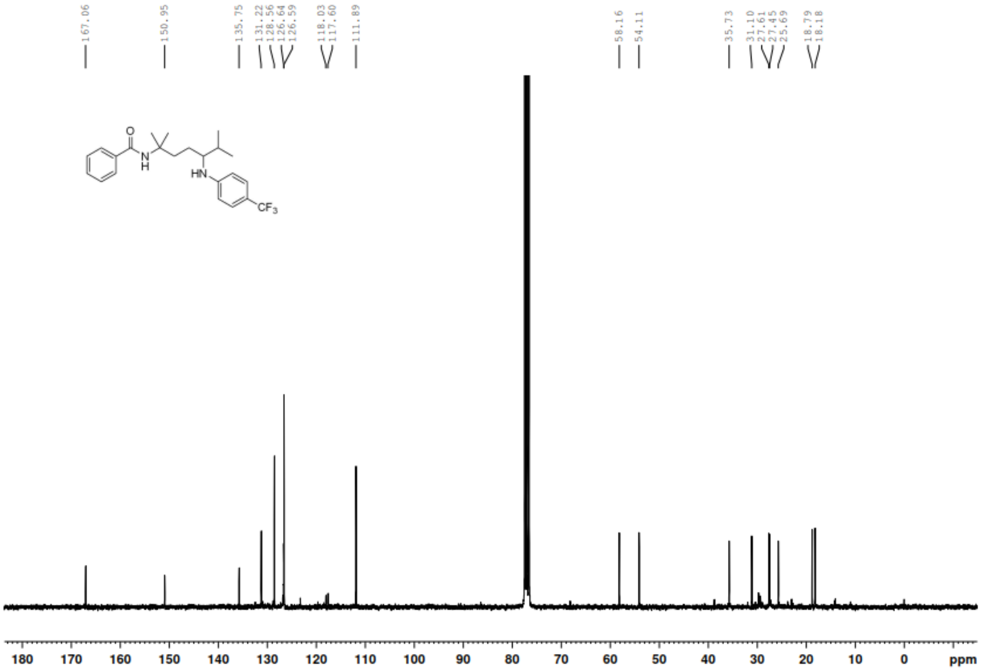

Supplementary Figure 79. ^{13}C NMR (75 MHz, $CDCl_3$) spectra for compound **3y.**

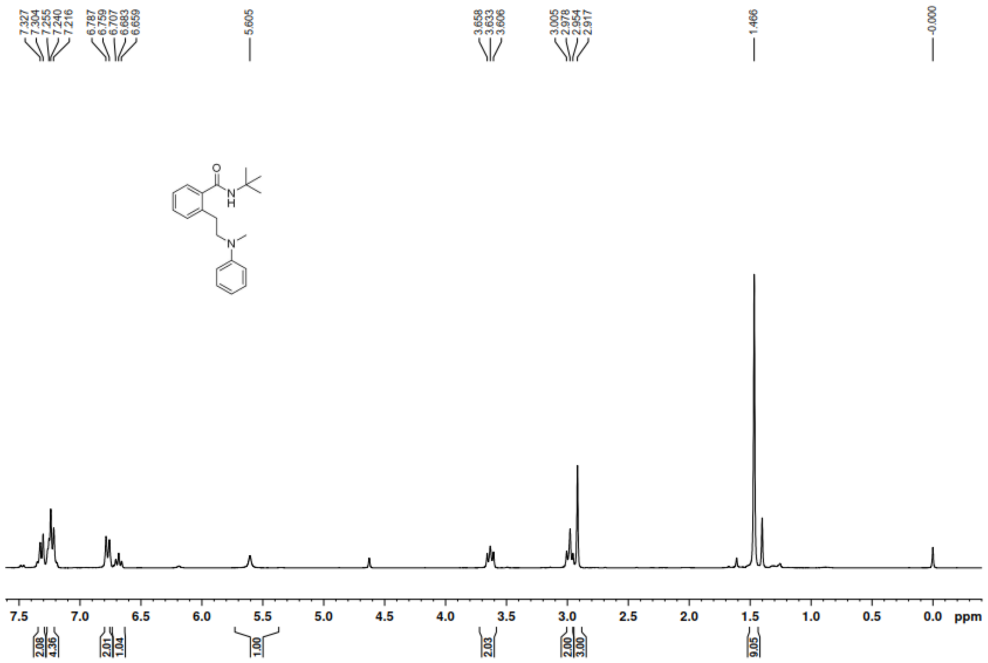

Supplementary Figure 80. ^{1}H NMR (300 MHz, $CDCl_3$) spectra for compound **4a.**

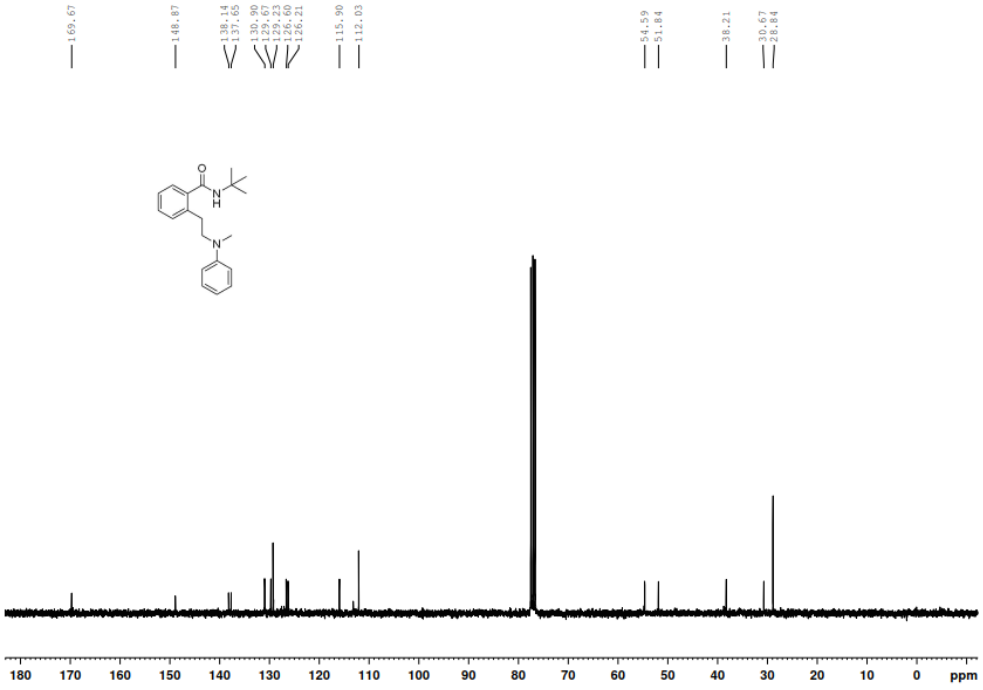

Supplementary Figure 81. ^{13}C NMR (75 MHz, $CDCl_3$) spectra for compound **4a.**

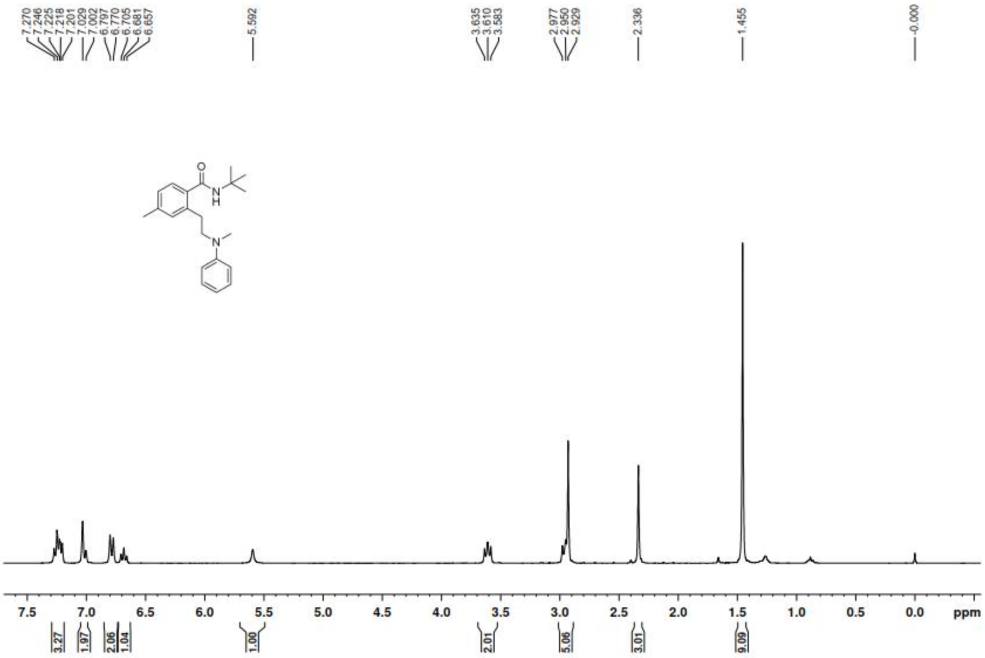

Supplementary Figure 82. ^{1}H NMR (300 MHz, $CDCl_3$) spectra for compound **4b.**

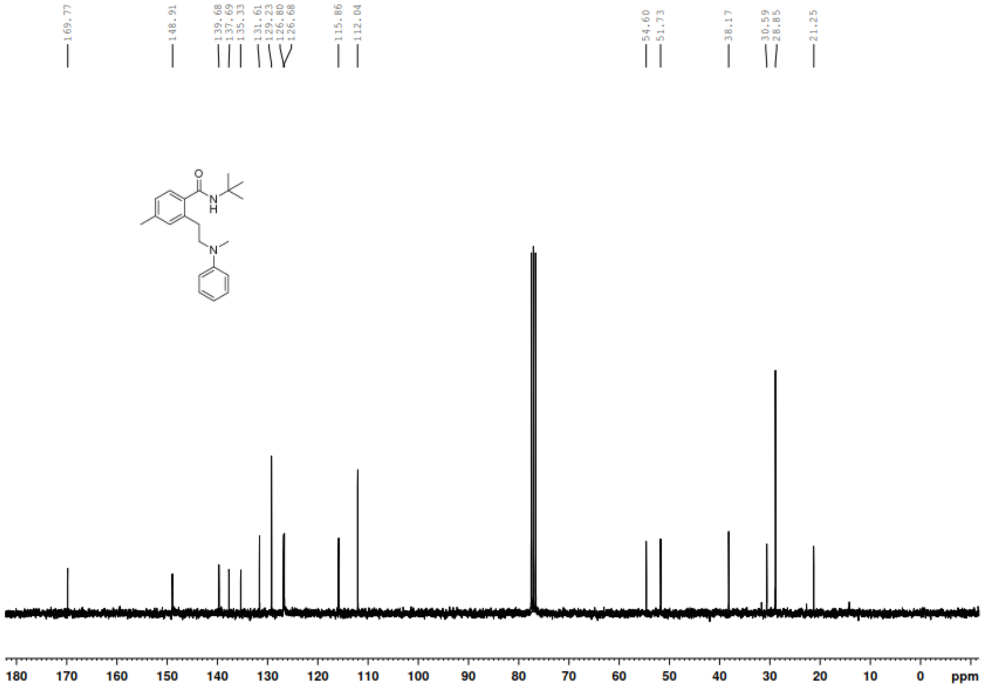

Supplementary Figure 83. ^{13}C NMR (75 MHz, $CDCl_3$) spectra for compound **4b.**

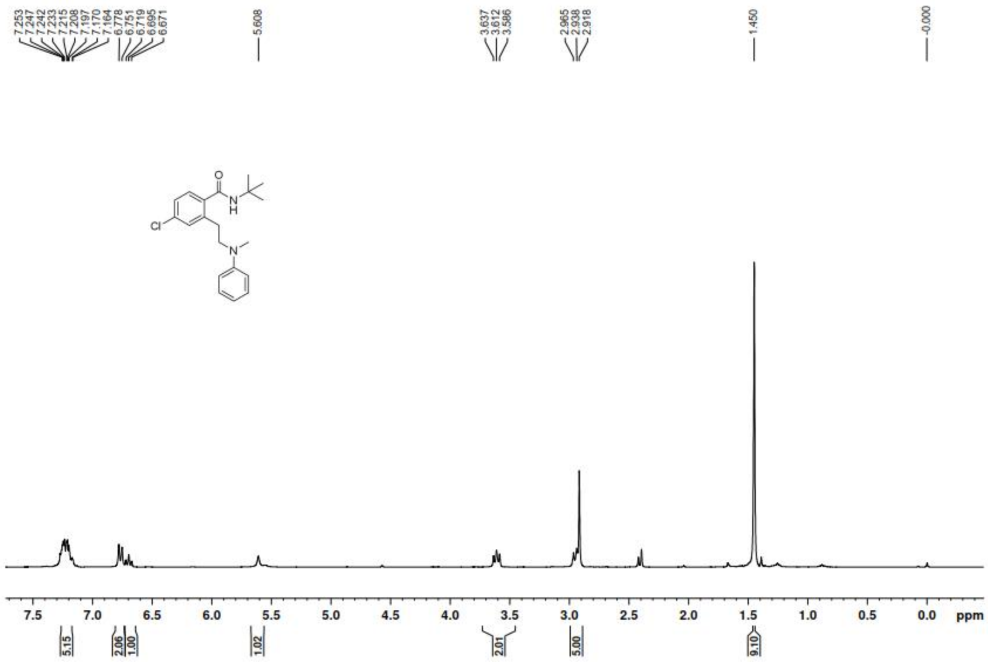

Supplementary Figure 84. ^{1}H NMR (300 MHz, $CDCl_3$) spectra for compound **4c.**

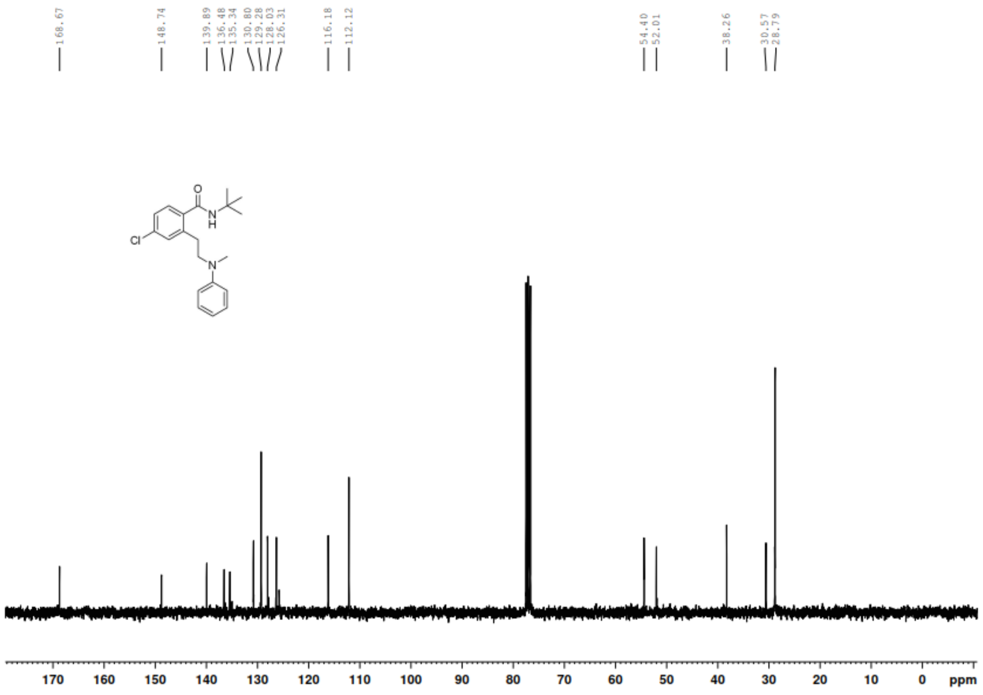

Supplementary Figure 85. ^{13}C NMR (75 MHz, $CDCl_3$) spectra for compound **4c.**

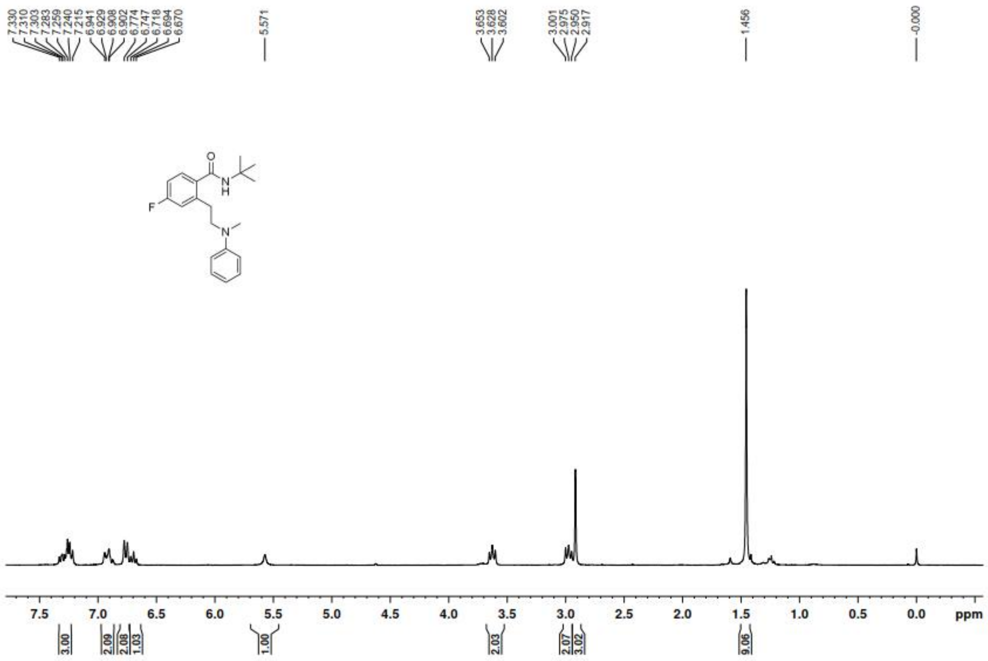

Supplementary Figure 86. ^{1}H NMR (300 MHz, $CDCl_3$) spectra for compound **4d.**

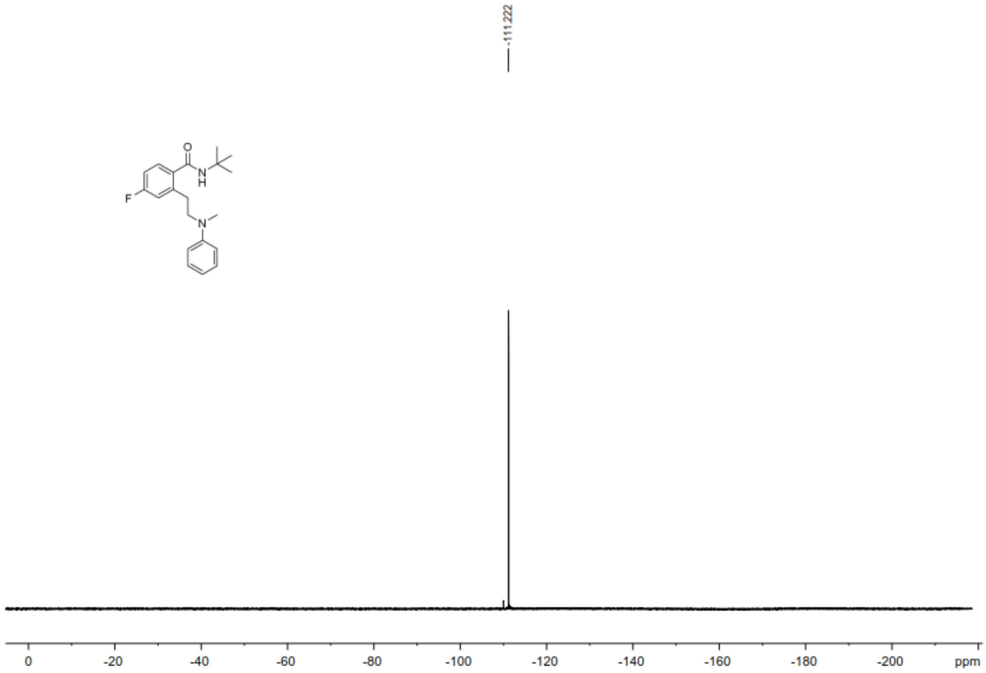

Supplementary Figure 87. ^{19}F NMR (282 MHz, $CDCl_3$) spectra for compound **4d.**

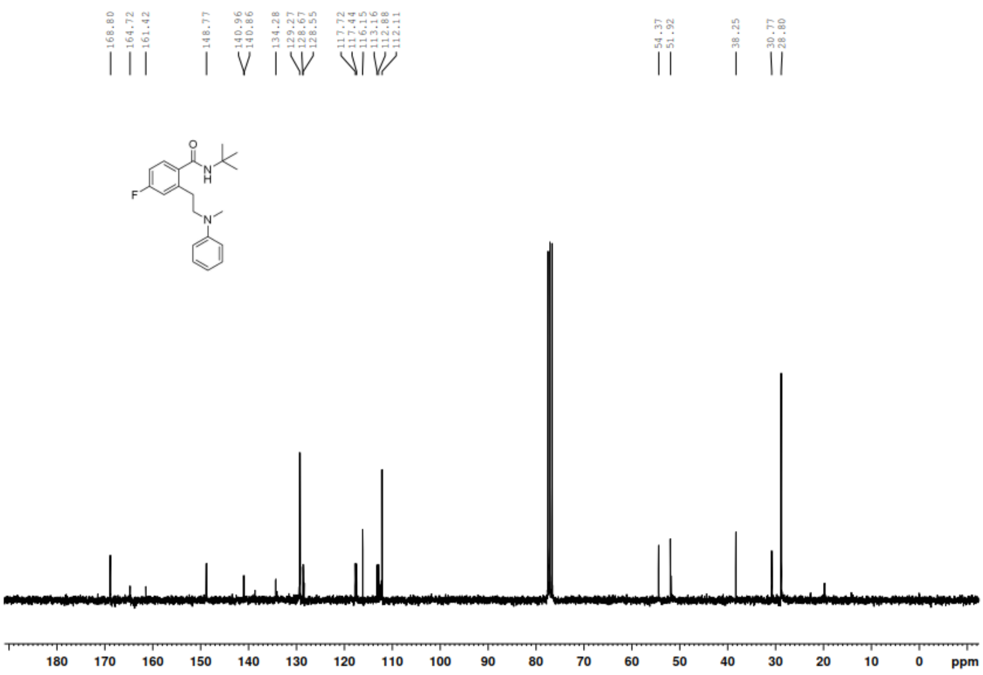

Supplementary Figure 88. ^{13}C NMR (75 MHz, $CDCl_3$) spectra for compound **4d.**

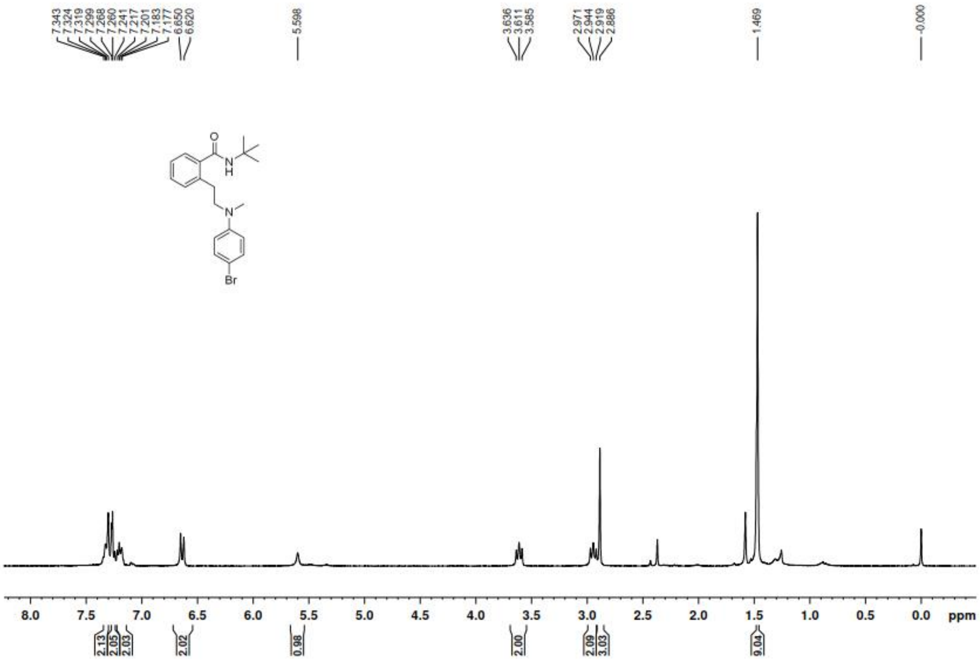

Supplementary Figure 89. ^{1}H NMR (300 MHz, $CDCl_3$) spectra for compound **4e.**

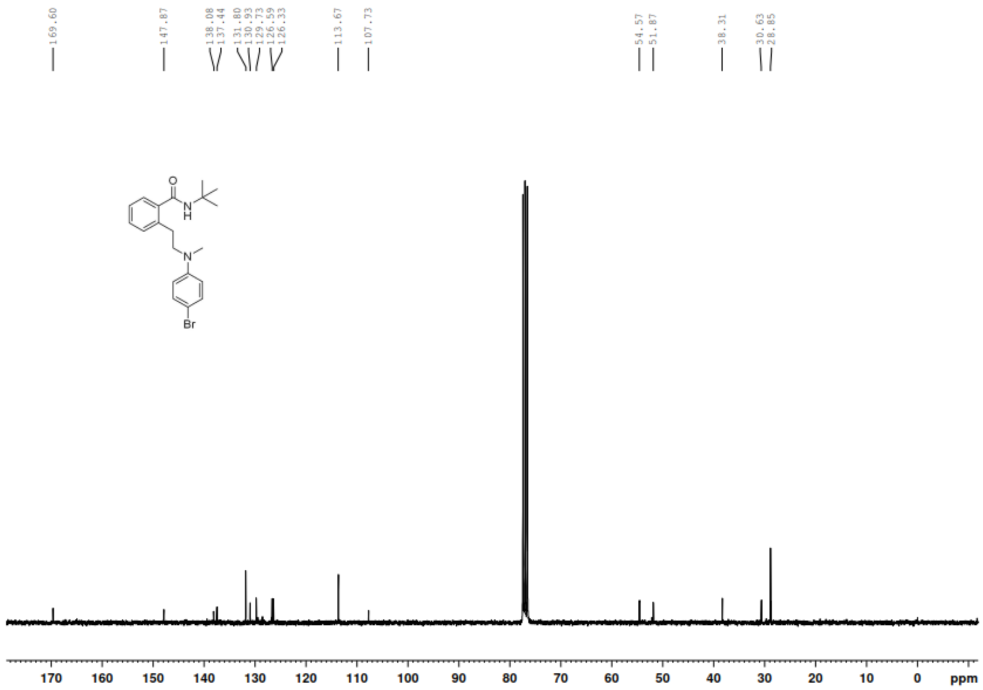

Supplementary Figure 90. ^{13}C NMR (75 MHz, $CDCl_3$) spectra for compound **4e.**

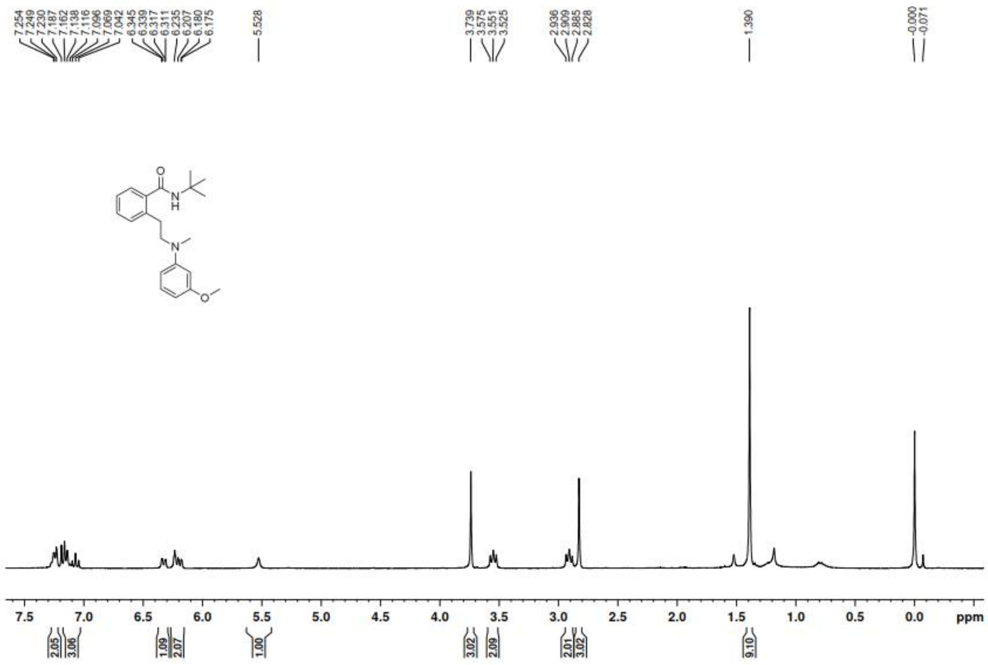

Supplementary Figure 91. ^{1}H NMR (300 MHz, $CDCl_3$) spectra for compound **4f.**

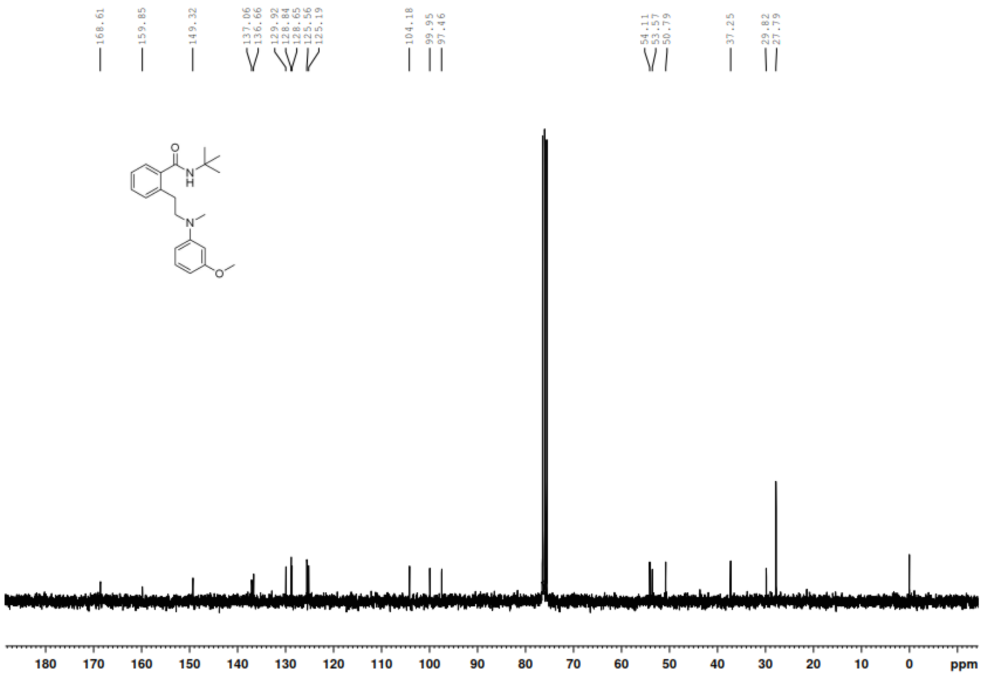

Supplementary Figure 92. ^{13}C NMR (75 MHz, $CDCl_3$) spectra for compound **4f.**

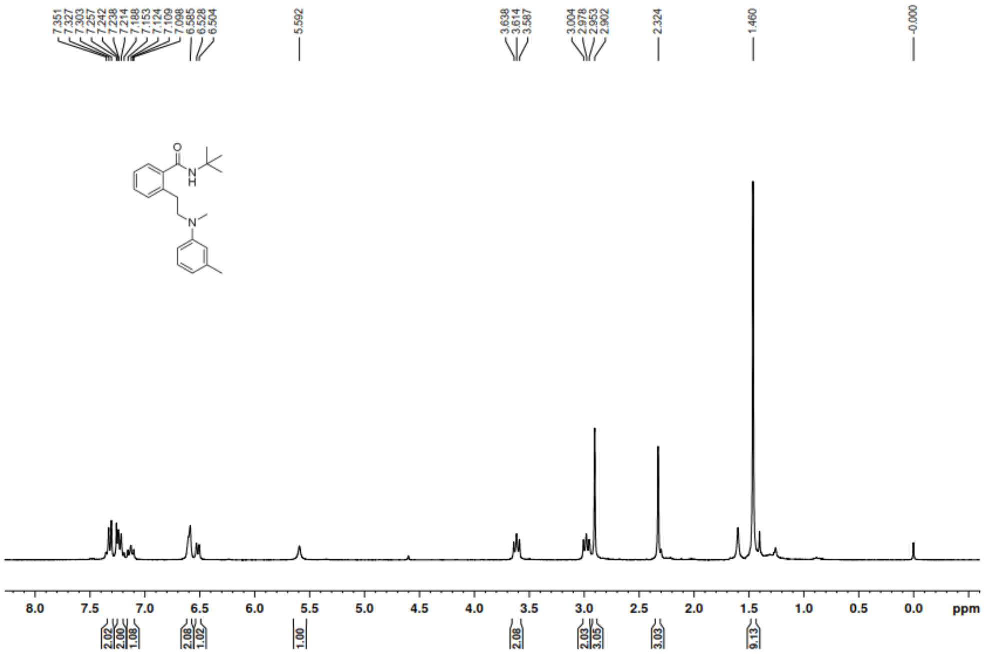

Supplementary Figure 93. ^{1}H NMR (300 MHz, $CDCl_3$) spectra for compound **4g.**

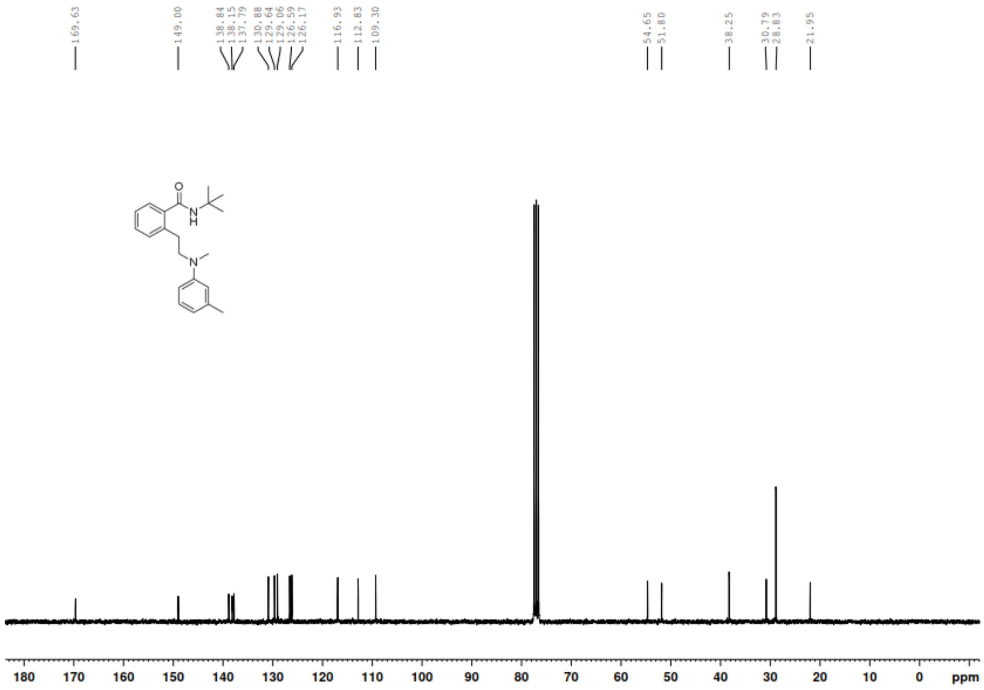

Supplementary Figure 94. ^{13}C NMR (75 MHz, $CDCl_3$) spectra for compound **4g.**

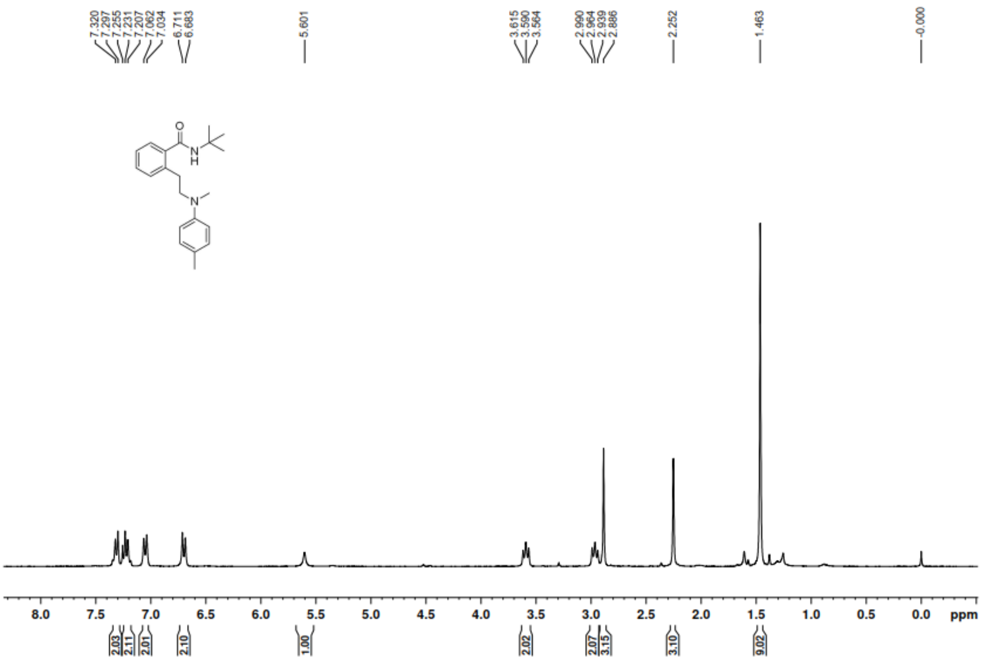

Supplementary Figure 95. ^{1}H NMR (300 MHz, $CDCl_3$) spectra for compound **4h.**

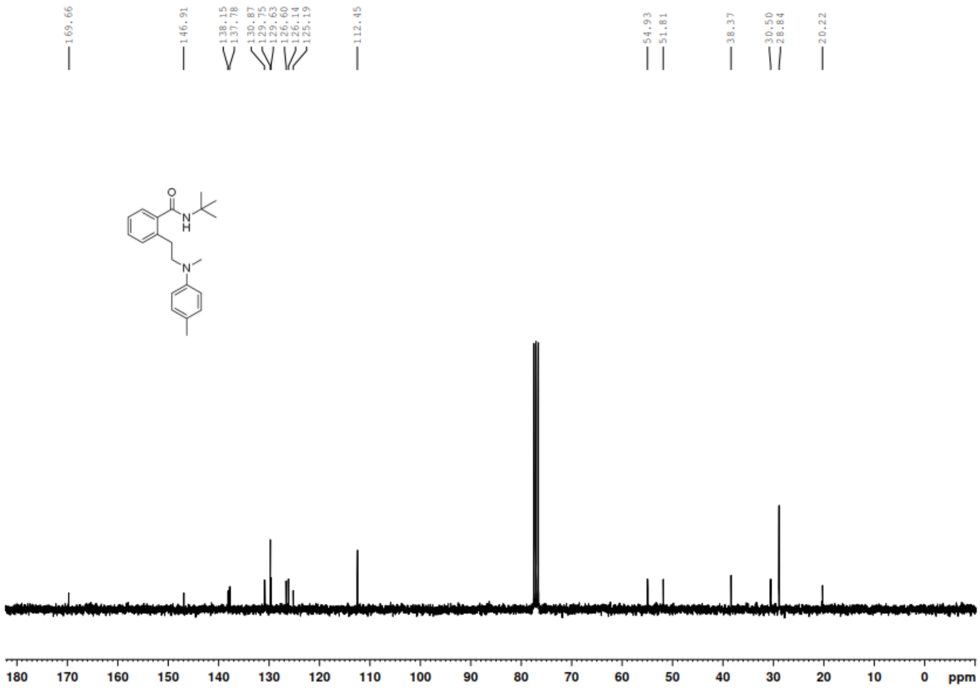

Supplementary Figure 96. ^{13}C NMR (75 MHz, $CDCl_3$) spectra for compound **4h.**

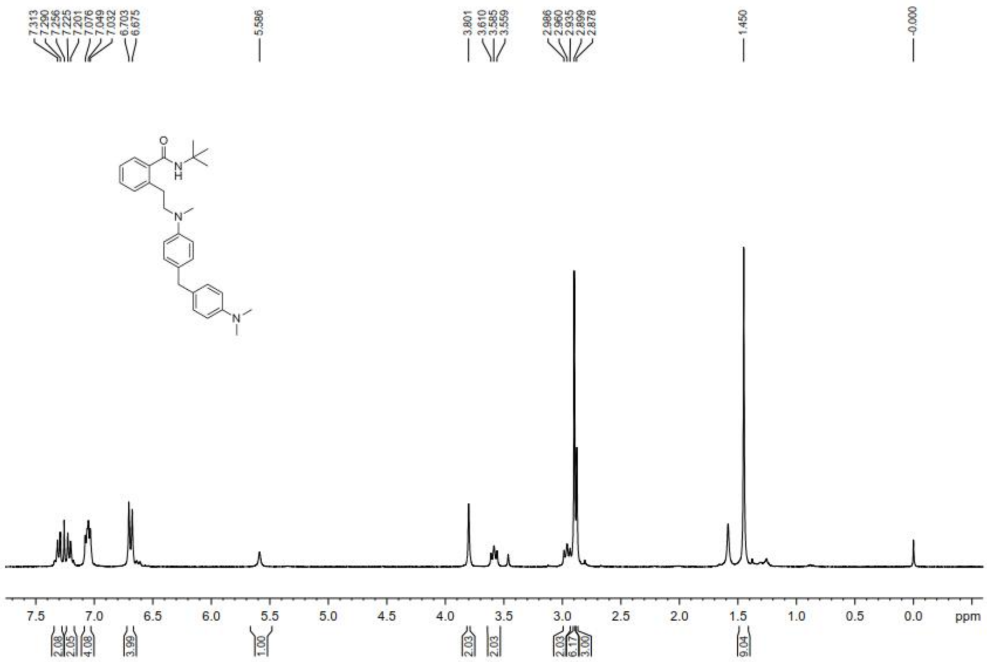

Supplementary Figure 97. ^{1}H NMR (300 MHz, $CDCl_3$) spectra for compound **4i.**

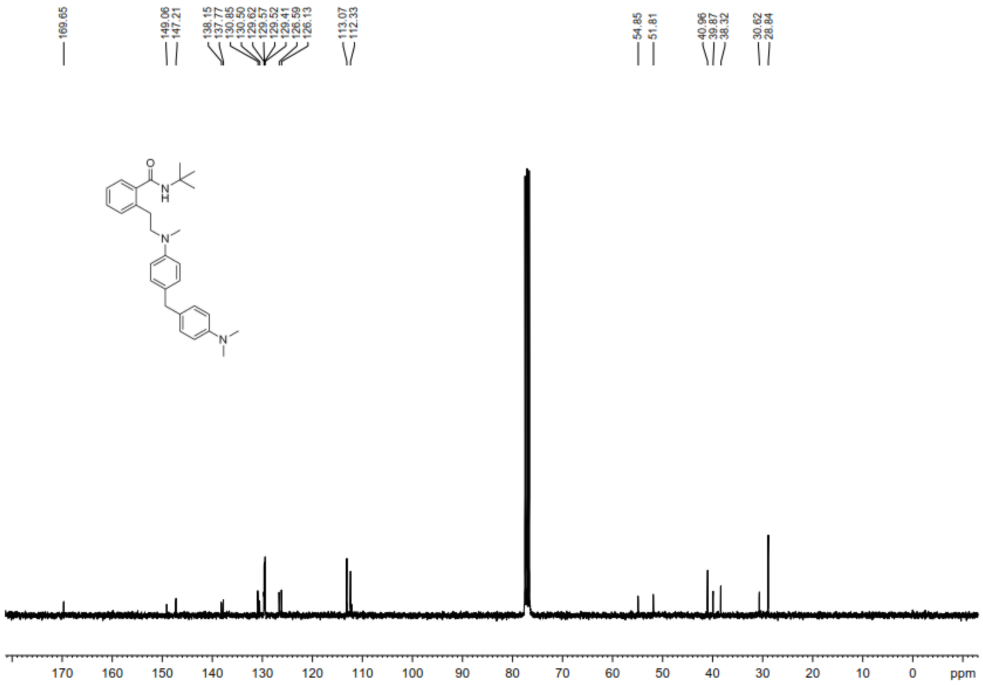

Supplementary Figure 98. ^{13}C NMR (75 MHz, $CDCl_3$) spectra for compound **4i.**

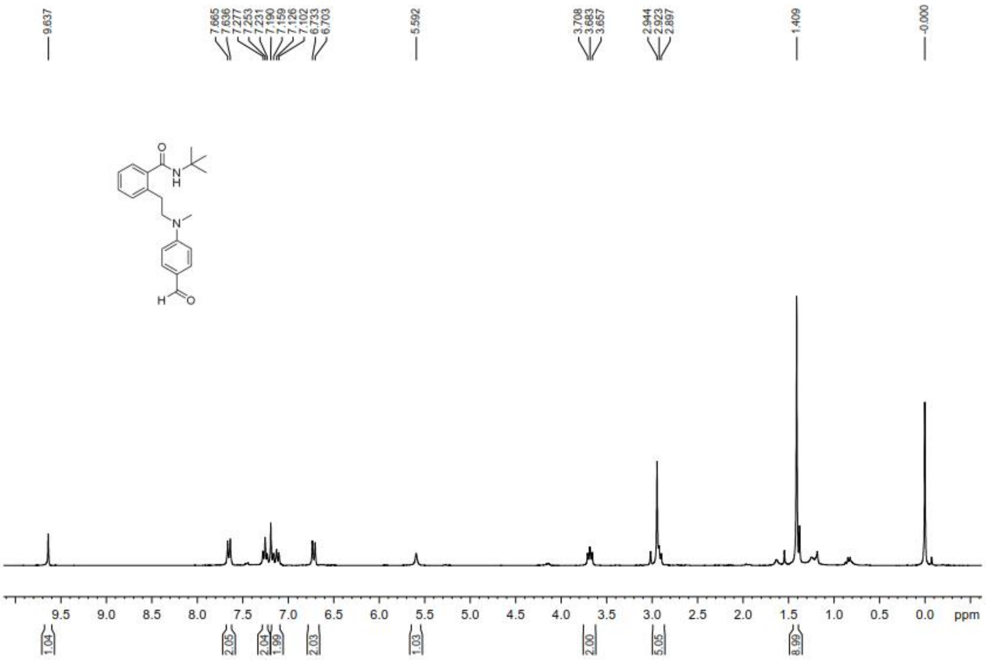

Supplementary Figure 99. ^{1}H NMR (300 MHz, $CDCl_3$) spectra for compound **4j.**

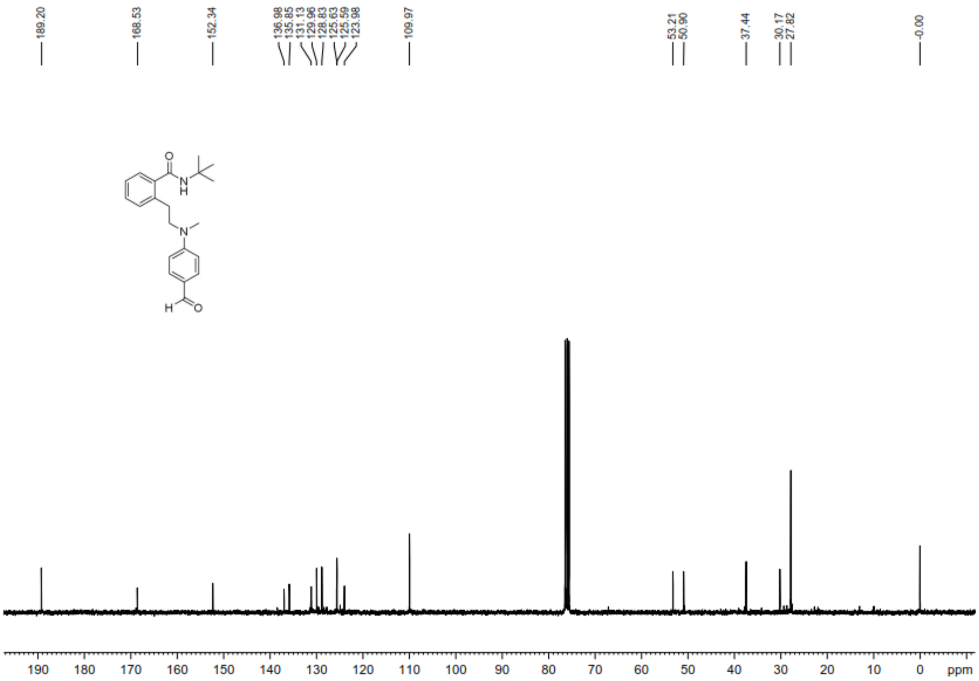

Supplementary Figure 100. ^{13}C NMR (75 MHz, $CDCl_3$) spectra for compound **4j.**

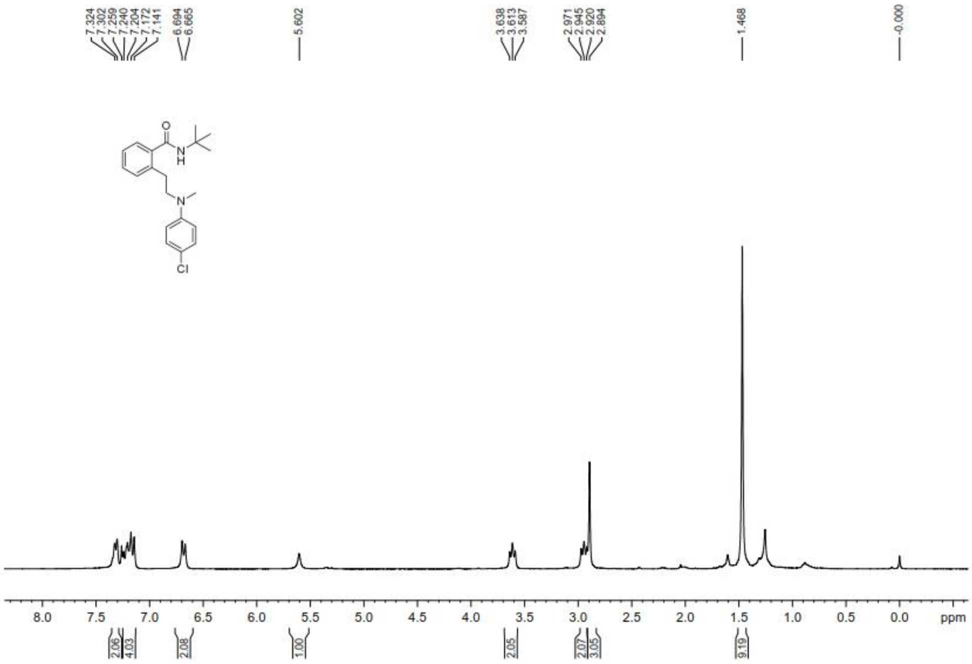

Supplementary Figure 101. ^{1}H NMR (300 MHz, $CDCl_3$) spectra for compound **4k.**

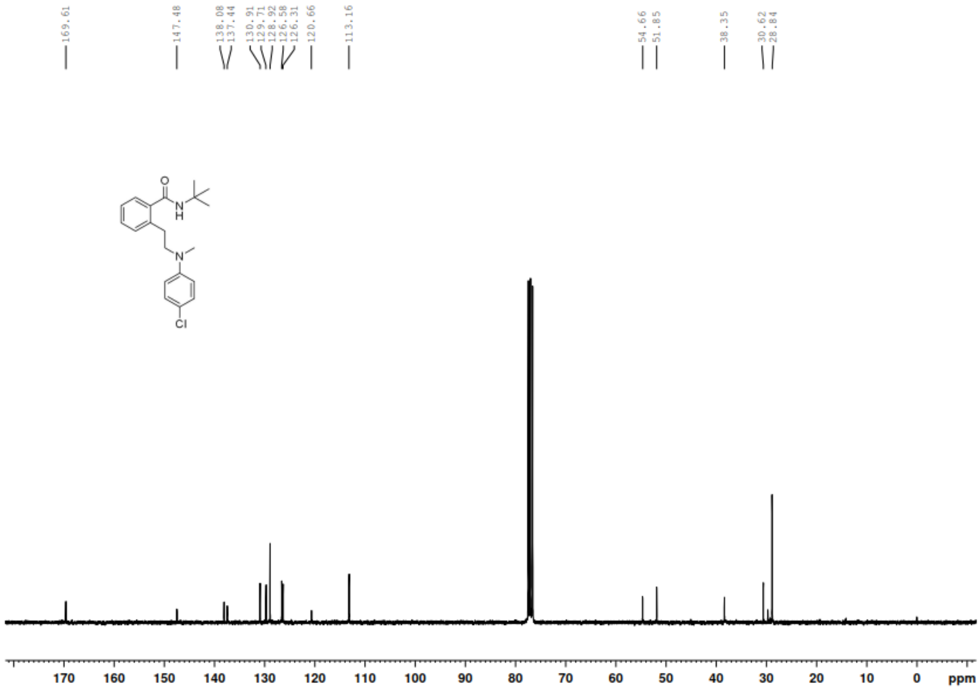

Supplementary Figure 102. ^{13}C NMR (75 MHz, $CDCl_3$) spectra for compound **4k.**

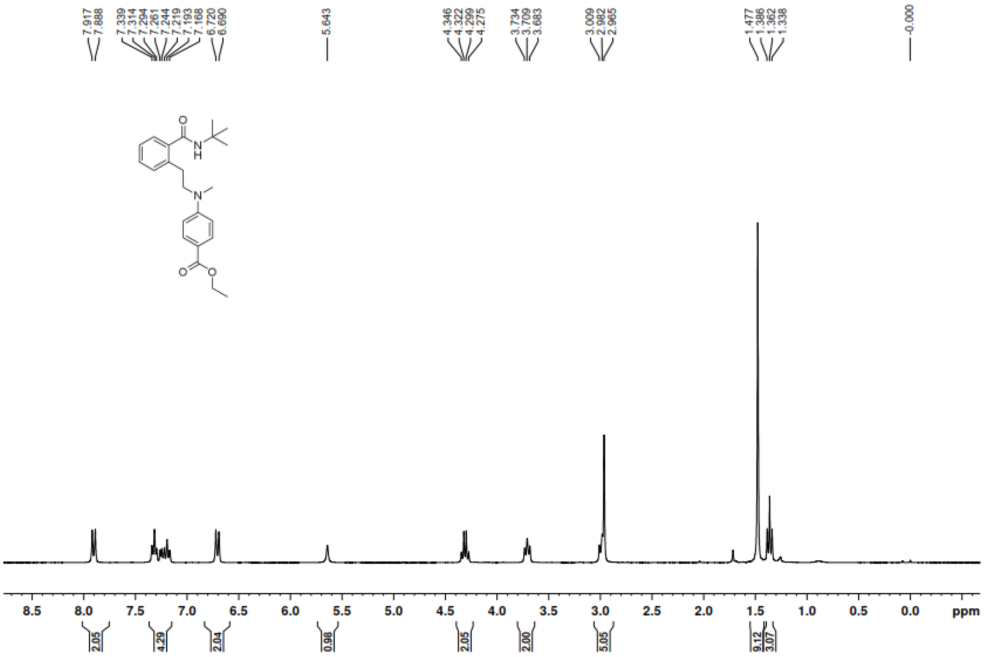

Supplementary Figure 103. ^{1}H NMR (300 MHz, $CDCl_3$) spectra for compound **4l.**

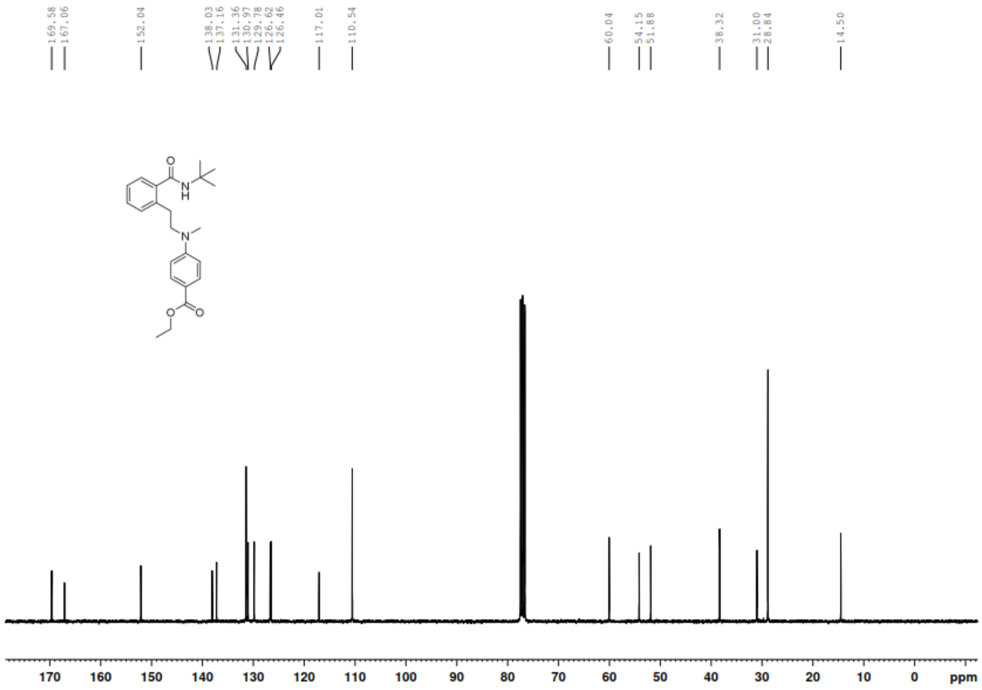

Supplementary Figure 104. ^{13}C NMR (75 MHz, $CDCl_3$) spectra for compound **4l.**

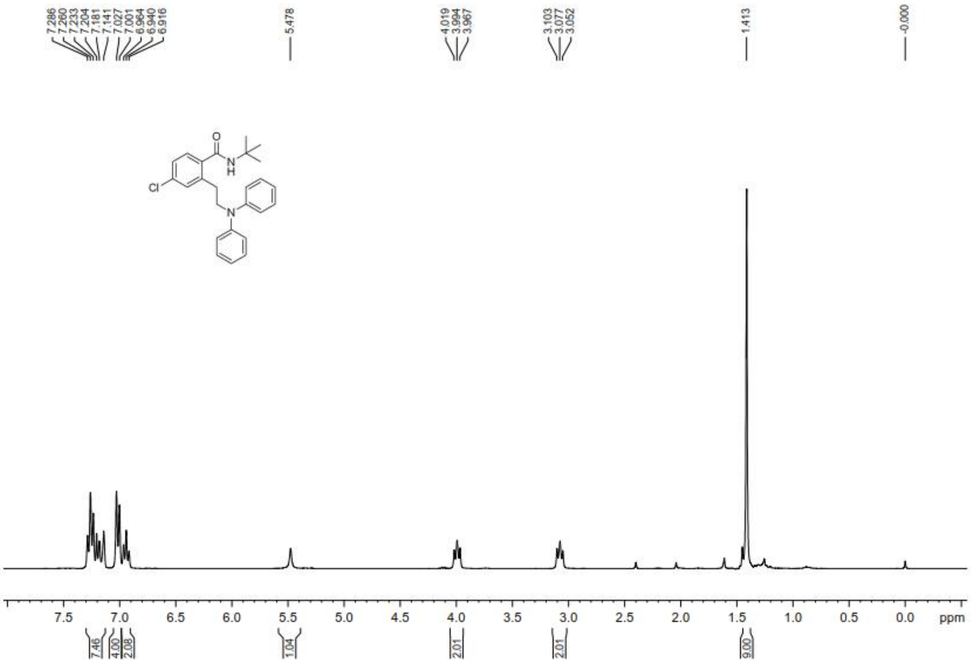

Supplementary Figure 105. ^{1}H NMR (300 MHz, $CDCl_3$) spectra for compound **4m.**

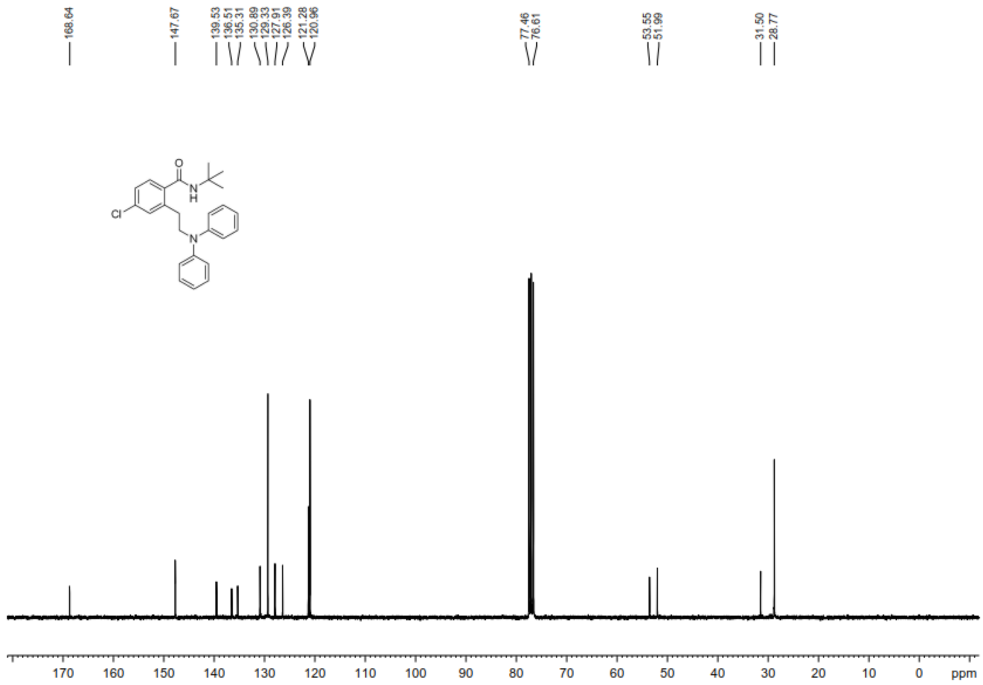

Supplementary Figure 106. ^{13}C NMR (75 MHz, $CDCl_3$) spectra for compound **4m.**

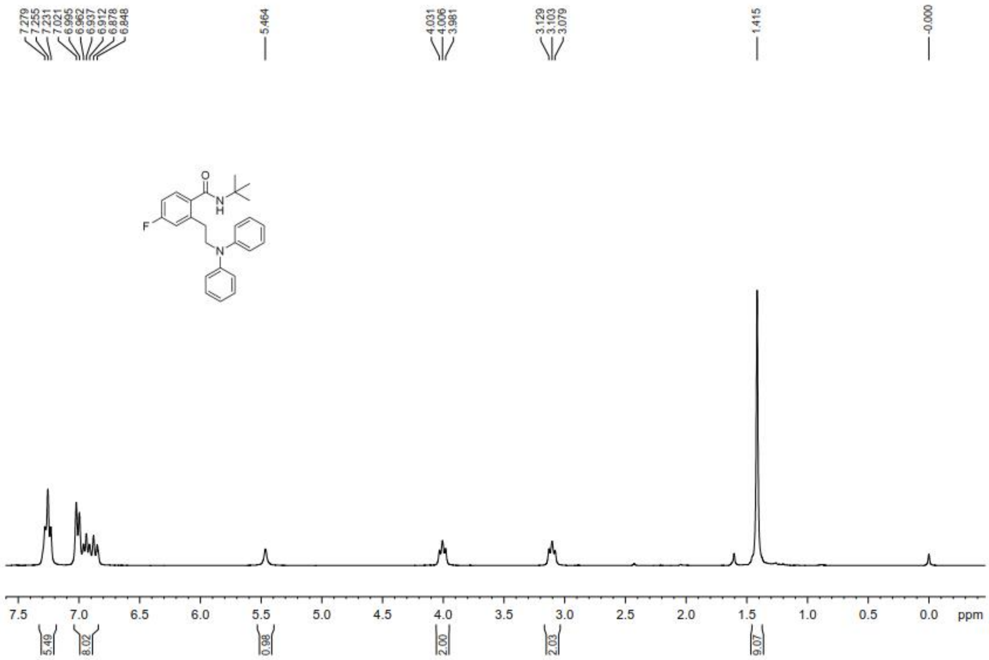

Supplementary Figure 107. ^{1}H NMR (300 MHz, $CDCl_3$) spectra for compound **4n.**

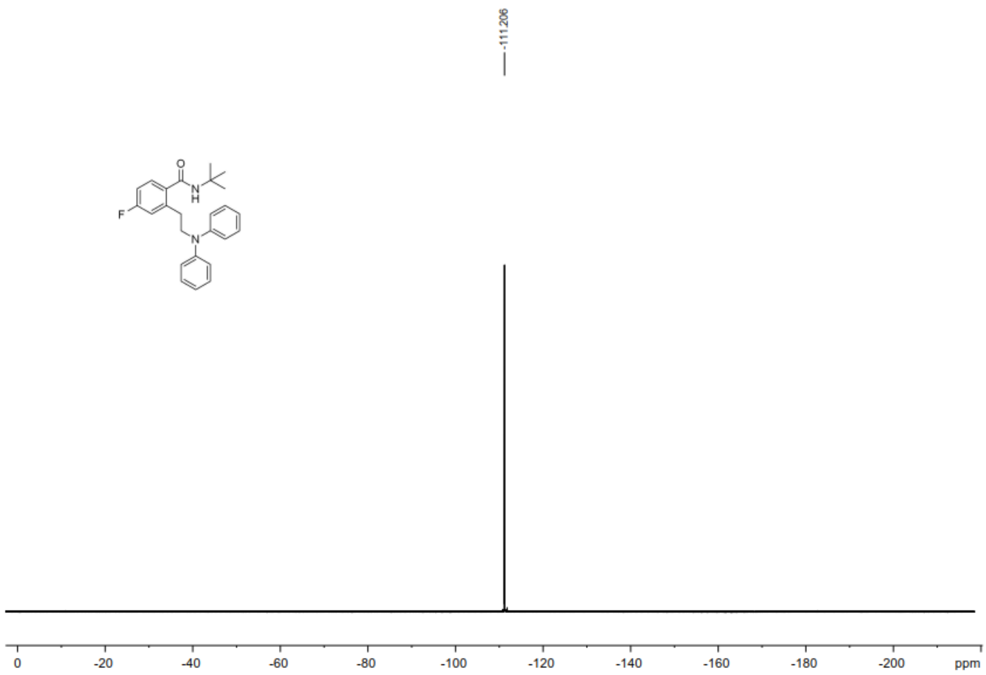

Supplementary Figure 108. ^{19}F NMR (282 MHz, $CDCl_3$) spectra for compound **4n.**

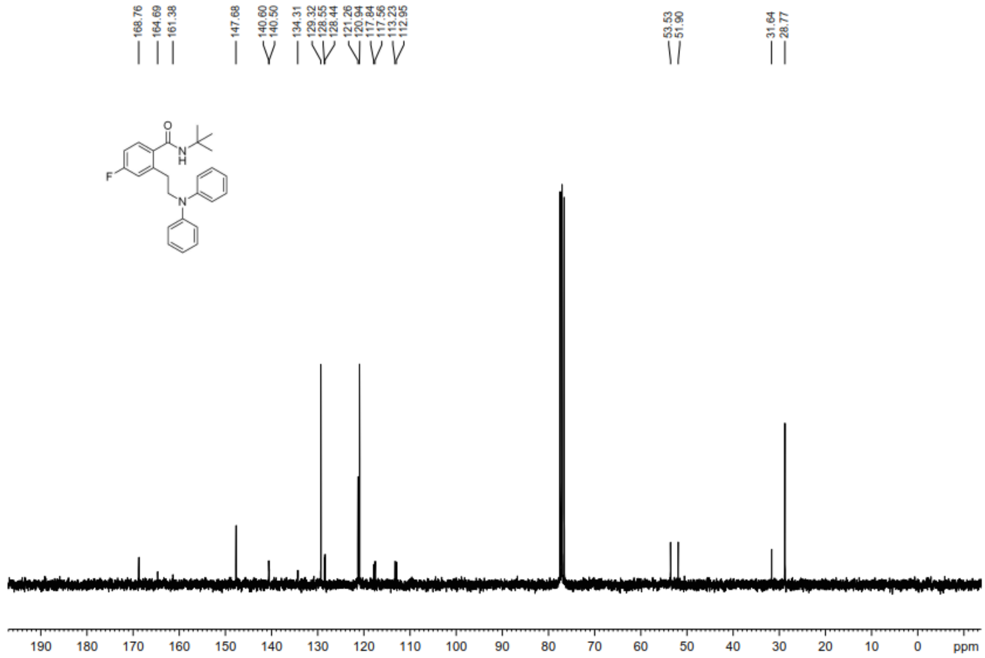

Supplementary Figure 109. ^{13}C NMR (75 MHz, $CDCl_3$) spectra for compound **4n.**

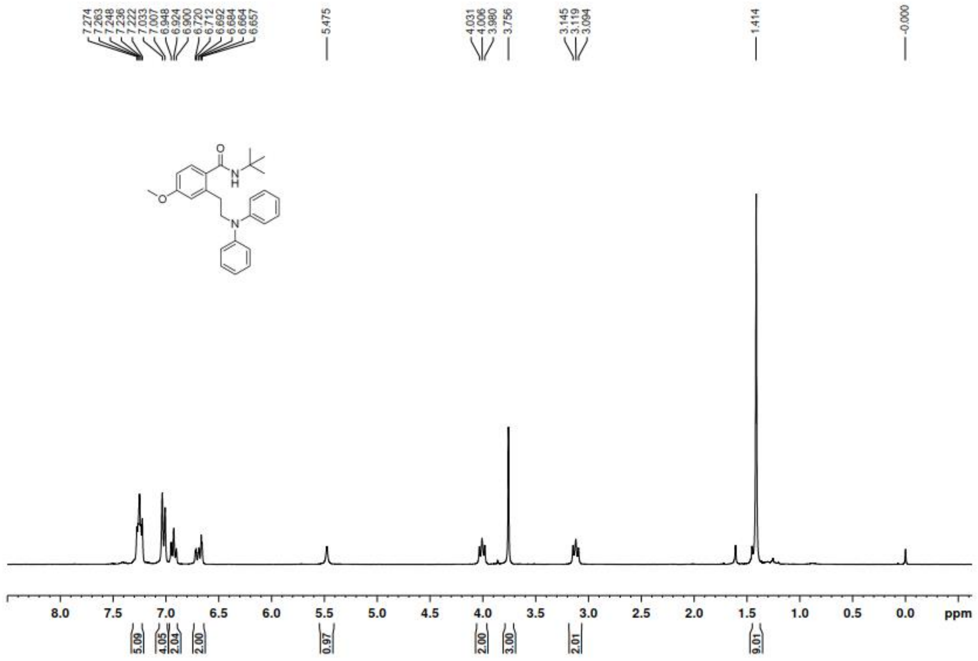

Supplementary Figure 110. ^{1}H NMR (300 MHz, $CDCl_3$) spectra for compound **4o.**

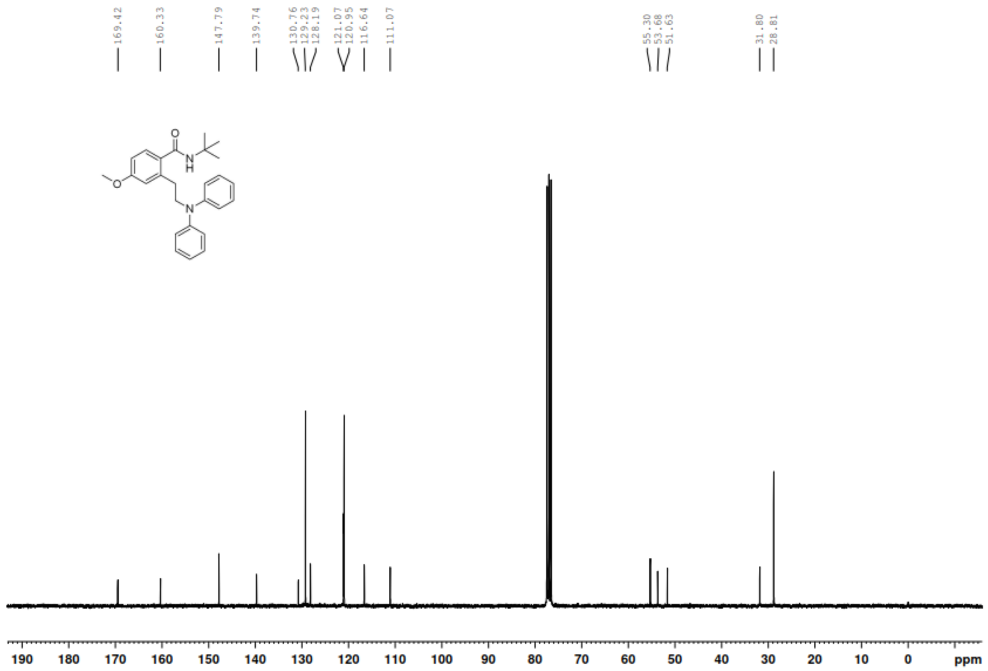

Supplementary Figure 111. ^{13}C NMR (75 MHz, $CDCl_3$) spectra for compound **4o.**

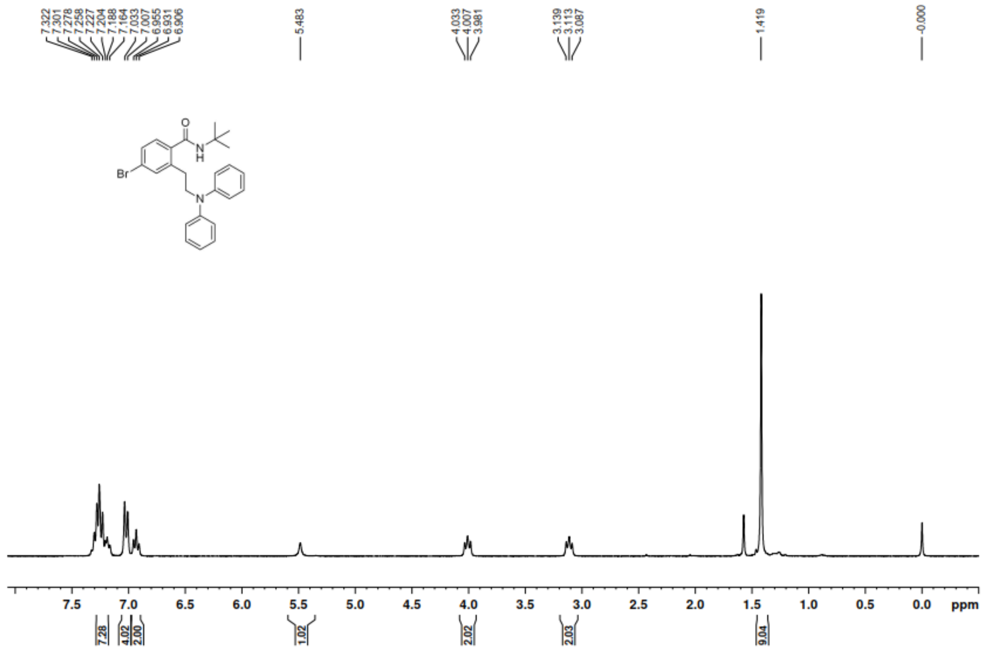

Supplementary Figure 112. ^{1}H NMR (300 MHz, $CDCl_3$) spectra for compound **4p.**

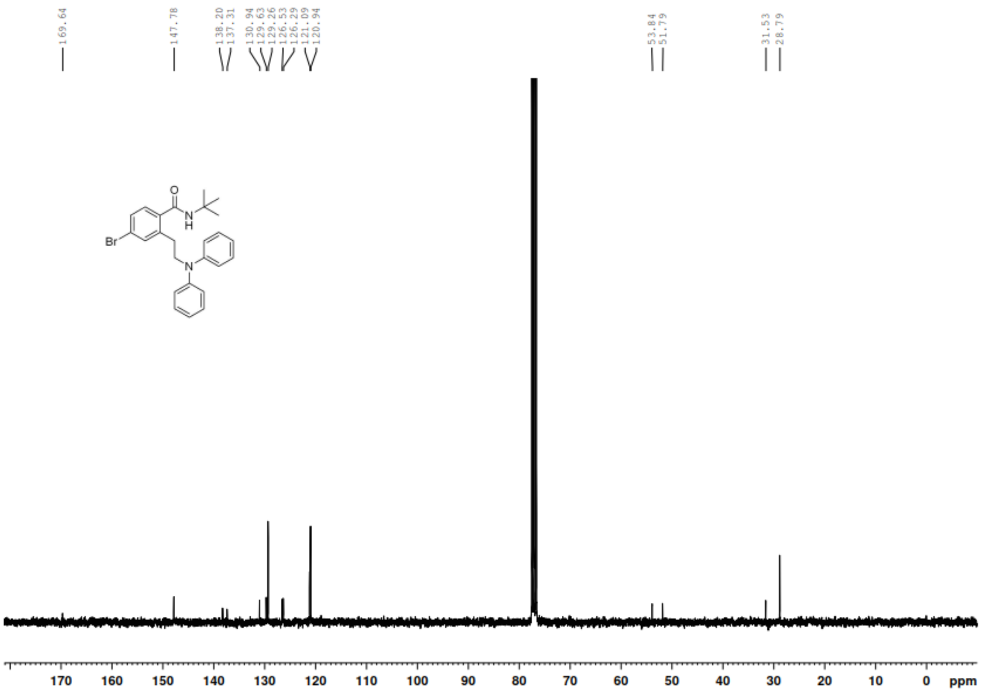

Supplementary Figure 113. ^{13}C NMR (75 MHz, $CDCl_3$) spectra for compound **4p.**

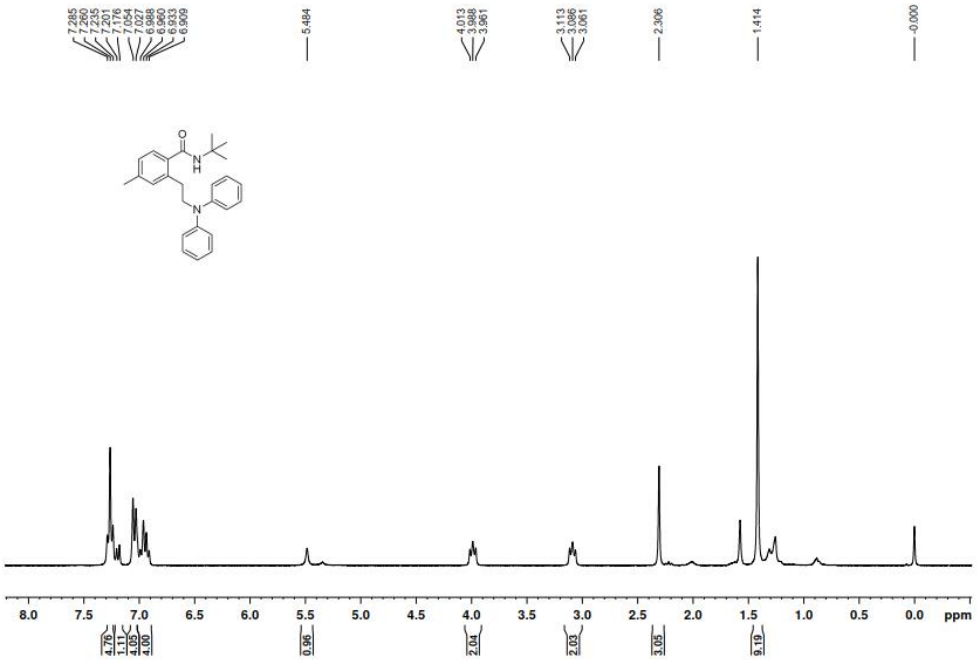

Supplementary Figure 114. ^{1}H NMR (300 MHz, $CDCl_3$) spectra for compound **4q.**

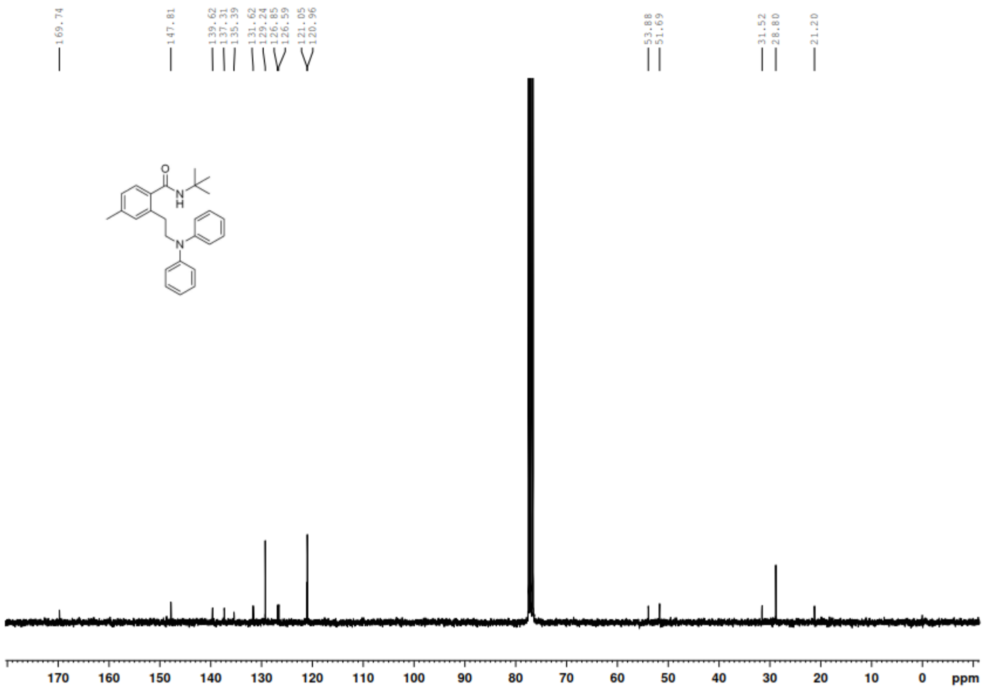

Supplementary Figure 115. ^{13}C NMR (75 MHz, $CDCl_3$) spectra for compound **4q.**

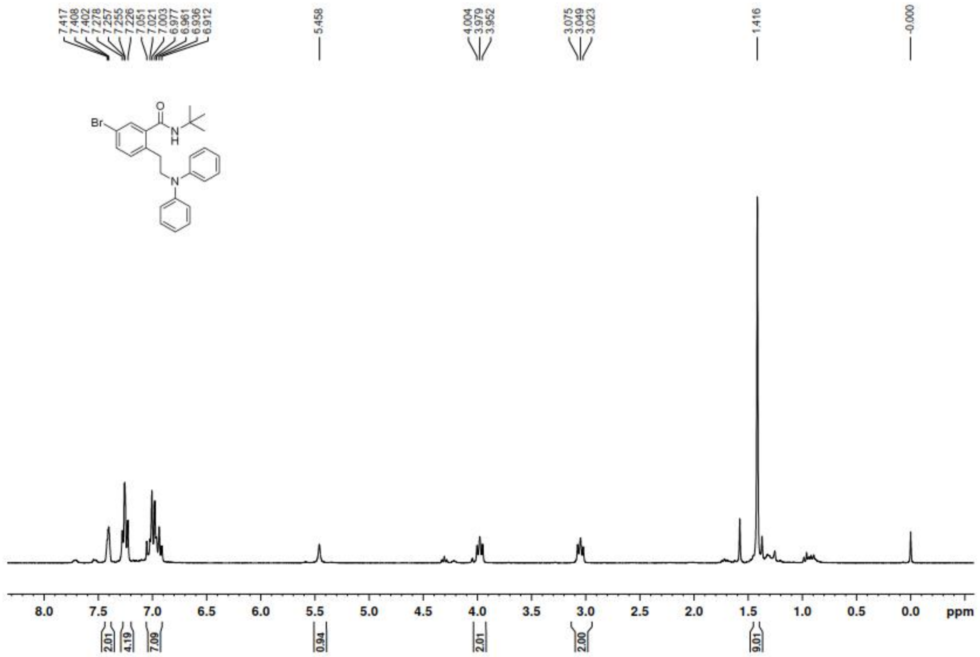

Supplementary Figure 116. ^{1}H NMR (300 MHz, $CDCl_3$) spectra for compound **4r.**

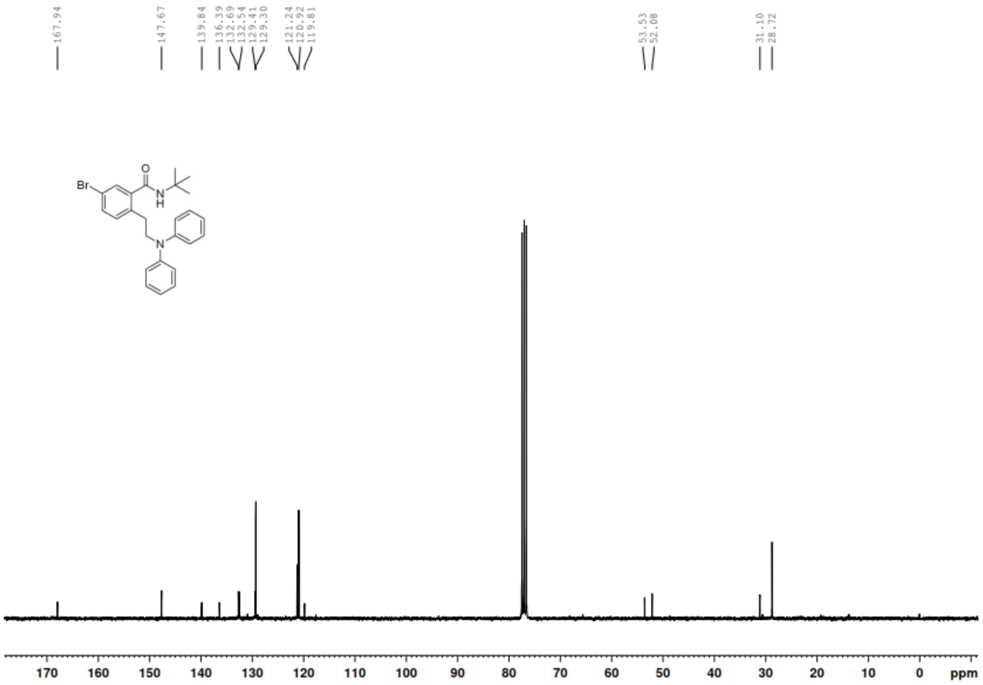

Supplementary Figure 117. ^{13}C NMR (75 MHz, $CDCl_3$) spectra for compound **4r.**

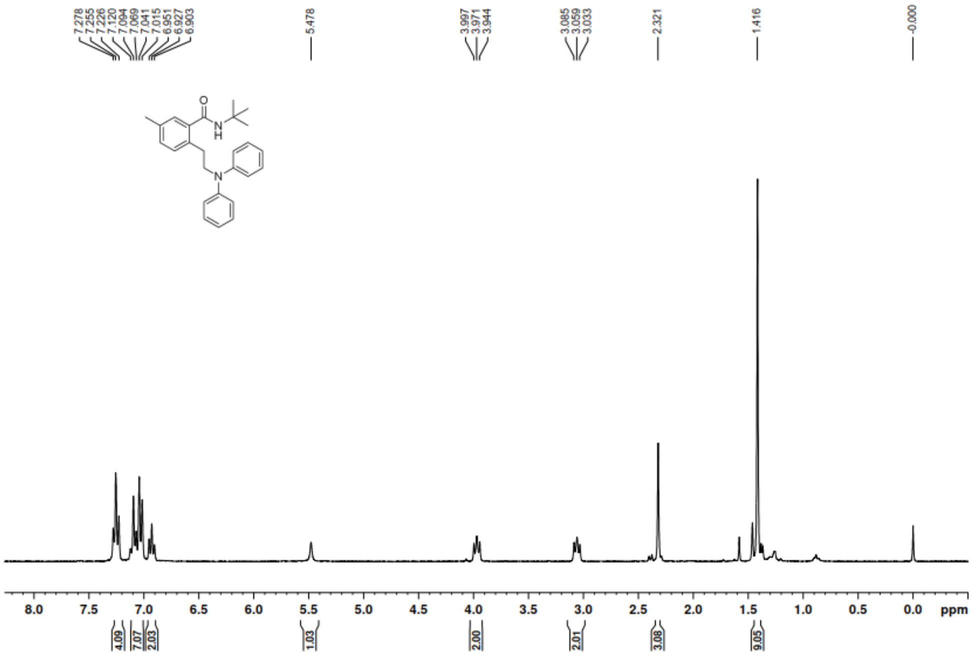

Supplementary Figure 118. ^{1}H NMR (300 MHz, $CDCl_3$) spectra for compound **4s.**

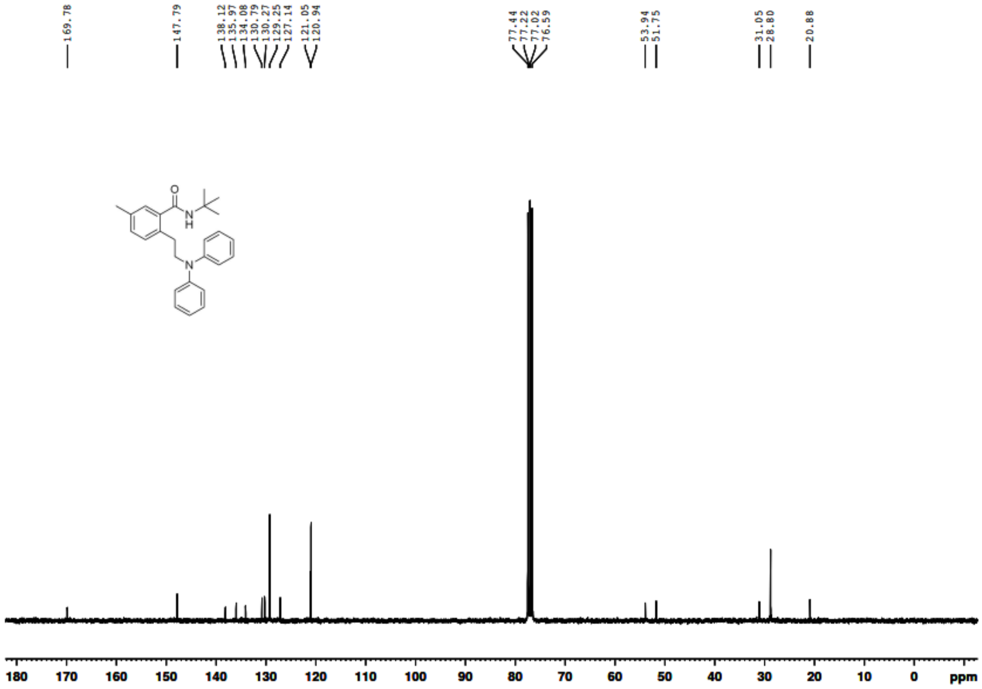

Supplementary Figure 119. ^{13}C NMR (75 MHz, $CDCl_3$) spectra for compound **4s.**

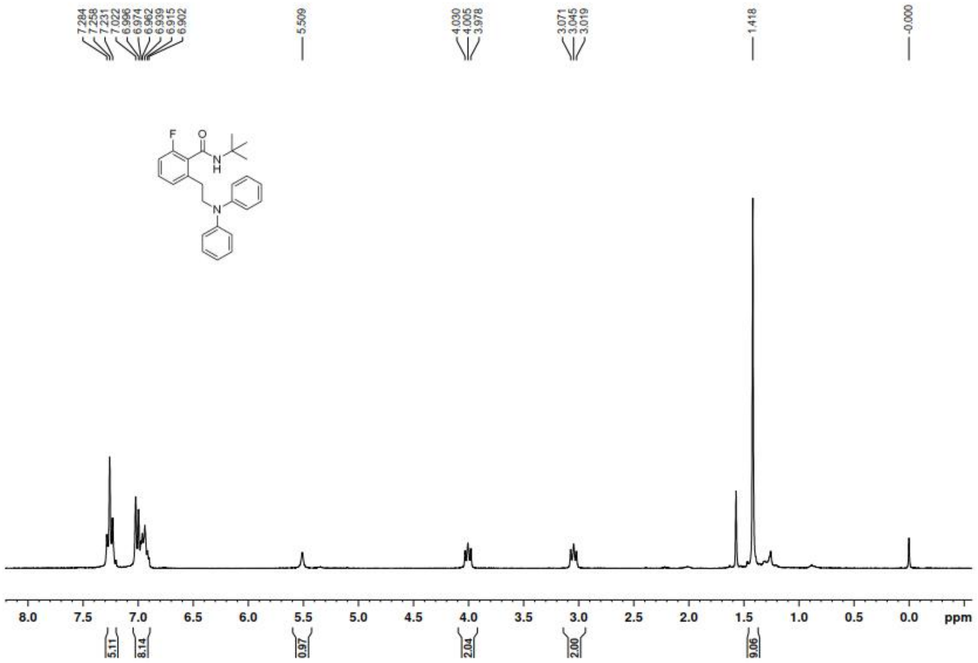

Supplementary Figure 120. ^{1}H NMR (300 MHz, $CDCl_3$) spectra for compound **4t.**

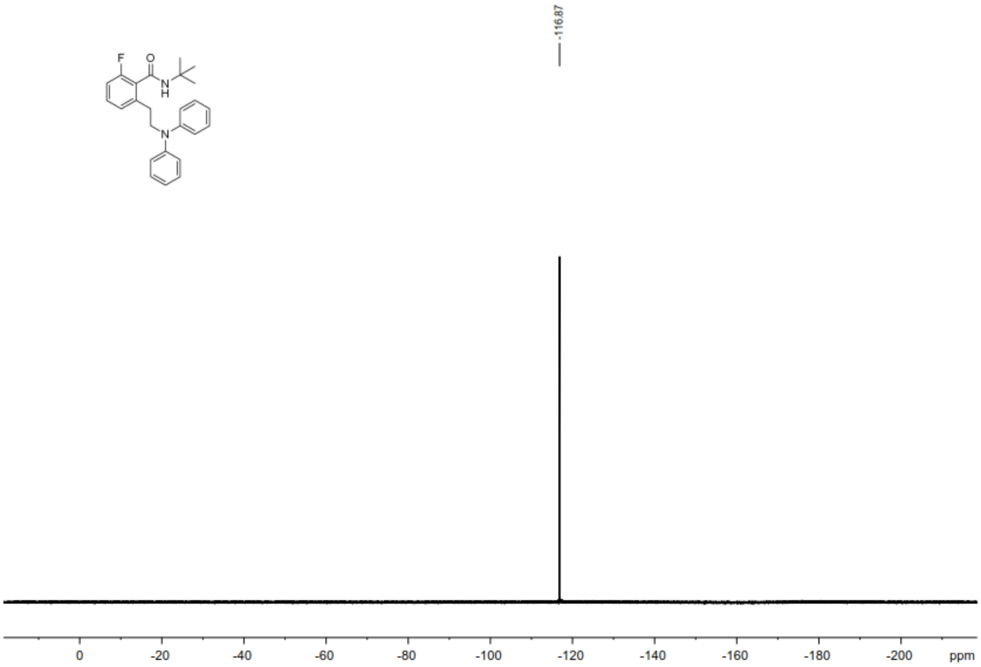

Supplementary Figure 121. ^{19}F NMR (282 MHz, $CDCl_3$) spectra for compound **4t.**

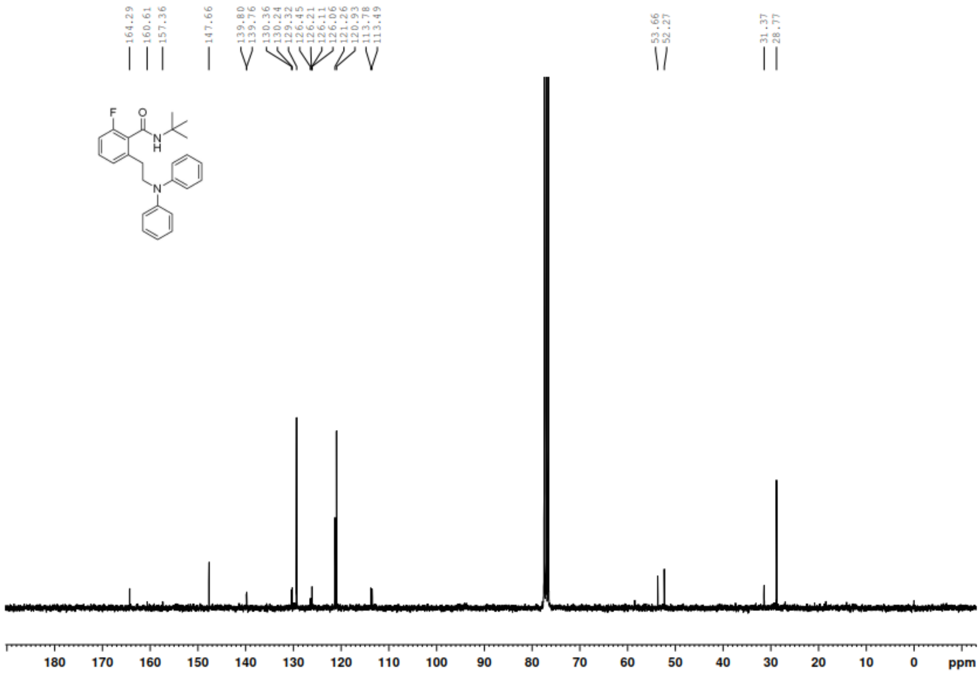

Supplementary Figure 122. ^{13}C NMR (75 MHz, $CDCl_3$) spectra for compound **4t.**

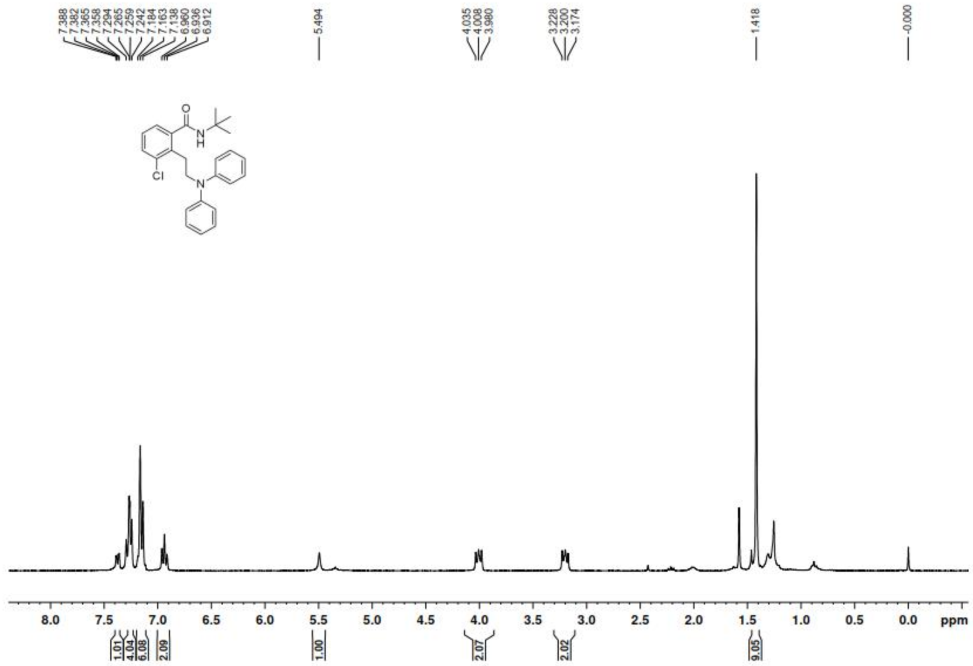

Supplementary Figure 123. ^{1}H NMR (300 MHz, $CDCl_3$) spectra for compound **4u.**

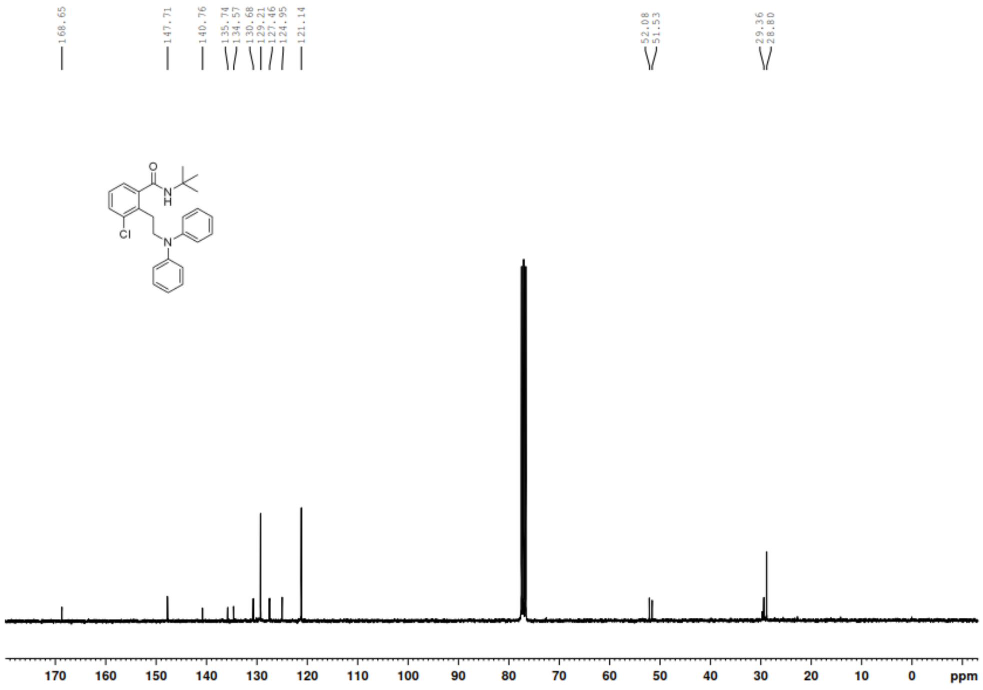

Supplementary Figure 124. ^{13}C NMR (75 MHz, $CDCl_3$) spectra for compound **4u.**

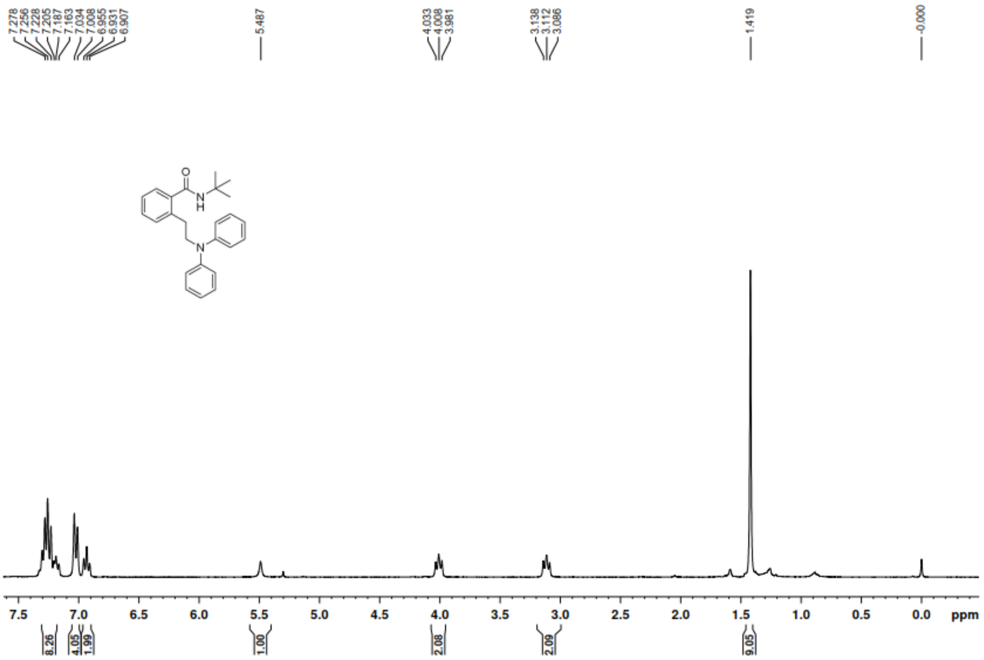

Supplementary Figure 125. ^{1}H NMR (300 MHz, $CDCl_3$) spectra for compound **4v.**

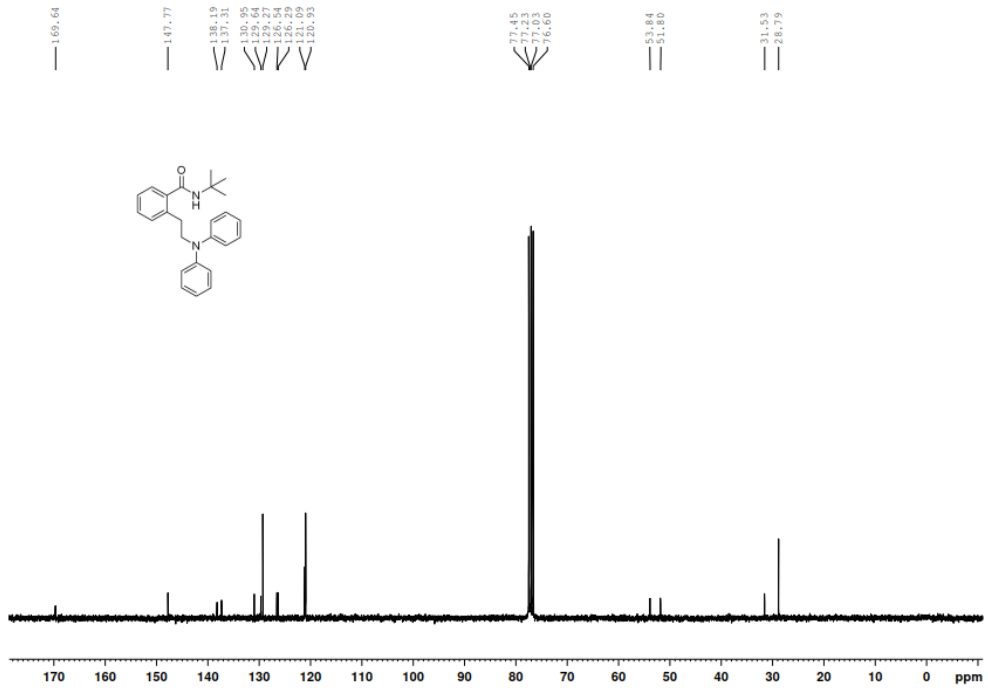

Supplementary Figure 126. ^{13}C NMR (75 MHz, $CDCl_3$) spectra for compound **4v.**

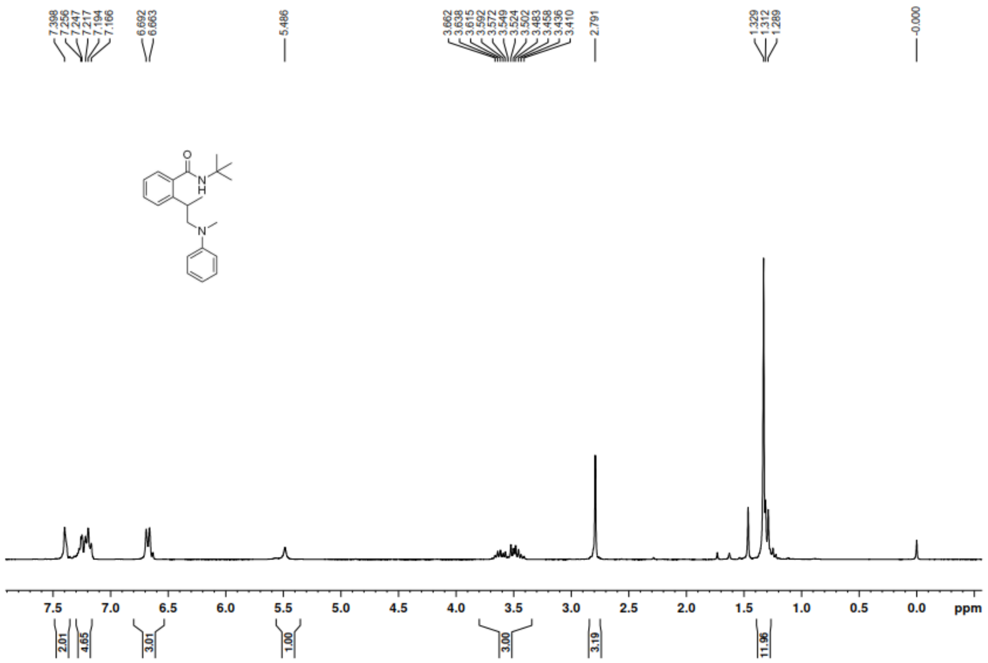

Supplementary Figure 127. ^{1}H NMR (300 MHz, $CDCl_3$) spectra for compound **4w.**

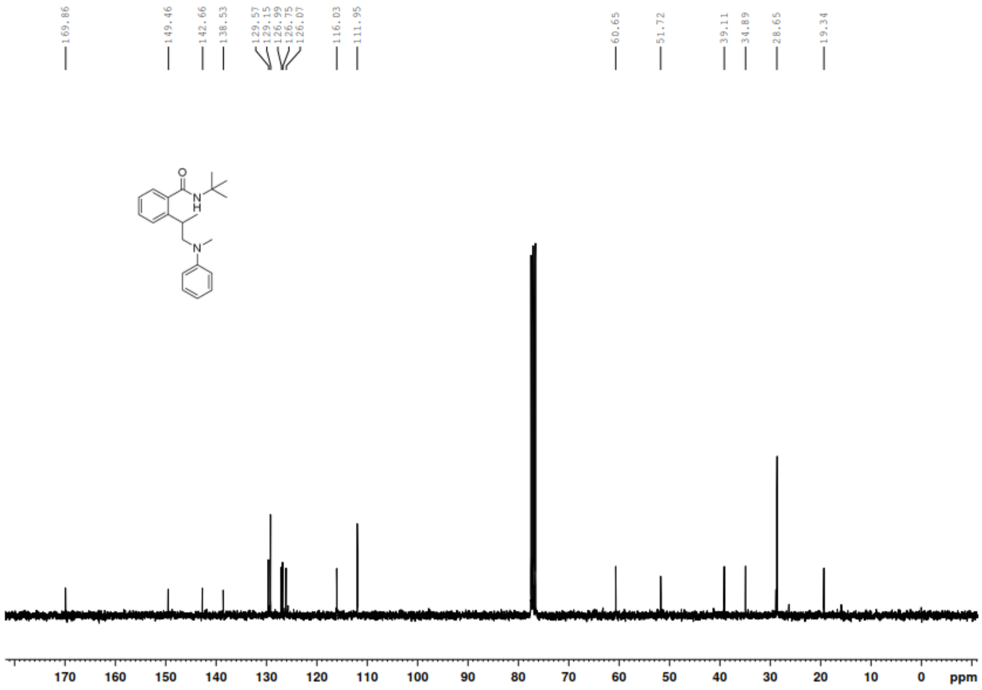

Supplementary Figure 128. ^{13}C NMR (75 MHz, $CDCl_3$) spectra for compound **4w.**

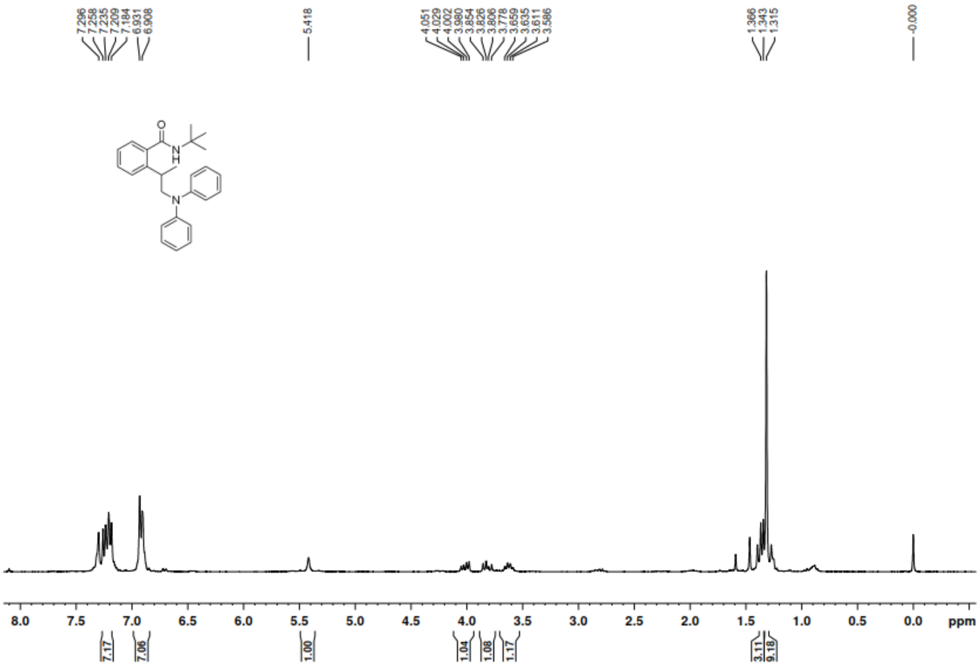

Supplementary Figure 129. ^{1}H NMR (300 MHz, $CDCl_3$) spectra for compound **4x.**

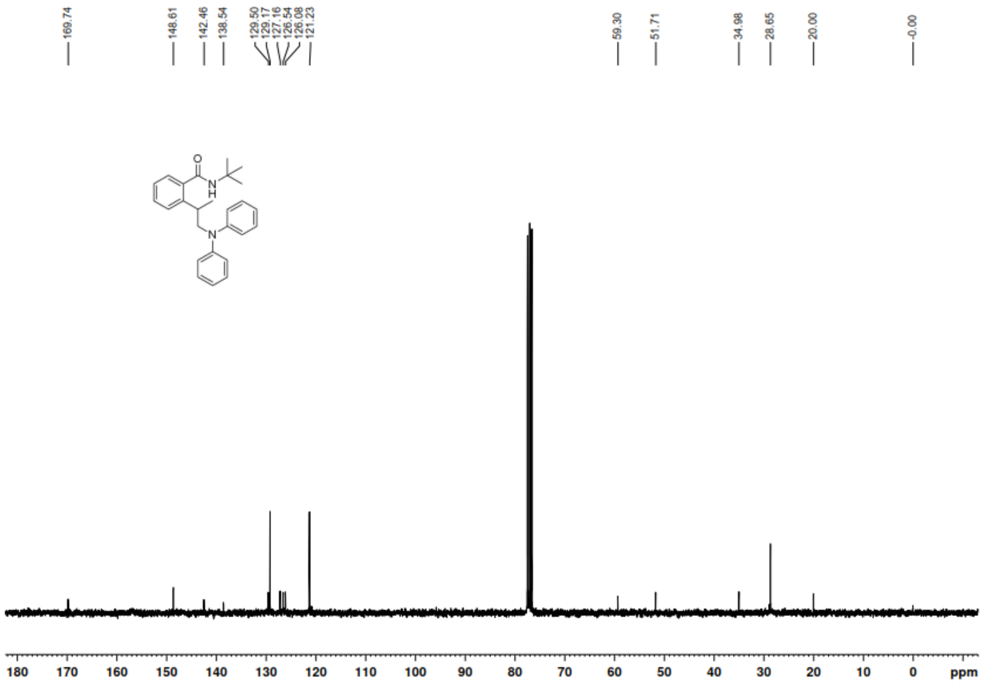

Supplementary Figure 130. ^{13}C NMR (75 MHz, $CDCl_3$) spectra for compound **4x.**

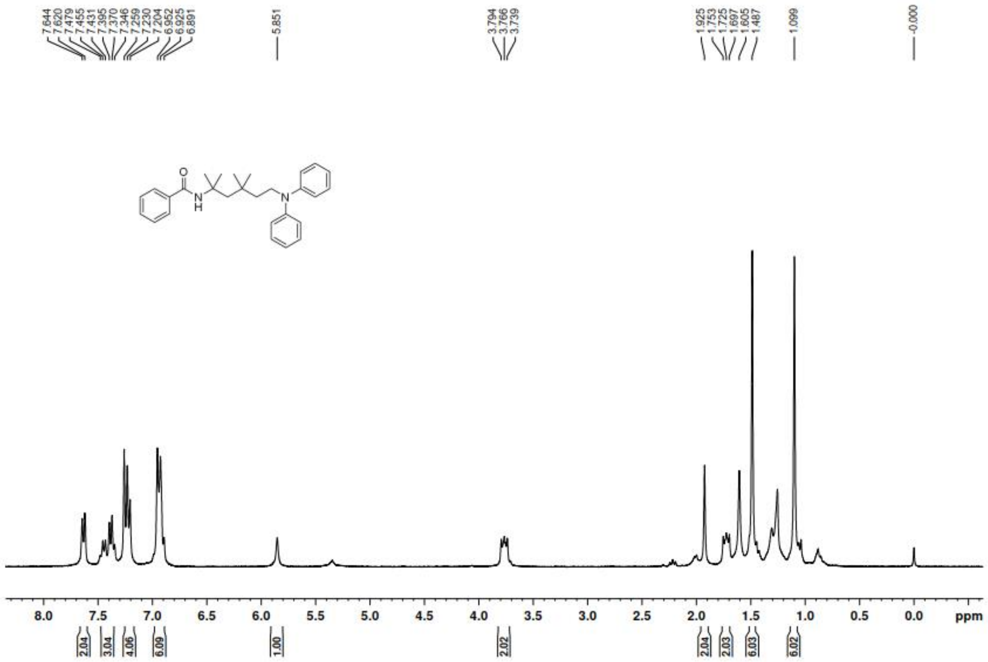

Supplementary Figure 131. ^{1}H NMR (300 MHz, $CDCl_3$) spectra for compound **5.**

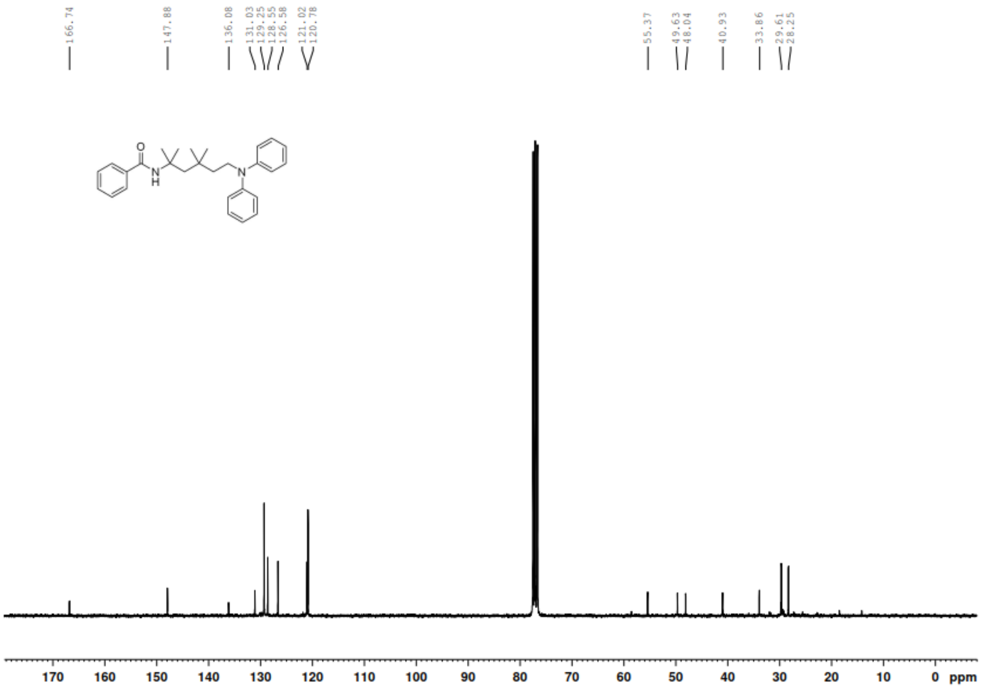

Supplementary Figure 132. ^{13}C NMR (75 MHz, $CDCl_3$) spectra for compound **5.**

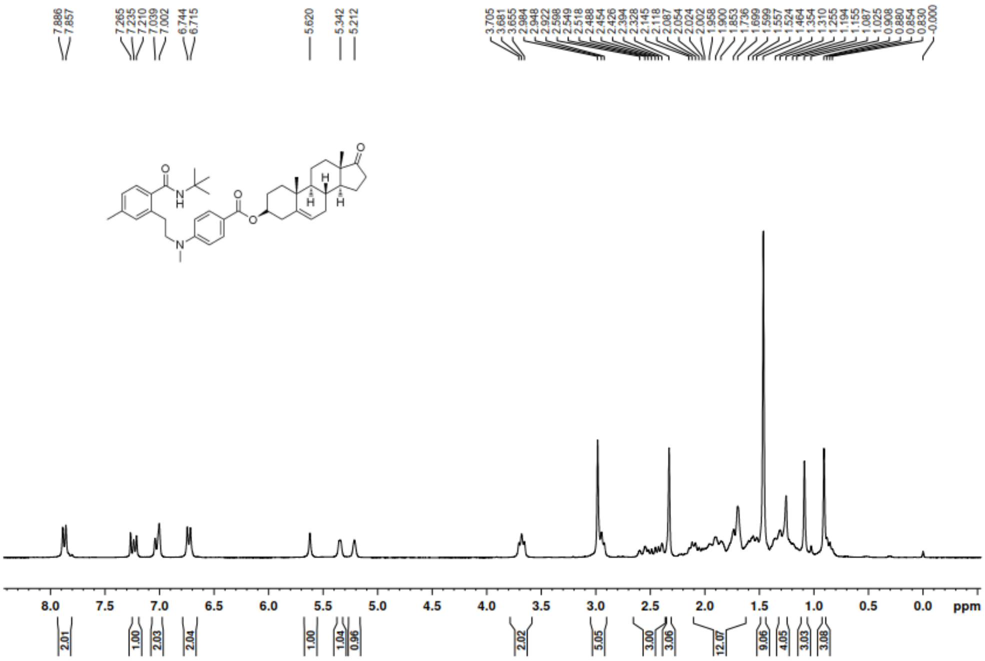

Supplementary Figure 133. ^{1}H NMR (300 MHz, $CDCl_3$) spectra for compound **6.**

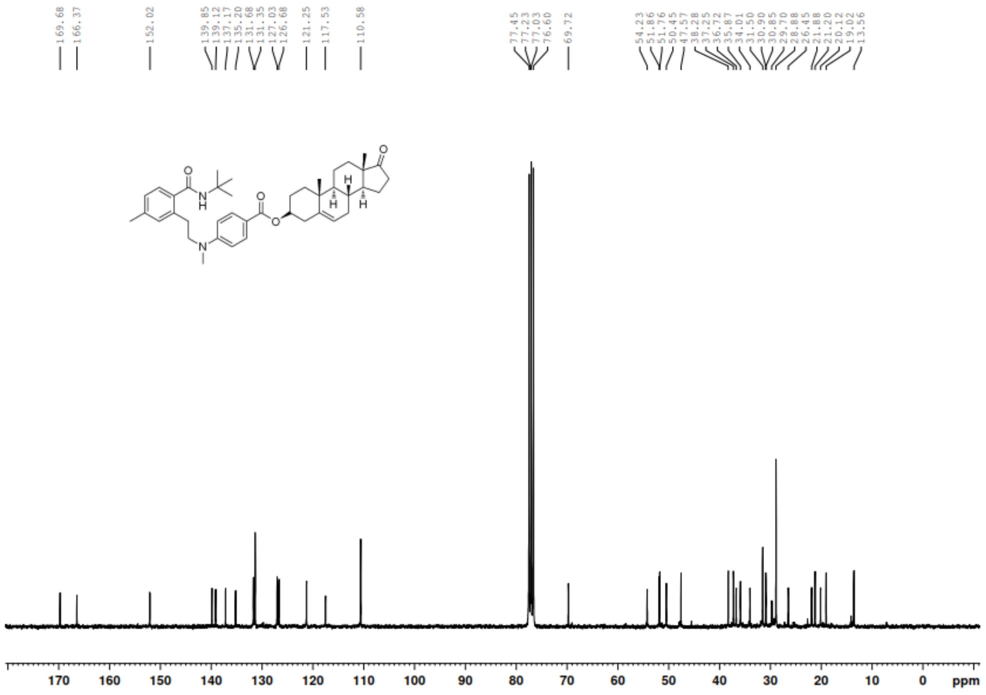

Supplementary Figure 134. ^{13}C NMR (75 MHz, $CDCl_3$) spectra for compound **6.**

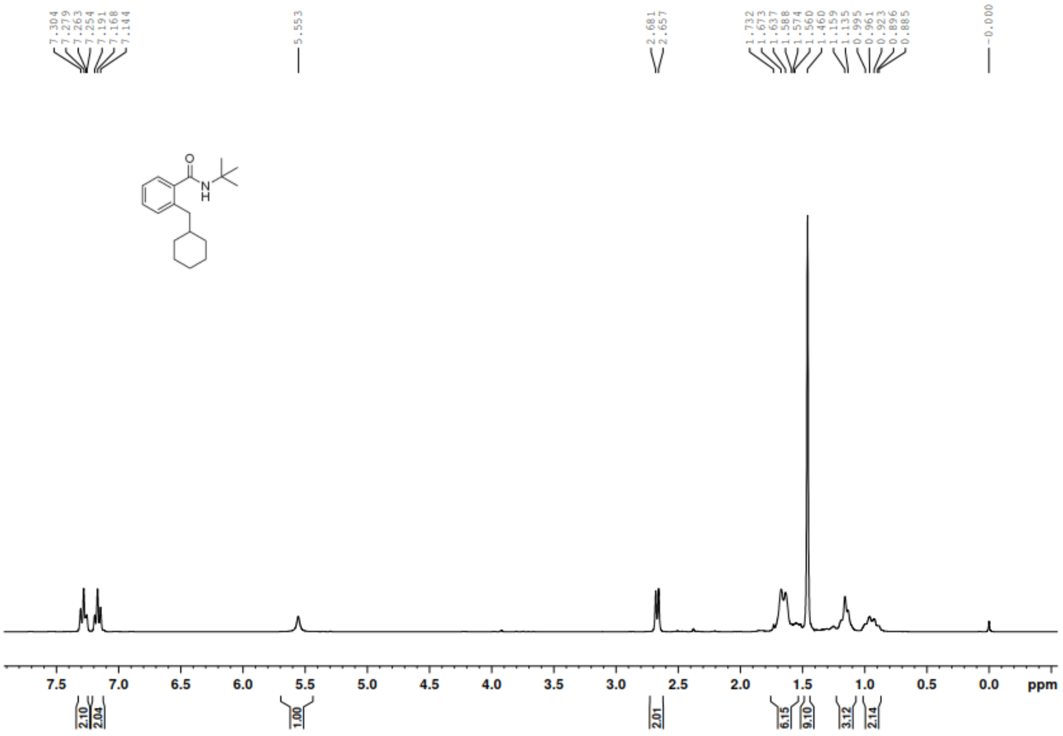

Supplementary Figure 135. ^{1}H NMR (300 MHz, $CDCl_3$) spectra for compound **8a.**

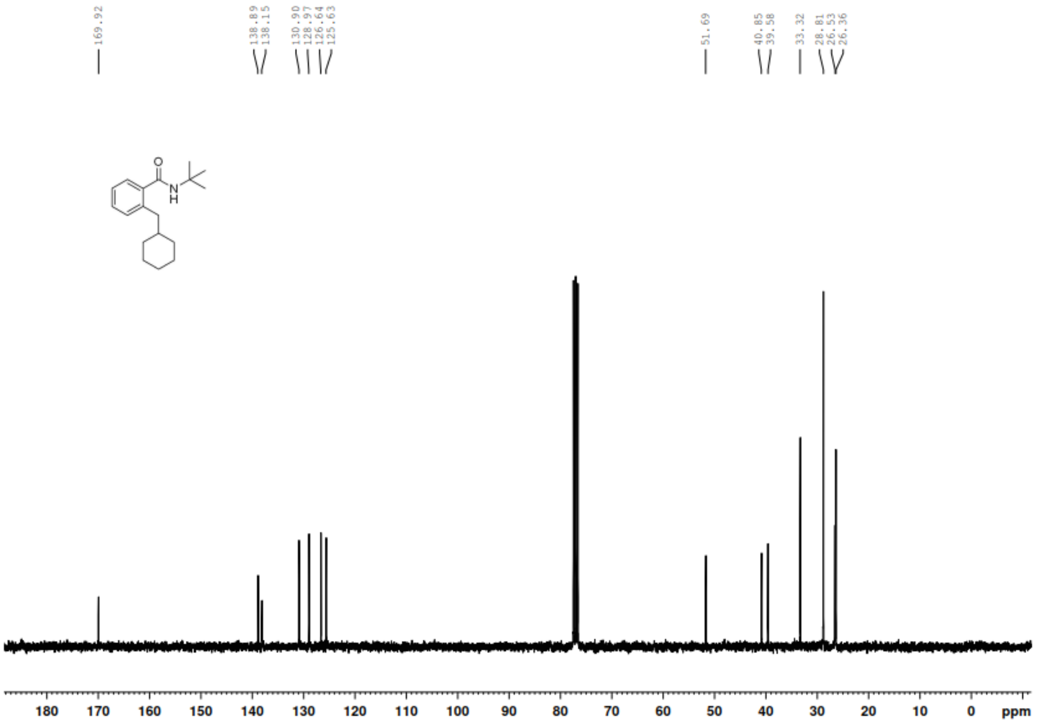

Supplementary Figure 136. ^{13}C NMR (75 MHz, $CDCl_3$) spectra for compound **8a.**

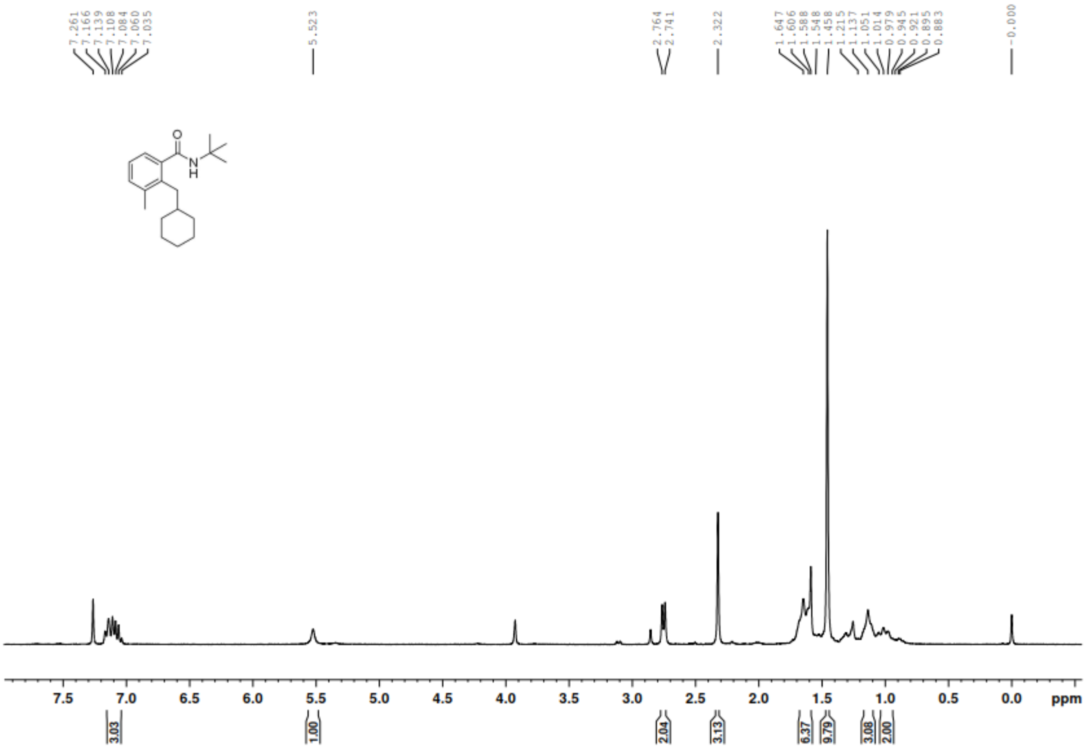

Supplementary Figure 137. ^{1}H NMR (300 MHz, $CDCl_3$) spectra for compound **8b.**

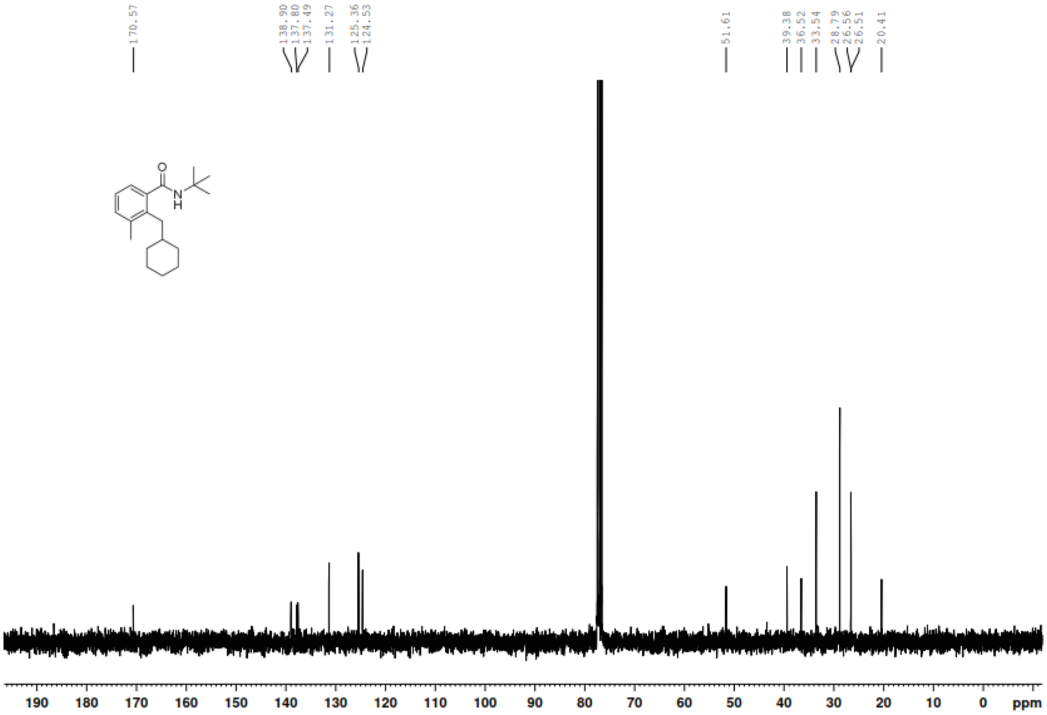

Supplementary Figure 138. ^{13}C NMR (75 MHz, $CDCl_3$) spectra for compound **8b.**

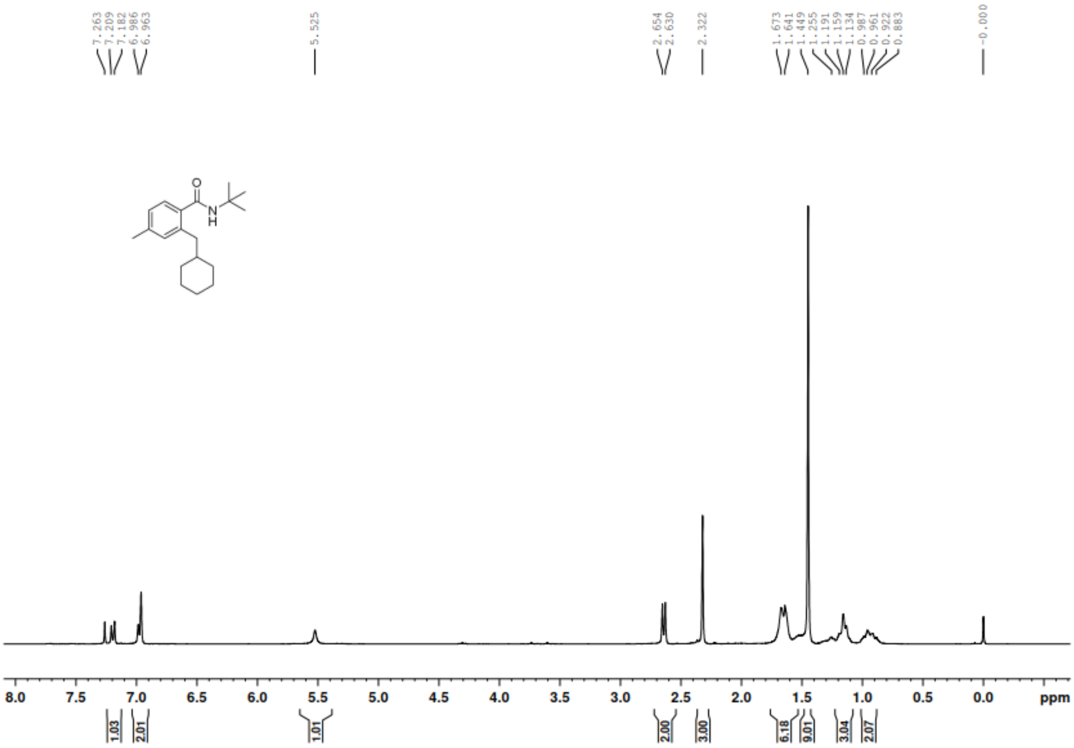

Supplementary Figure 139. ^{1}H NMR (300 MHz, $CDCl_3$) spectra for compound **8c.**

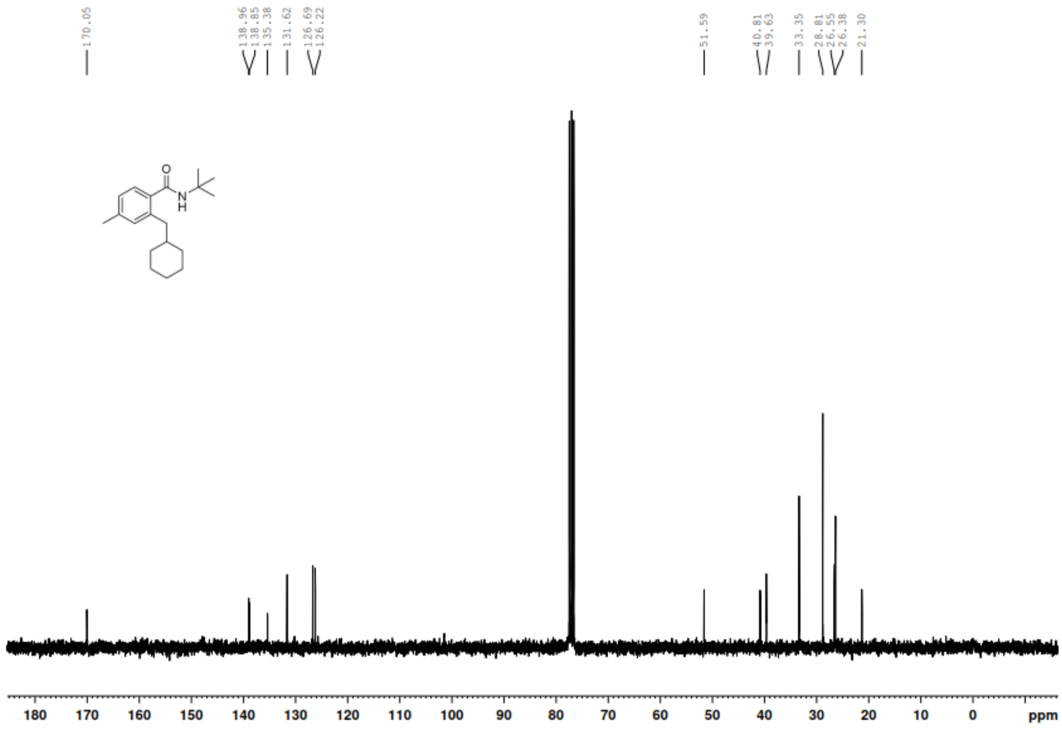

Supplementary Figure 140. ^{13}C NMR (75 MHz, $CDCl_3$) spectra for compound **8c.**

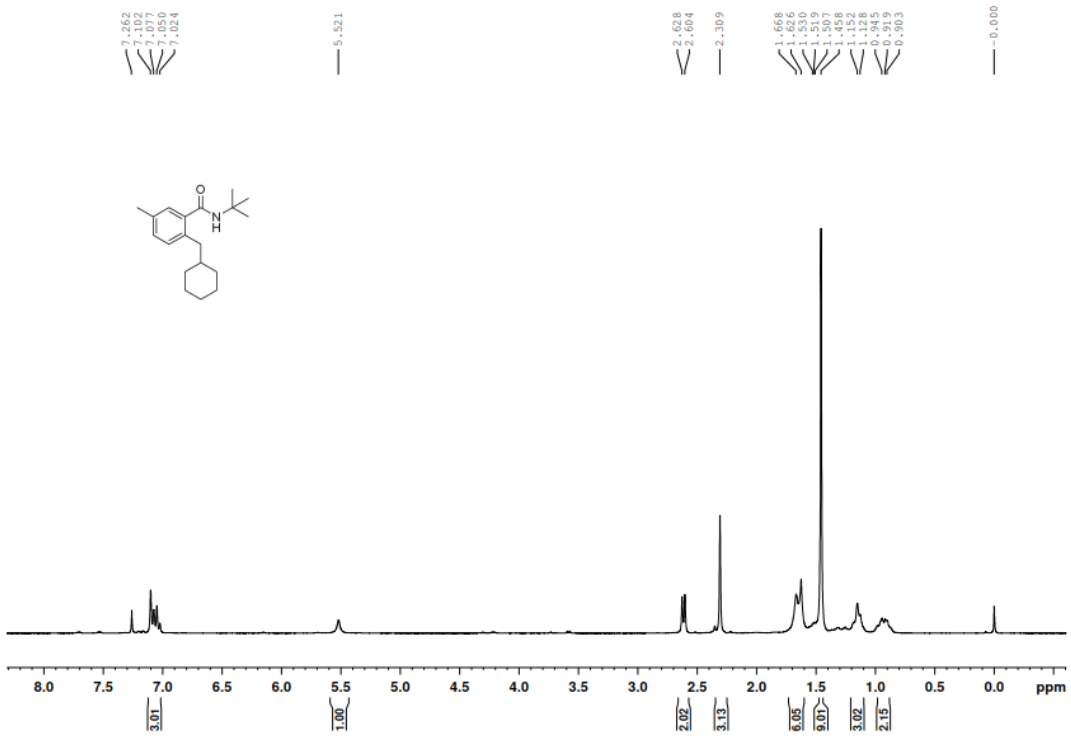

Supplementary Figure 141. ^{1}H NMR (300 MHz, $CDCl_3$) spectra for compound **8d.**

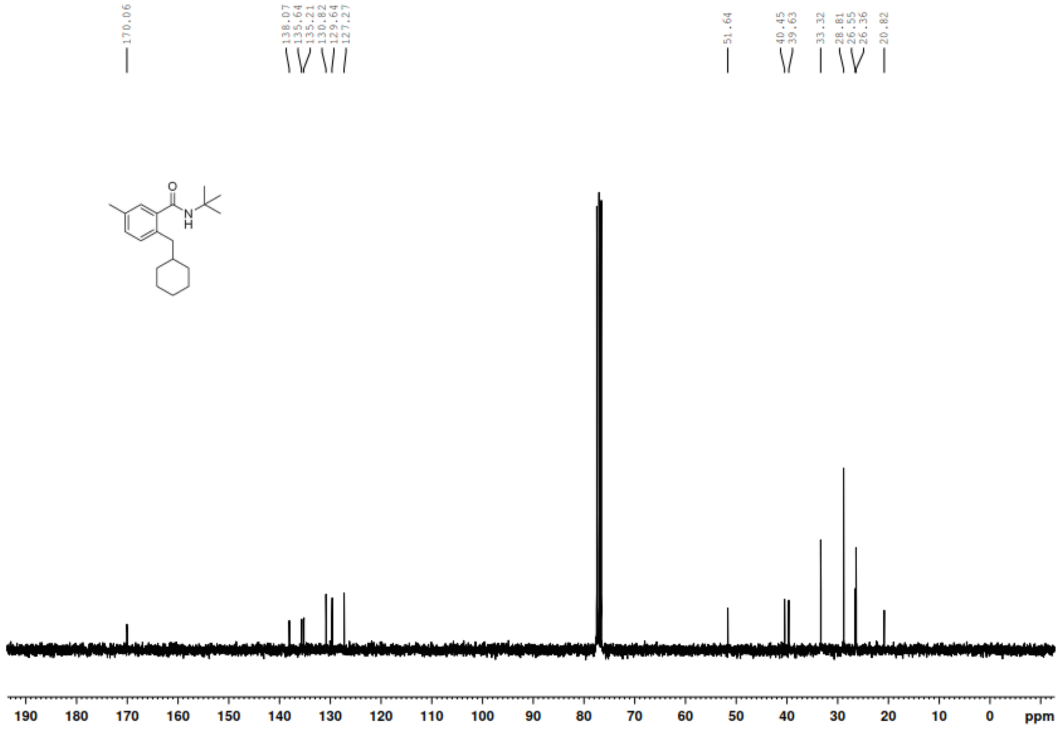

Supplementary Figure 142. ^{13}C NMR (75 MHz, $CDCl_3$) spectra for compound **8d.**

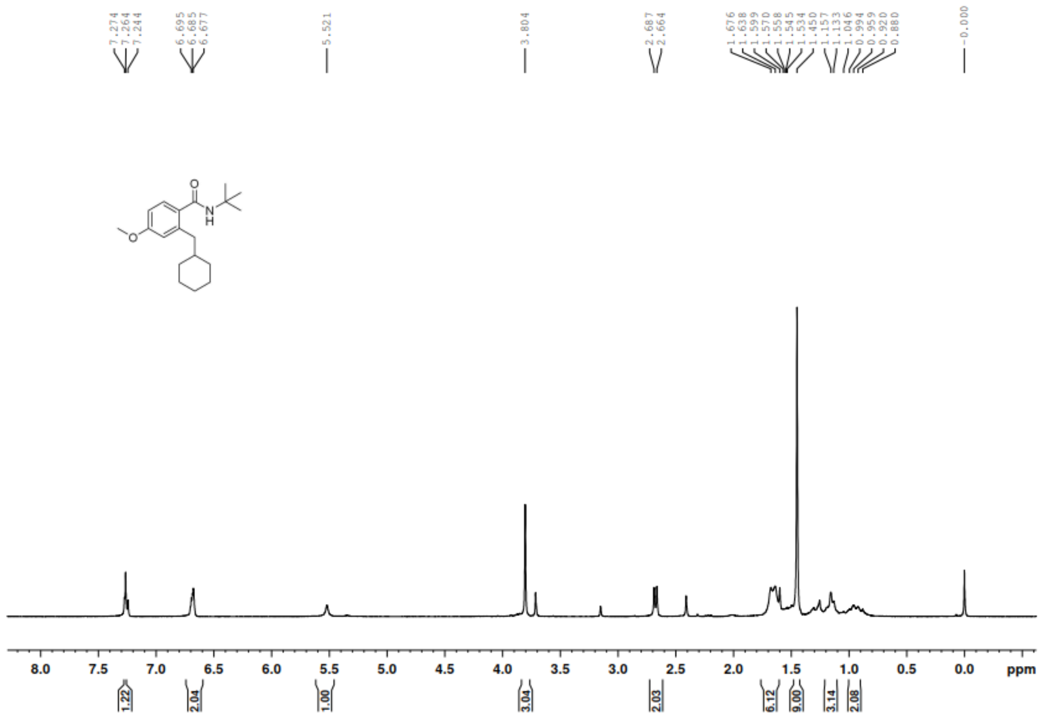

Supplementary Figure 143. ^{1}H NMR (300 MHz, $CDCl_3$) spectra for compound **8e.**

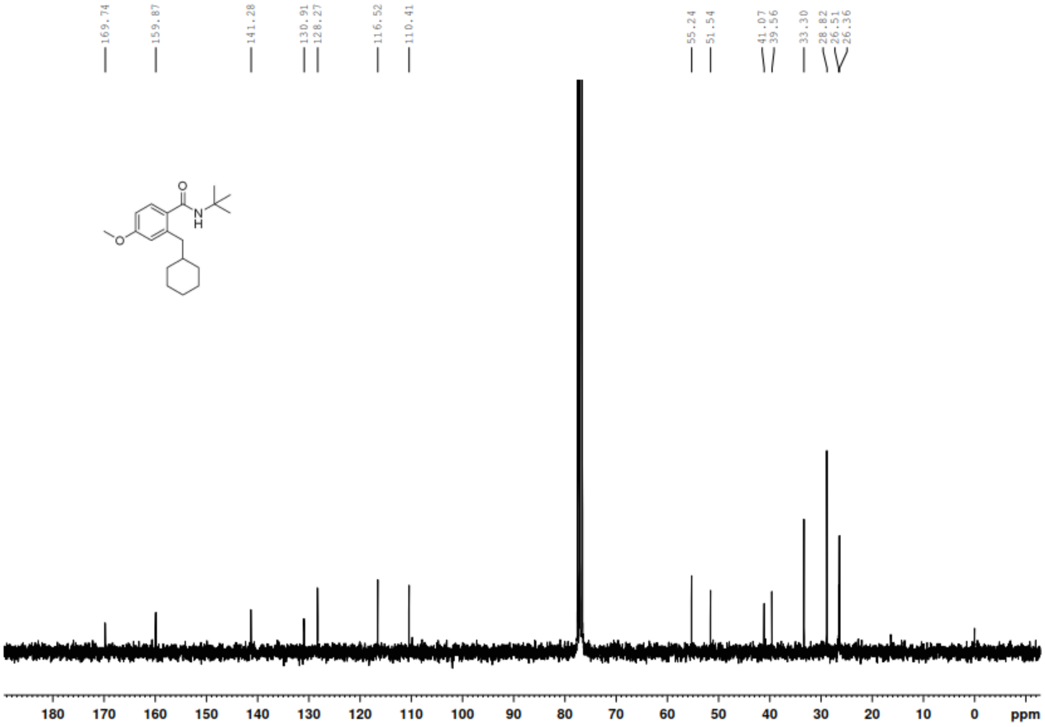

Supplementary Figure 144. ^{13}C NMR (75 MHz, $CDCl_3$) spectra for compound **8e.**

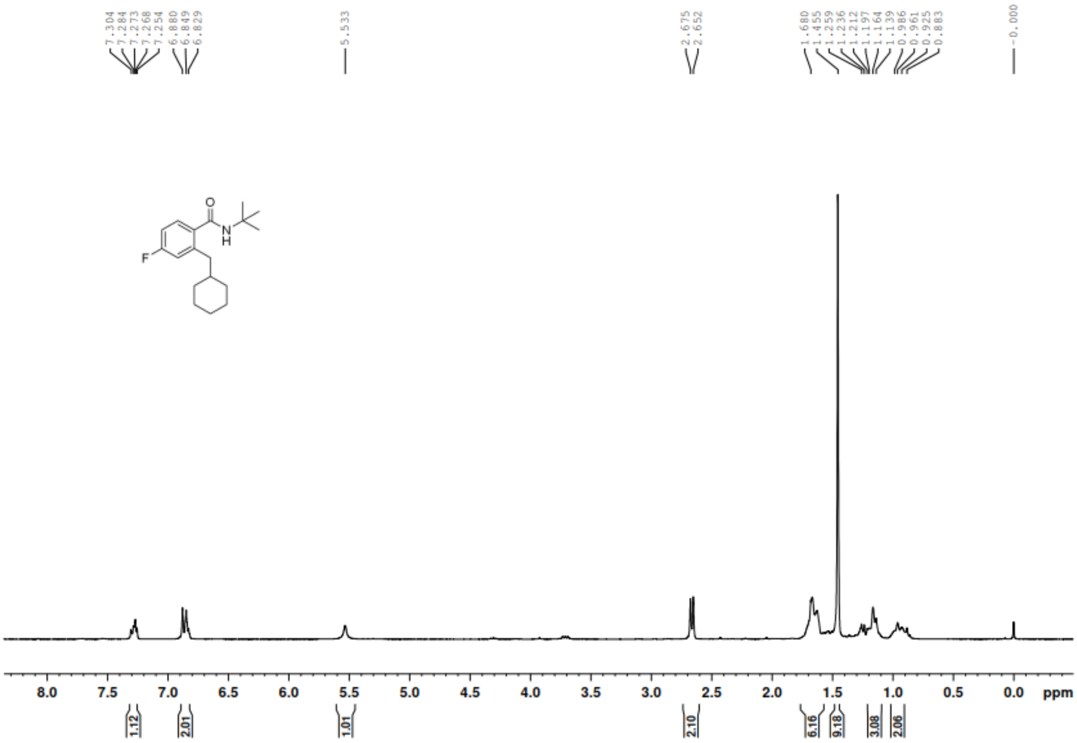

Supplementary Figure 145. ^{1}H NMR (300 MHz, $CDCl_3$) spectra for compound **8f.**

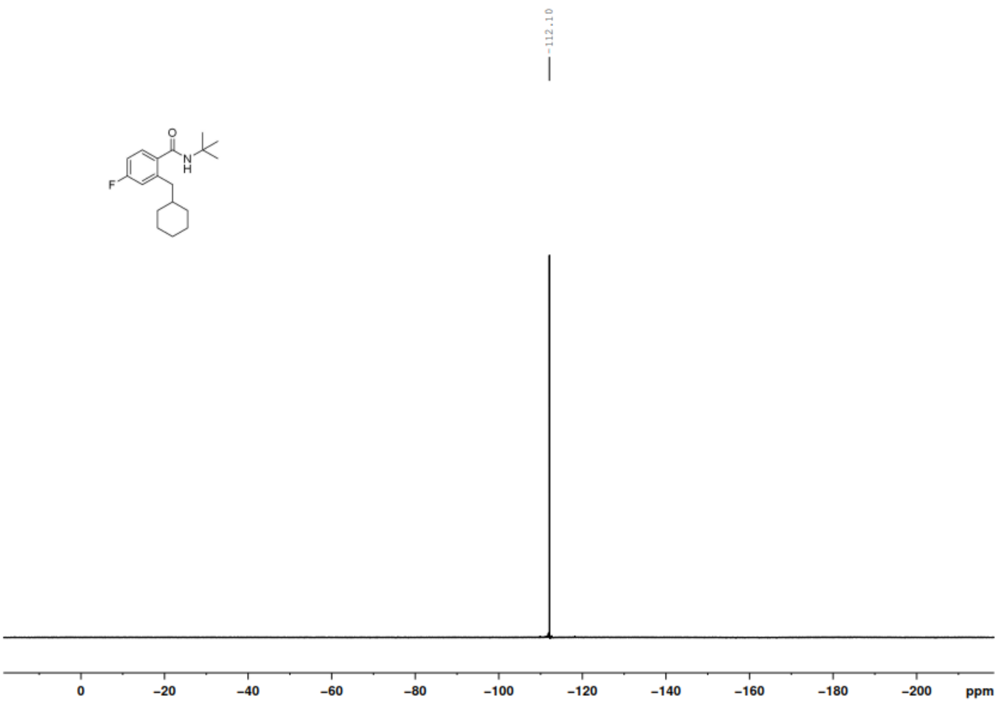

Supplementary Figure 146. ^{19}F NMR (282 MHz, $CDCl_3$) spectra for compound **8f.**

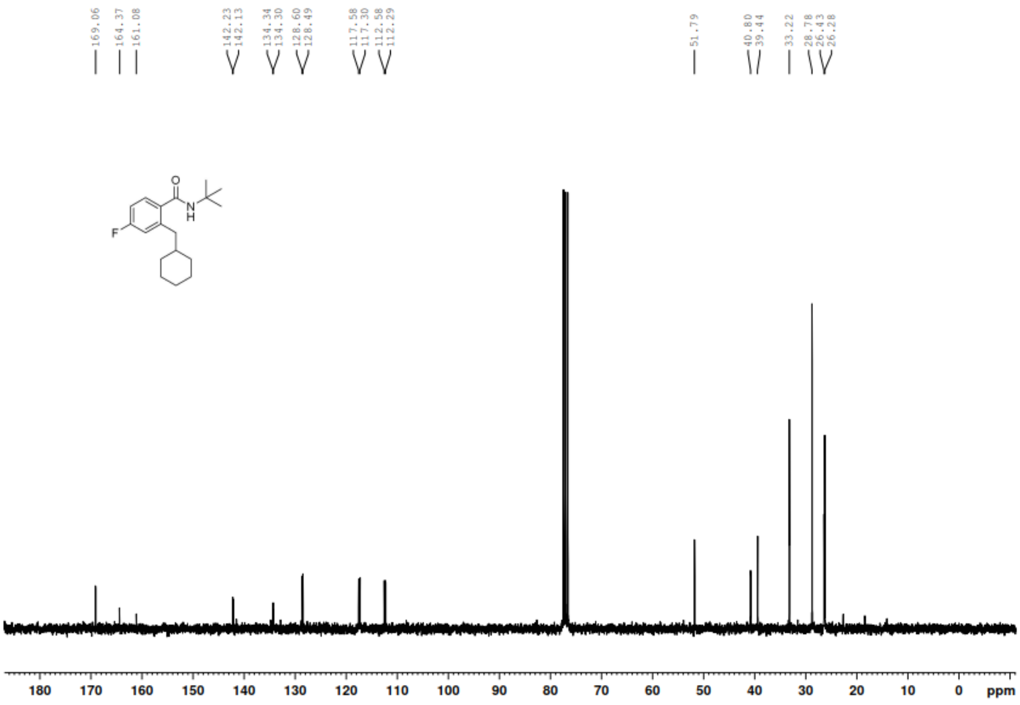

Supplementary Figure 147. ^{13}C NMR (75 MHz, $CDCl_3$) spectra for compound **8f.**

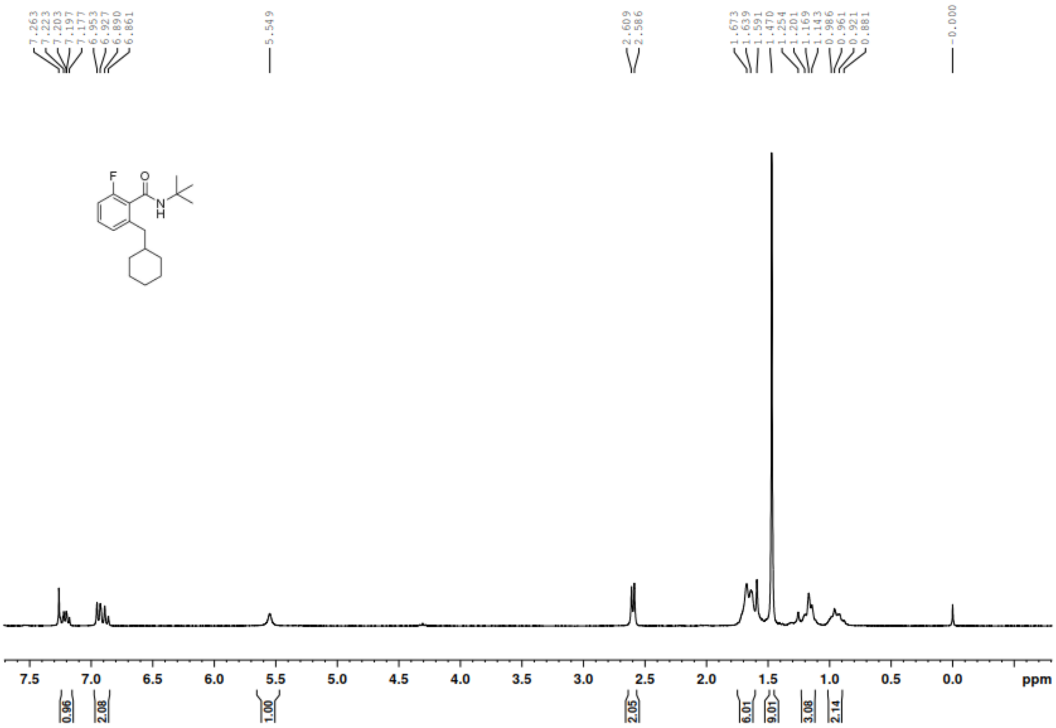

Supplementary Figure 148. ^{1}H NMR (300 MHz, $CDCl_3$) spectra for compound **8g.**

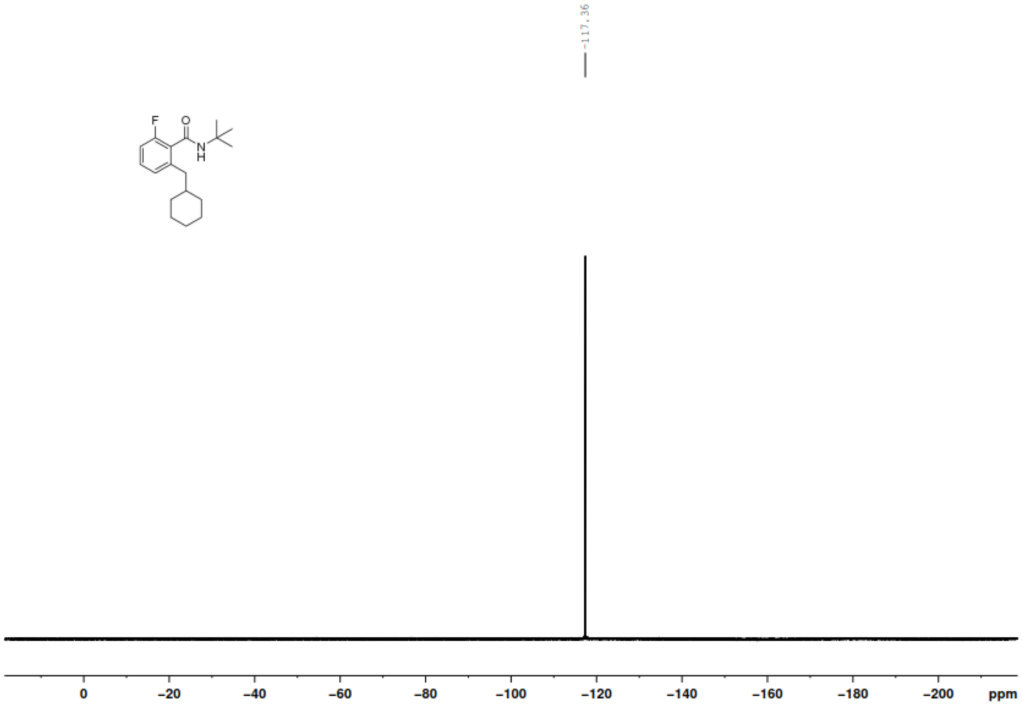

Supplementary Figure 149. ^{19}F NMR (282 MHz, $CDCl_3$) spectra for compound **8g.**

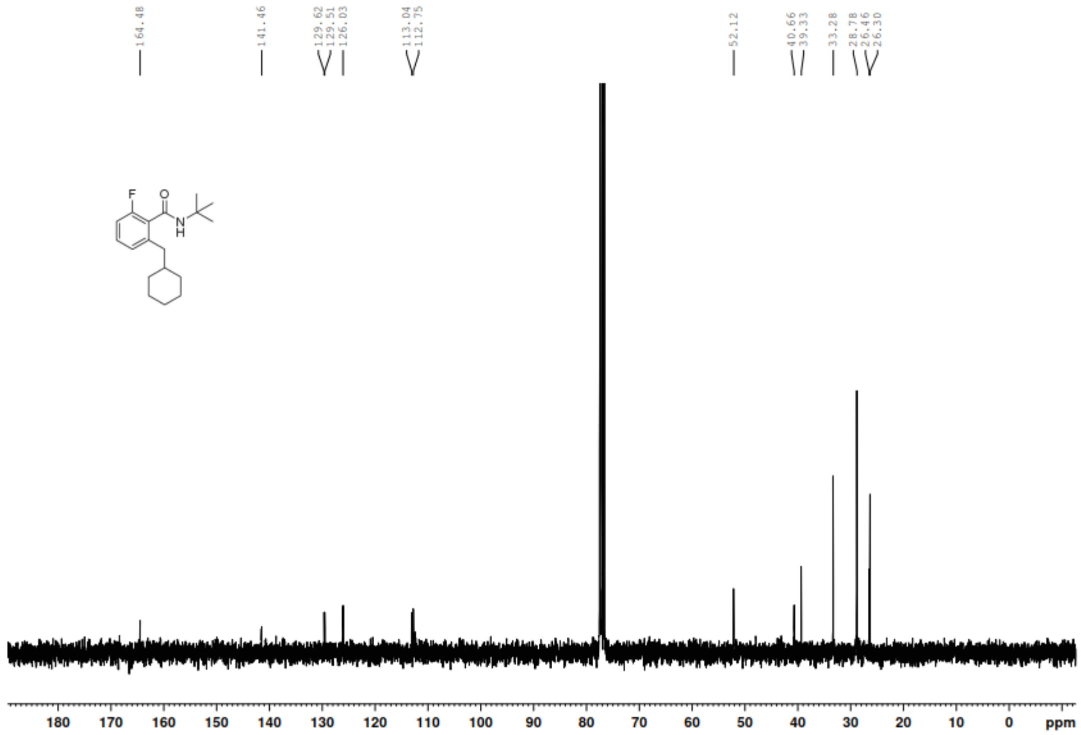

Supplementary Figure 150. ^{13}C NMR (75 MHz, $CDCl_3$) spectra for compound **8g.**

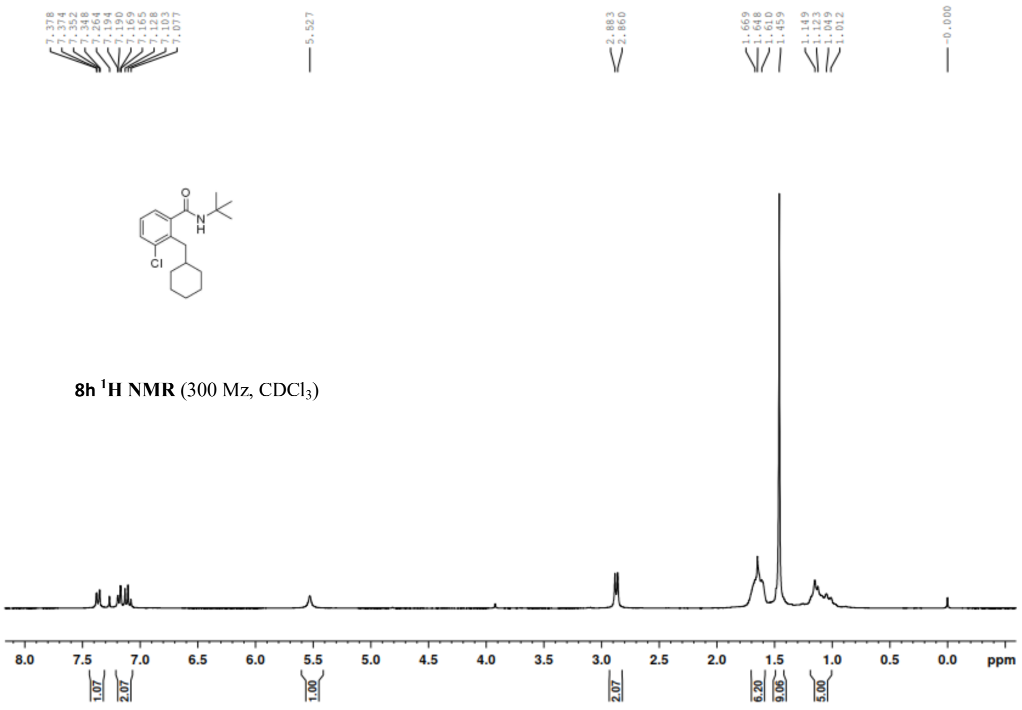

Supplementary Figure 151. ^{1}H NMR (300 MHz, $CDCl_3$) spectra for compound **8h.**

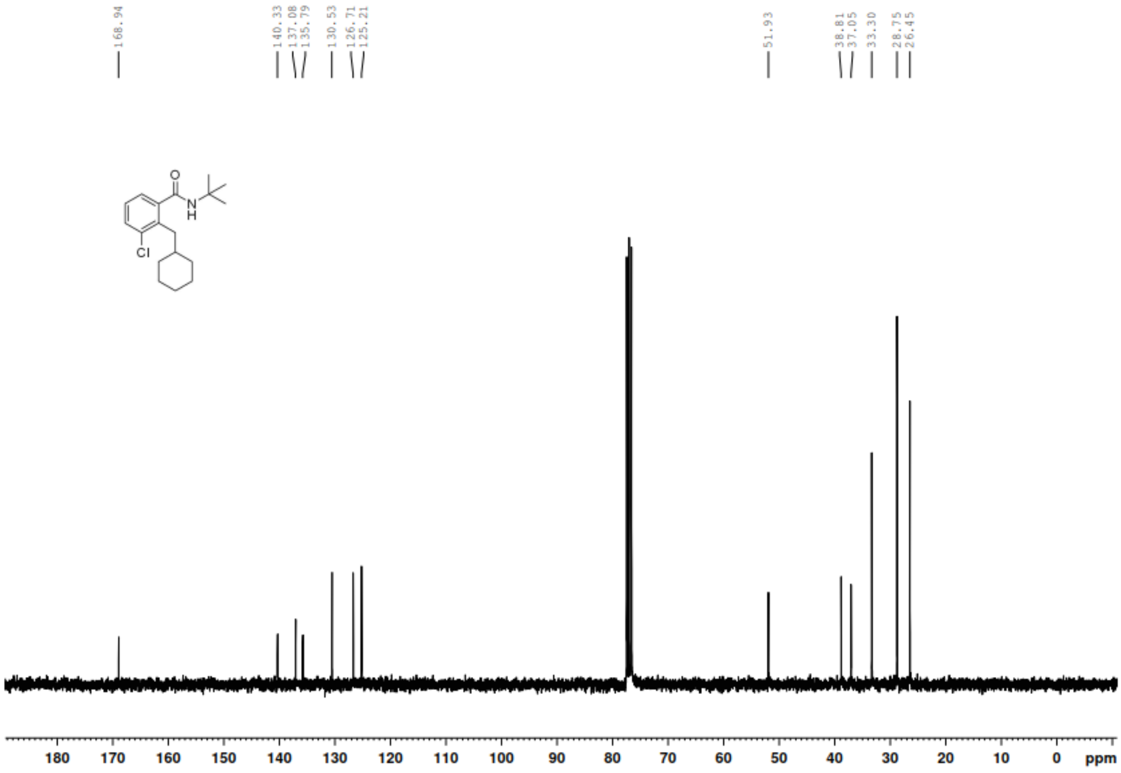

Supplementary Figure 152. ^{13}C NMR (75 MHz, $CDCl_3$) spectra for compound **8h.**

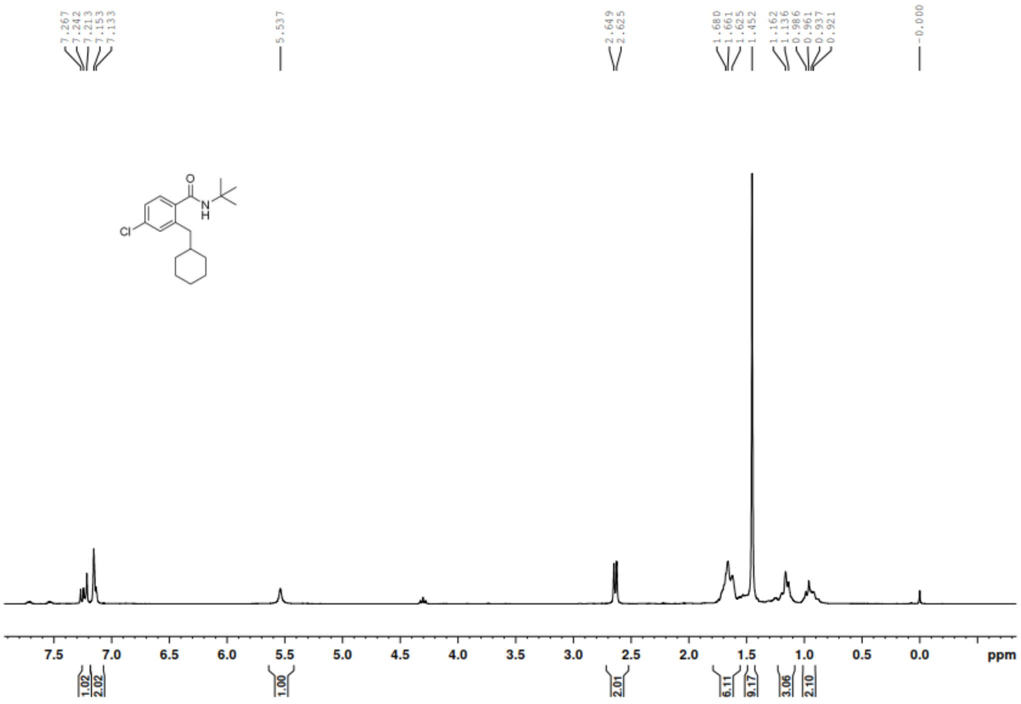

Supplementary Figure 153. ^{1}H NMR (300 MHz, $CDCl_3$) spectra for compound **8i.**

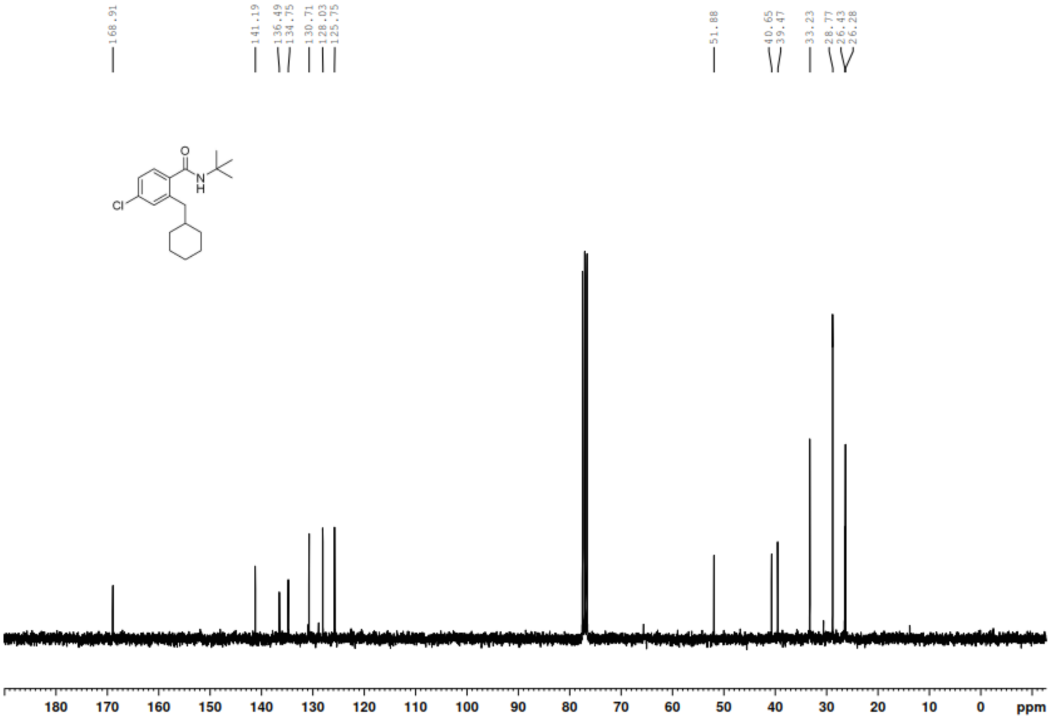

Supplementary Figure 154. ^{13}C NMR (75 MHz, $CDCl_3$) spectra for compound **8i.**

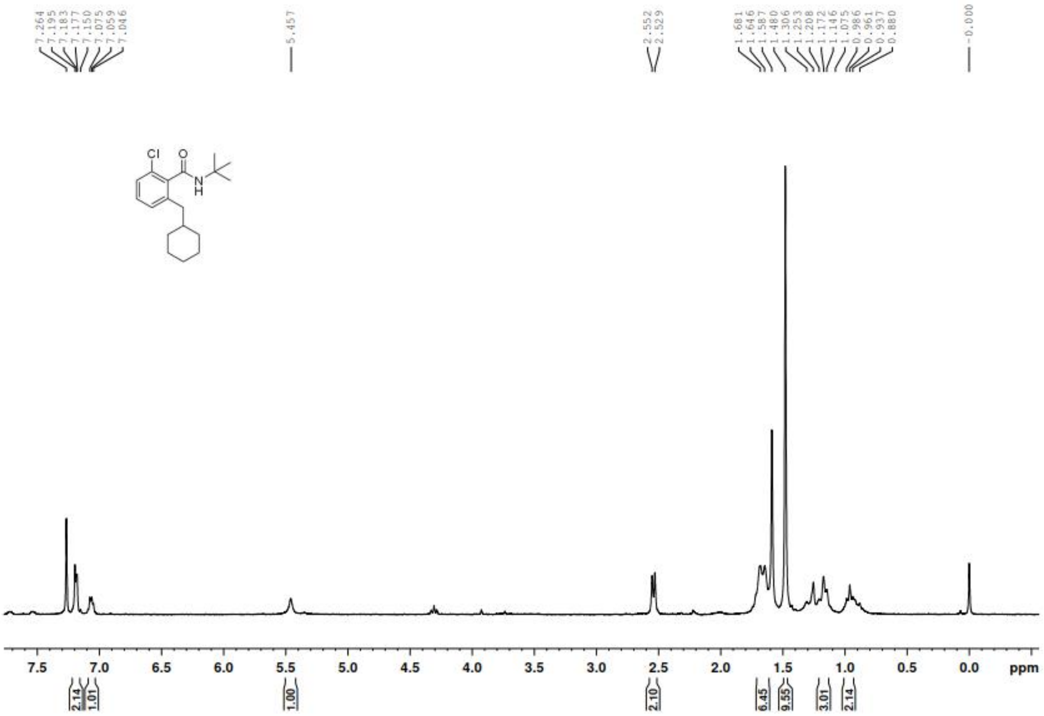

Supplementary Figure 155. ^{1}H NMR (300 MHz, $CDCl_3$) spectra for compound **8j.**

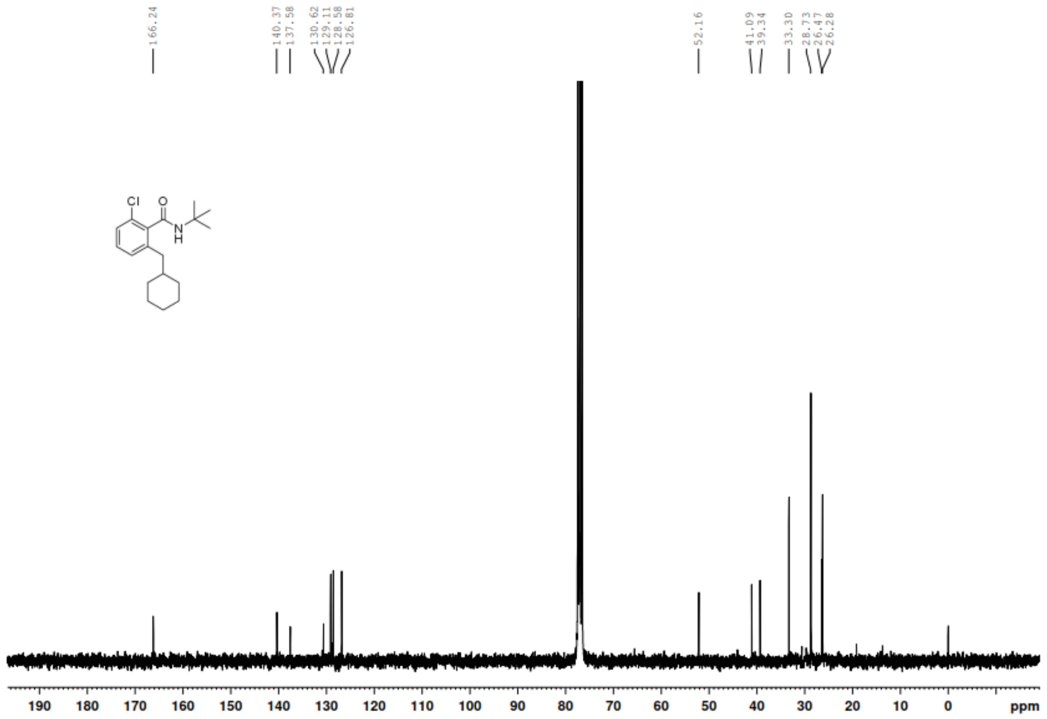

Supplementary Figure 156. ^{13}C NMR (75 MHz, $CDCl_3$) spectra for compound **8j.**

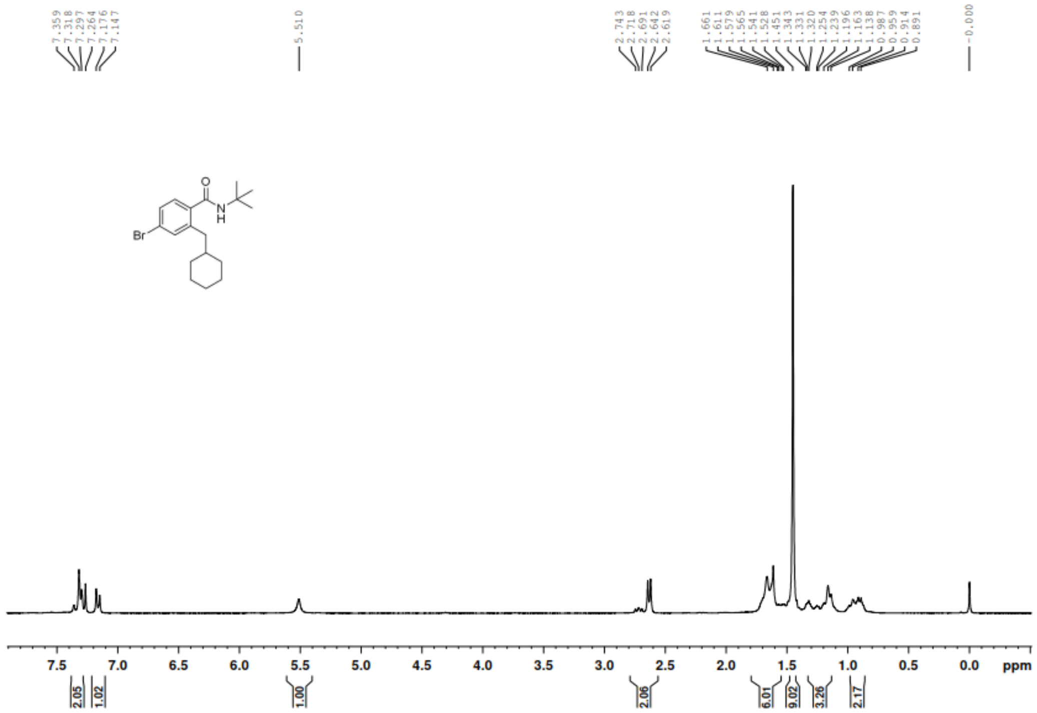

Supplementary Figure 157. ^{1}H NMR (300 MHz, $CDCl_3$) spectra for compound **8k.**

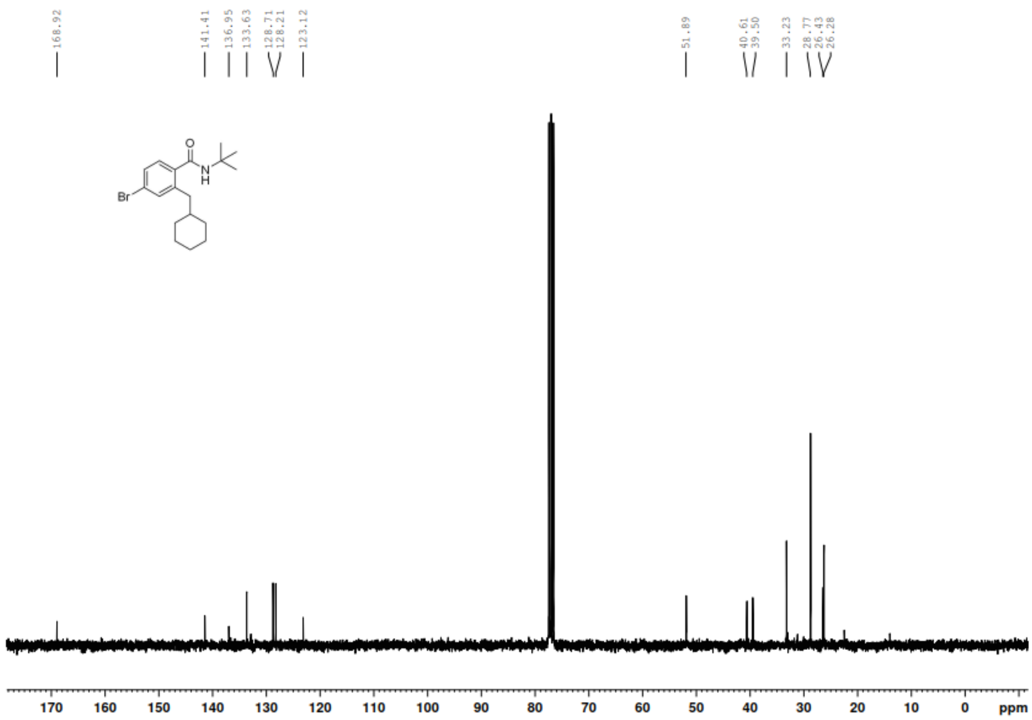

Supplementary Figure 158. ^{13}C NMR (75 MHz, $CDCl_3$) spectra for compound **8k.**

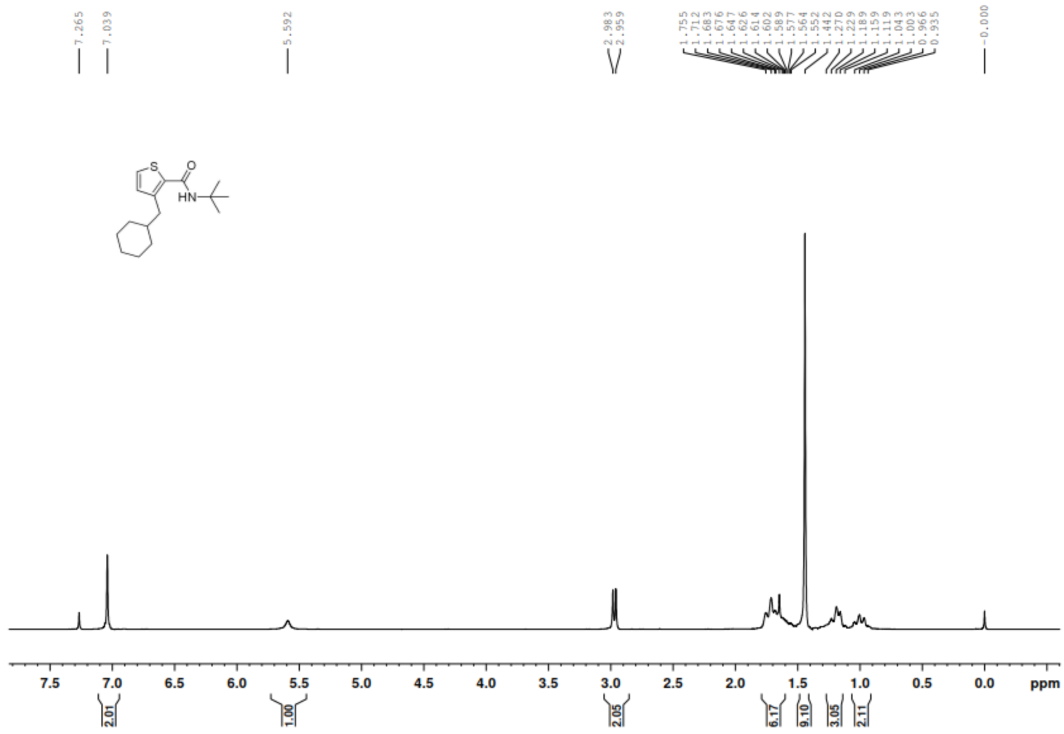

Supplementary Figure 159. ^{1}H NMR (300 MHz, $CDCl_3$) spectra for compound **8l.**

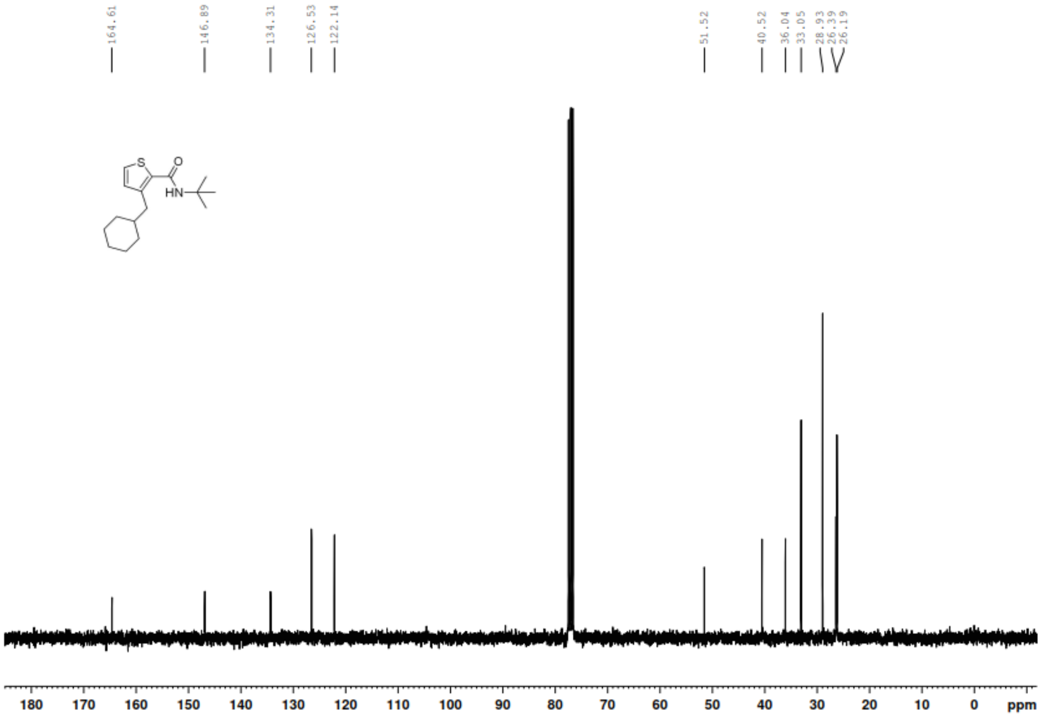

Supplementary Figure 160. ^{13}C NMR (75 MHz, $CDCl_3$) spectra for compound **8l.**

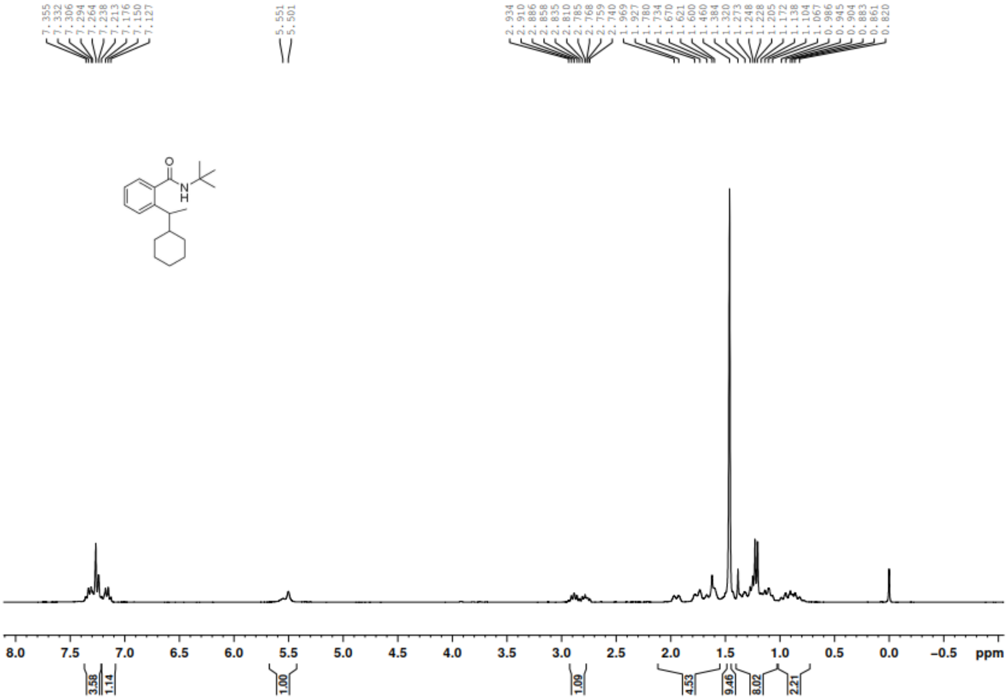

Supplementary Figure 161. ^{1}H NMR (300 MHz, $CDCl_3$) spectra for compound **8m.**

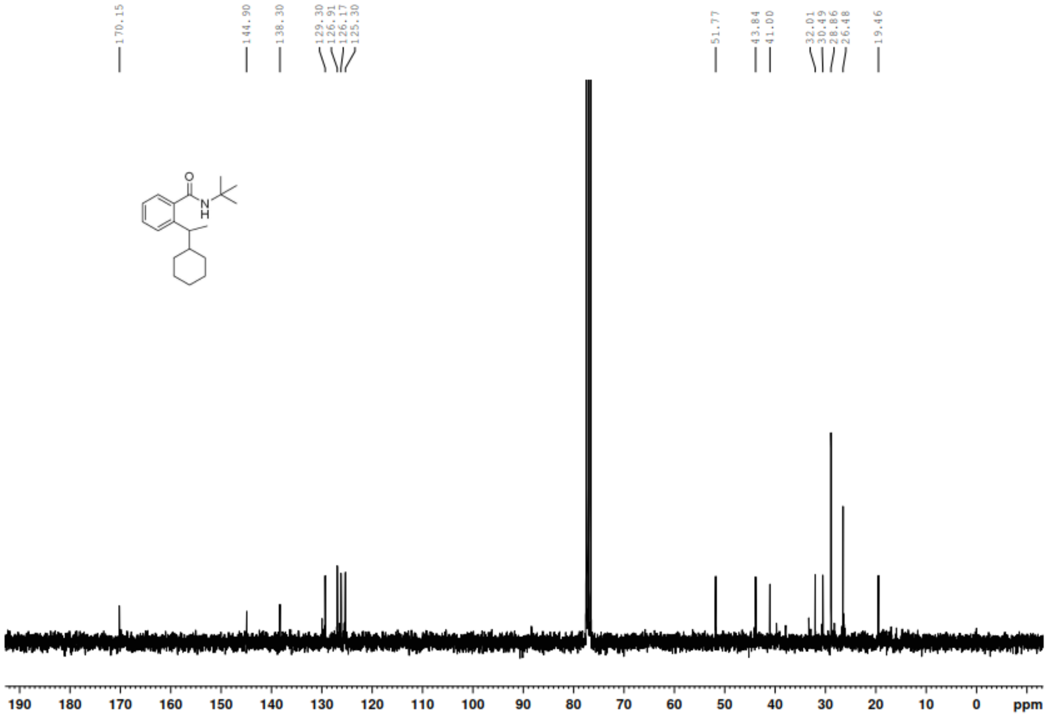

Supplementary Figure 162. ^{13}C NMR (75 MHz, $CDCl_3$) spectra for compound **8m.**

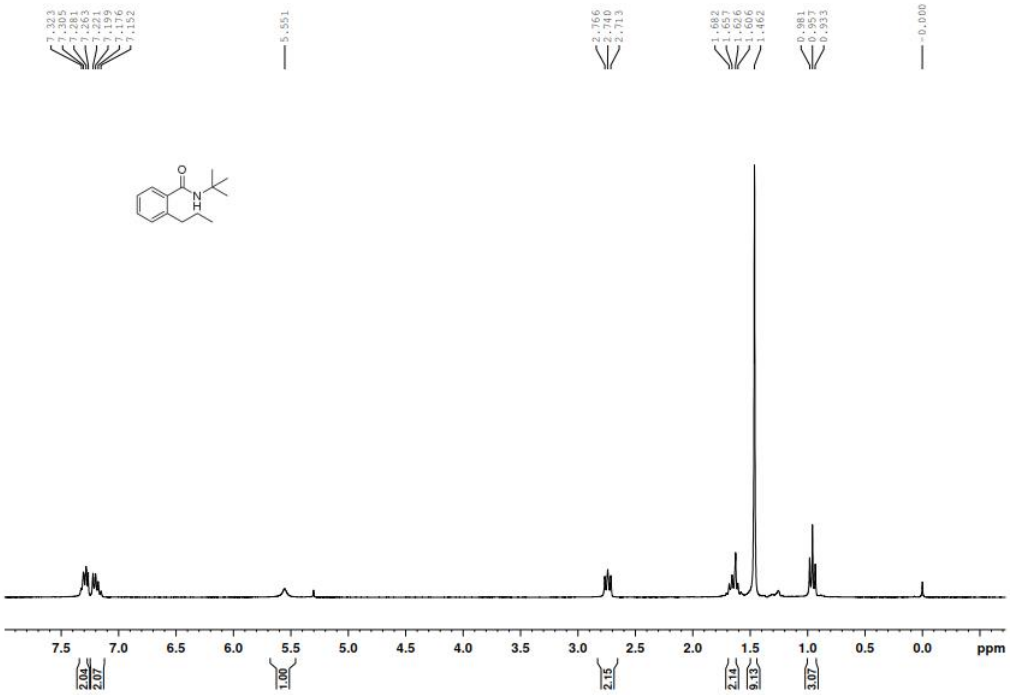

Supplementary Figure 163. ^{1}H NMR (300 MHz, $CDCl_3$) spectra for compound **9a.**

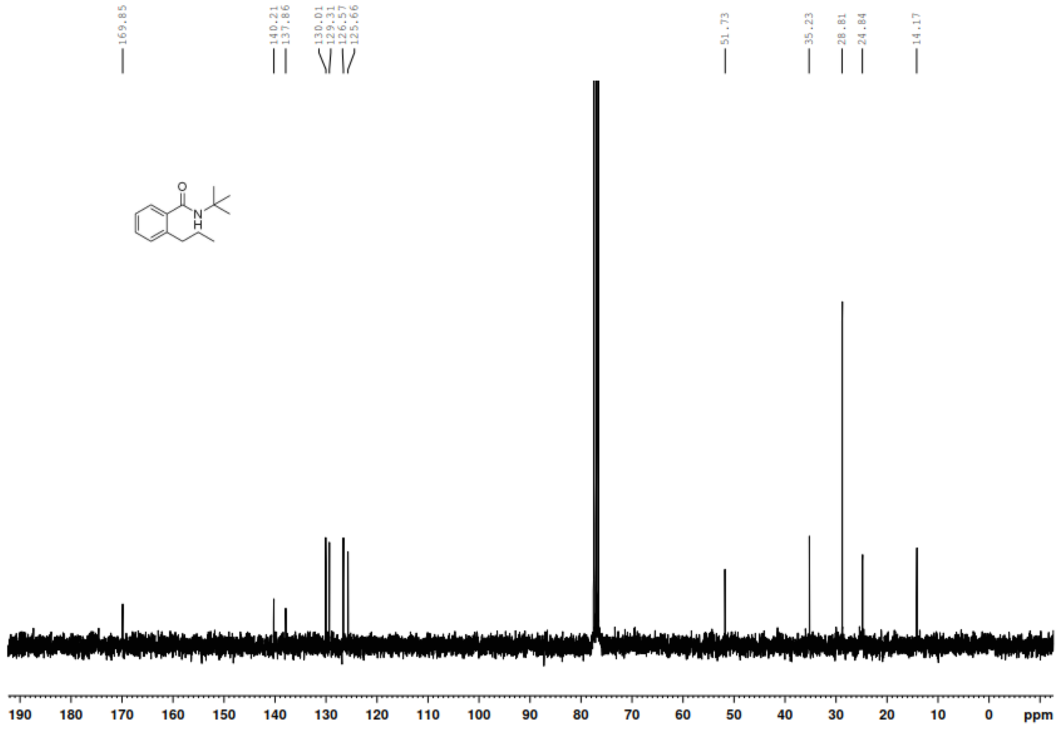

Supplementary Figure 164. ^{13}C NMR (75 MHz, $CDCl_3$) spectra for compound **9a.**

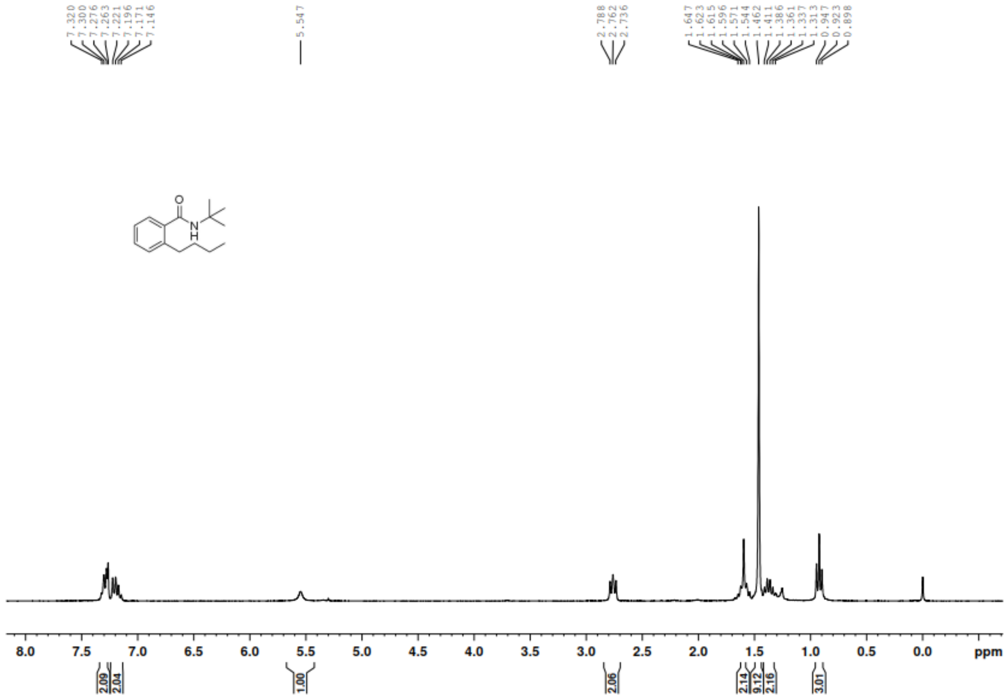

Supplementary Figure 165. ^{1}H NMR (300 MHz, $CDCl_3$) spectra for compound **9b.**

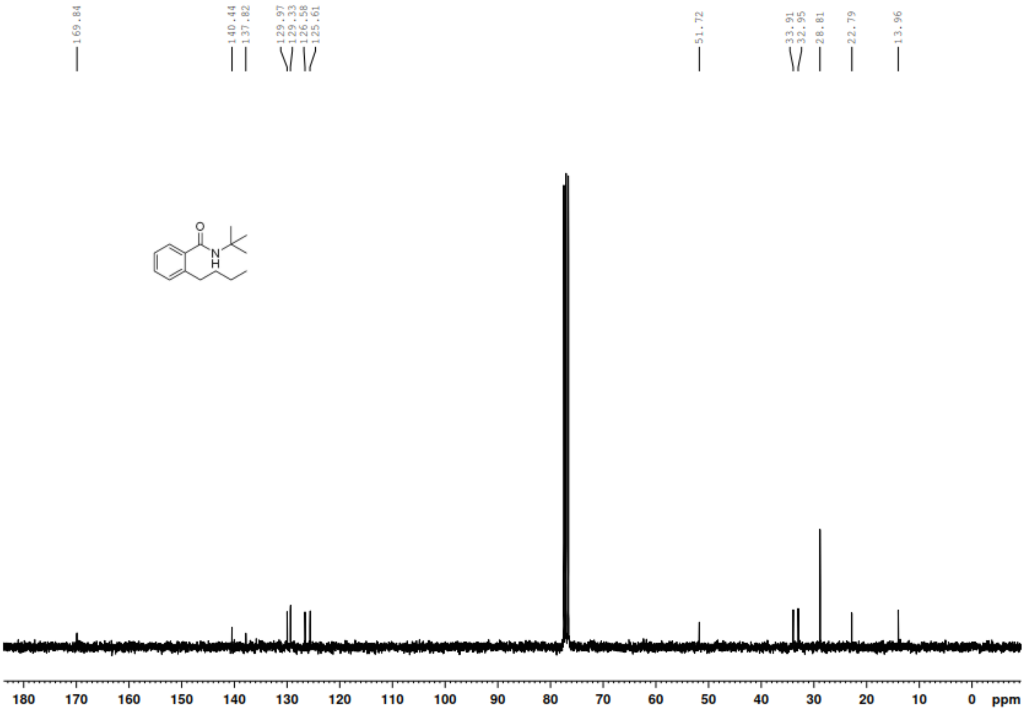

Supplementary Figure 166. ^{13}C NMR (75 MHz, $CDCl_3$) spectra for compound **9b.**

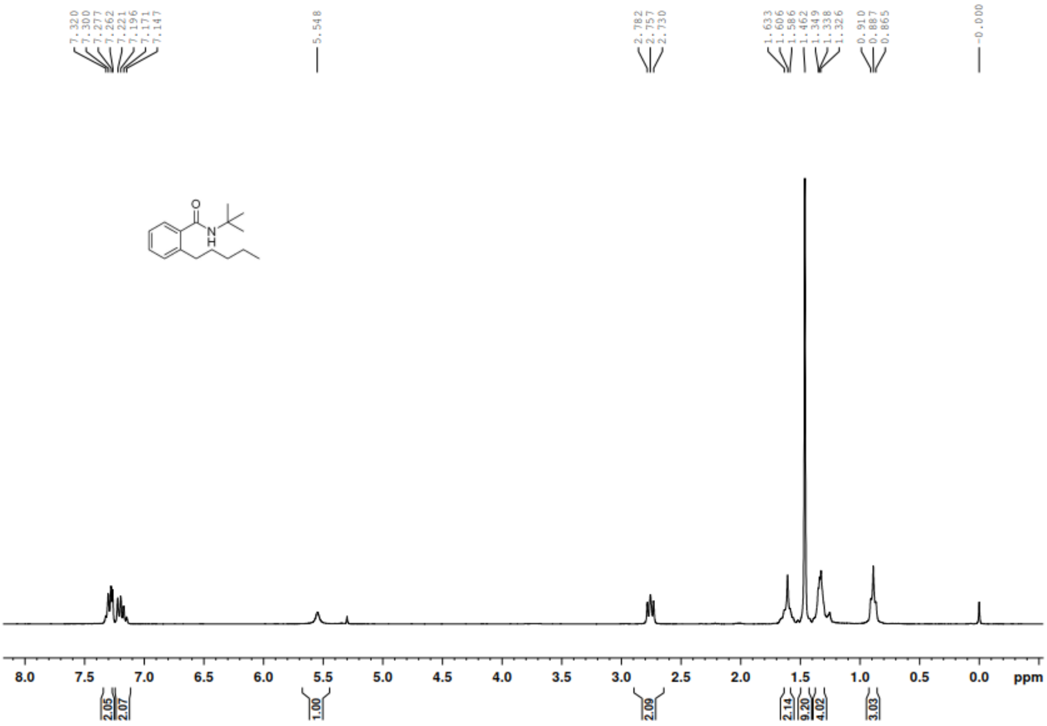

Supplementary Figure 167. ^{1}H NMR (300 MHz, $CDCl_3$) spectra for compound **9c.**

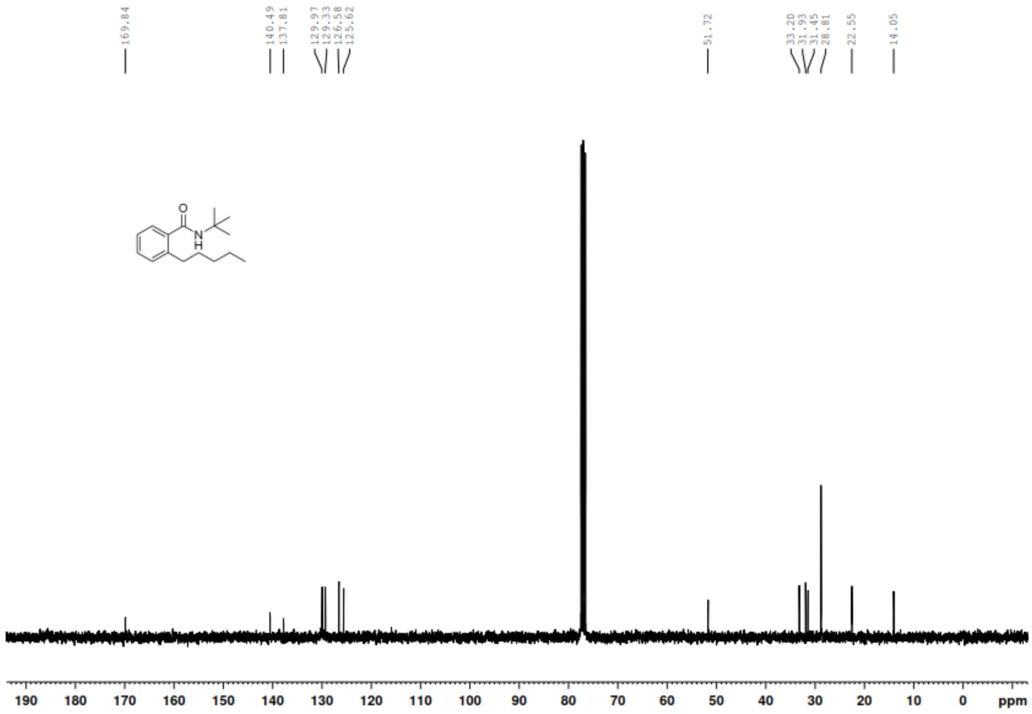

Supplementary Figure 168. ^{13}C NMR (75 MHz, $CDCl_3$) spectra for compound **9c.**

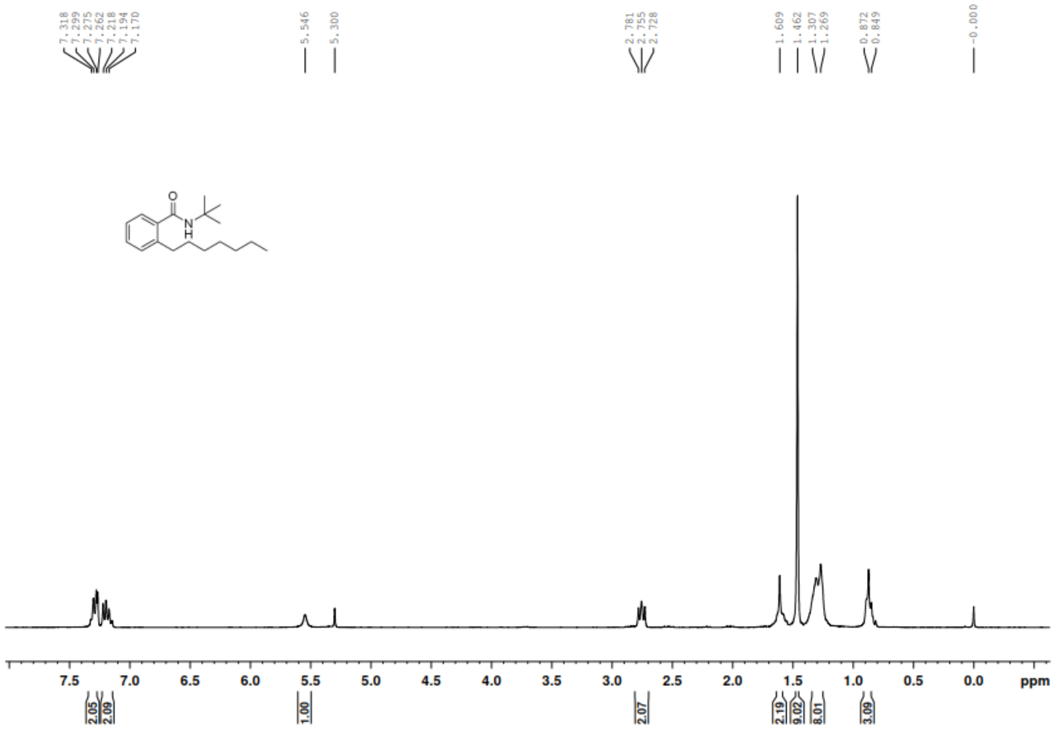

Supplementary Figure 169. ^{1}H NMR (300 MHz, $CDCl_3$) spectra for compound **9d.**

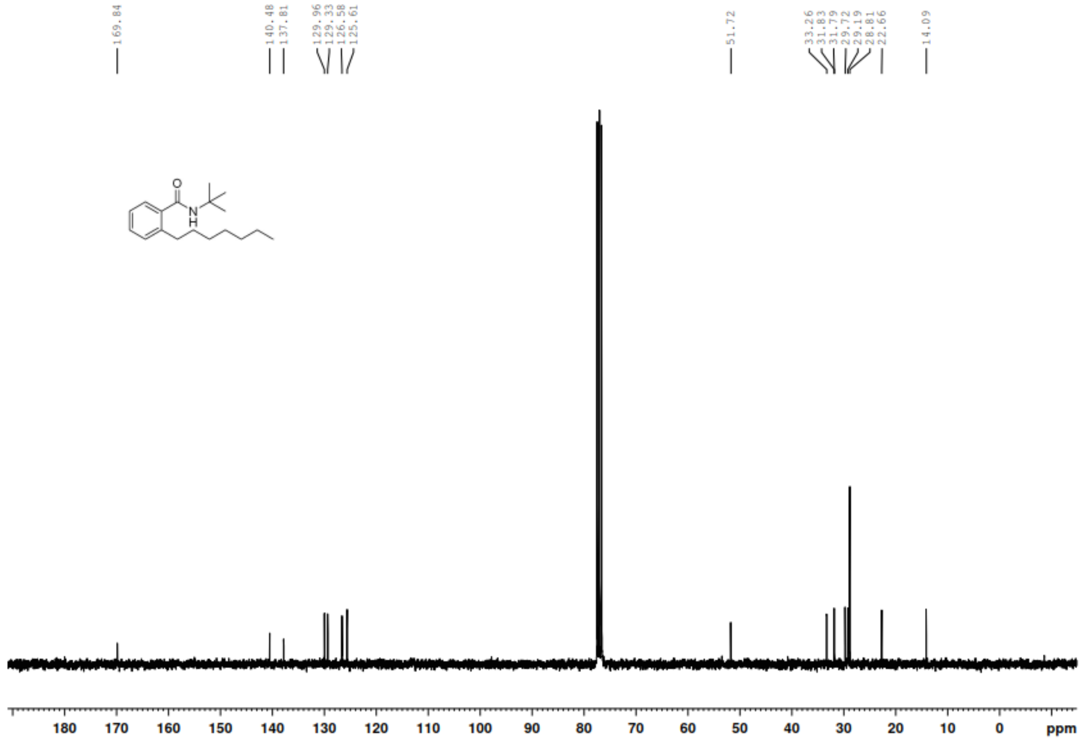

Supplementary Figure 170. ^{13}C NMR (75 MHz, $CDCl_3$) spectra for compound **9d.**

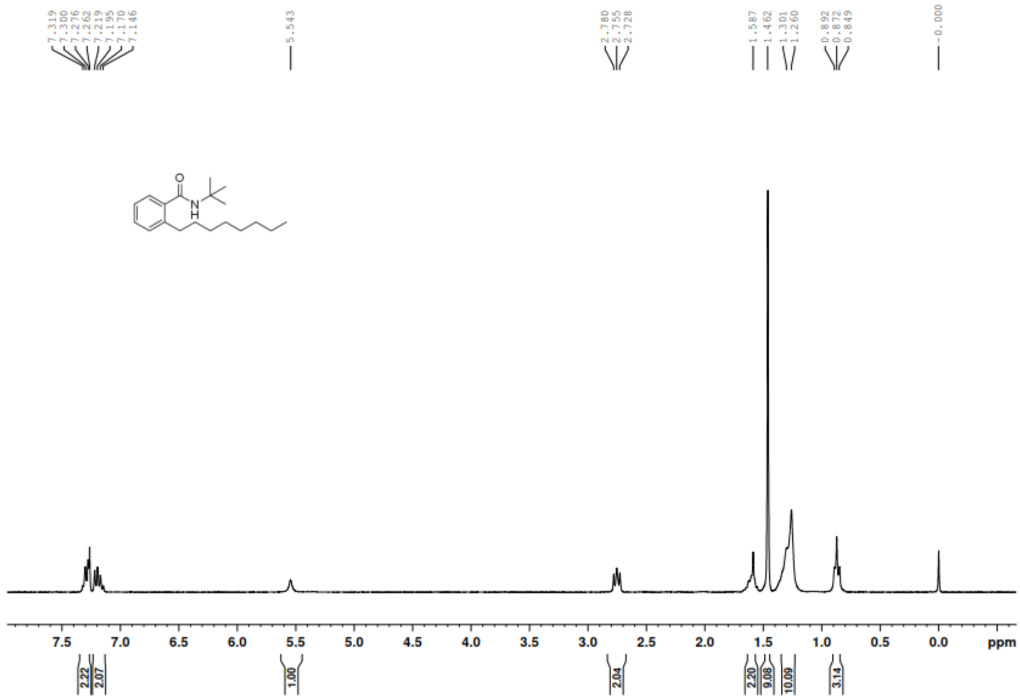

Supplementary Figure 171. ^{1}H NMR (300 MHz, $CDCl_3$) spectra for compound **9e.**

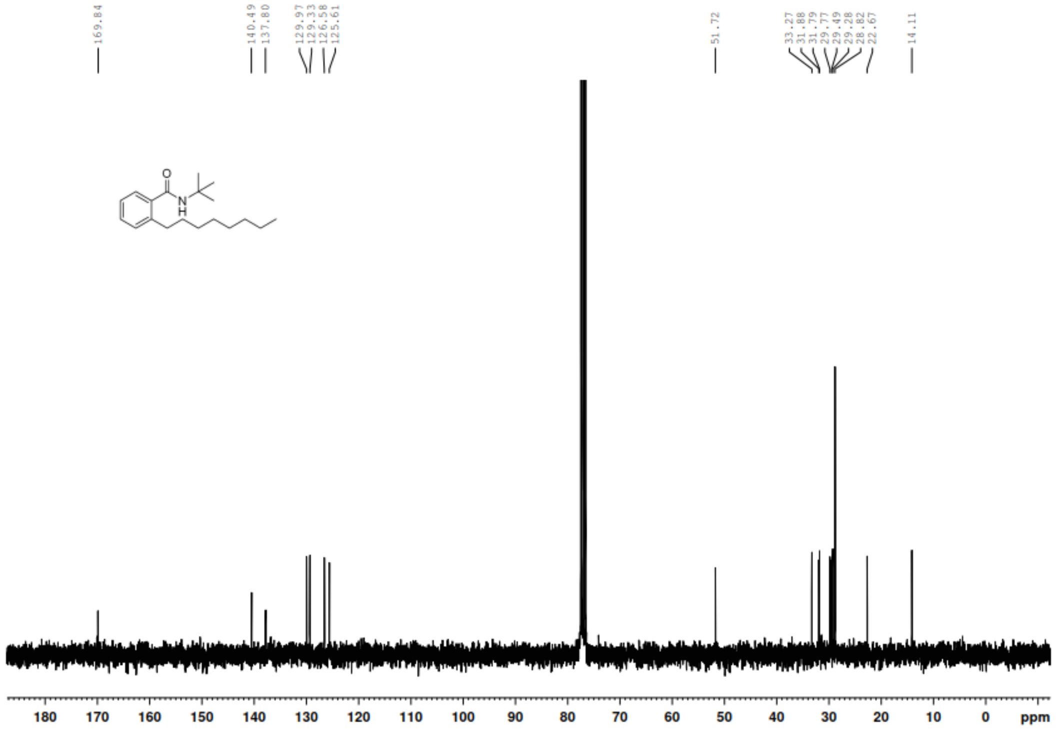

Supplementary Figure 172. ^{13}C NMR (75 MHz, $CDCl_3$) spectra for compound **9e.**

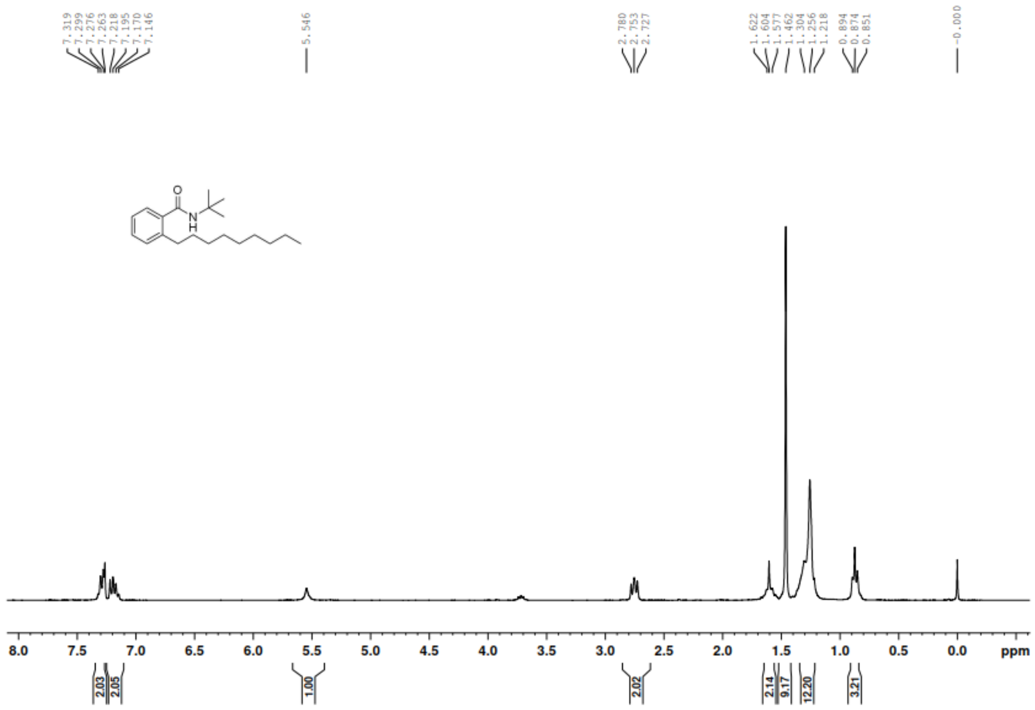

Supplementary Figure 173. ^{1}H NMR (300 MHz, $CDCl_3$) spectra for compound **9f.**

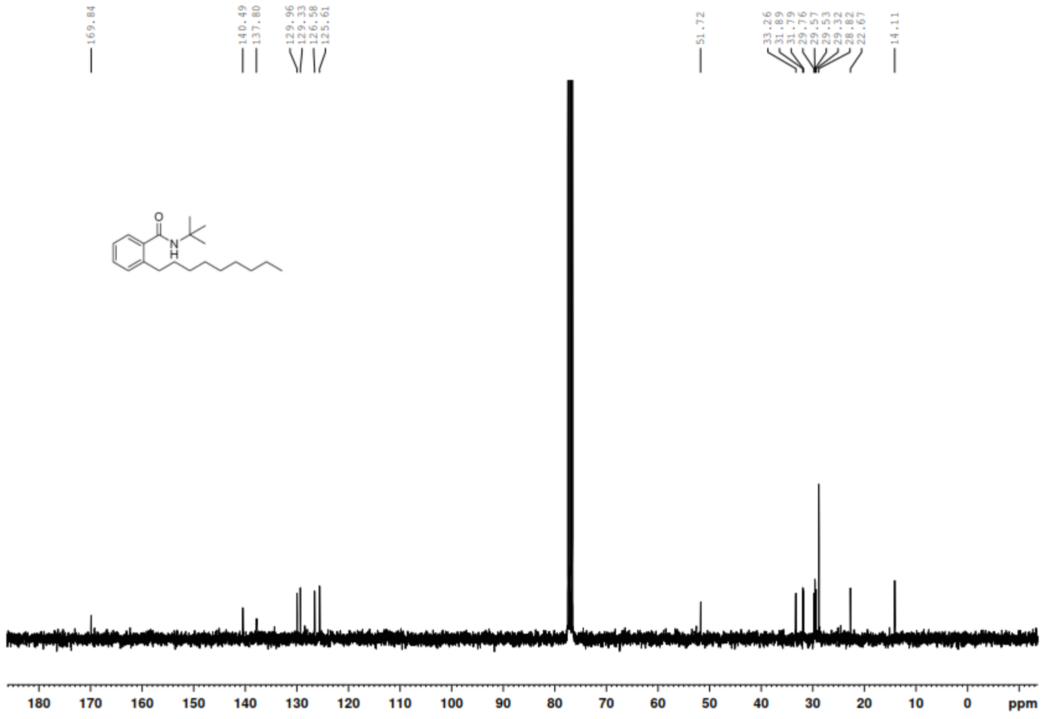

Supplementary Figure 174. ^{13}C NMR (75 MHz, $CDCl_3$) spectra for compound **9f.**

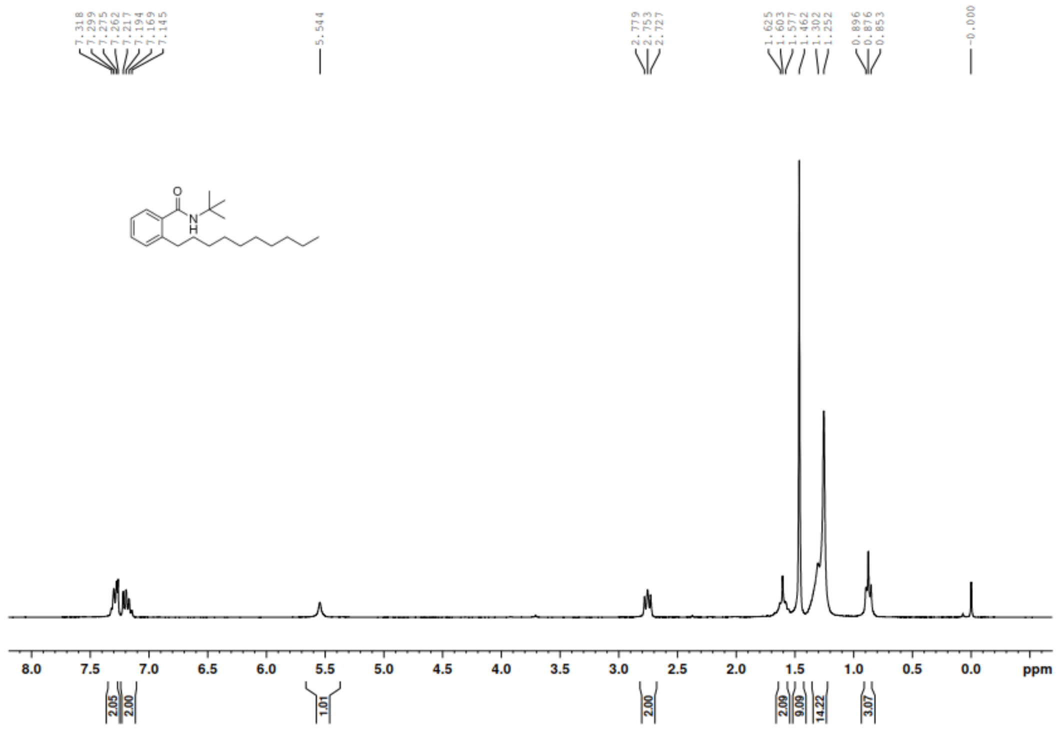

Supplementary Figure 175. ^{1}H NMR (300 MHz, $CDCl_3$) spectra for compound **9g.**

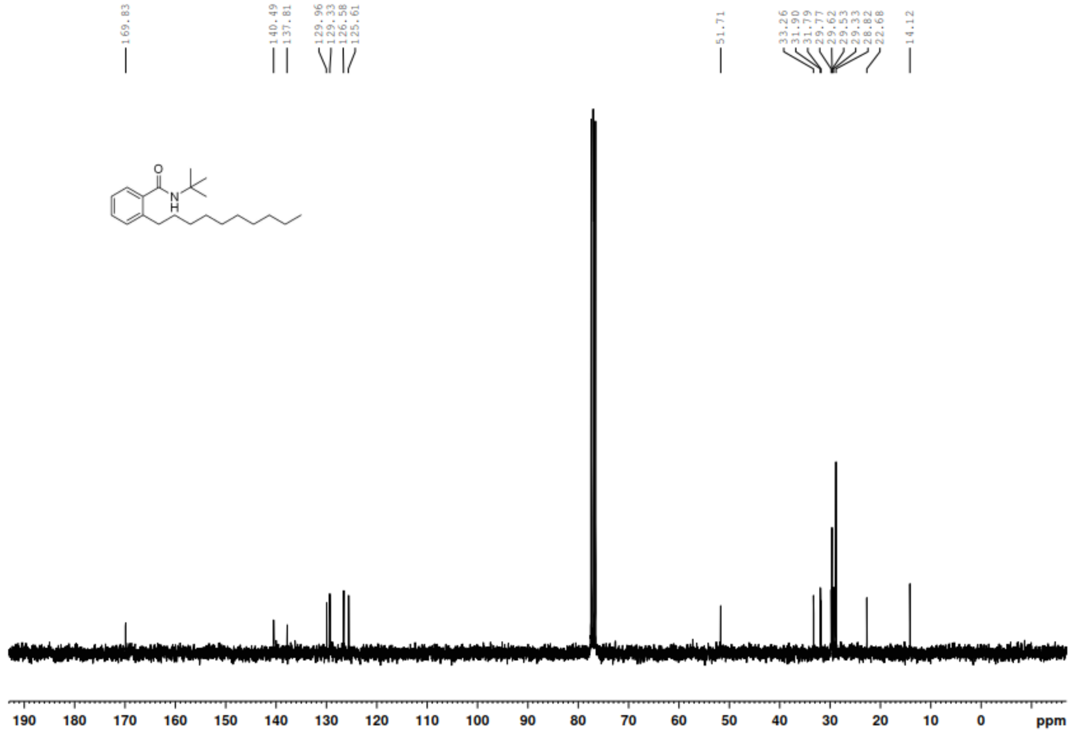

Supplementary Figure 176. ^{13}C NMR (75 MHz, $CDCl_3$) spectra for compound **9g.**

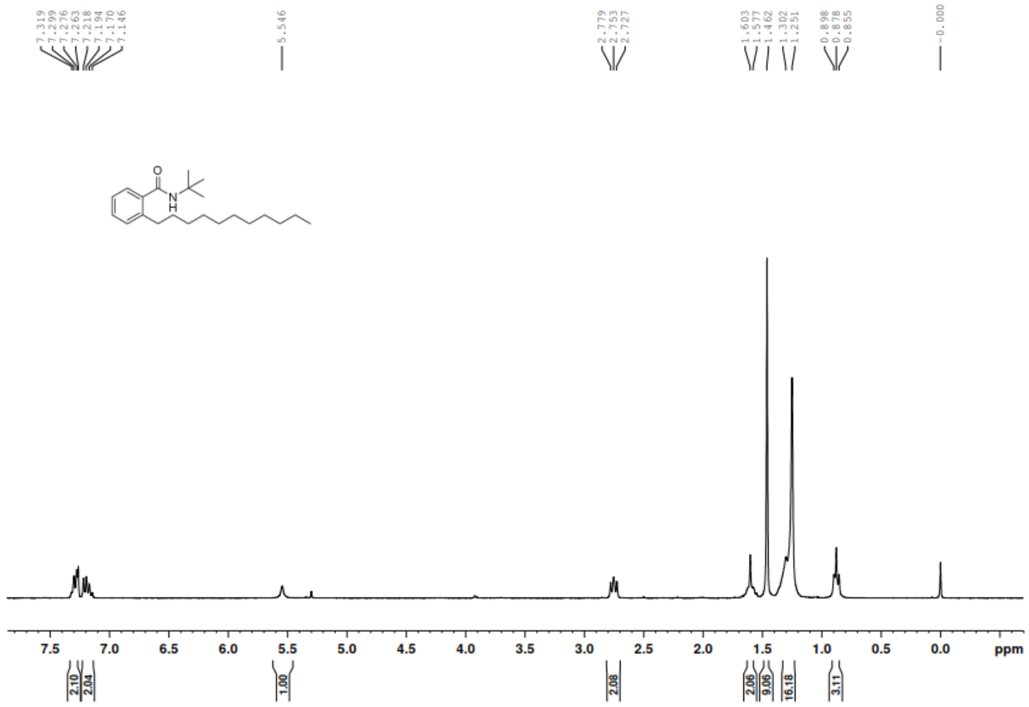

Supplementary Figure 177. ^{1}H NMR (300 MHz, $CDCl_3$) spectra for compound **9h.**

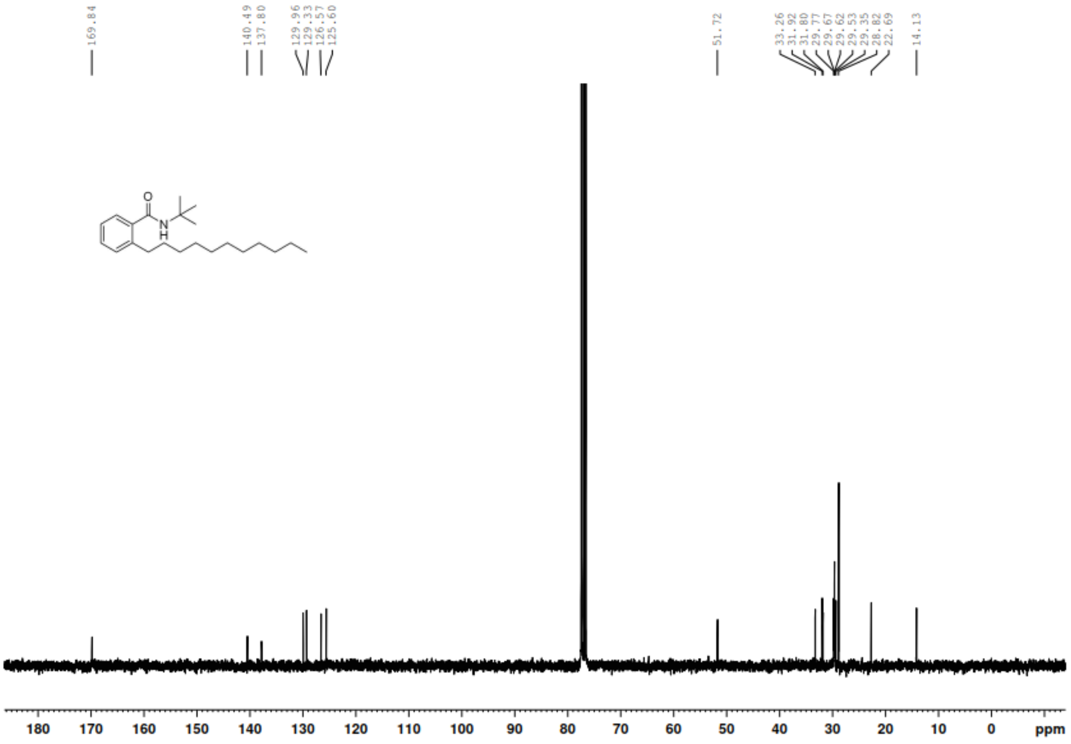

Supplementary Figure 178. ^{13}C NMR (75 MHz, $CDCl_3$) spectra for compound **9h.**

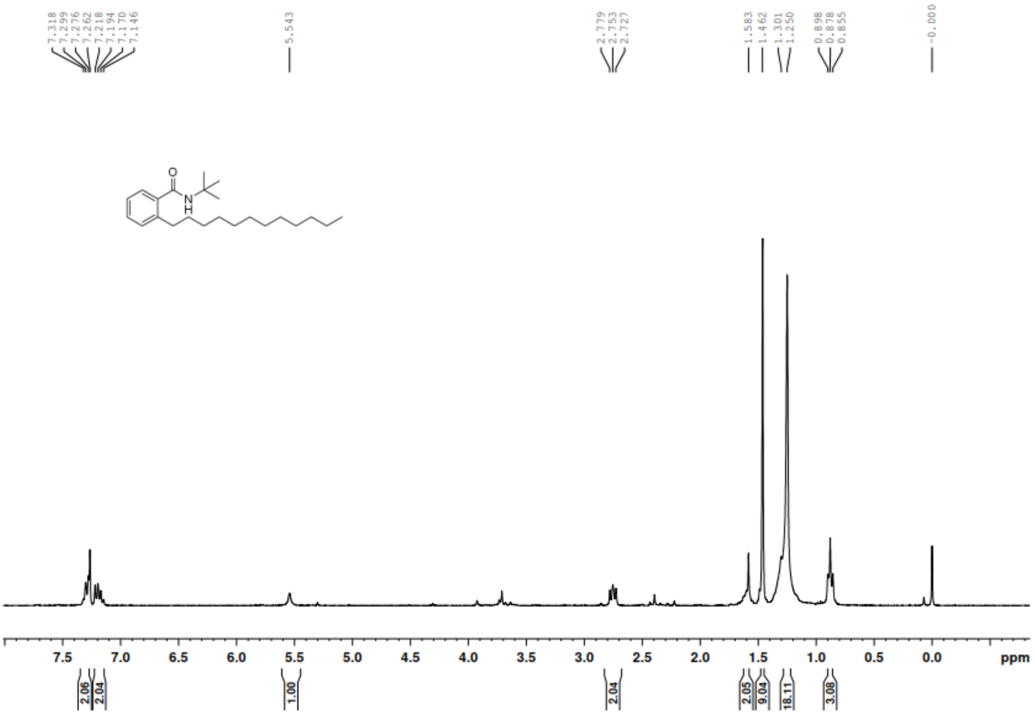

Supplementary Figure 179. ^{1}H NMR (300 MHz, $CDCl_3$) spectra for compound **9i.**

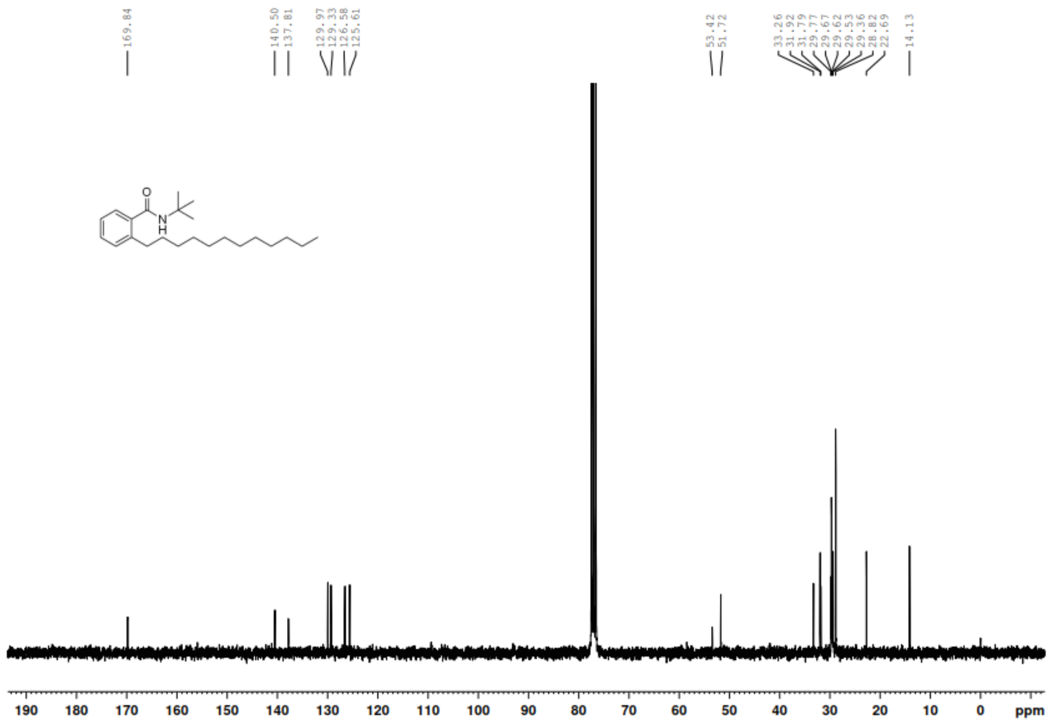

Supplementary Figure 180. ^{13}C NMR (75 MHz, $CDCl_3$) spectra for compound **9i.**

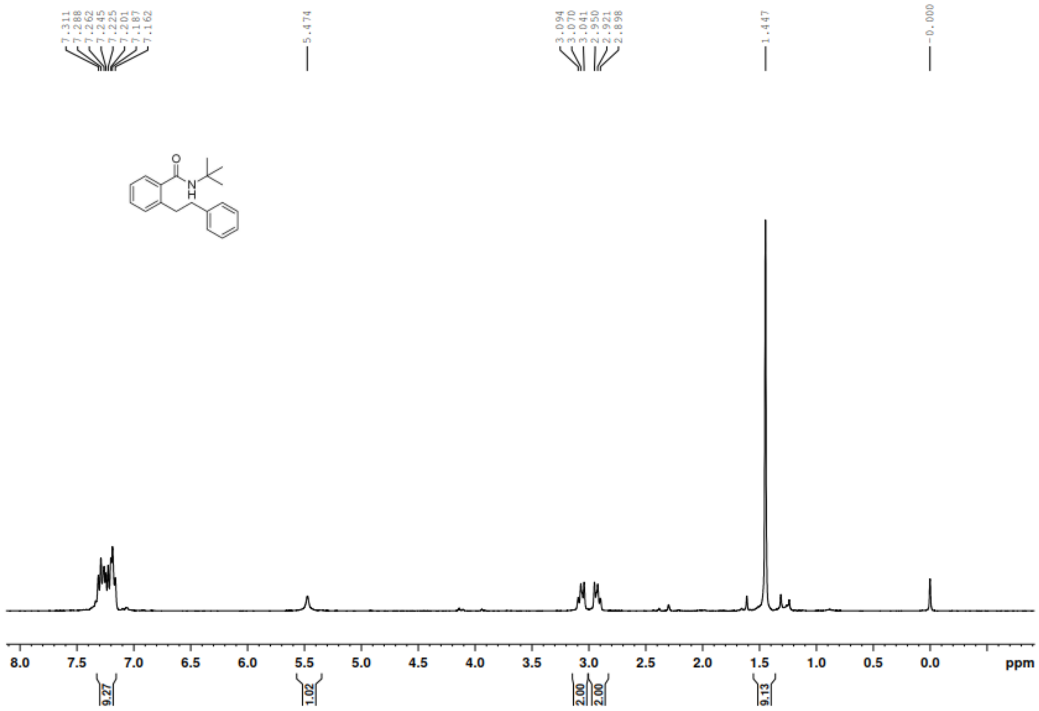

Supplementary Figure 181. ^{1}H NMR (300 MHz, $CDCl_3$) spectra for compound **9j.**

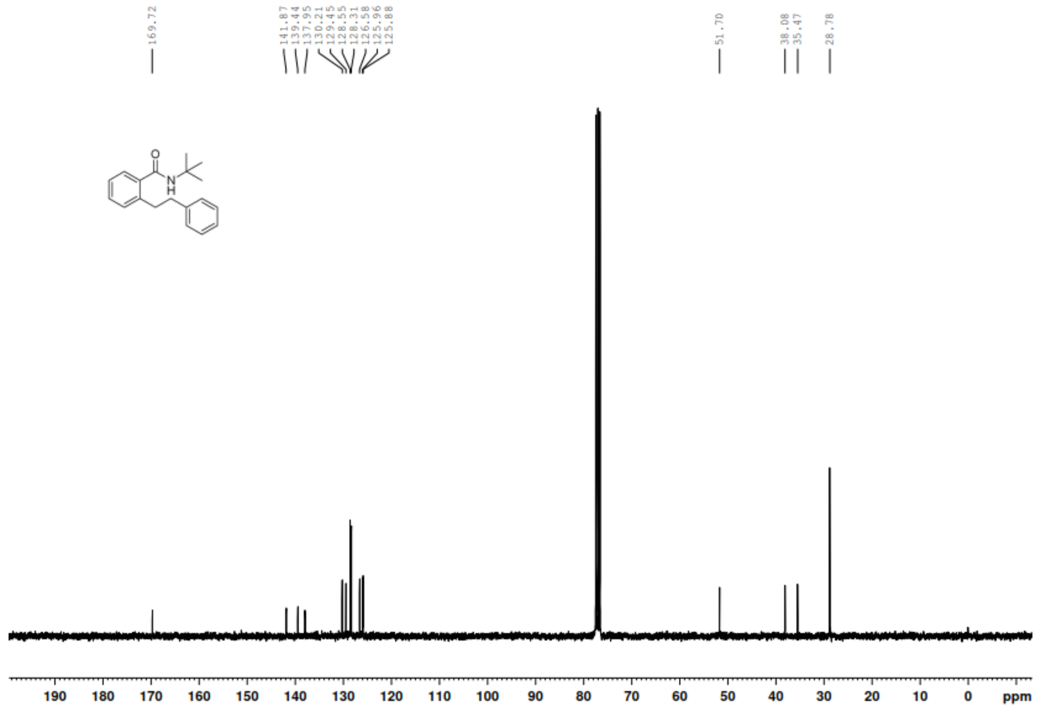

Supplementary Figure 182. ^{13}C NMR (75 MHz, $CDCl_3$) spectra for compound **9j.**

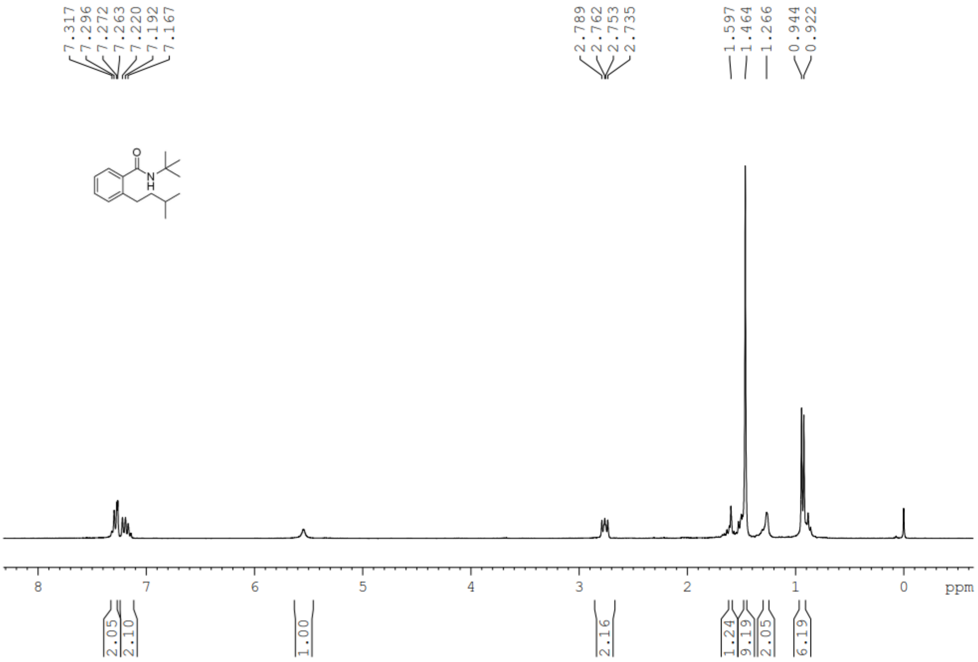

Supplementary Figure 183. ^{1}H NMR (300 MHz, $CDCl_3$) spectra for compound **9k.**

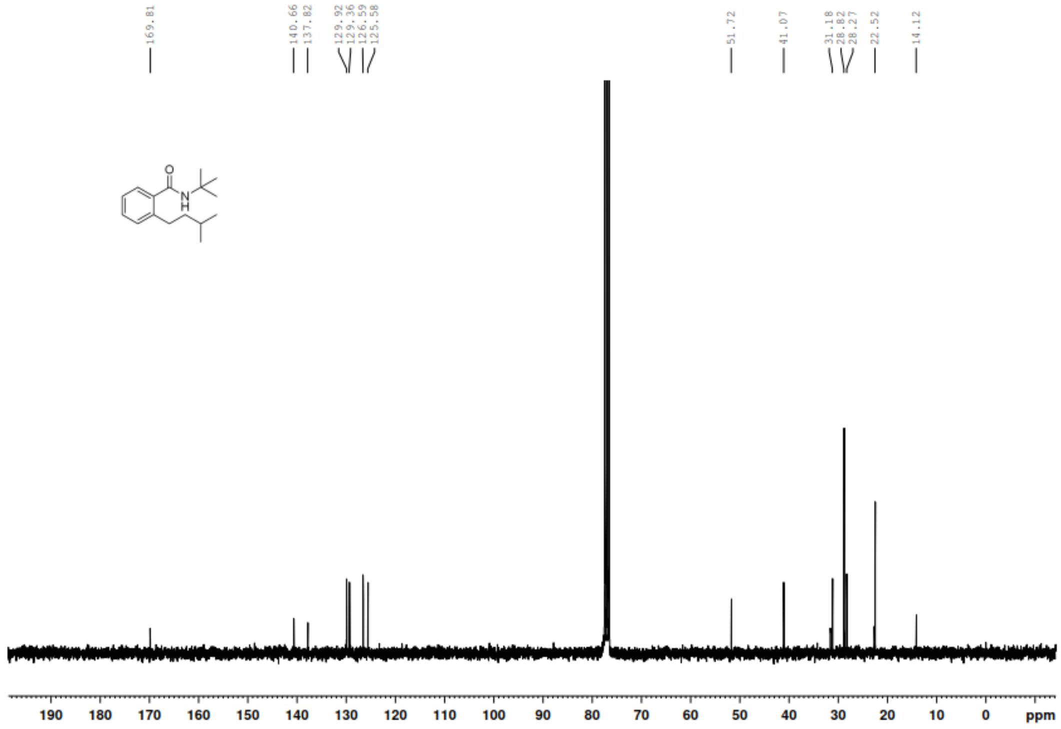

Supplementary Figure 184. ^{13}C NMR (75 MHz, $CDCl_3$) spectra for compound **9k.**

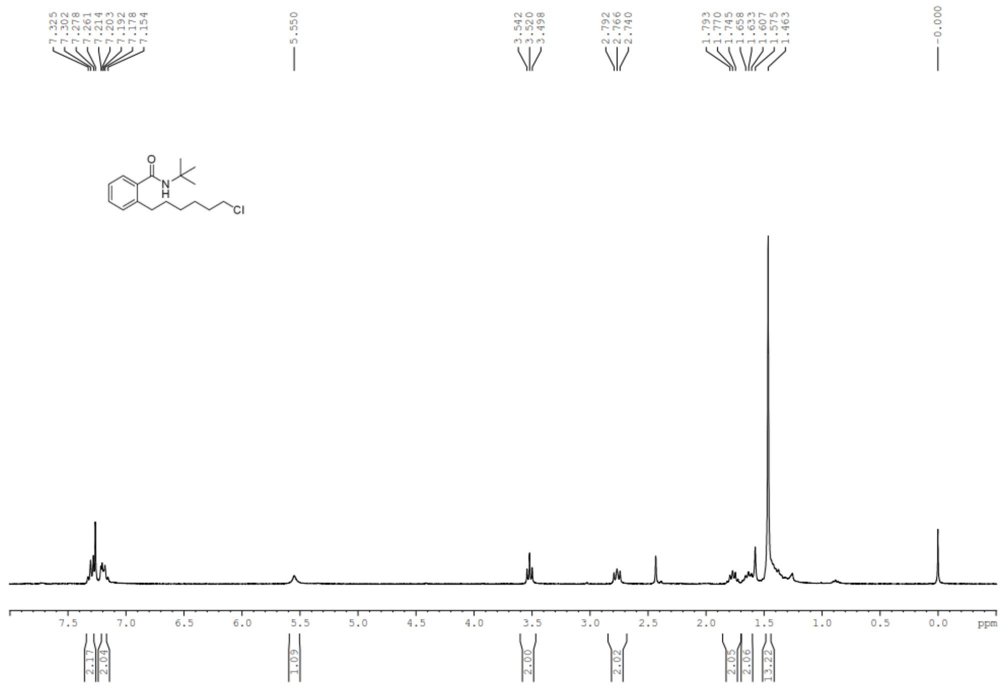

Supplementary Figure 185. ^{1}H NMR (300 MHz, $CDCl_3$) spectra for compound **9l.**

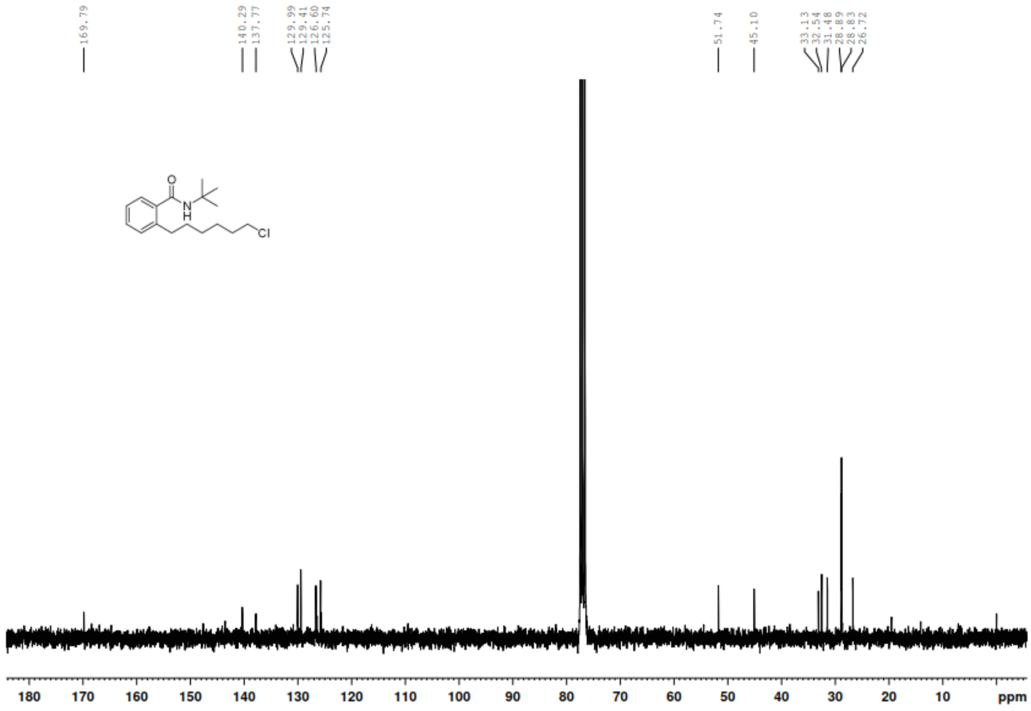

Supplementary Figure 186. ^{13}C NMR (75 MHz, $CDCl_3$) spectra for compound **9l.**

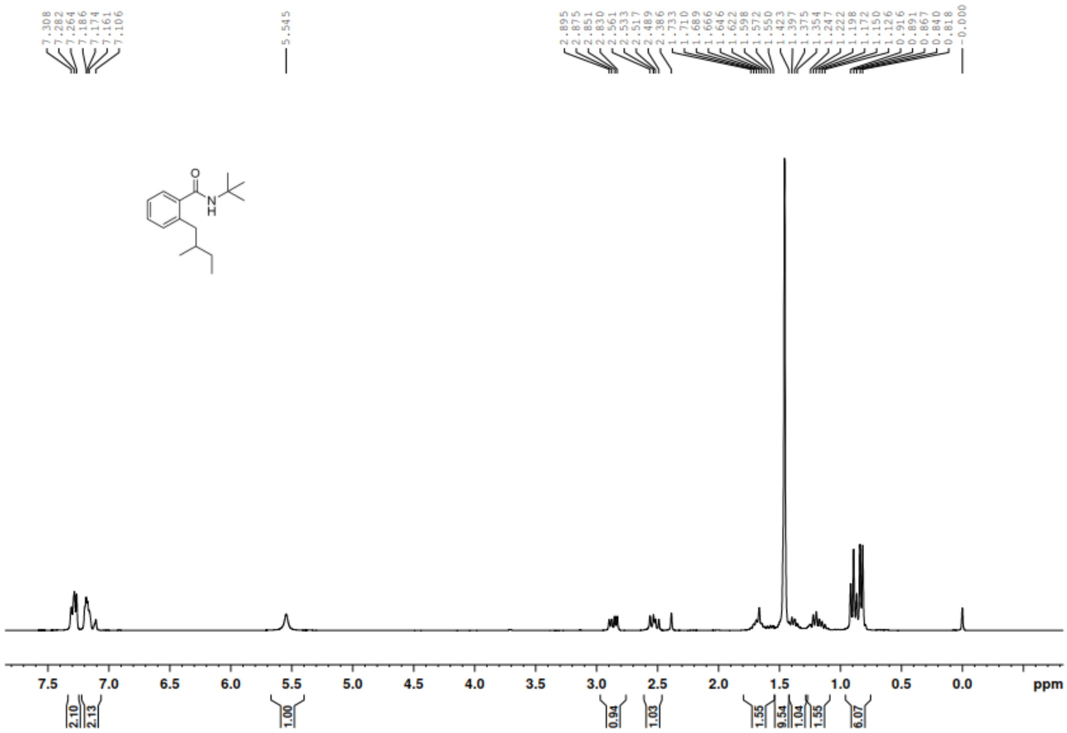

Supplementary Figure 187. ^{1}H NMR (300 MHz, $CDCl_3$) spectra for compound **10a.**

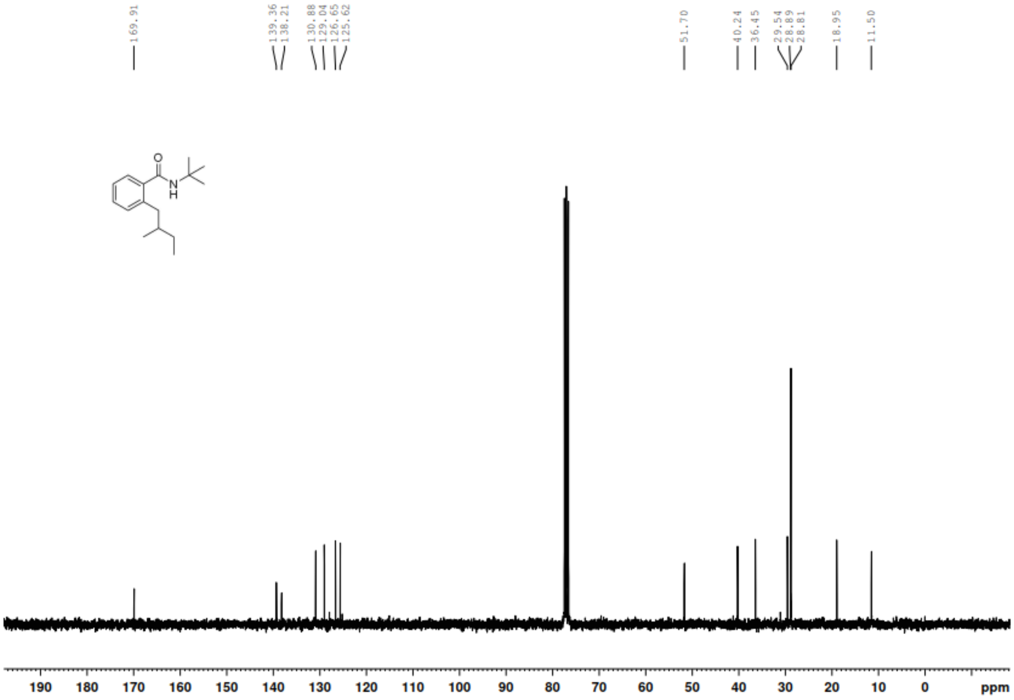

Supplementary Figure 188. ^{13}C NMR (75 MHz, $CDCl_3$) spectra for compound **10a.**

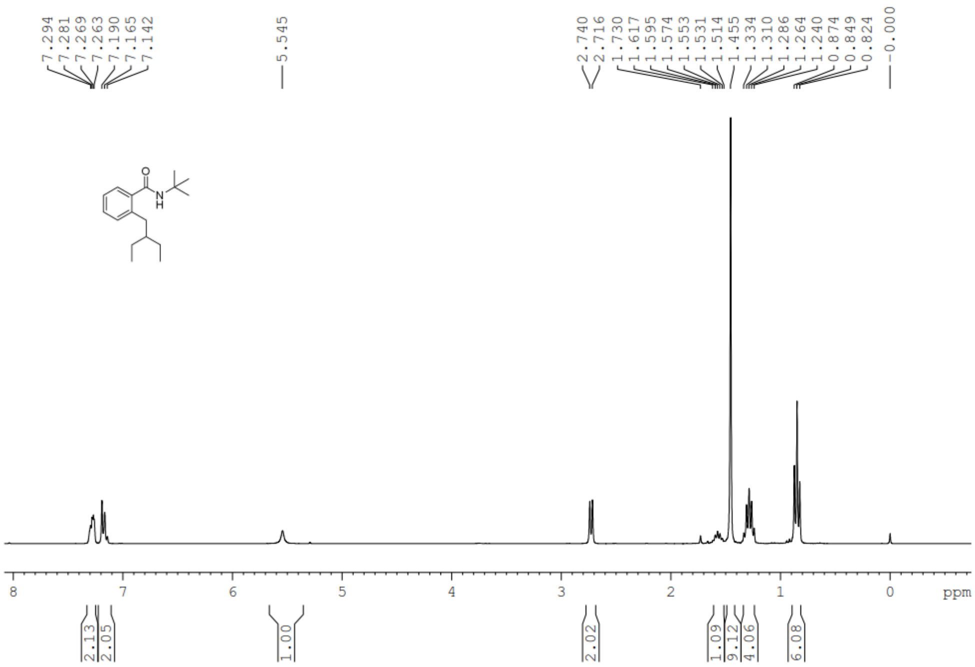

Supplementary Figure 189. ^{1}H NMR (300 MHz, $CDCl_3$) spectra for compound **10b.**

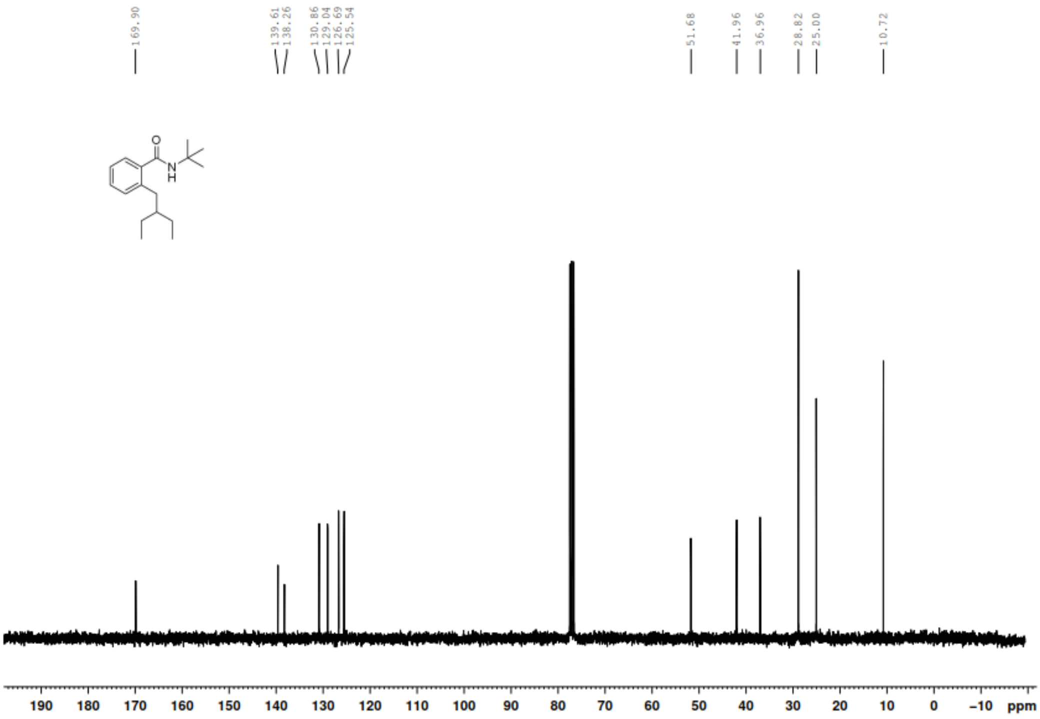

Supplementary Figure 190. ^{13}C NMR (75 MHz, $CDCl_3$) spectra for compound **10b.**

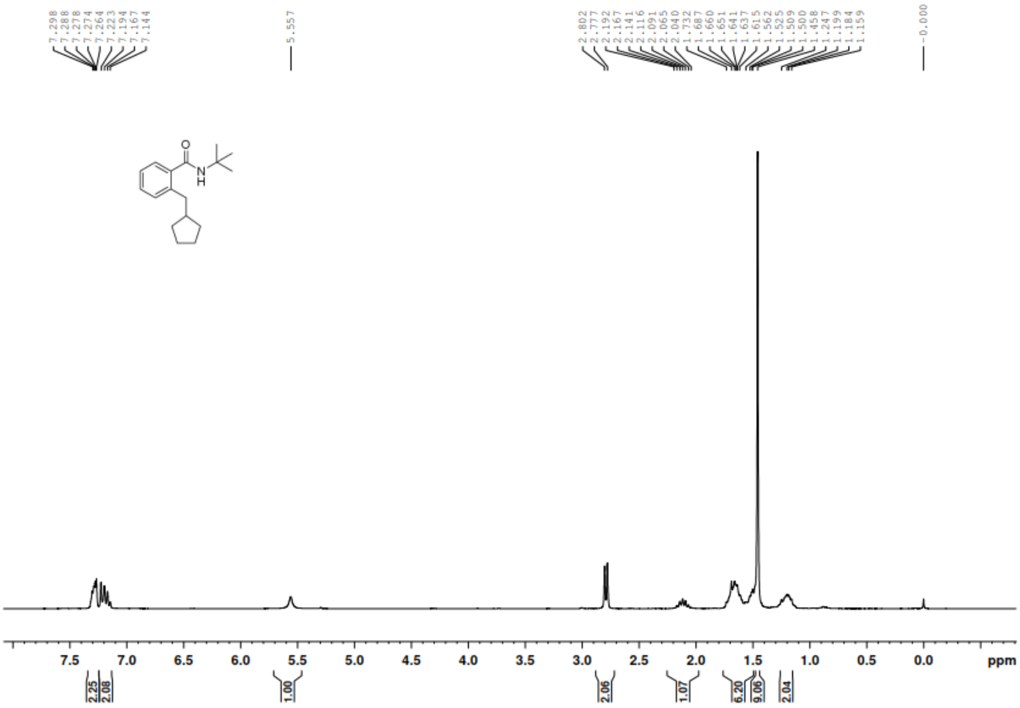

Supplementary Figure 191. ^{1}H NMR (300 MHz, $CDCl_3$) spectra for compound **10c.**

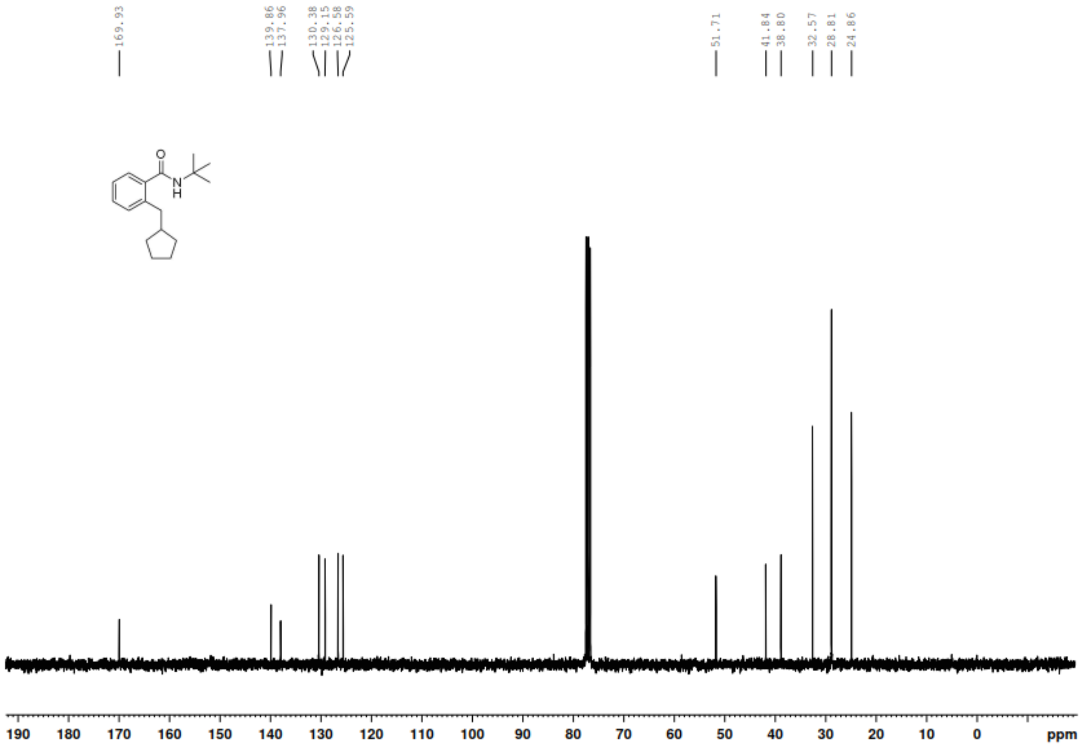

Supplementary Figure 192. ^{13}C NMR (75 MHz, $CDCl_3$) spectra for compound **10c.**

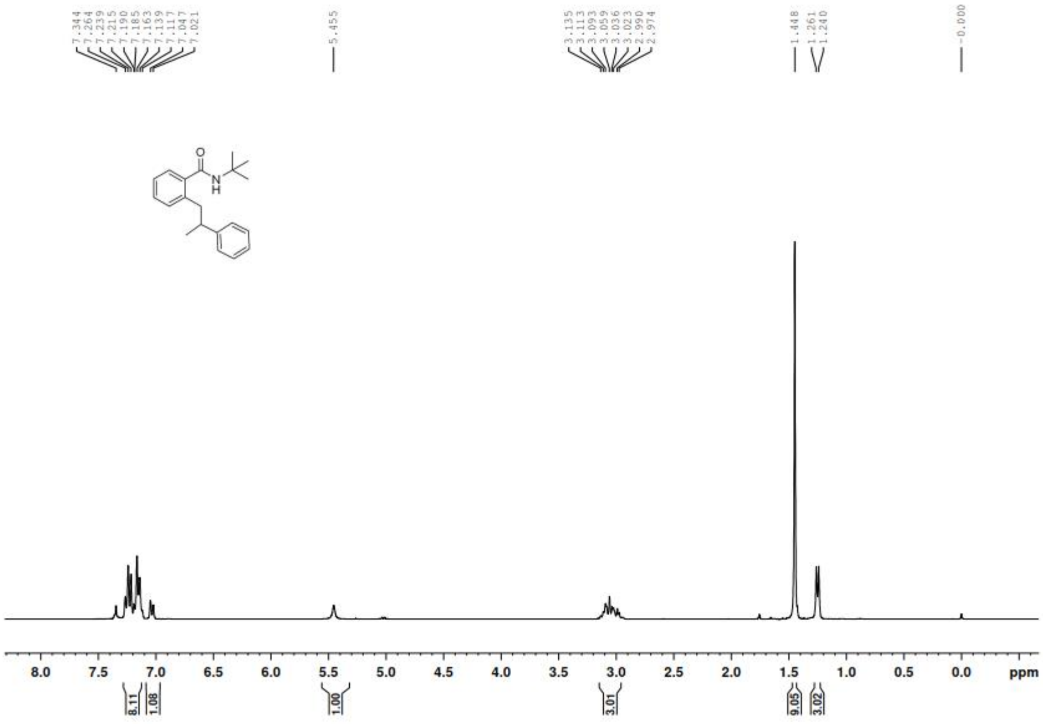

Supplementary Figure 193. ^{1}H NMR (300 MHz, $CDCl_3$) spectra for compound **10d.**

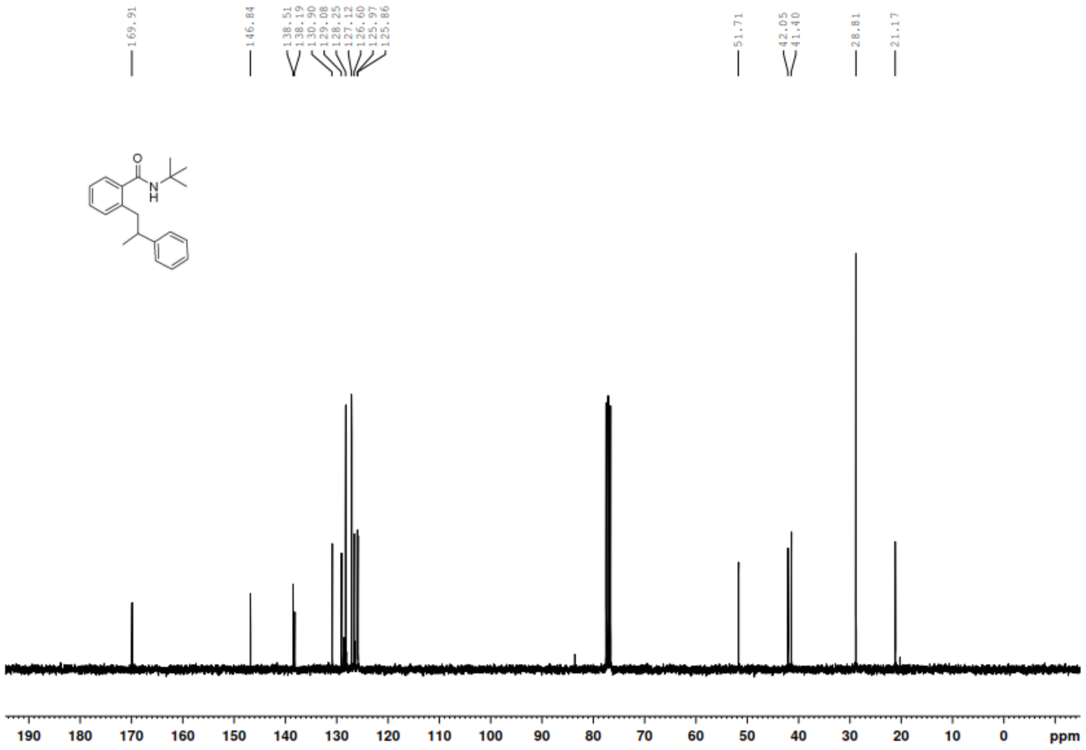

Supplementary Figure 194. ^{13}C NMR (75 MHz, $CDCl_3$) spectra for compound **10d.**

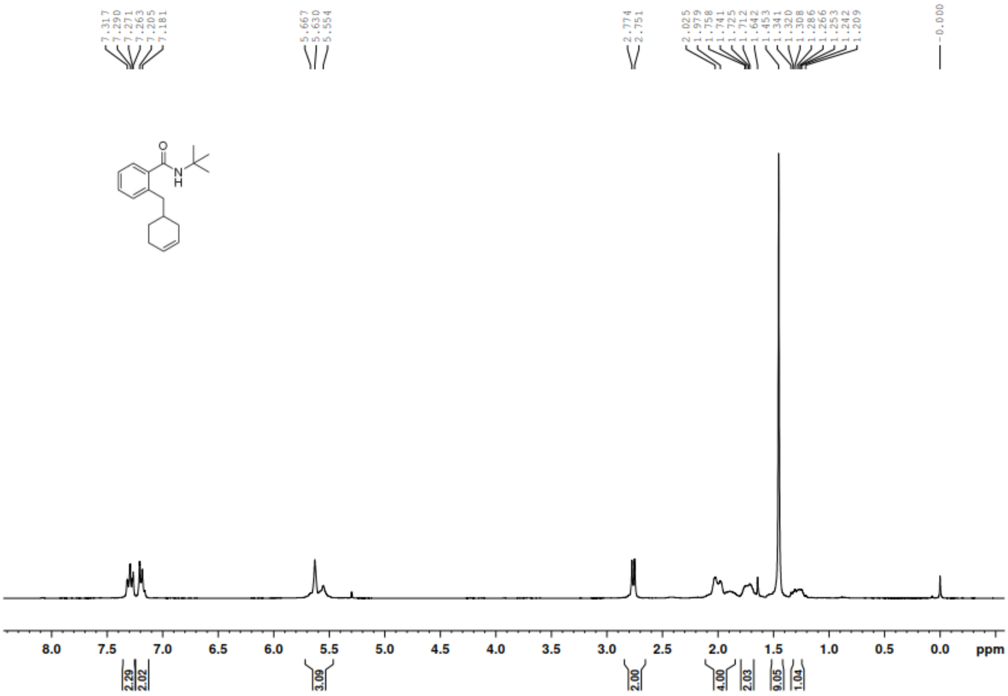

Supplementary Figure 195. ^{1}H NMR (300 MHz, $CDCl_3$) spectra for compound **10e.**

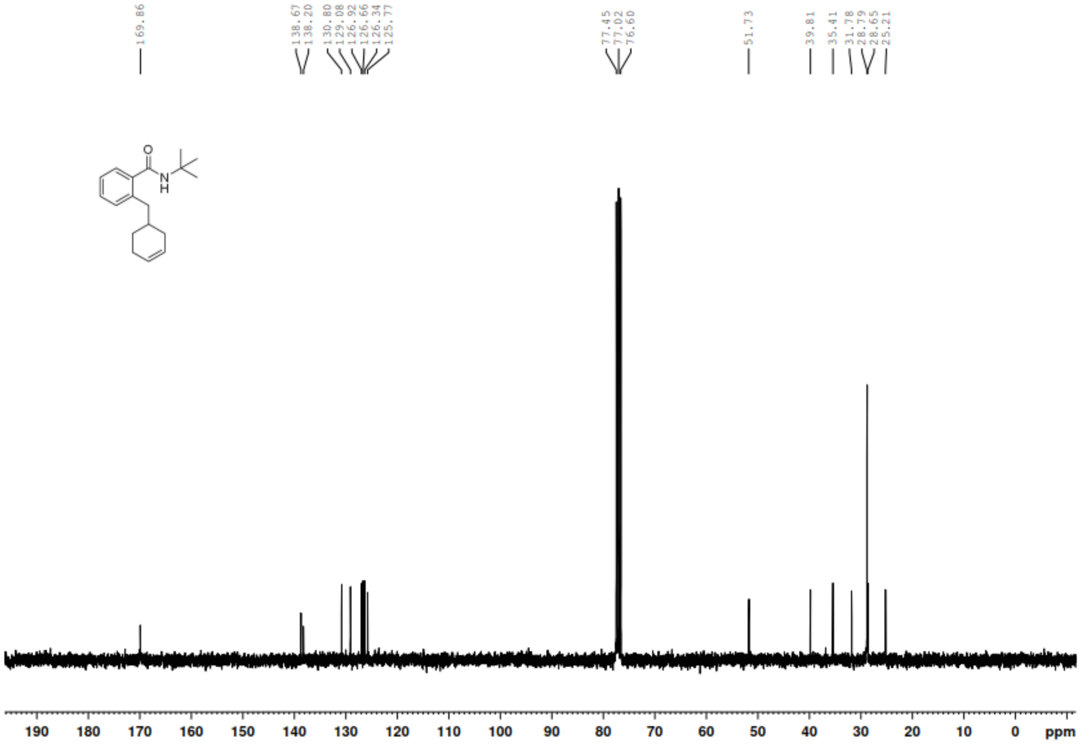

Supplementary Figure 196. ^{13}C NMR (75 MHz, $CDCl_3$) spectra for compound **10e.**

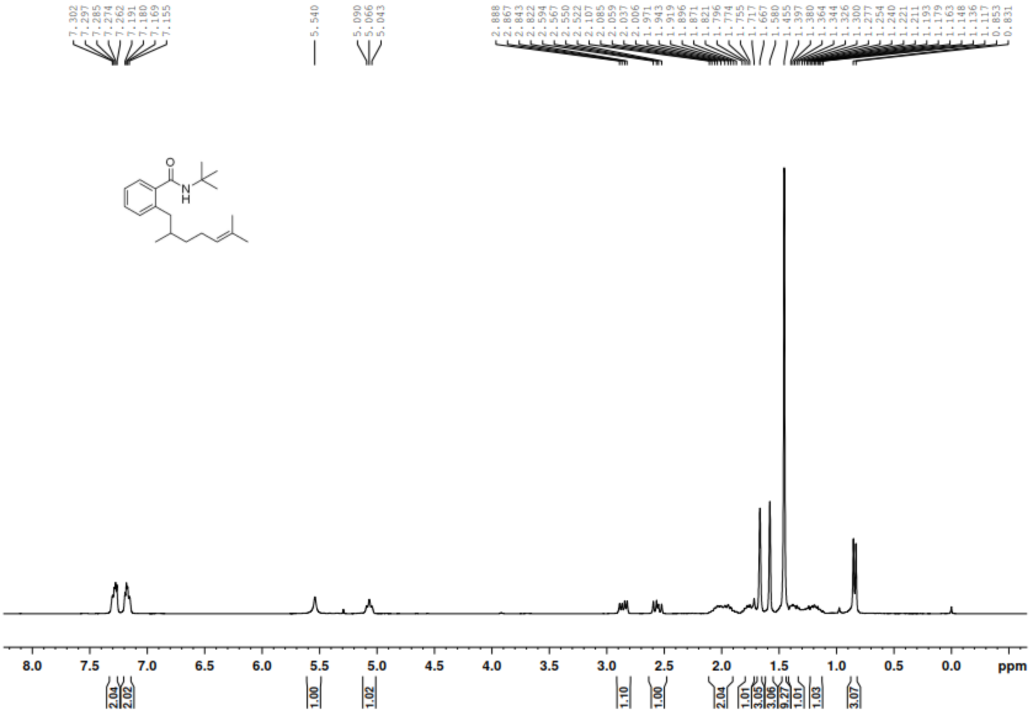

Supplementary Figure 197. ^{1}H NMR (300 MHz, $CDCl_3$) spectra for compound **10f.**

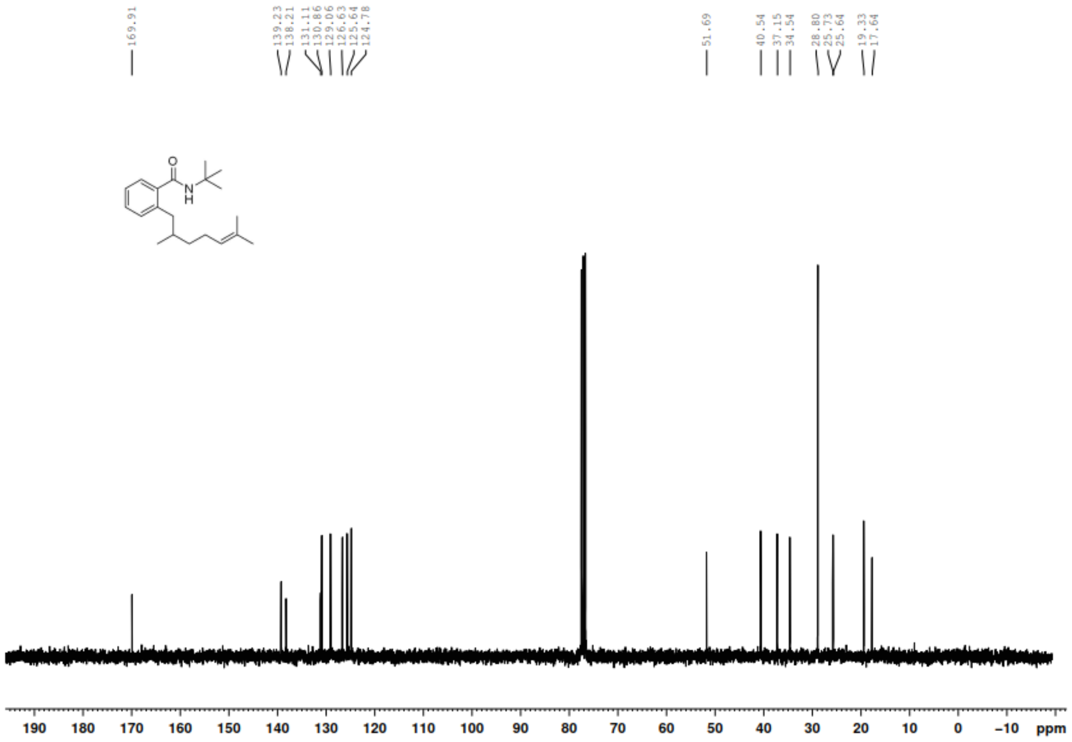

Supplementary Figure 198. ^{13}C NMR (75 MHz, $CDCl_3$) spectra for compound **10f.**

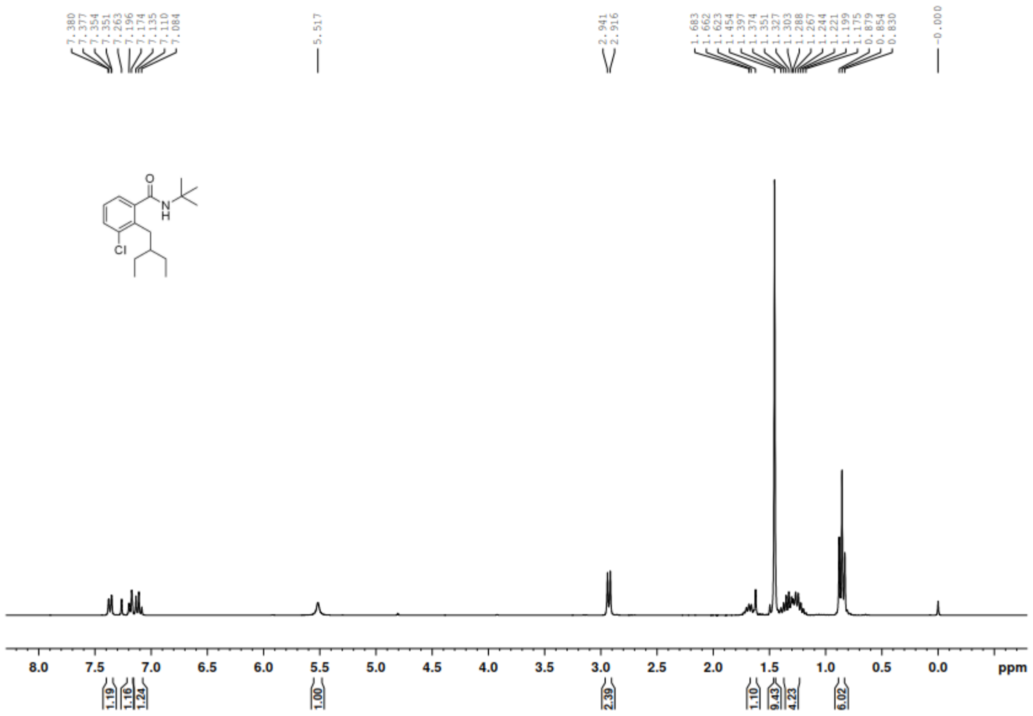

Supplementary Figure 199. ^{1}H NMR (300 MHz, $CDCl_3$) spectra for compound **10g.**

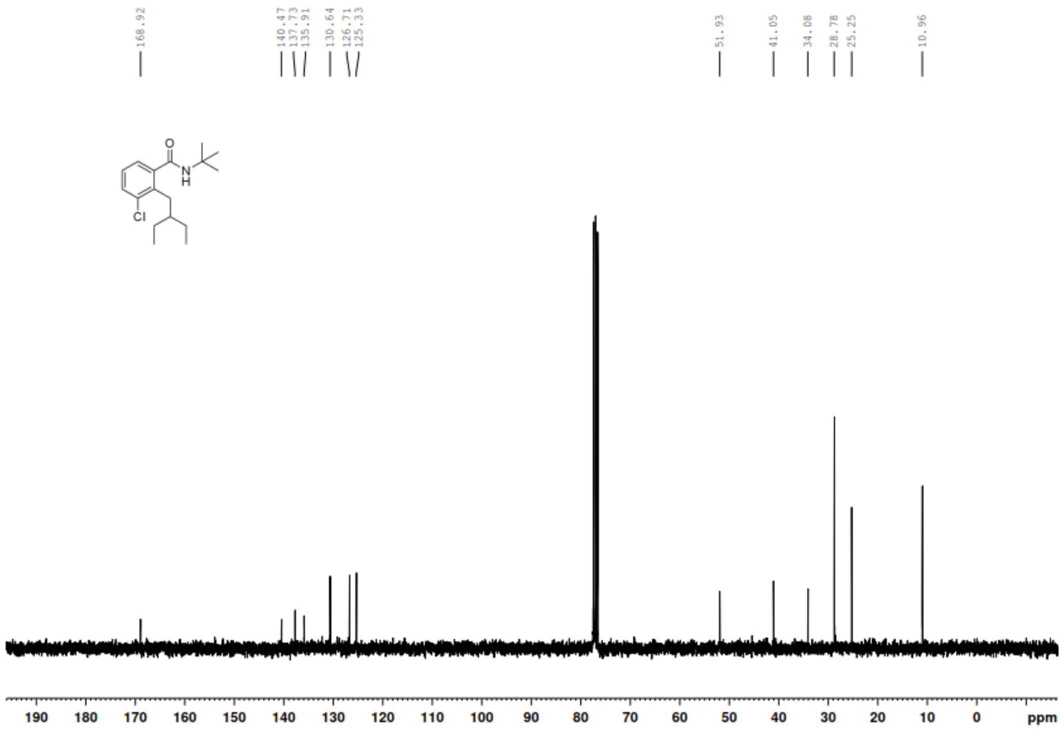

Supplementary Figure 200. ^{13}C NMR (75 MHz, $CDCl_3$) spectra for compound **10g.**

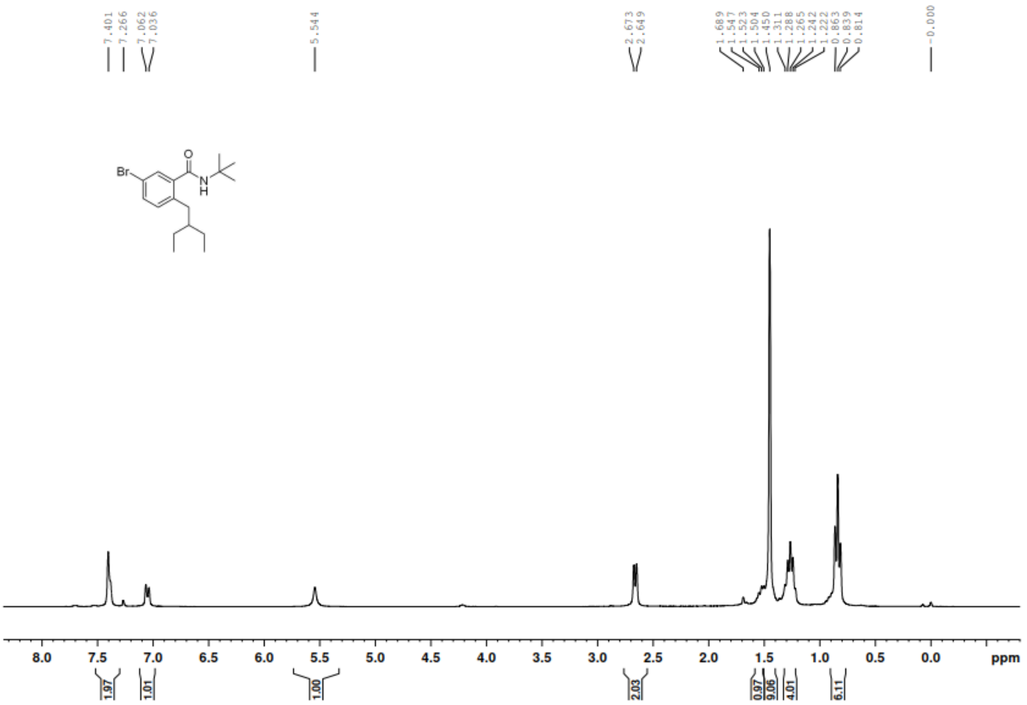

Supplementary Figure 201. ^{1}H NMR (300 MHz, $CDCl_3$) spectra for compound **10h.**

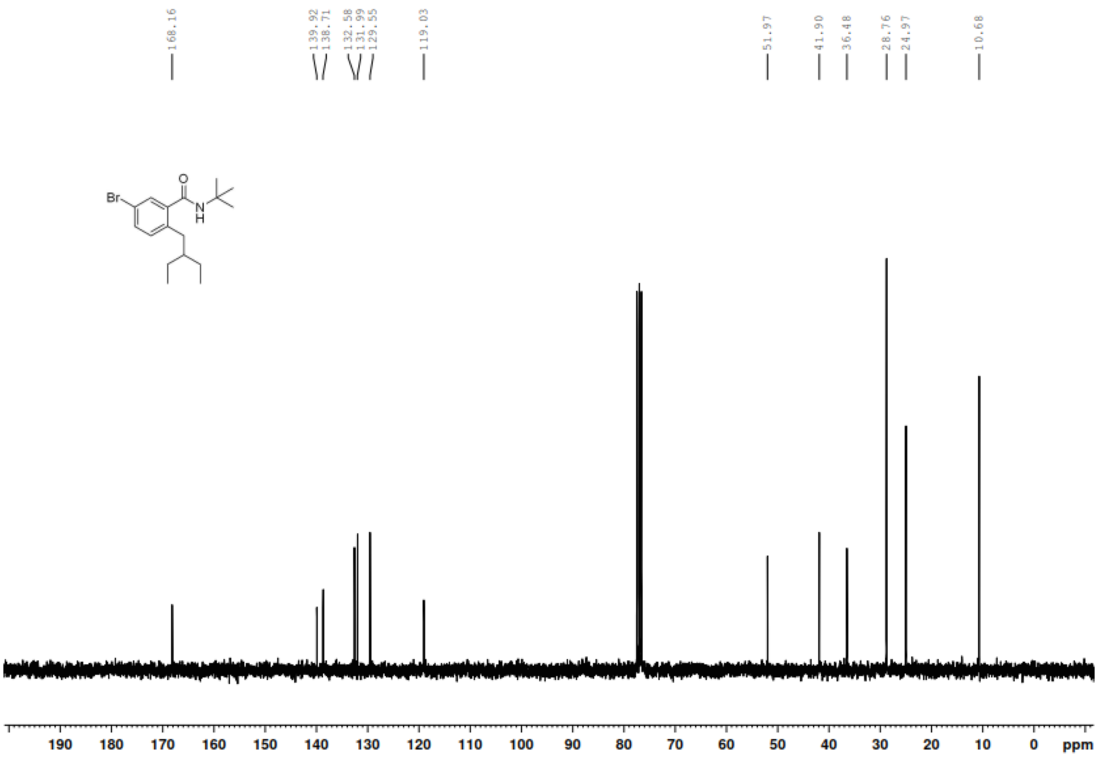

Supplementary Figure 202. ^{13}C NMR (75 MHz, $CDCl_3$) spectra for compound **10h.**

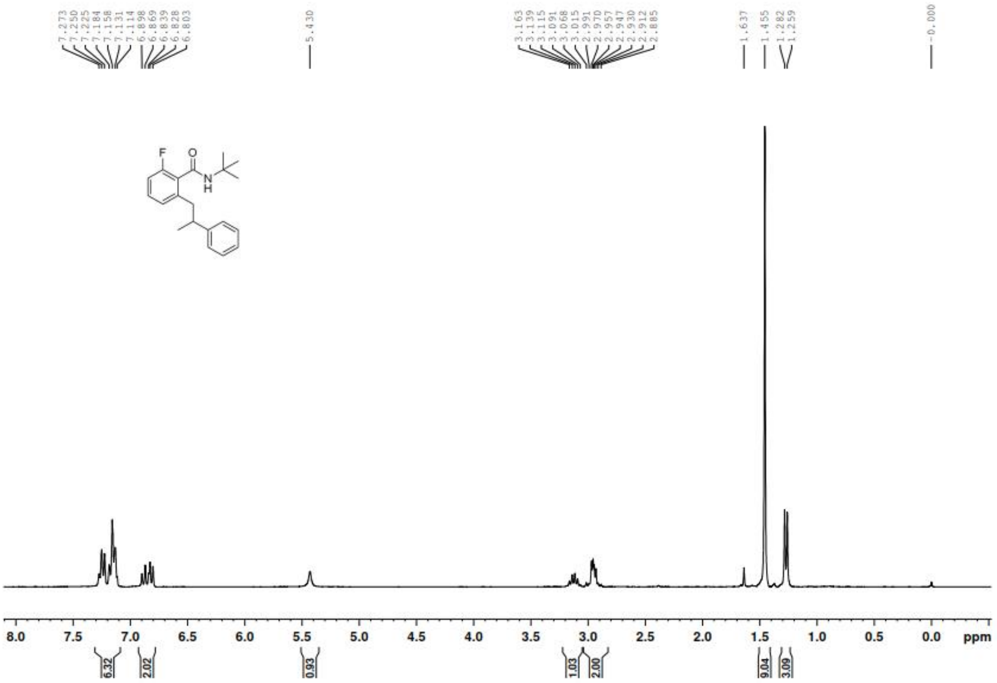

Supplementary Figure 203. ^{1}H NMR (300 MHz, $CDCl_3$) spectra for compound **10i.**

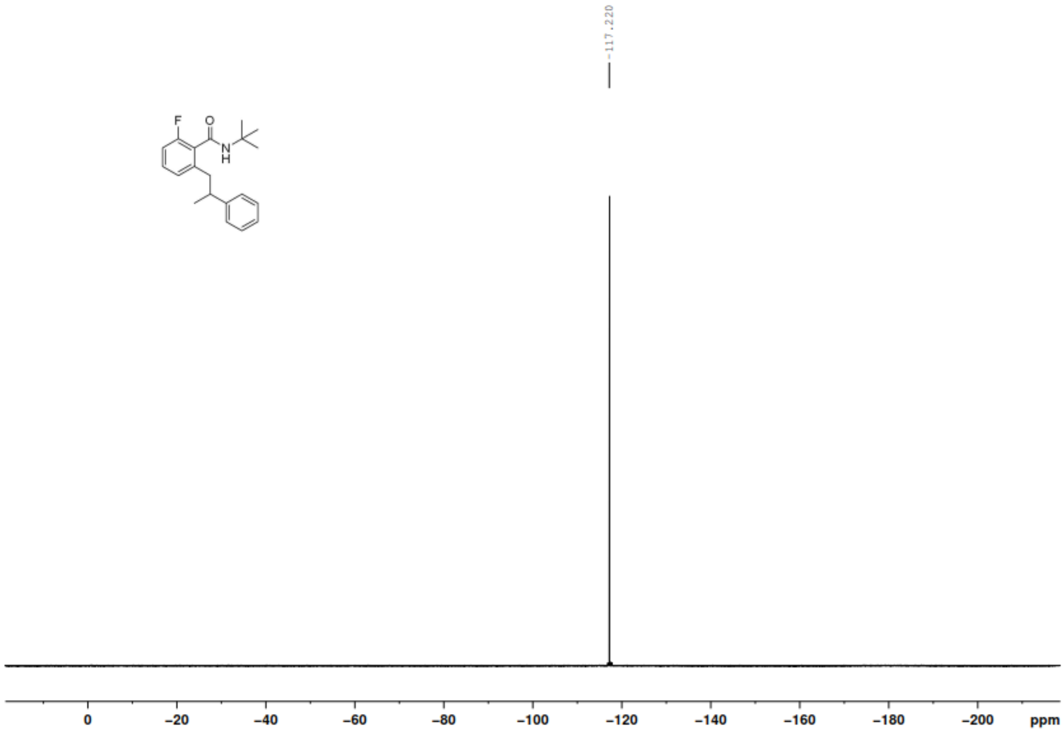

Supplementary Figure 204. ^{19}F NMR (282 MHz, $CDCl_3$) spectra for compound **10i.**

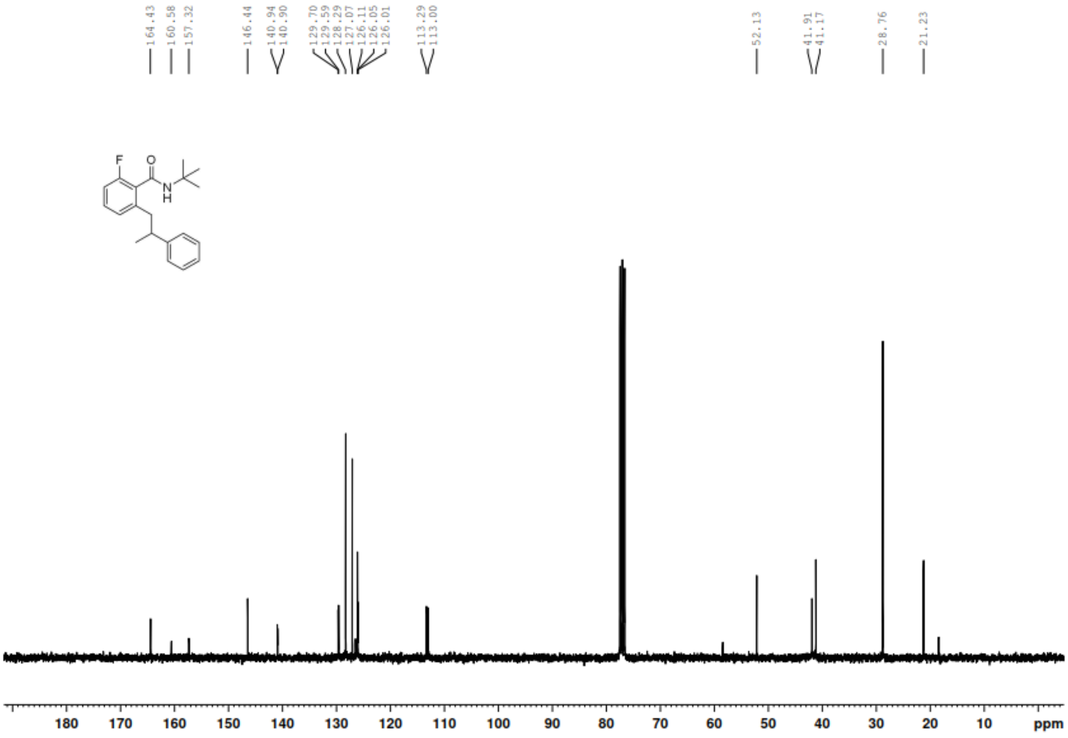

Supplementary Figure 205. ^{13}C NMR (75 MHz, $CDCl_3$) spectra for compound **10i.**

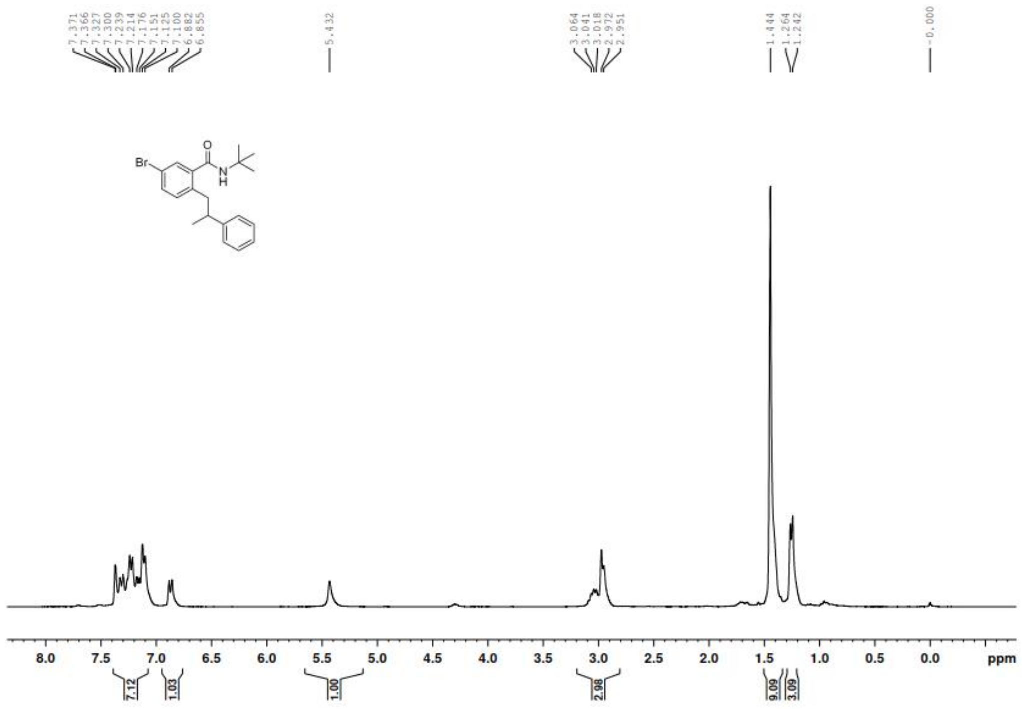

Supplementary Figure 206. ^{1}H NMR (300 MHz, $CDCl_3$) spectra for compound **10j.**

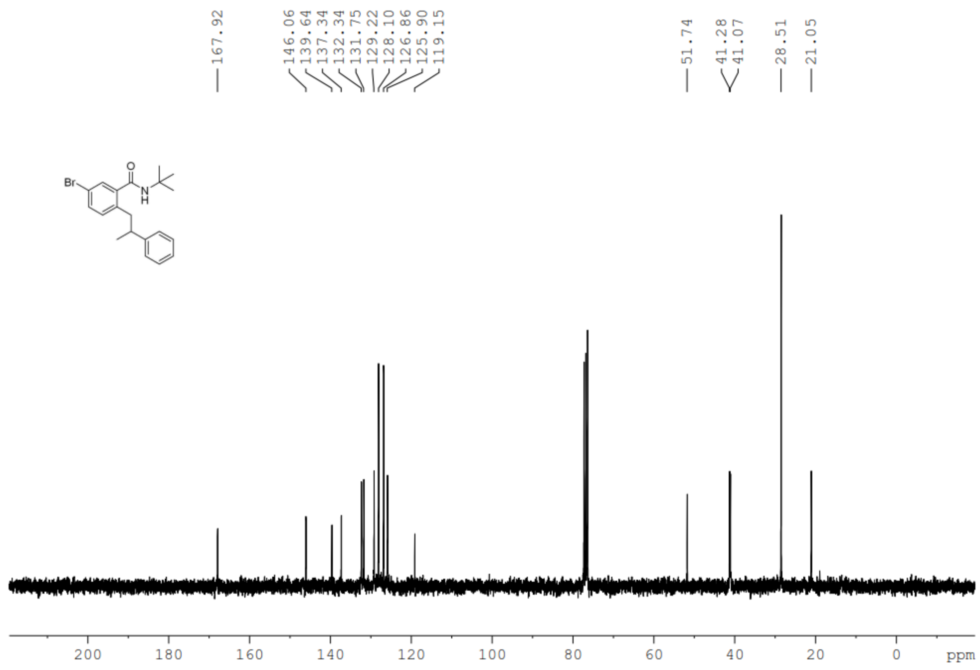

Supplementary Figure 207. ^{13}C NMR (75 MHz, $CDCl_3$) spectra for compound **10j.**

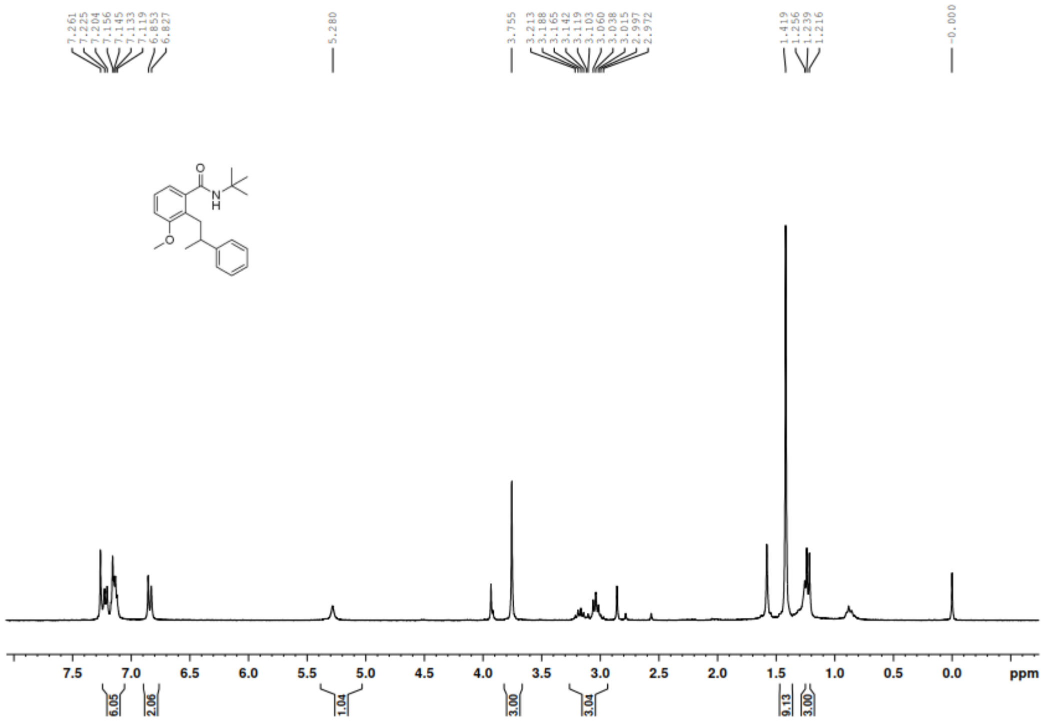

Supplementary Figure 208. ^{1}H NMR (300 MHz, $CDCl_3$) spectra for compound **10k.**

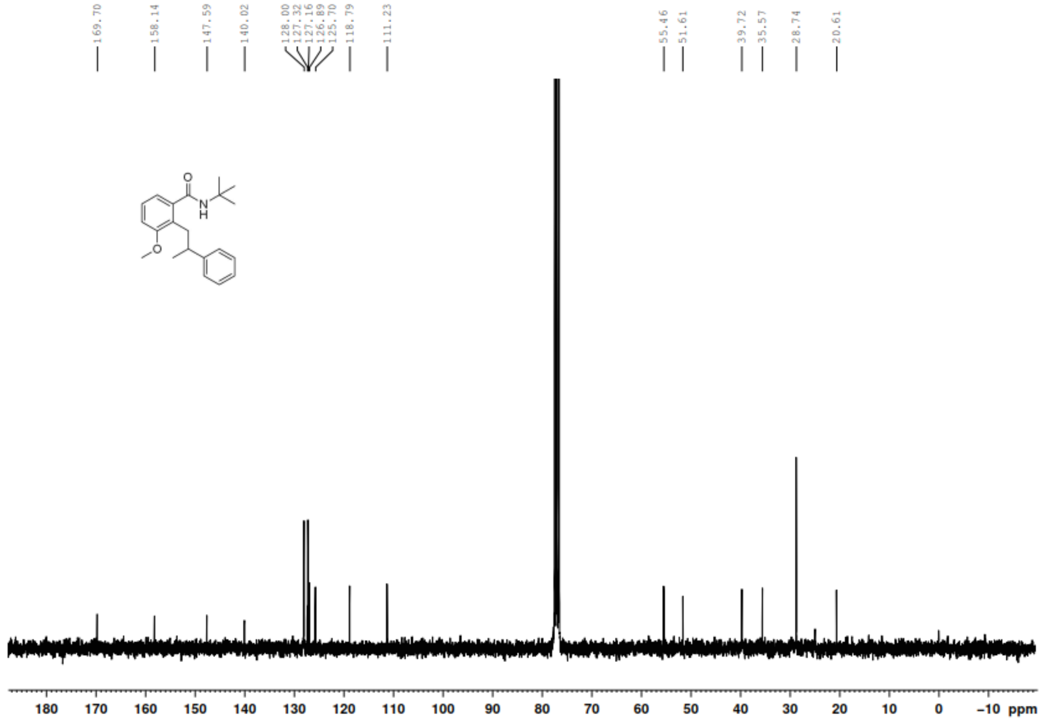

Supplementary Figure 209. ^{13}C NMR (75 MHz, $CDCl_3$) spectra for compound **10k.**

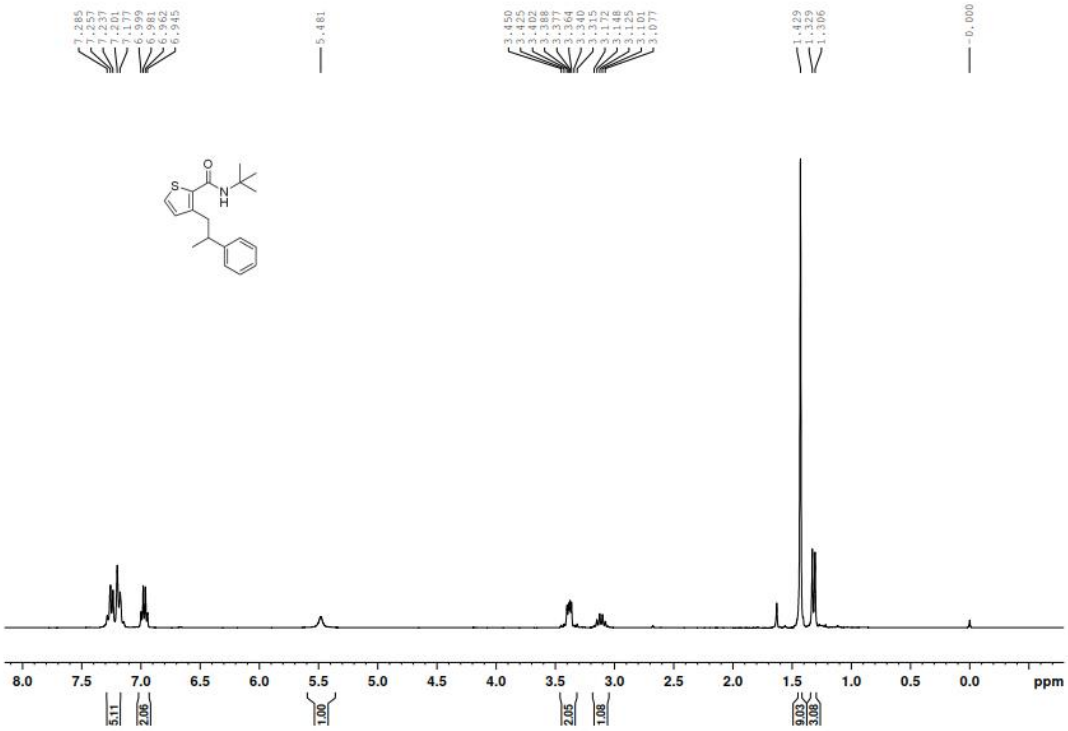

Supplementary Figure 210. ^{1}H NMR (300 MHz, $CDCl_3$) spectra for compound **10l.**

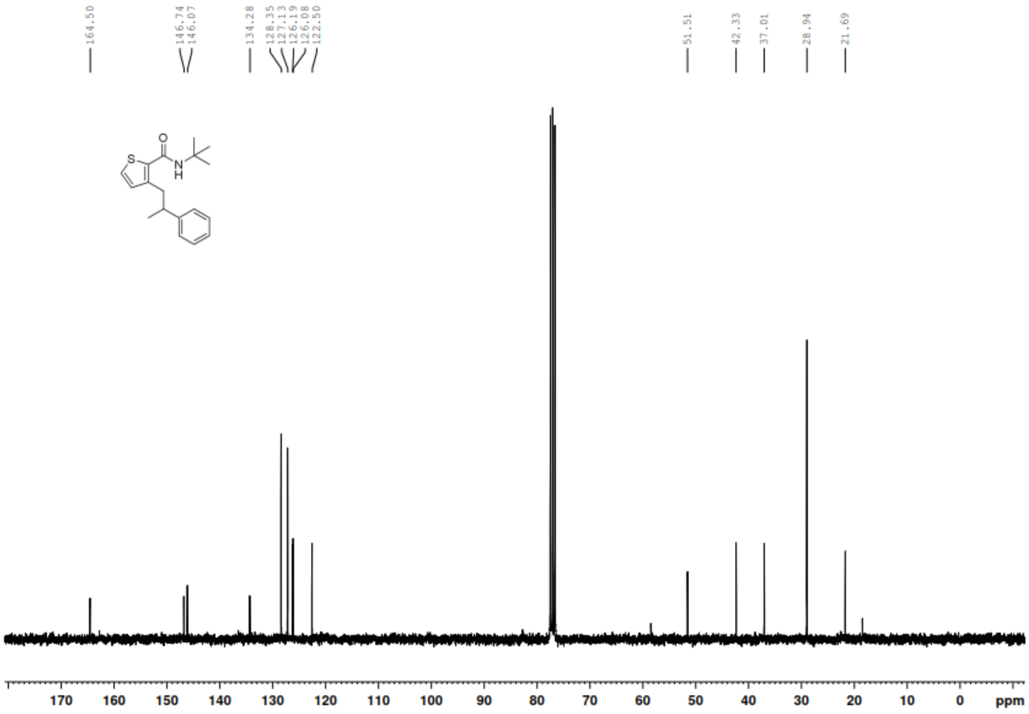

Supplementary Figure 211. ^{13}C NMR (75 MHz, $CDCl_3$) spectra for compound **10l.**

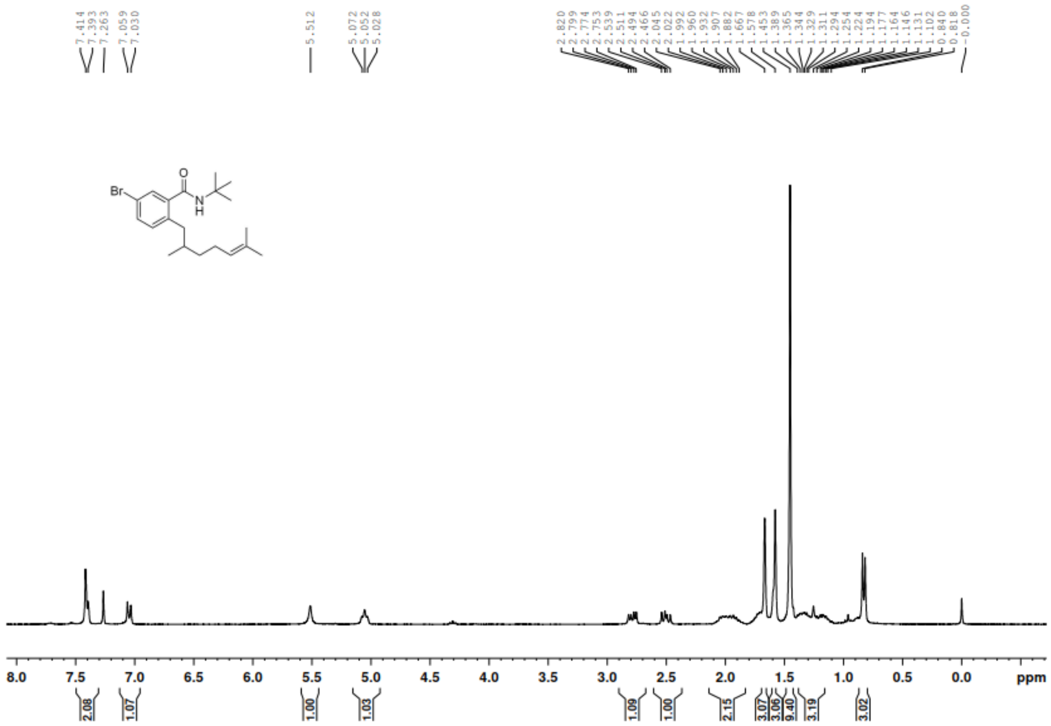

Supplementary Figure 212. ^{1}H NMR (300 MHz, $CDCl_3$) spectra for compound **10m.**

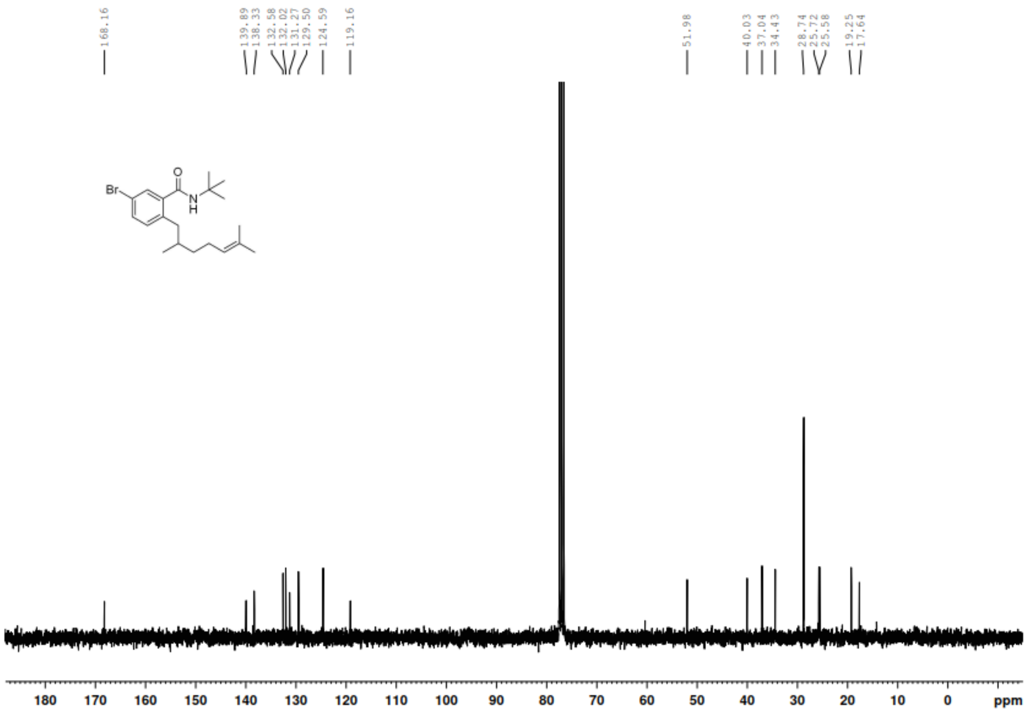

Supplementary Figure 213. ^{13}C NMR (75 MHz, $CDCl_3$) spectra for compound **10m.**

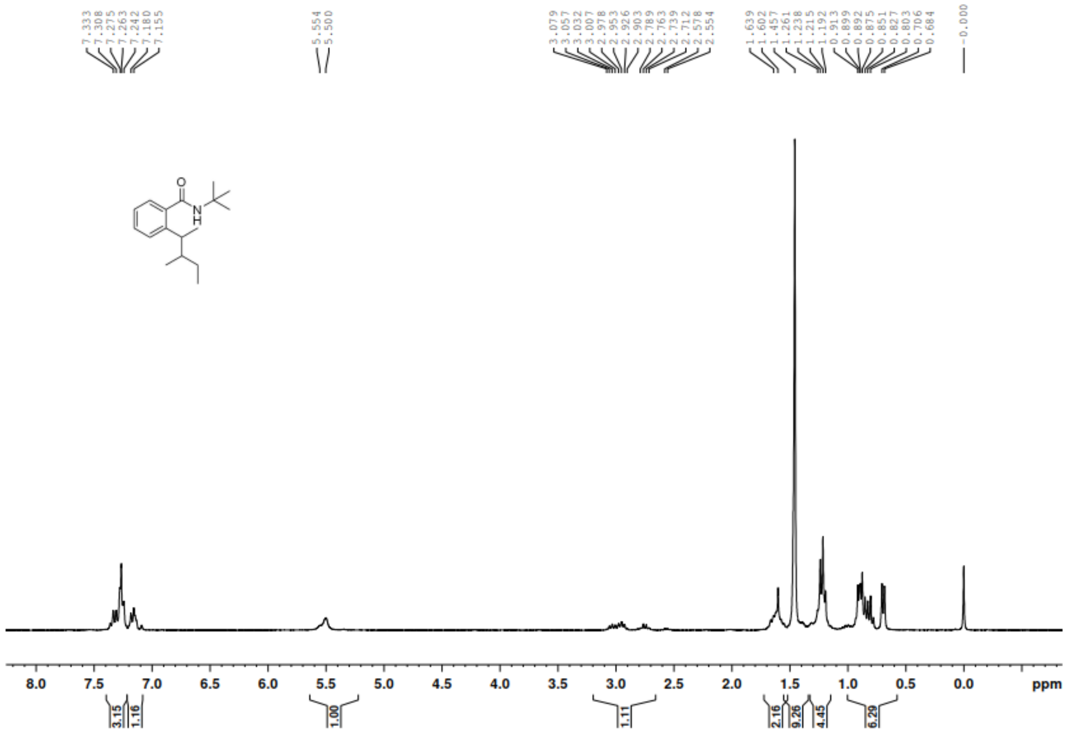

Supplementary Figure 214. ^{1}H NMR (300 MHz, $CDCl_3$) spectra for compound **10n.**

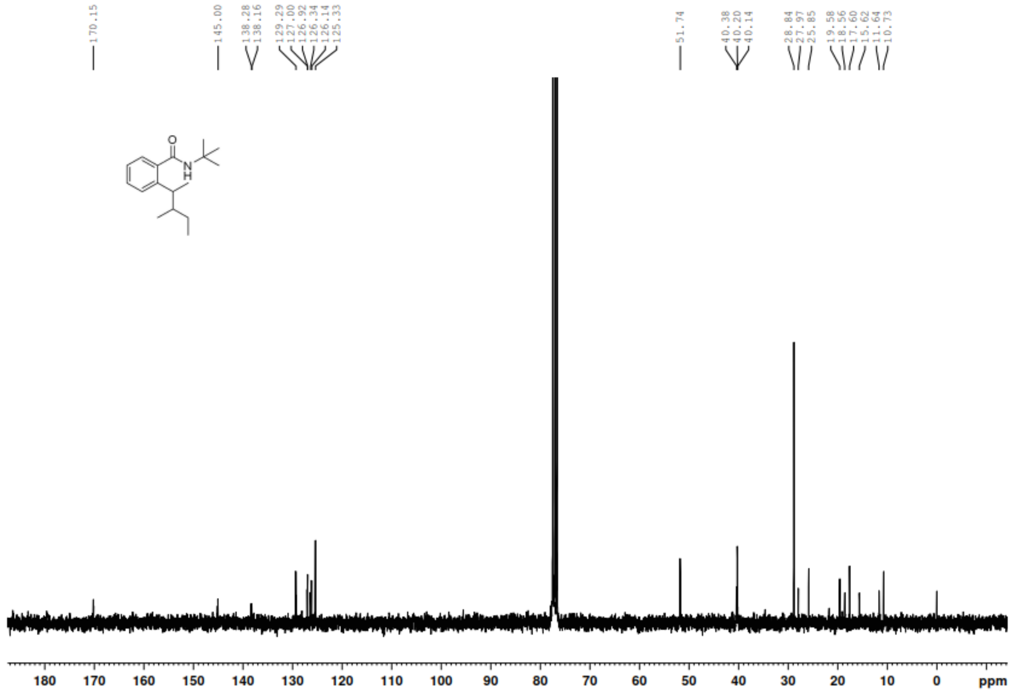

Supplementary Figure 215. ^{13}C NMR (75 MHz, $CDCl_3$) spectra for compound **10n.**

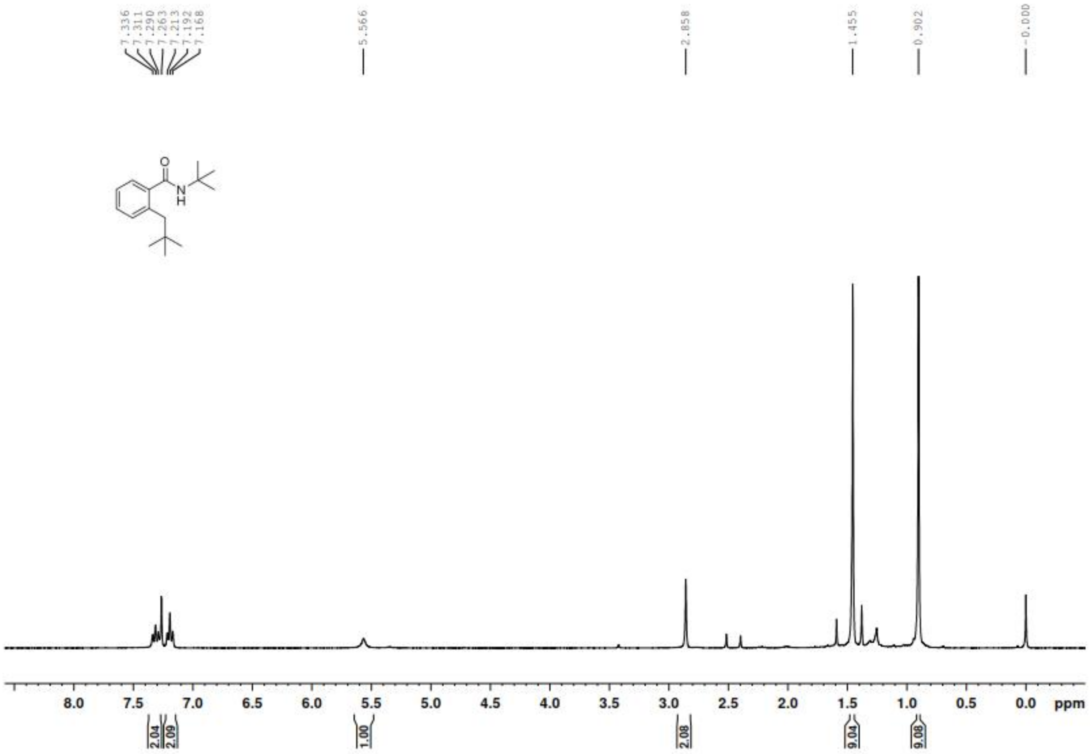

Supplementary Figure 216. ^{1}H NMR (300 MHz, $CDCl_3$) spectra for compound **11a.**

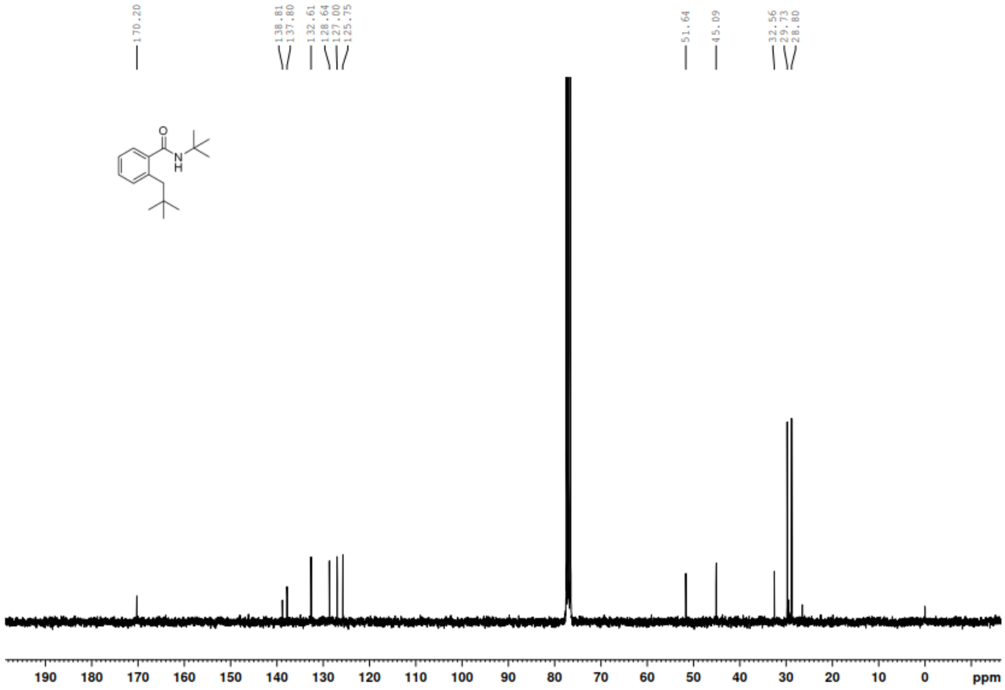

Supplementary Figure 217. ^{13}C NMR (75 MHz, $CDCl_3$) spectra for compound **11a.**

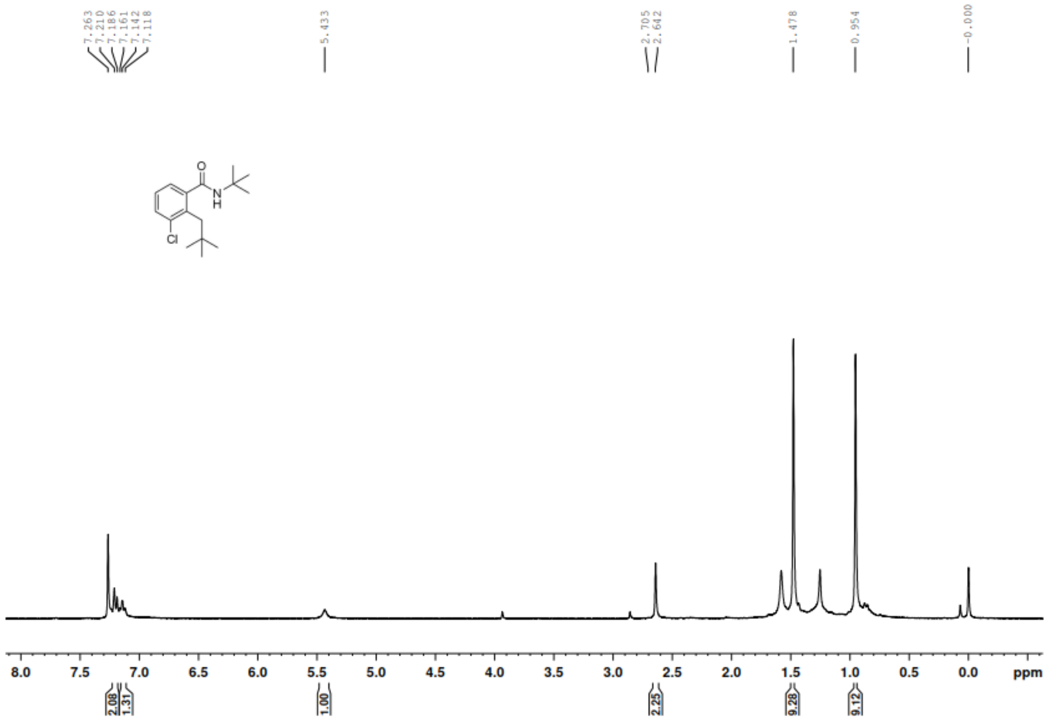

Supplementary Figure 218. ^{1}H NMR (300 MHz, $CDCl_3$) spectra for compound **11b.**

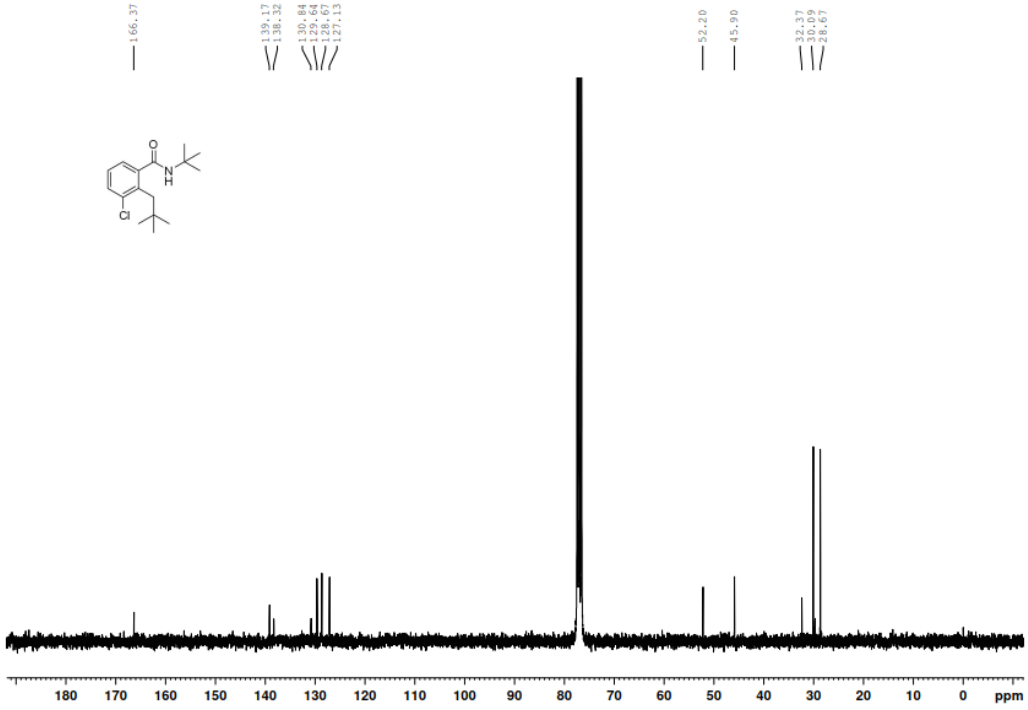

Supplementary Figure 219. ^{13}C NMR (75 MHz, $CDCl_3$) spectra for compound **11b.**

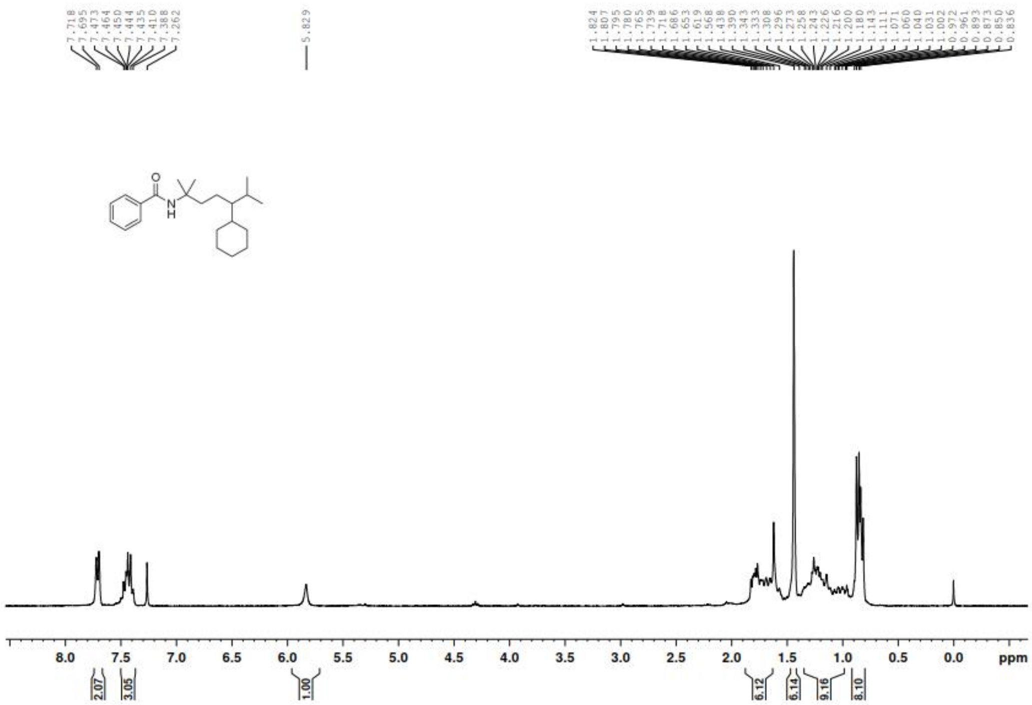

Supplementary Figure 220. ^{1}H NMR (300 MHz, $CDCl_3$) spectra for compound **12a.**

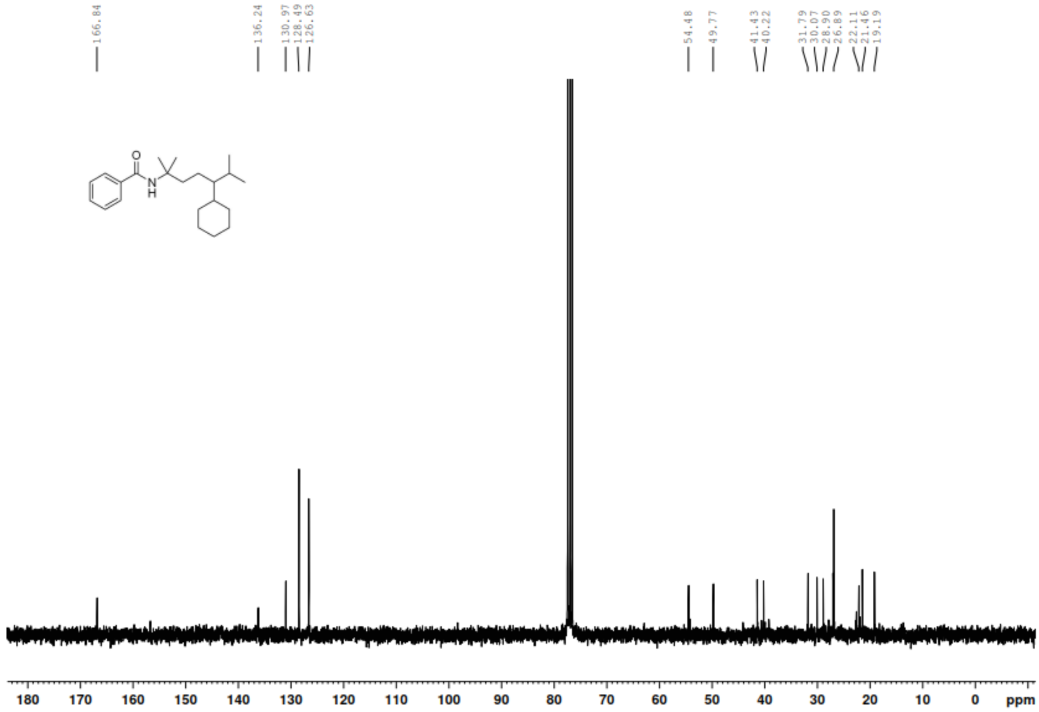

Supplementary Figure 221. ^{13}C NMR (75 MHz, $CDCl_3$) spectra for compound **12a.**

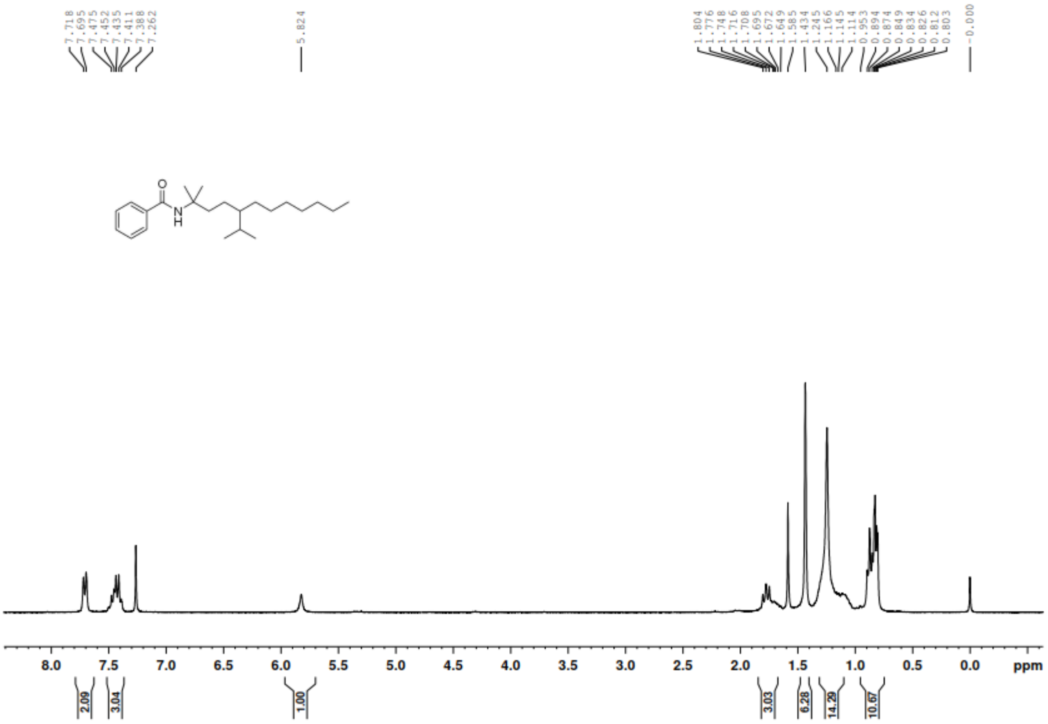

Supplementary Figure 222. ^{1}H NMR (300 MHz, $CDCl_3$) spectra for compound **12b.**

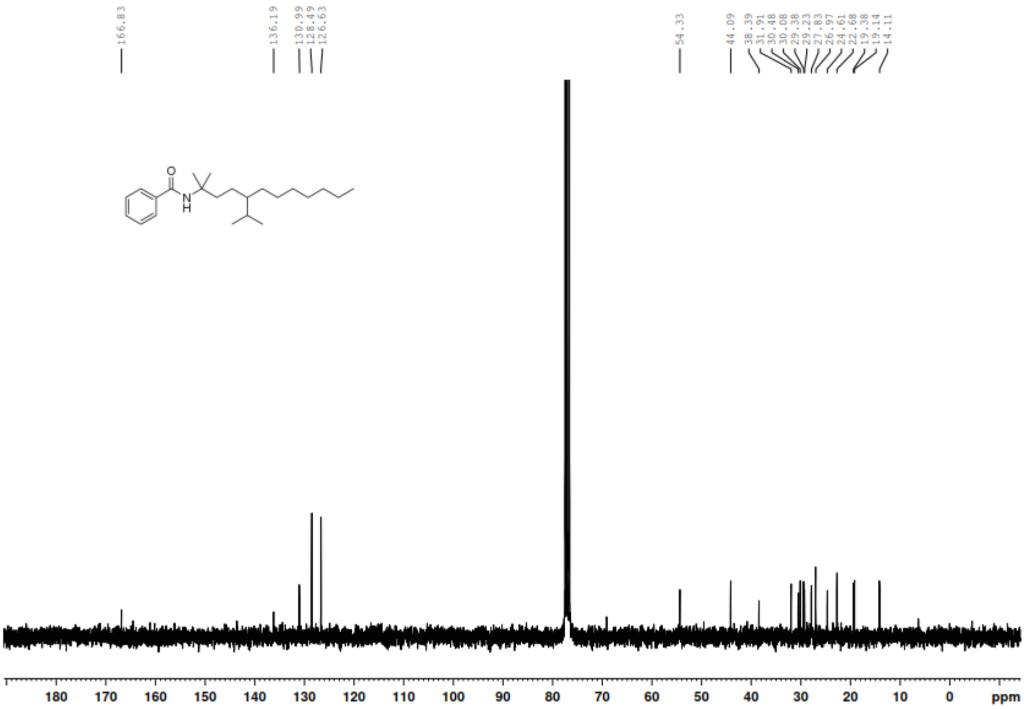

Supplementary Figure 223. ^{13}C NMR (75 MHz, $CDCl_3$) spectra for compound **12b.**

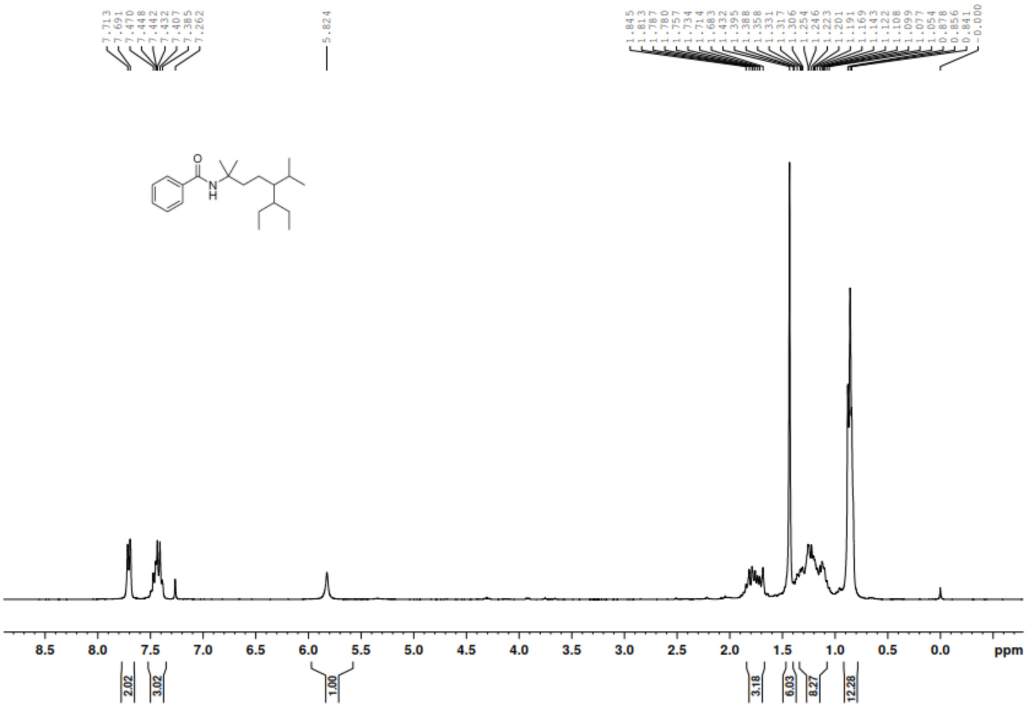

Supplementary Figure 224. ^{1}H NMR (300 MHz, $CDCl_3$) spectra for compound **12c.**

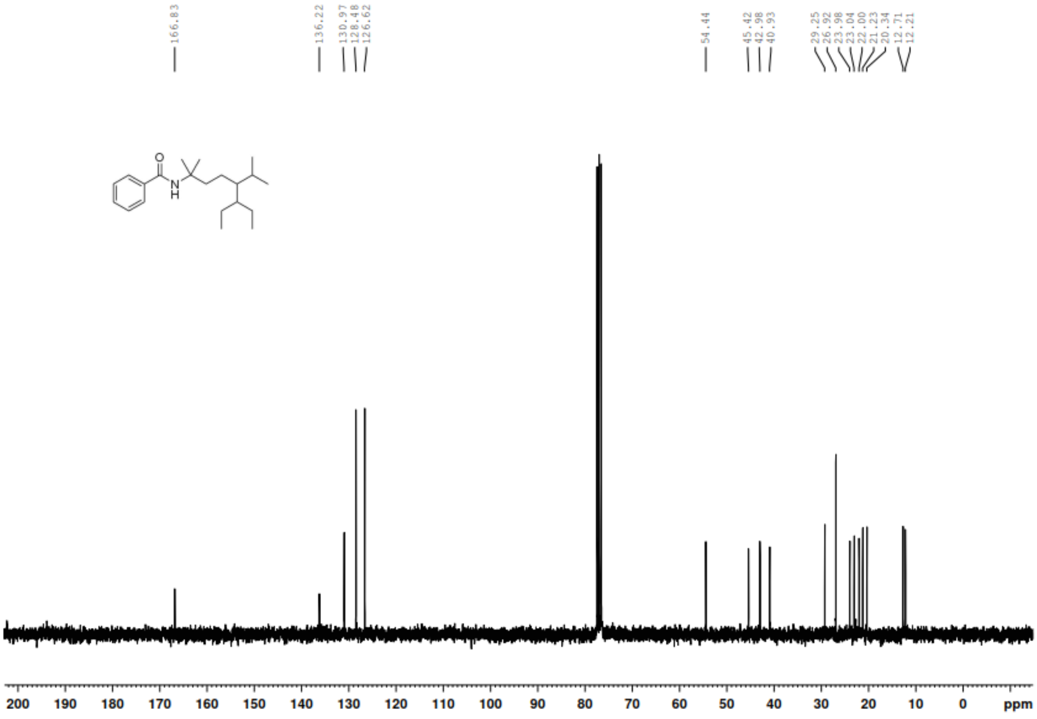

Supplementary Figure 225. ^{13}C NMR (75 MHz, $CDCl_3$) spectra for compound **12c.**

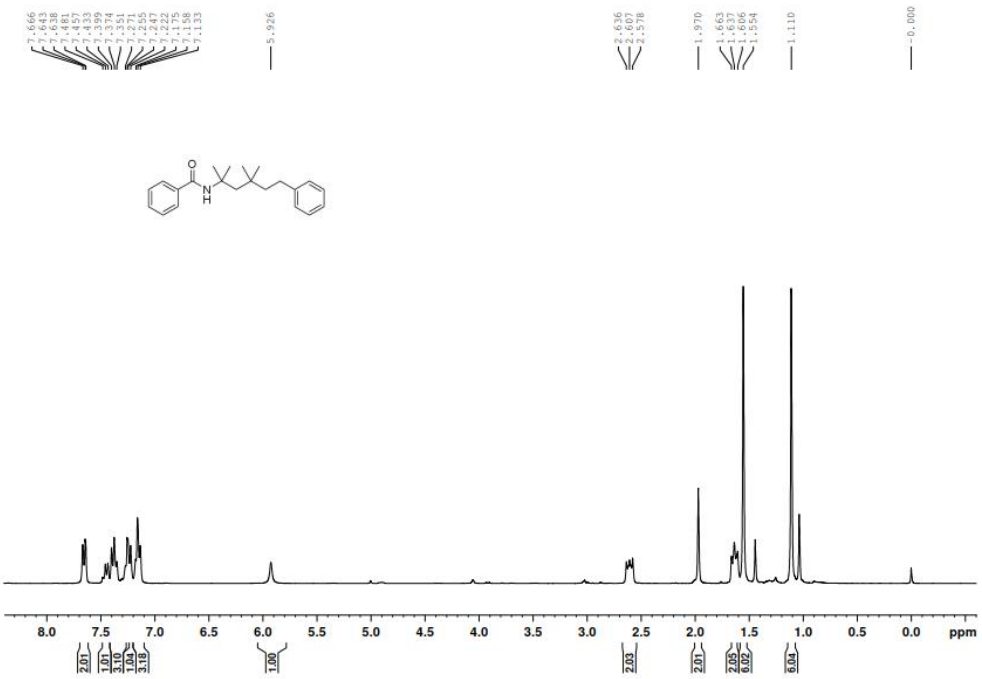

Supplementary Figure 226. ^{1}H NMR (300 MHz, $CDCl_3$) spectra for compound **12d.**

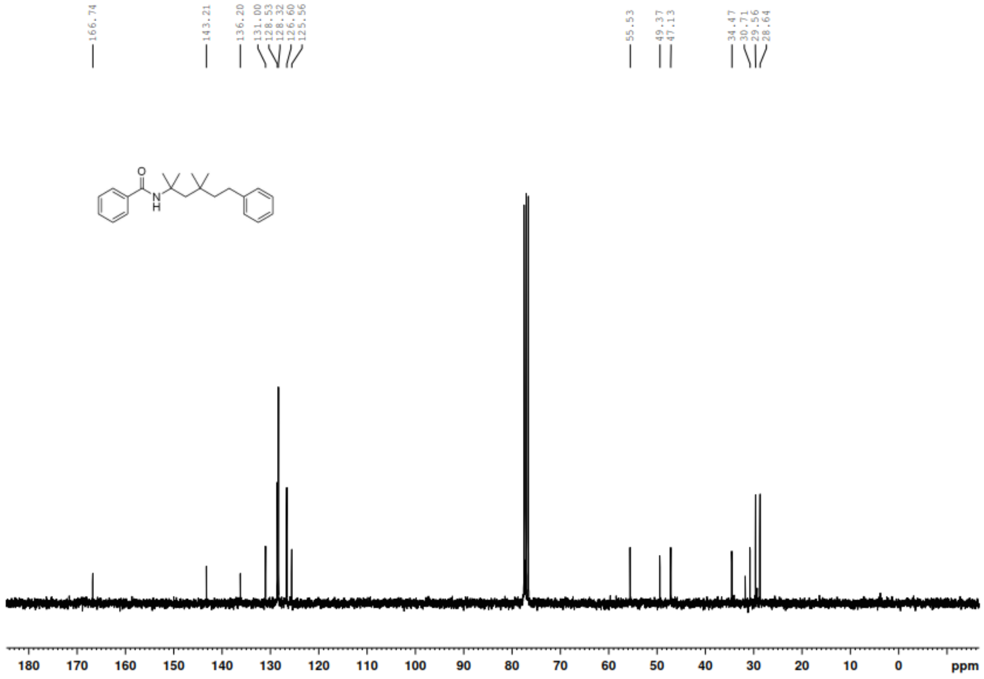

Supplementary Figure 227. ^{13}C NMR (75 MHz, $CDCl_3$) spectra for compound **12d.**

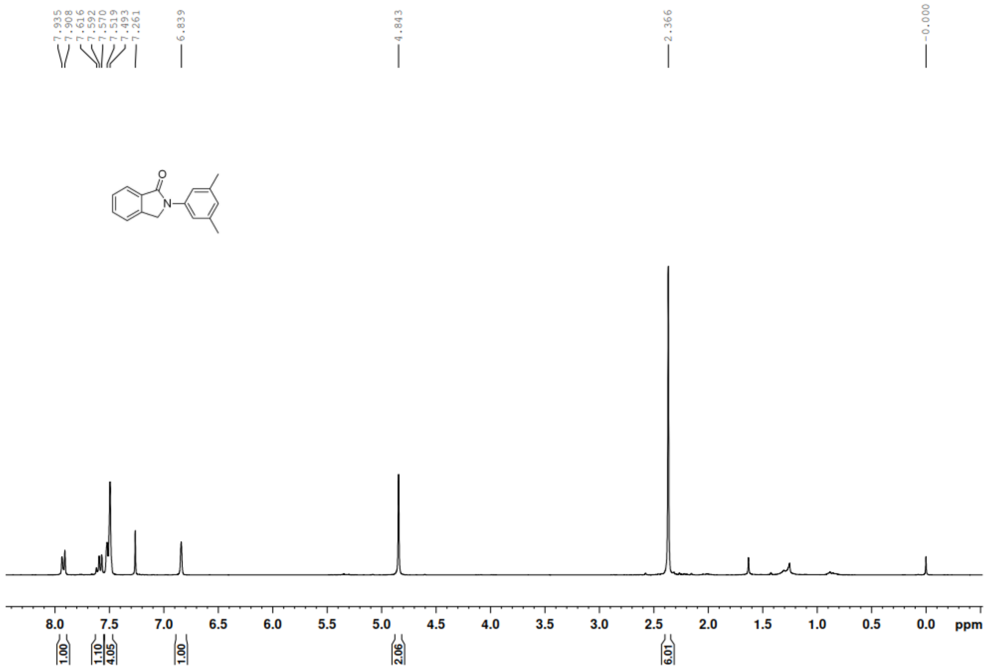

Supplementary Figure 228. ^{1}H NMR (300 MHz, $CDCl_3$) spectra for compound **3f'.**

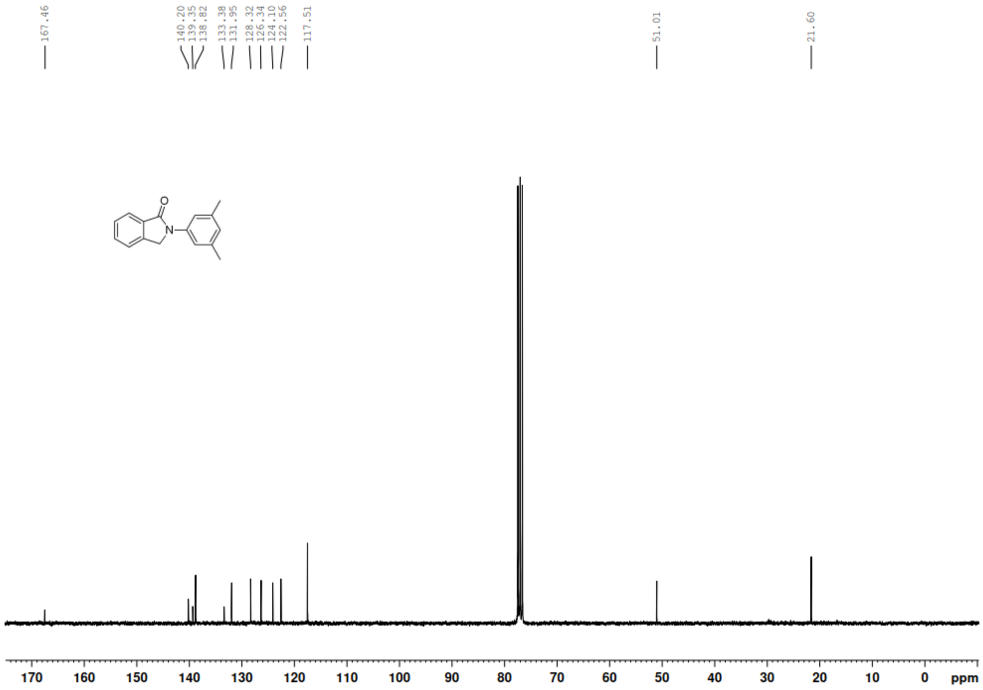

Supplementary Figure 229. ^{13}C NMR (75 MHz, $CDCl_3$) spectra for compound **3f'.**

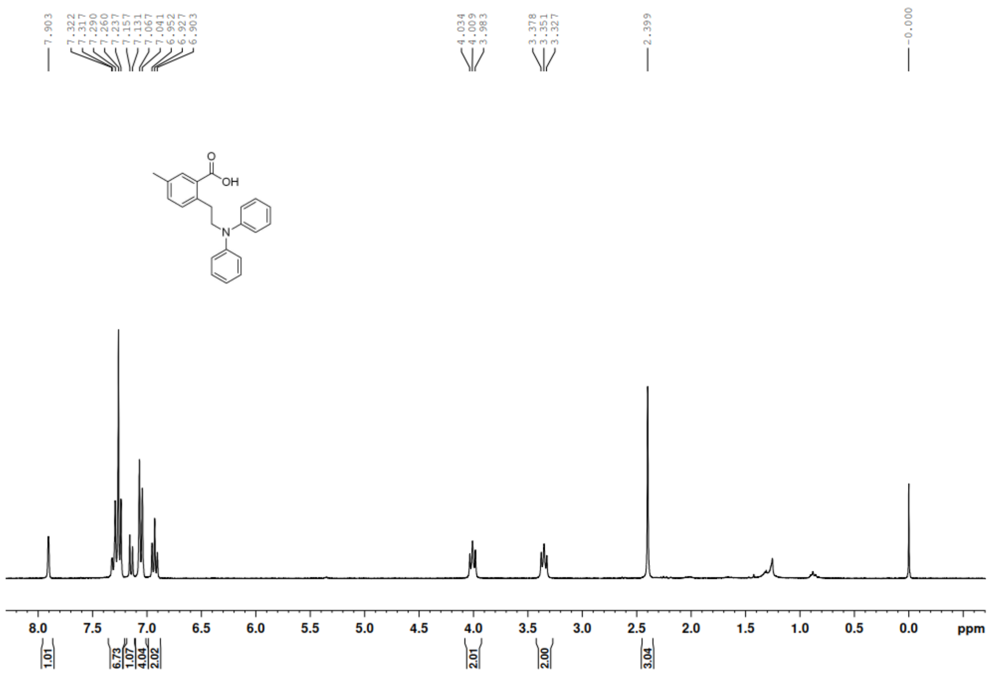

Supplementary Figure 230. ^{1}H NMR (300 MHz, $CDCl_3$) spectra for compound **4s'.**

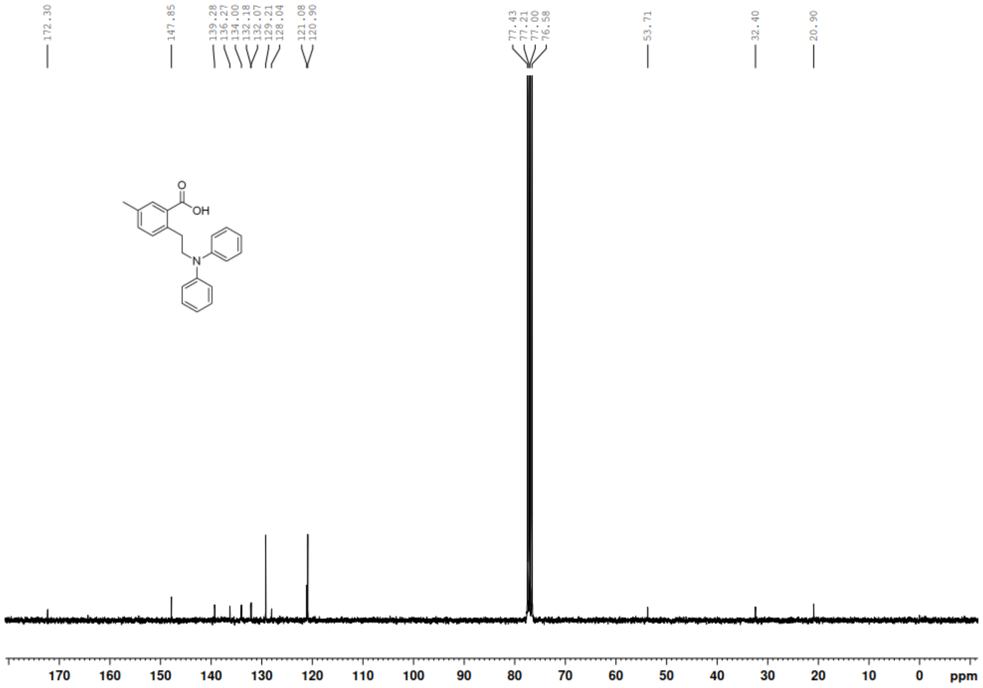

Supplementary Figure 231. ^{13}C NMR (75 MHz, $CDCl_3$) spectra for compound **4s'.**

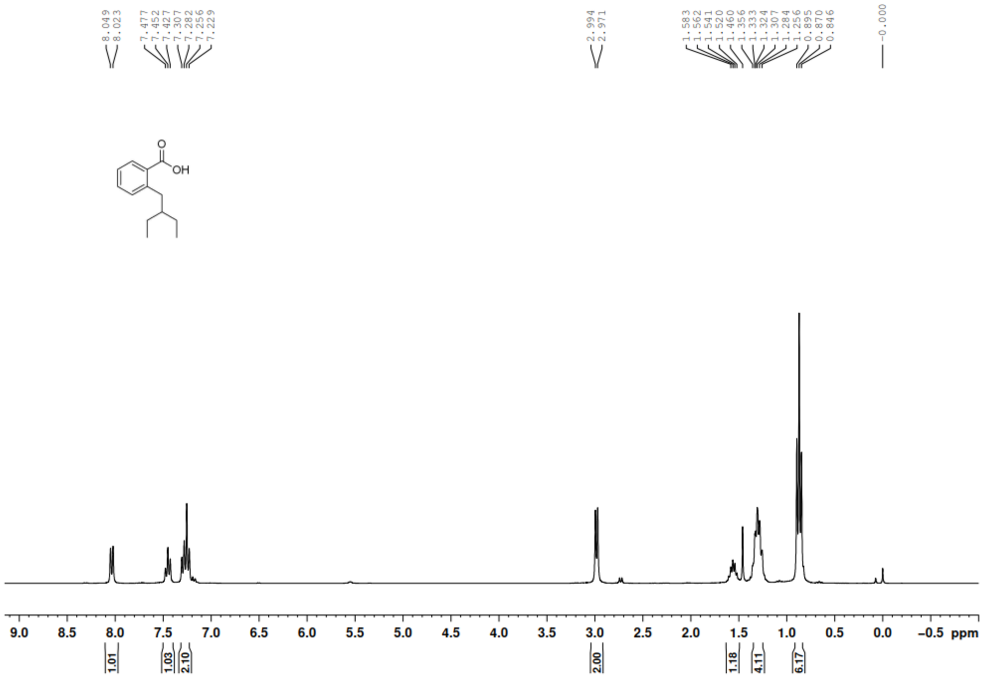

Supplementary Figure 232. ^{1}H NMR (300 MHz, $CDCl_3$) spectra for compound **10b'.**

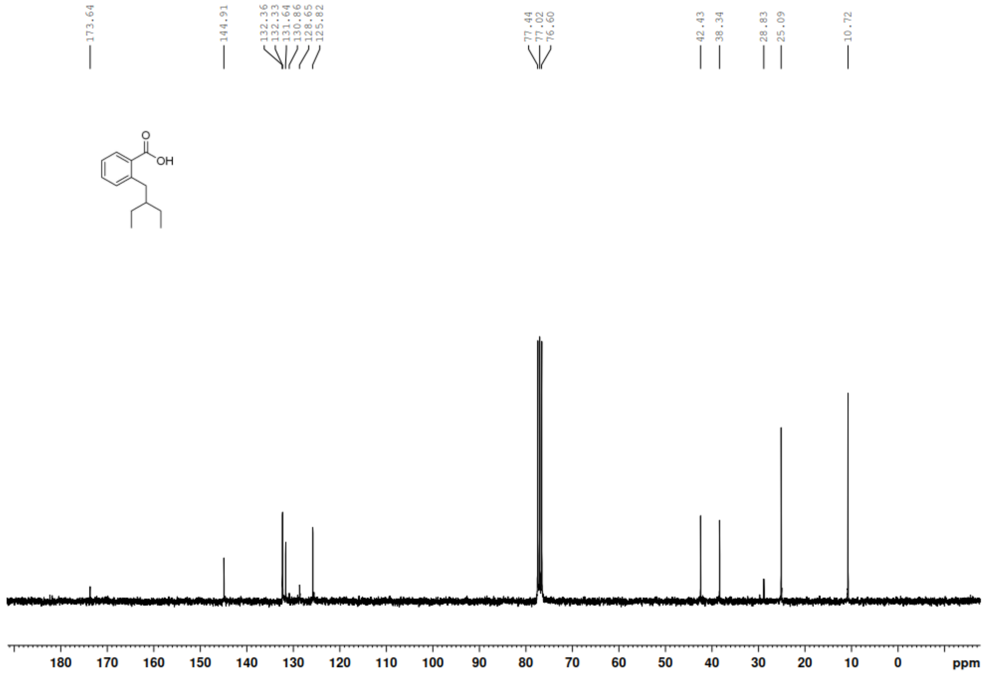

Supplementary Figure 233. ^{13}C NMR (75 MHz, $CDCl_3$) spectra for compound **10b'.**

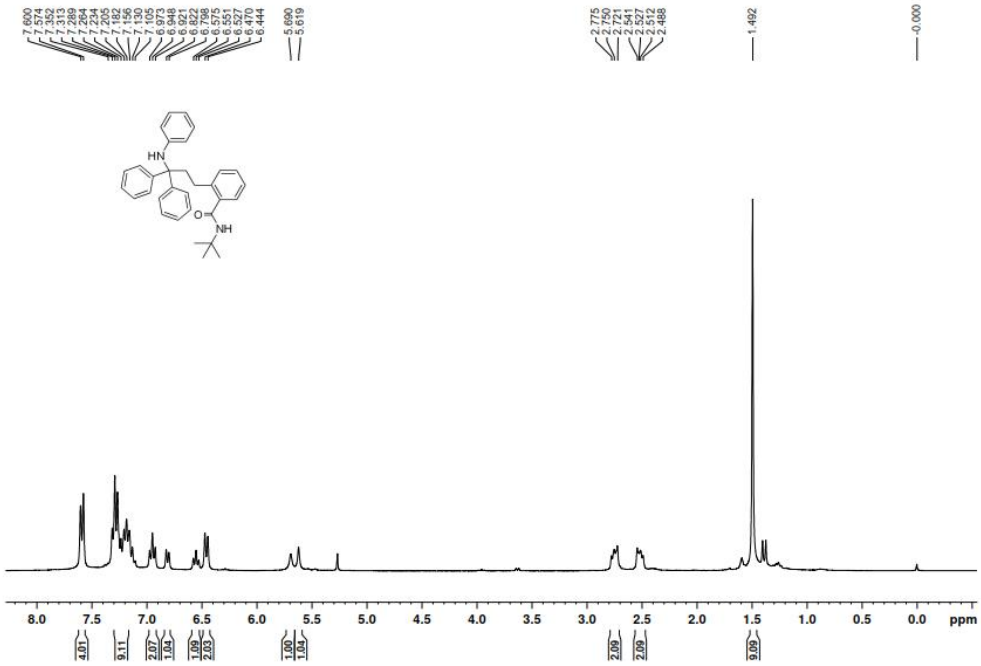

Supplementary Figure 234. ^{1}H NMR (300 MHz, $CDCl_3$) spectra for compound **13.**

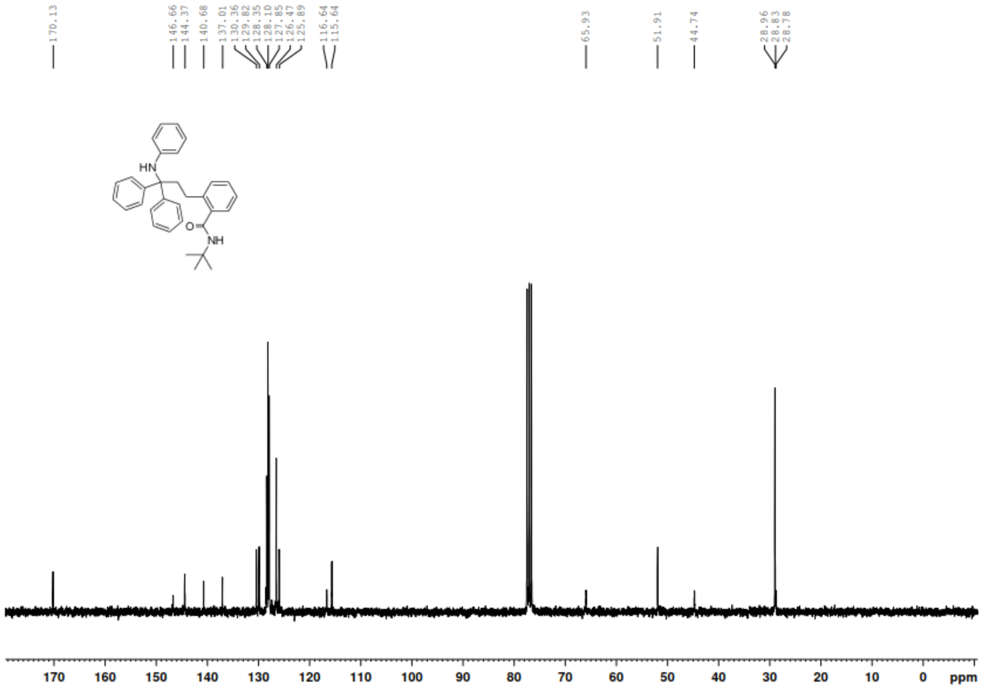

Supplementary Figure 235. ^{13}C NMR (75 MHz, $CDCl_3$) spectra for compound **13.**

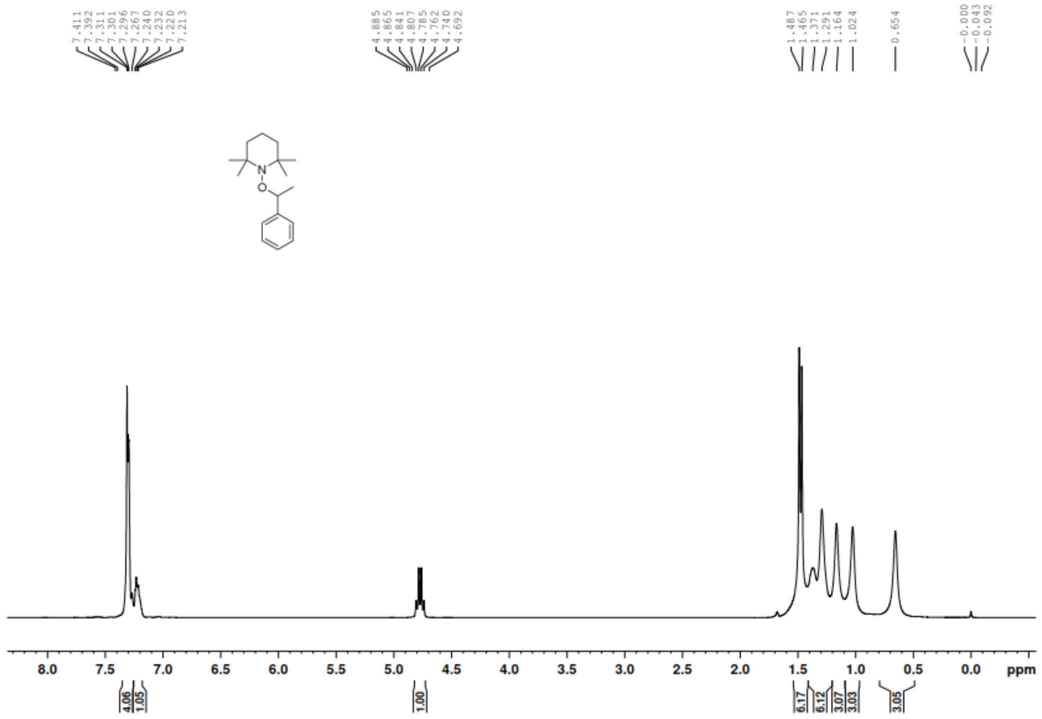

Supplementary Figure 236. ^{1}H NMR (300 MHz, $CDCl_3$) spectra for compound **16.**

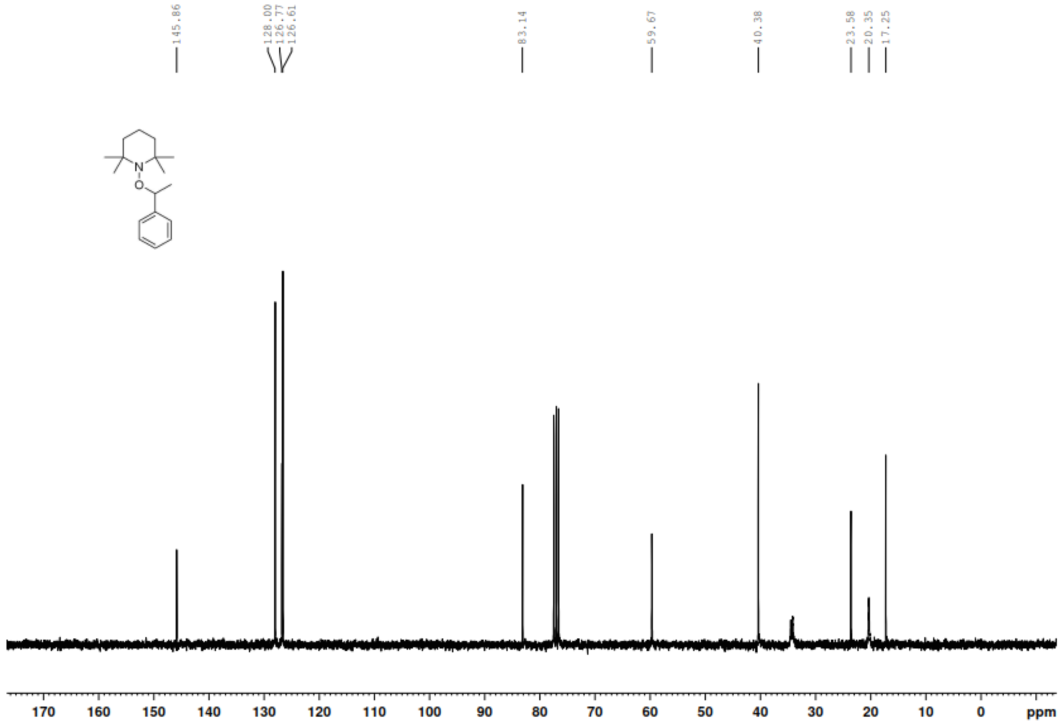

Supplementary Figure 237. ^{13}C NMR (75 MHz, $CDCl_3$) spectra for compound **16.**

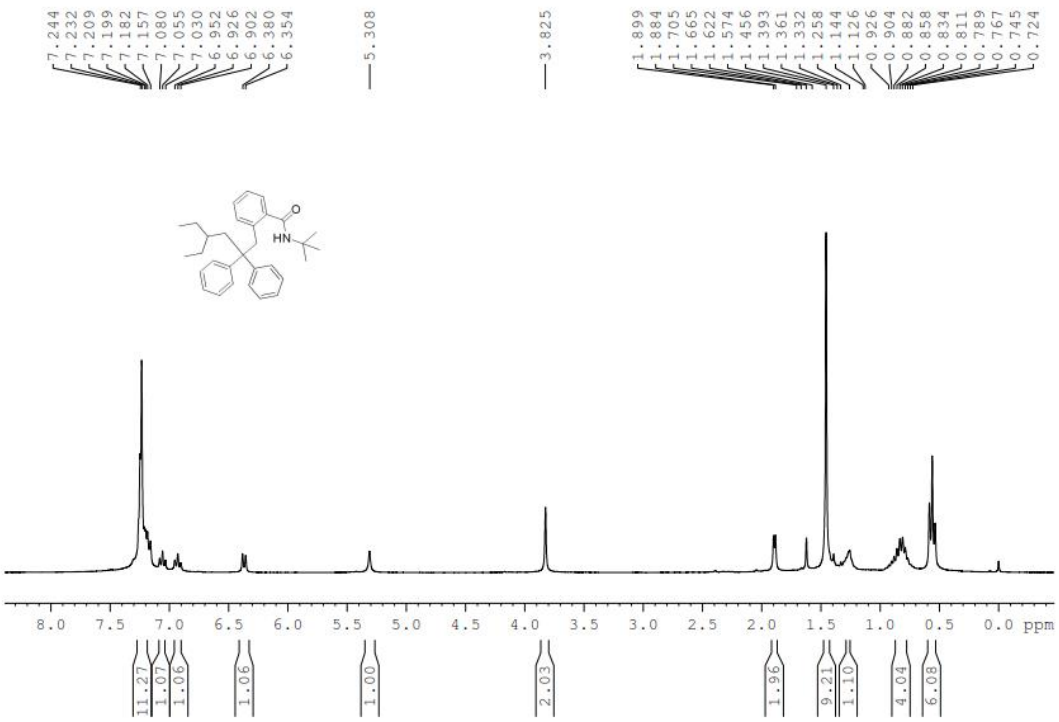

Supplementary Figure 238. ^{1}H NMR (300 MHz, $CDCl_3$) spectra for compound **17.**

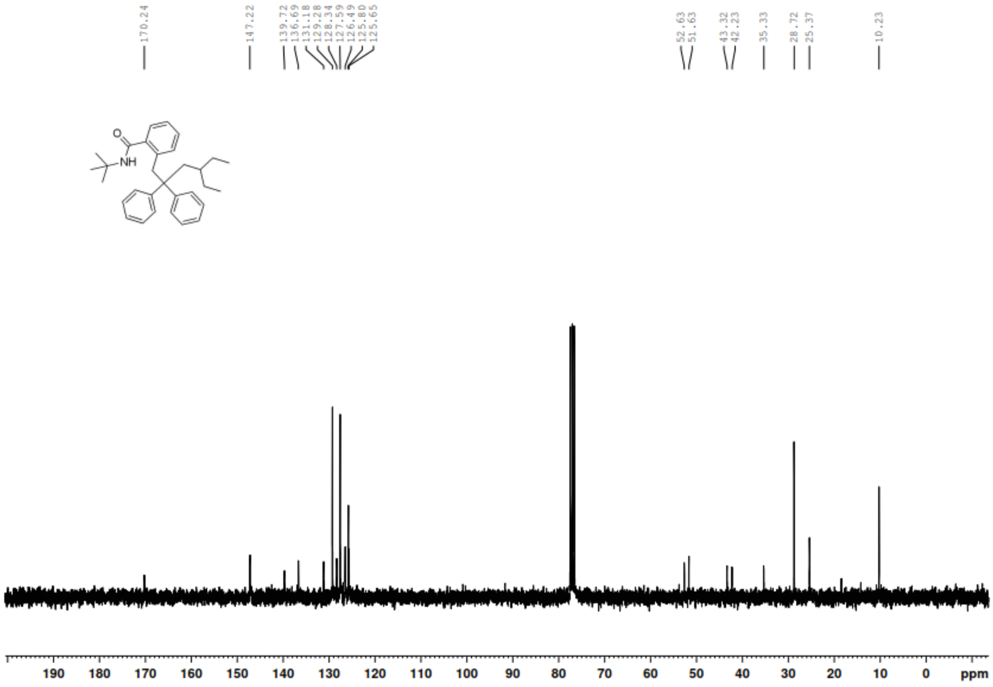

Supplementary Figure 239. ^{13}C NMR (75 MHz, $CDCl_3$) spectra for compound **17.**

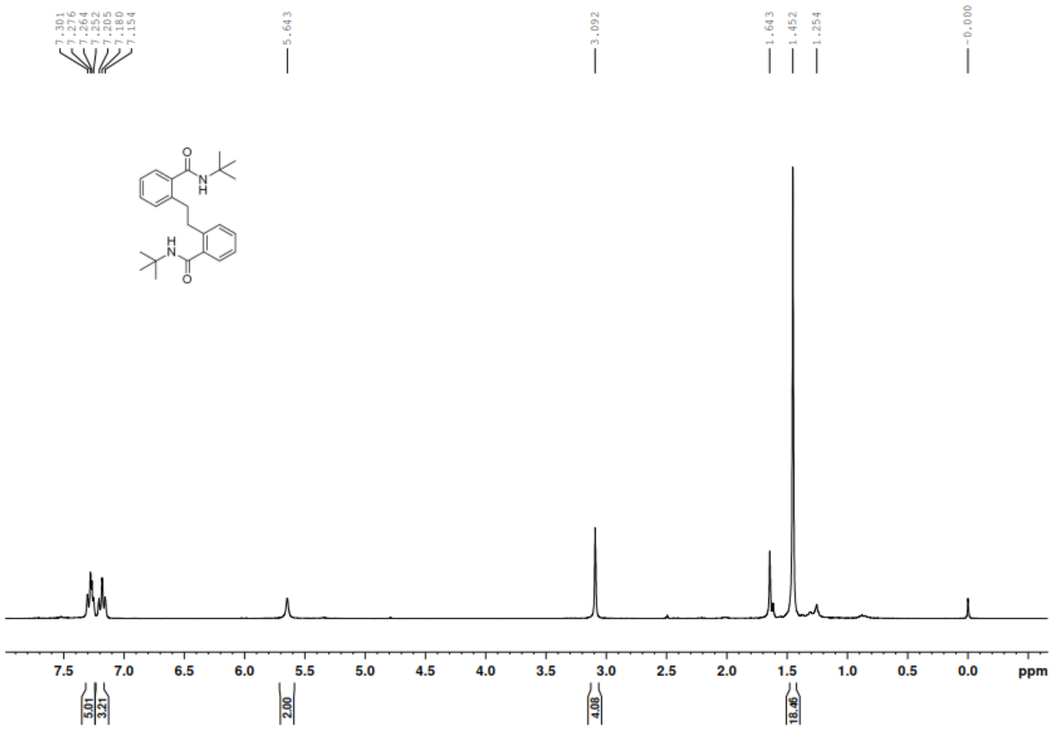

Supplementary Figure 240. ^{1}H NMR (300 MHz, $CDCl_3$) spectra for compound **14.**

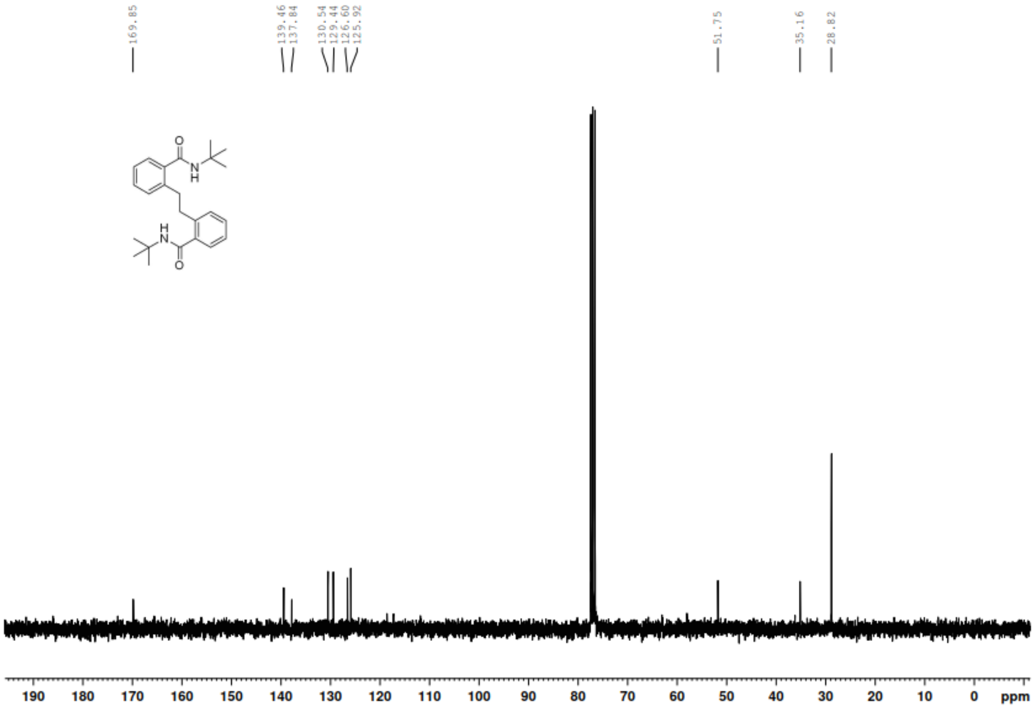

Supplementary Figure 241. ^{13}C NMR (75 MHz, $CDCl_3$) spectra for compound **14.**

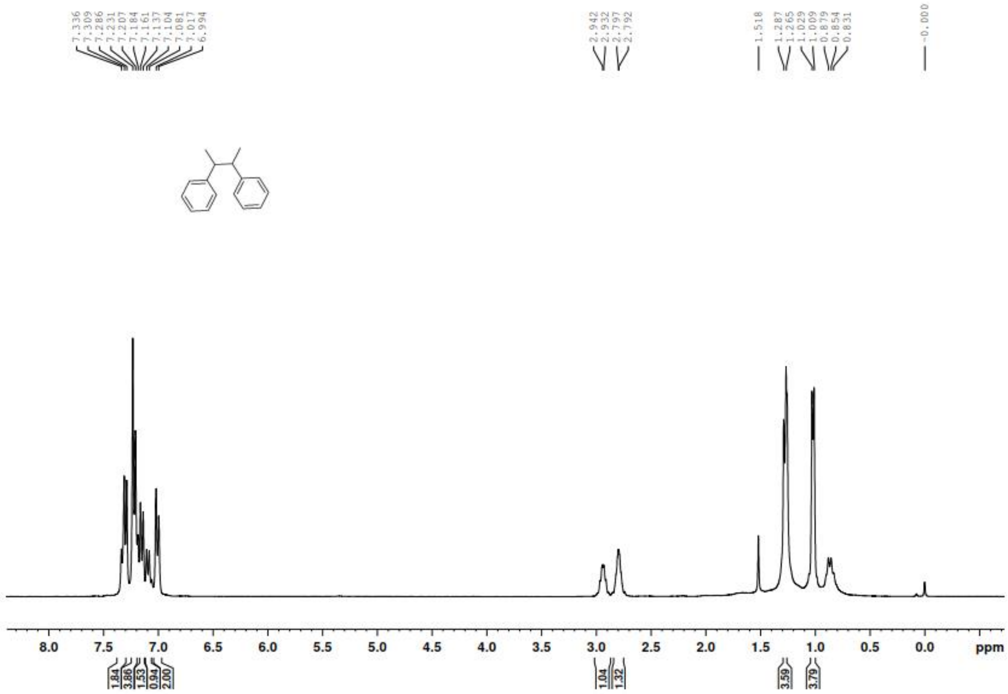

Supplementary Figure 242. ^{1}H NMR (300 MHz, $CDCl_3$) spectra for compound **18.**

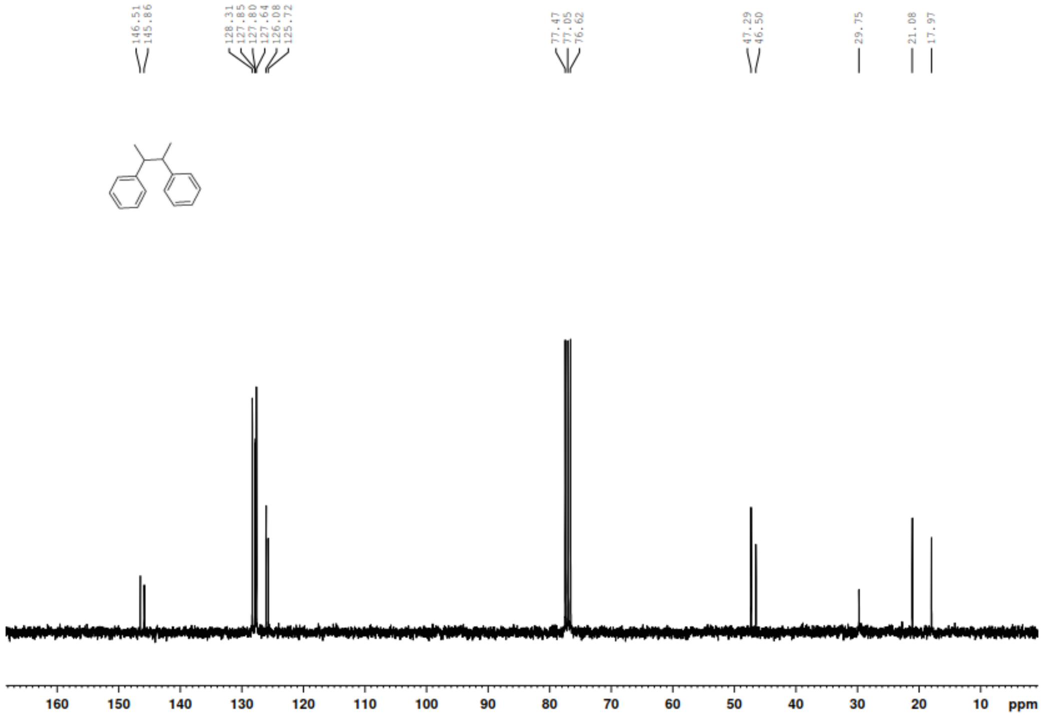

Supplementary Figure 243. ^{13}C NMR (75 MHz, $CDCl_3$) spectra for compound **18.**

Supplementary References.

1. Groendyke, B. J., AbuSalim, D. I. & Cook, S. P. Iron-catalyzed, fluoroamide-directed C–H fluorination. *J. Am. Chem. Soc.* **138**, 12771-12774 (2016).

2. Xie, J., Yu, J., Rudolph, Rominger, M., F. & Hashmi, A. S. K. Monofluoroalkenylation of dimethylamino compounds through radical-radical cross-coupling. *Angew. Chem. Int. Ed.* **55**, 9416 –9421 (2016).

3. Chen, W., Liu, Z., Tian, J., Li, J., Ma, J., Cheng, X. & Li, G. Building congested ketone: substituted Hantzsch ester and nitrile as alkylation reagents in photoredox catalysis. *J. Am. Chem. Soc.* **138**, 12312-12315 (2016).

4. Kim, H. & Chang, S. Iridium-catalyzed direct C−H amination with alkylamines: facile oxidative insertion of amino group into iridacycle. *ACS Catal*. **5**, 6665−6669 (2015).

5. Li, S., Wang, B., Dong, G., Li, C. & Liu, H. Cobalt-catalyzed C(sp^3)–H/C(sp^2)–H oxidative coupling between alkanes and benzamides. *RSC Adv.* **8**, 13454–13458 (2018).

6. Zhao, Y., Huang, B., Yang, C., Li, B., Gou, B., & Xia, W. Photocatalytic cross-dehydrogenative amination reactions between phenols and diarylamines. *ACS Catal.* **7**, 2446−2451 (2017).

7. Jin, J. & MacMillan, D. W. C. Alcohols as alkylating agents in heteroarene C–H functionalization. *Nature*, **525**, 87-90 (2015).

8. Lowry, M. S., Goldsmith, J. I., Slinker, J. D., Rohl, R., Pascal, R. A., Malliaras, G. G. & Bernhard, S. Single-layer electroluminescent devices and photoinduced hydrogen production from an ionic iridium(III) complex. *Chem. Mater*. **17**, 5712−5719 (2005).

9. Slinker, J. D., Gorodetsky, A. A., Lowry, M. S., Wang, J., Parker, S., Rohl, R., Bernhard, S. & Malliaras, G. G. Efficient yellow electroluminescence from a single layer of a cyclometalated iridium complex. *J. Am. Chem. Soc*. **126**, 2763−2767 (2004).
